# Whole-genome *de novo* sequencing reveals unique genes that contributed to the adaptive evolution of the Mikado pheasant

**DOI:** 10.1093/gigascience/giy044

**Published:** 2018-05-02

**Authors:** Chien-Yueh Lee, Ping-Han Hsieh, Li-Mei Chiang, Amrita Chattopadhyay, Kuan-Yi Li, Yi-Fang Lee, Tzu-Pin Lu, Liang-Chuan Lai, En-Chung Lin, Hsinyu Lee, Shih-Torng Ding, Mong-Hsun Tsai, Chien-Yu Chen, Eric Y. Chuang

**Affiliations:** 1 Graduate Institute of Biomedical Electronics and Bioinformatics, National Taiwan University, Taipei 10617, Taiwan; 2 Bioinformatics and Biostatistics Core, Center of Genomic Medicine, National Taiwan University, Taipei 10055, Taiwan; 3 Department of Bio-Industrial Mechatronics Engineering, National Taiwan University, Taipei 10617, Taiwan; 4 Institute of Plant and Microbial Biology, Academia Sinica, Taipei, 11529, Taiwan; 5 Institute of Epidemiology and Preventive Medicine, National Taiwan University, Taipei 10055, Taiwan; 6 Graduate Institute of Physiology, National Taiwan University, Taipei 10051, Taiwan; 7 Department of Animal Science and Technology, National Taiwan University, Taipei 10617, Taiwan; 8 Department of Life Science, National Taiwan University, Taipei 10617, Taiwan; 9 Center for Biotechnology, National Taiwan University, Taipei 10672, Taiwan; 10 Institute of Biotechnology, National Taiwan University, Taipei 10672, Taiwan; 11 Agricultural Biotechnology Research Center, Academia Sinica, Taipei 11529, Taiwan University, Taipei, Taiwan; 12 Center for Systems Biology, National Taiwan University, Taipei 10672, Taiwan; 13 Graduate Institute of Chinese Medical Science, China Medical University, Taichung 40402, Taiwan

**Keywords:** Mikado pheasant, *Syrmaticus mikado*, long-tailed pheasant, whole-genome sequencing, *de novo* genome assembly, adaptive evolution

## Abstract

**Background:**

The Mikado pheasant (*Syrmaticus mikado*) is a nearly endangered species indigenous to high-altitude regions of Taiwan. This pheasant provides an opportunity to investigate evolutionary processes following geographic isolation. Currently, the genetic background and adaptive evolution of the Mikado pheasant remain unclear.

**Results:**

We present the draft genome of the Mikado pheasant, which consists of 1.04 Gb of DNA and 15,972 annotated protein-coding genes. The Mikado pheasant displays expansion and positive selection of genes related to features that contribute to its adaptive evolution, such as energy metabolism, oxygen transport, hemoglobin binding, radiation response, immune response, and DNA repair. To investigate the molecular evolution of the major histocompatibility complex (MHC) across several avian species, 39 putative genes spanning 227 kb on a contiguous region were annotated and manually curated. The MHC loci of the pheasant revealed a high level of synteny, several rapidly evolving genes, and inverse regions compared to the same loci in the chicken. The complete mitochondrial genome was also sequenced, assembled, and compared against four long-tailed pheasants. The results from molecular clock analysis suggest that ancestors of the Mikado pheasant migrated from the north to Taiwan about 3.47 million years ago.

**Conclusions:**

This study provides a valuable genomic resource for the Mikado pheasant, insights into its adaptation to high altitude, and the evolutionary history of the genus *Syrmaticus*, which could potentially be useful for future studies that investigate molecular evolution, genomics, ecology, and immunogenetics.

## Background

The Mikado pheasant (*Syrmaticus mikado*), which is a long-tailed pheasant indigenous to Taiwan, belongs to the family *Phasianidae* in the order Galliformes (Additional file 1: Fig. S1A, B). The Mikado pheasant is known to inhabit a variety of habitats in the mountainous regions of central and southern Taiwan at very high elevations ranging from 1,600 to 3,500 meters [1, 2]. The Mikado pheasant faced endangerment due to hunting pressure and habitat destruction [3, 4] until it became protected under the Wildlife Conservation Act. Currently, the International Union for Conservation of Nature Red List has classified the Mikado pheasant as a nearly threatened species, showing a decreasing trend in the overall population, with an estimate of approximately 15,000 mature birds. The rare and precious Mikado pheasant is a national icon in Taiwan and is depicted on its 1000 dollar banknote.

The *de novo* genome assembly of endangered species is an effective approach to identify genomic signatures associated with environmental adaptation and behavioral attributes [5, 6]. Genome resources can also provide great insights into effective population size, genetic defects, and deleterious mutations [7, 8]. Moreover, reconstruction of a phylogenetic tree can reveal genetic relationships and evolutionary history [9-11]. Together, they can lead to the conservation and rescue of endangered species.

The Mikado pheasant possesses ideal characteristics for evolutionary research because of its flightlessness and habitat isolation. It is one of five long-tailed pheasants in the *Syrmaticus* genus, which forms a monophyletic group [12]. Due to limited molecular data, very few studies have been conducted to investigate the phylogenetic relationships and divergence time of species within the genus. Moreover, the Mikado pheasant is mainly found in Yushan National Park [13], which has numerous extremely high mountains that exceed an altitude of 3,000 meters (Additional file 1: Fig. S2). As high altitudes are associated with extremely cold climates and lower oxygen concentrations, hypoxic stress may be observed in the pheasant. Considering its importance as a species facing endangerment, the present unavailability of genetic information regarding the Mikado pheasant motivated the *de novo* assembly of its genome, followed by a detailed study of its genetic background and subsequent adaptive evolution.

Here, we report the whole-genome assembly of the Mikado pheasant and provide insights into its adaptive mechanisms. This genome-wide study reveals the evolutionary adaptation of the Mikado pheasant to high altitudes, including changes in gene family size and/or molecular signatures of positive selection associated with energy metabolism, oxygen transport, hemoglobin binding, radiation response, immune response, and DNA repair. The estimated time of divergence among the five long-tailed pheasant species reconstructs the evolutionary history of the lineage and allows us to propose a hypothesis for the biogeographical speciation of the Mikado pheasant. Additionally, the manually curated major histocompatibility complex (MHC) gene loci of the Mikado pheasant display evidence for molecular evolution with a high level of synteny, mainly across inverse regions in gene blocks, and several rapidly evolving genes in comparison with the chicken.

## Data Description

The details about sample collection, library construction, sequencing, assembly, gene prediction, and annotation can be found in the Materials and Methods section.

## Results

### Genome assembly and annotation

In total, 171.7 Gb of raw DNA sequence reads (Additional file 1: Table S1) were generated, resulting in an approximately 160-fold sequencing coverage based on the 1.07 Gb genome size estimated by KmerGenie [14]. The contigs were built and assembled into a 1.04 Gb sequence of the draft genome. The N50 lengths of the contigs and scaffolds were 13.46 kb and 11.46 Mb, respectively. The overall GC content of the Mikado pheasant genome was 41.13%, which is similar to that of the chicken, duck, turkey, and zebra finch (Additional file 1: Fig. S3). The size of the longest assembled sequence was 50.28 Mb, and 928 scaffolds were longer than 10 kb. The basic statistics of both the contigs and scaffolds assembled using MaSuRCA [15] are shown in Table 1. The cumulative length plots (Additional file 1: Fig. S4A, B) and the Nx plot for the scaffolds (Additional file 1: Fig. S5) showed that most of the draft genome consisted of large scaffolds; though many short scaffolds were present, they only contributed a small portion of the genome size.

**Table 1:** DNA contigs and scaffolds from the genomic data of the Mikado pheasant

	Contigs	Scaffolds
Total length	1,054,607,905	1,035,947,982
Maximum length	195,342	50,275,205
Number of Ns	0	19,577,473
Average length	5,050	110,714
N50*	13,461	11,461,115
N75*	6,528	5,708,287
L50^†^	22,195	28
L75^†^	50,081	59
Counts ≥300 bp	208,810	-
Counts ≥1 kb	123,006	9,357
Counts ≥5 kb	61,237	1,489
Counts ≥10 kb	32,868	928

* The N50/N75 length is defined as the shortest sequence length at 50%/75% of the genome.

† The L50/L75 count is defined as the smallest number of contigs (or scaffolds) that those length sum produces N50/N75.

Values of the genome assembly were calculated using the contigs ≥300 bp and scaffolds ≥1000 bp.

Before performing gene prediction and annotation, the interspersed and low complexity regions were first masked using RepeatMasker (RepeatMasker, RRID:SCR_012954) [16]. Approximately 8.91% of the sequences were identified as interspersed repeats, 1.32% of the sequences were identified as long tandem repeat elements, and overall 11.46% of the total bases were identified (Additional file 1: Table S2). After masking the repeats and extrinsic data, an *ab initio* gene prediction was performed using Augustus (Augustus: Gene Prediction, RRID:SCR_008417) [17], followed by EVidenceModeler [18]. The final gene models comprised 27,254 transcripts (proteins). Of the predicted proteins, 15,972 (58.6%) could be strictly aligned to the National Center for Biotechnology Information (NCBI) nonredundant (NR) protein database for Aves and Reptilians. The statistics of annotated genes in the Mikado pheasant averaged 19.9 kb per gene, 1,625 bp per coding DNA sequence, 164.1 bp per exon, and 2,053 bp per intron (Additional file 1: Table S3), which are similar composition of gene elements in length to other avian species [19]. Of the 15,972 NR annotated proteins, 14,124 proteins were well annotated to the Pfam domains. A total of 5,626 Pfam domains were identified based on our predictions.

### Assessment of the assembly quality

The overall DNA mapping rate of the paired-end libraries was >90% for the concordant paired read alignment and >96% for both paired and single read alignment (Additional file 1: Table S4). Thus, the assembly utilized most of the DNA reads. We further examined the per-base alignment coverage. The results (Additional file 1: Fig. S6) showed that most of the genome positions had a coverage between approximately 57- and 121-fold and an average sequence coverage of 88-fold, which is very similar to the sequencing depth of DNA paired-end libraries (98.7x). Thus, our draft genome is well assembled.

To evaluate the quality of the assembled genome [20], the RNA reads were mapped onto the draft genome. The overall alignment rate of both RNA libraries showed that approximately >93% of the reads could be aligned to the scaffolds, indicating that most of the expressed protein-coding genes could be found in the draft genome (Additional file 1: Table S5). Moreover, the Benchmarking Universal Single-Copy Orthologs (BUSCO [BUSCO, RRID:SCR_015008]) [21] benchmark was used to evaluate the genes predicted from the genome assembly (Additional file 1: Table S6). Of the 3,023 single-copy orthologs in the vertebrate lineage, approximately 88.6% of the orthologs were found in our assembly, which is similar to the results obtained in duck (88.6%), turkey (87.5%), and zebra finch (88.8%). These results suggest that a potentially large number of genes, along with their complete structure, could be predicted from the genome.

### Genome comparison

**Figure 1:**
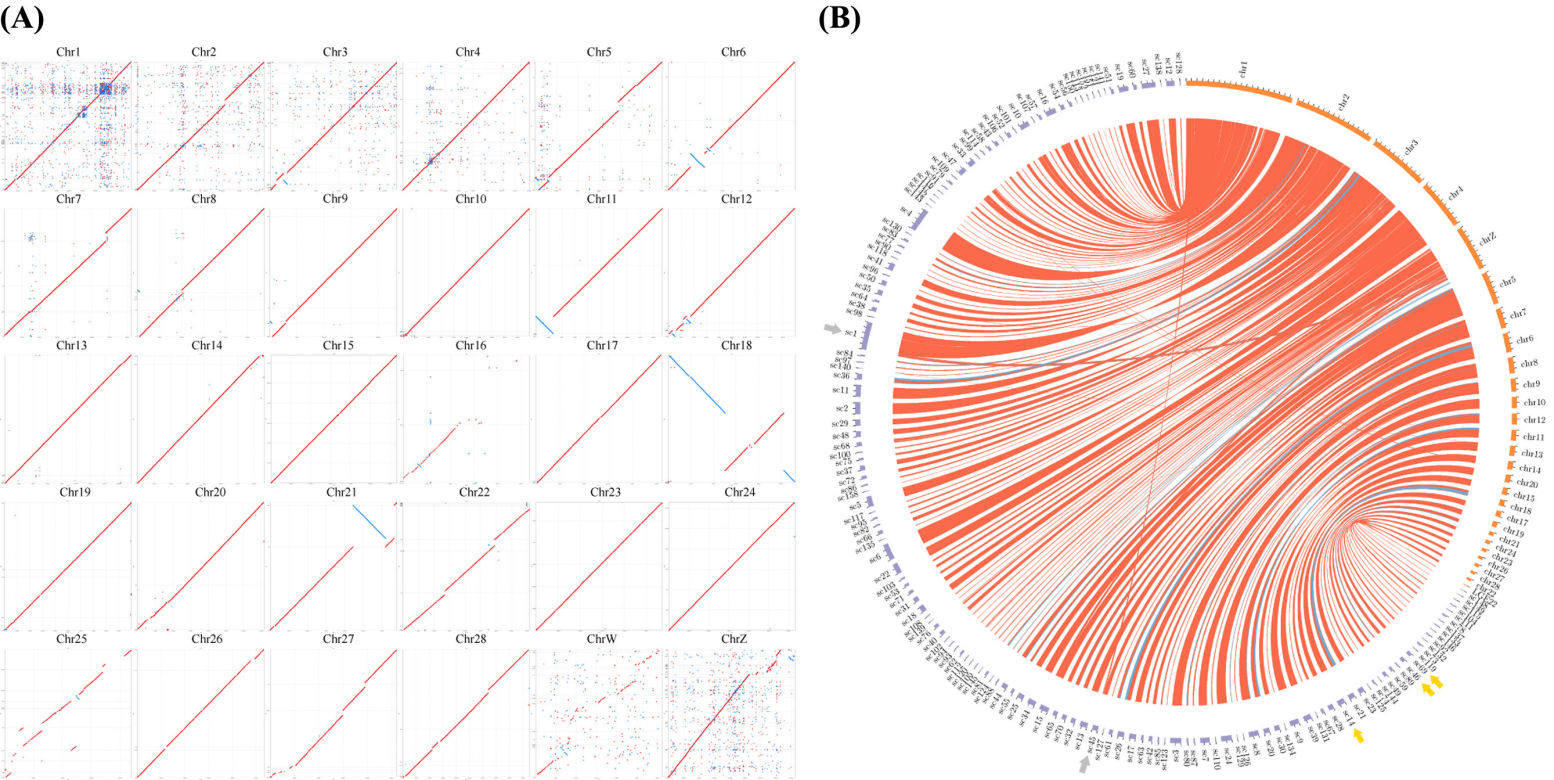
A chromosome-level comparison of the Mikado pheasant and the chicken. **(A)** A syntenic map of the Mikado pheasant and chicken genomes. The *x*-axis specifies the chromosome position in the chicken, whereas the *y*-axis specifies the scaffold position in the Mikado pheasant. The red dots (or lines) indicate that the sequences were aligned in the same orientation, and the blue dots indicate an alignment with a reverse complement. **(B)** A chord diagram of scaffolds with a total length greater than 500 kb and an alignment length greater than 10 kb. The orange perimeters specify the chromosomes (chr) of the chicken, whereas the purple perimeters specify the scaffolds (sc) of the Mikado pheasant. The red links represent the sequences aligned in the same orientation, and the blue links represent an alignment with a reverse complement. Yellow arrows indicate the scaffolds that were fully aligned, and gray arrows indicate the multiple alignment.

To understand the similarities between the Mikado pheasant and the chicken at the genomic level, assembled scaffolds that were longer than 0.25% of the aligned chicken chromosome were selected and plotted onto a syntenic map with an alignment length of at least 3 kb using MUMmer [22]. The identities of each chicken chromosome with the scaffolds of Mikado pheasant were between 86.24% and 89.98%, and the overall coverage was 85.28% (i.e., 855.35 Mb of the assembled scaffolds could be mapped onto the chicken genome; Additional file 1: Table S7). The syntenic relationships between the Mikado pheasant scaffolds and the chicken chromosomes were highly conserved, but a few of the chromosomes could be only partially aligned. In particular, three well-assembled scaffolds, i.e., scaffold14, scaffold69, and scaffold46, were mapped to nearly the full length of chicken chromosomes 15, 23, and 24, respectively. Notably, compared to the scaffolds of the Mikado pheasant, the chicken chromosomes, including chromosomes 6, 11, 18, and 21, were properly aligned but with obvious inversions (Fig. 1A). More stringent conditions were then considered in order to evaluate the alignment of certain scaffolds to multiple chromosomes (e.g., scaffold1 and scaffold45; Fig. 1B); however, further confirmation is required to determine whether this finding represents the actual presence of chromosomal translocations in the Mikado pheasant genome. Additionally, the alignment between the Mikado pheasant scaffolds and the turkey chromosomes provided similar results (Additional file 1: Fig. S7A), but the Mikado pheasant scaffolds were poorly aligned with the zebra finch chromosomes (Additional file 1: Fig. S7B). In general, the Mikado scaffolds displayed high conservation with the genomes of chicken and turkey. We also observed several intrachromosomal inversions and chromosomal translocations. This is the first genome-wide analysis to identify multiple intrachromosomal inversions between the Mikado pheasant and chicken genomes.

### Phylogenetic relationships of the Mikado pheasant

**Figure 2:**
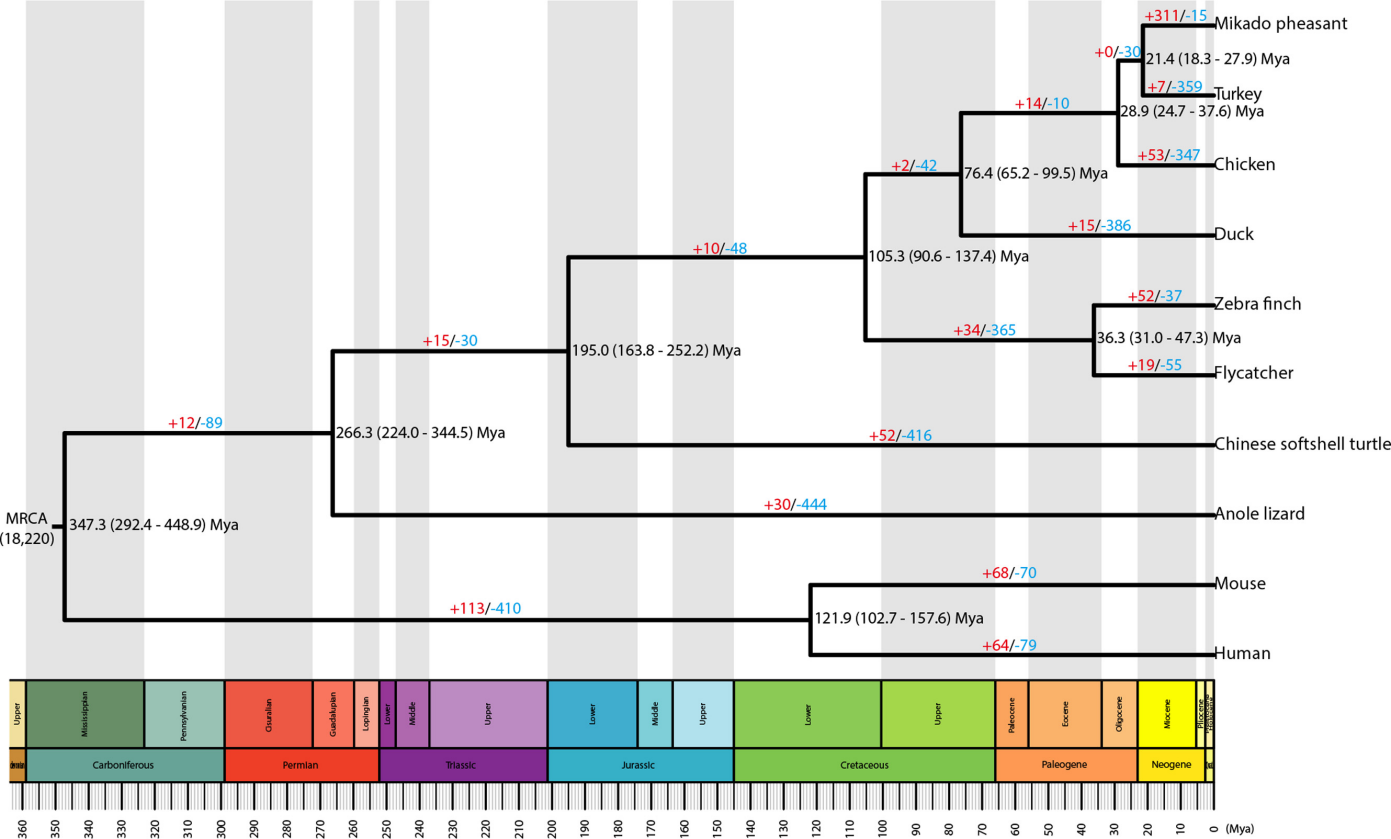
Evolution of gene families among various animal species. A phylogenetic tree was reconstructed based on 5,287 single-copy orthologs of 10 species. The most recent common ancestor (MRCA) contains 18,220 gene families that were used to examine gene families with expansions or contractions. The numbers of gene families with significant expansions and contractions are shown in red and blue, respectively, at each branch. The divergence times and associated 95% confidence intervals (in parentheses) are indicated at the nodes of the tree in Mya. All nodes had 100% support in 500 bootstrap replicates.

To compare the protein sequences of the Mikado pheasant against homologous protein families of other birds and organisms, OrthoMCL (OrthoMCL DB: Ortholog Groups of Protein Sequences, RRID:SCR_007839) [23] was used to define the gene families in 10 species. Proteins with sequences that were similar to those of the Mikado pheasant—five birds (i.e., chicken, duck, flycatcher, turkey, and zebra finch), two reptiles (anole lizard and Chinese softshell turtle), and two mammals (human and mouse)—were classified into each gene family. In this way, we obtained 18,220 gene families from 10 species. Next, 5,287 single-copy orthologs that were common across these species were used to construct a Bayesian maximum clade credibility phylogenetic tree and to estimate the time of divergence [24] (Fig. 2). The estimated time of the Mikado pheasant-turkey divergence was 21.4 million years ago (Mya); the divergence time between chicken and the sister clade of the Mikado pheasant-turkey was estimated at 28.9 Mya. In the Galliformes order, the Mikado pheasant was found to be more closely related to the turkey than to the chicken. The branches of the Galliformes and duck (76.4 Mya), Passeriformes and Galliformes (105.3 Mya), and anole lizard and Aves (266.3 Mya) displayed divergence times that were similar to those reported in the literature [25-27].

### Gene family evolution

To assess the changes in the gene family sizes, a likelihood model was used to examine significant expansions and contractions of gene families, particularly in the Mikado pheasant lineage. Expansions or contractions in gene families indicate that total numbers of genes in a gene family are increasing or decreasing, respectively. The results revealed 311 expanded and 15 contracted gene families compared with the common ancestor of the Mikado pheasant and turkey (Fig. 2). In total, 86 gene ontology (GO) categories were significantly enriched (*p* < 0.05, empirical test) among the 311 expanded genes. Fifty of these GO categories were further classified into eight main categories, including actin cytoskeleton, morphogenesis, catalytic activity, cell differentiation, binding, metabolism, cytoplasm, and organelle organization and biogenesis (Additional file 2: Table S8). In particular, the gene families involved in oxygen and heme binding (GO:0019825 and GO:0020037, respectively), monooxygenase activity (GO:0004497), and energy metabolism (GO:0046034, ATP metabolic process; GO:0005977, glycogen metabolic process) were substantially expanded in the Mikado pheasant. Conversely, 7 of the 25 GO categories in the contracted gene families were significantly enriched in immune system processes and apoptosis (Additional file 1: Table S9). From the Pfam database [28], 8 of the 75 expanded gene families were annotated as olfactory receptors (Additional file 2: Table S10).

### Positive selection

To detect the genes that evolved rapidly due to positive selection under the influence of high elevation (Mikado pheasant) as opposed to low elevation (chicken, turkey, duck, and zebra finch), 7,132 single-copy orthologs were analyzed from 9,038 genes common across the five species (Additional file 1: Fig. S8). According to the branch-site model and the likelihood ratio test, the 889 positively selected genes (PSGs) identified in the Mikado pheasant were mainly enriched in functions such as metabolism (GO:0008152), cell (GO:0005623), and binding (GO:0005488) that belong to biological process, cellular component, and molecular function ontology terms, respectively. We further examined the PSGs involved in metabolism, which constituted the largest number of PSGs and GO functions (Additional file 1: Fig. S9). The 45 PSGs enriched in metabolism-related functions (*p*-values < 0.05) were classified according to the GOSlim categories into lipid metabolism (GO:0006629), carbohydrate metabolic processes (GO:0005975), and generation of precursor metabolites and energy (GO:0006091), which included 13, 3, and 2 GO functions, respectively (Additional file 2: Table S11). Of these metabolism-related PSGs, four genes were found to be involved in the inositol phosphate metabolism (map00562; *p*-value < 0.01) and phosphatidylinositol signaling system (map04070; *p*-value < 0.05) through a functional enrichment analysis from the Kyoto Encyclopedia of Genes and Genomes (KEGG [KEGG, RRID:SCR_012773]) database (Additional file 1: Table S12).

In addition to metabolism, other high-altitude adaptations were observed, such as response to radiation (GO:0010212, response to ionizing radiation; GO:0010332, response to gamma radiation; GO:0034644, cellular response to ultraviolet radiation; and GO:0071480, cellular response to gamma radiation), DNA repair (GO:0000731, DNA synthesis involved in DNA repair; GO:0045739, positive regulation of DNA repair; and GO:0006284, base-excision repair), and oxygen transport (GO:0016706, oxidoreductase activity; GO:0072593, reactive oxygen species metabolic process; GO:0019825, oxygen binding; and GO:2000377, regulation of reactive oxygen species metabolic process; Additional file 2: Table S13). Moreover, 43 PSGs in the Mikado pheasant were significantly enriched in the categories of lymphocyte activation (GO:0046649; including eight GO terms) and cytokine production (GO:0001816; including eight GO terms; Additional file 2: Table S14). We also identified the janus kinase/signal transducer and activator of transcription (Jak-STAT) signaling pathway (map04630; *p*-value < 0.05), which was enriched in five PSGs (i.e., *BCL2*, *CCND3*, *IL12RB2*, *IL23R*, and *IL7*), in the KEGG analysis (Additional file 1: Table S15).

### Identification of the MHC-B region of the Mikado pheasant

**Figure 3:**
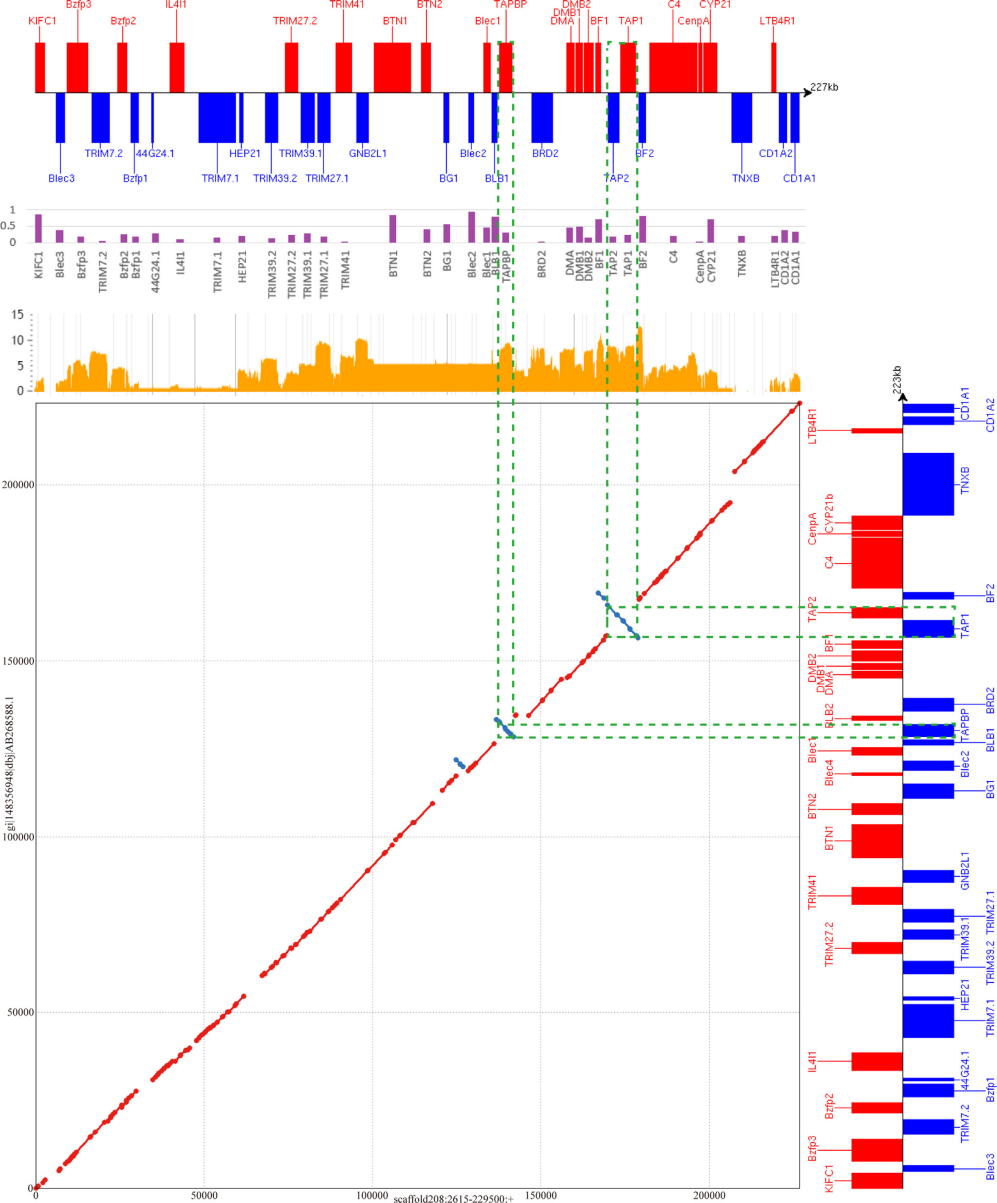
An identity plot of the MHC regions of the Mikado pheasant and the chicken. The chicken MHC sequence was downloaded from GenBank (AB268588). Its nucleotide sequence from 17,978 to 241,251 was aligned against the Mikado pheasant MHC sequence from 2,615 to 229,500 in scaffold208. The gene structure boxes on the horizontal and vertical axes, respectively, represent the gene loci in the Mikado pheasant and the chicken. Boxes with different sizes exhibit different gene locus sizes, and red/blue coloring indicates genes in forward/reverse orientation. The red dots (or lines) on the diagonal indicate that the sequences were aligned in the same orientation, whereas the blue dots indicate alignments with reverse complements. The green dotted lines highlight the sequence of the inverted *TAPBP* locus and *TAP1*-*TAP2* block. The orange peaks show the read counts on a natural log scale of the gene expression based on our RNA-seq data. The box plot colored in purple indicates *d_N_/d_S_* ratios of genes.

The MHC is a cluster of genes that are associated with functions such as infectious disease resistance and immune responses in all jawed vertebrates [29]. The MHC B-locus (MHC-B) performs the main MHC functions in the chicken [30, 31]. Based on the above analysis, an assembled scaffold (scaffold208) was almost able to cover the known chicken sequence of the MHC-B contiguous region published by Shiina et al. (GenBank accession: AB268588.1) [32] (Fig. 3). To understand the evolution of the MHC-B genes between the Mikado pheasant and the chicken, the predicted gene loci were manually curated by incorporating evidence from the aligned RNA-sequencing (RNA-seq) data and homologous genes from chicken and turkey using Web Apollo software [33]. After the curation, 39 putative MHC genes of the Mikado pheasant were identified within a 227 kb sequence (Table 2) including seven MHC class II loci (*BLB1*, *TAPBP*, *BLB2*, *BRD2*, *DMA*, *DMB1*, and *DMB2*), four MHC class I loci (*BF1*, *TAP1*, *TAP2*, and *BF2*), and five MHC class III loci (*C4*, *CenpA*, *CYP21*, *TNXB*, and *LTB4R1*).

**Table 2:** Coding sequences of MHC-B genes in the Mikado pheasant and comparisons with the chicken

Mikado pheasant						Chicken					
Gene	Position	Strand	Gene length	Amino acid length	Exon	Aligned base	Nucleotide identity (%)	Aligned amino acid	Amino acid identity (%)	Amino acid substitutions	d_N_/d_S_*
KIFC1	2615–5304	+	1,140	380	7	1,131	91.76	377	90.53	33	0.8669
Blec3	8997–11 221	-	552	183	5	507	82.43	168	78.14	25	0.3821
Bzfp3	12 126–18 213	+	1,449	482	13	1,569	85.8	522	83.62	40	0.1884
TRIM7.2	19 507–24 562	-	1,518	505	7	1,518	95.98	505	98.61	7	0.0391
Bzfp2^†^	27 027–29 946	+	1,368	455	4	1,396	70.41	N/A	N/A	N/A	0.2438
Bzfp1	31 049–33 298	-	1,425	474	2	1,426	88.23	471	86.79	54	0.1900
44G24.1	37 266–37 673	-	408	136	1	408	85.78	136	80.15	27	0.2762
IL4I1	42 730–46 759	+	1,578	525	6	1,572	92.25	523	93.75	25	0.1011
TRIM7.1	51 325–62 131	-	1,758	585	8	1,767	92.49	588	92.69	40	0.1545
HEP21	63 362–64 247	-	324	107	3	324	93.52	107	91.59	9	0.2148
TRIM39.2	70 980–74 640	-	1,392	464	6	1,389	93.68	463	94.61	24	0.1167
TRIM27.2	76 988–80 522	+	1,431	476	7	1,431	94.13	476	92.23	37	0.2415
TRIM39.1	81 560–85 449	-	798	266	5	798	93.23	266	91.35	23	0.2753
TRIM27.1	86 518–90 228	-	1,485	495	7	1,485	94.48	495	94.34	28	0.1715
TRIM41	91 918–96 605	+	1,656	551	7	1,770	89.58	589	91.71	7	0.0375
GNB2L1	98 038–101 512	-	954	317	8	954	96.86	317	100	0	N/A
BTN1	103 411–114 264	+	930	309	8	939	74.64	339	57.26	96	0.8357
BTN2	117 466–120 157	+	1,461	487	7	1,481	90.41	469	83.37	52	0.3996
BG1	124 105–125 436	-	549	183	3	546	91.99	182	87.98	21	0.5591
Blec2	131 358–133 021	-	579	192	5	579	86.32	190	71.88	52	0.9375
Blec1	135 818–137 846	+	567	188	5	567	92.59	188	88.3	22	0.4683
BLB1	138 411–139 729	-	339	112	3	345	83.38	113	42.98	62	0.7904
TAPBP	140 657–144 216	+	1,293	430	8	1,293	92.19	430	89.77	44	0.3179
BLB2^‡^	N/A	N/A	792	263	N/A	792	92.93	263	85.93	37	1.4489
BRD2	150 146–156 295	-	2,976	991	13	3,078	86.85	776	75.28	30	0.0306
DMA	160 545–162 778	+	789	263	4	789	92.65	263	89.73	27	0.4528
DMB1	163 010–165 184	+	930	310	6	930	91.29	310	86.45	42	0.4978
DMB2	165 617–168 363	+	768	256	5	768	92.71	256	92.58	19	0.1622
BF1	169 254–170 740	+	996	331	5	1,001	83.66	345	64.12	95	0.7116
TAP2	172 793–176 021	-	2,100	700	9	2,100	92.48	700	93.14	48	0.1675
TAP1	176 574–180 981	+	1,752	584	11	1,739	93.21	580	92.81	38	0.2191
BF2	181 900–184 038	-	1,530	509	6	1,213	62.28	326	57.39	119	0.8157
C4	185 102–199 258	+	5,031	1,676	40	4,998	93.33	1,665	93.2	101	0.1974
CenpA	199 593–200 795	+	396	131	4	396	96.72	131	99.24	1	0.0324
CYP21	201 291–205 141	+	1,431	477	11	1,431	92.67	477	94.13	28	0.7109
TNXB	209 524–215 604	-	2,472	824	10	2,496	92.14	832	92.34	50	0.2002
LTB4R1	221 450–222 538	+	1,089	363	1	1,089	94.12	363	94.49	20	0.1954
CD1A2	223 740–225 788	-	1,044	348	6	1,044	92.24	348	87.93	42	0.3796
CD1A1	227 030–229 500	-	1,122	374	6	1,122	93.4	374	90.64	35	0.3294

KIFC1, kinesin family member C1; Blec, C-type lectin-like receptor; Bzfp, B-locus zinc finger-like protein; TRIM, tripartite motif containing protein; 44G24.1, histone H2B-like protein; IL4I1, interleukin 4 induced 1; HEP21, hen egg protein 21 kDa; GNB2L1, guanine nucleotide binding-like protein; BTN, B-butyrophilin protein; BG1, BG-like antigen; CD1A1/A2, CD1-like proteins.

* d_N_/d_S_ = ratio of nonsynonymous (d_N_) to synonymous (d_S_) substitutions.

† Defined as a pseudogene in chicken.

‡ No predicted result was identified from the DNA assembly. The transcript sequence was alternatively derived from the transcriptome assembly by RNA-seq.

Gene loci involved in immunity have been shown to have a higher ratio of nonsynonymous (*d_N_*) to synonymous (*d_S_*) amino acid substitutions due to interactions with rapidly evolving pathogens under selective pressures [34-36]. *KIFC1*, *BTN1*, *Blec2*, *BLB1*, *BLB2*, and *BF2* had comparatively high *d_N_/d_S_* ratios between the Mikado pheasant and the chicken (Table 2). Conversely, the genes with comparatively lower *d_N_/d_S_* ratios included *TRIM7.2*, *TRIM41*, *BRD2*, and *CenpA*. As shown in Fig. 3, the Mikado pheasant and the chicken displayed similarity in the MHC-B region and shared an almost perfect syntenic gene order. Notably, no *BLB2* genes were predicted between the *TAPBP* and *BRD2* intergenic regions in the Mikado pheasant MHC-B locus; however, these regions could be detected among the transcripts of our RNA-seq data. A likely explanation for the absence of a prediction of the BLB2-like gene might be the existence of two unsequenced gap regions with a size of 1,098 bp within the *TAPBP*-*BRD2* block (5,931 bp). Since *BLB2* is only 792 bp in length, it could reside within the missing sequence. Based on the RNA-seq results, 2.54 million reads were mapped onto 38 MHC-B genes (except for *BLB2*) of the Mikado pheasant, 27 of which had at least a 1-fold average coverage per nucleotide. Furthermore, 15 genes possessed more than 100-fold average coverage per nucleotide, providing concrete evidence of a reliable prediction. Intriguingly, two gene loci, i.e., *TAPBP* and the *TAP1*-*TAP2* block, were inversely oriented compared to the chicken sequence.

### Evolutionary history of *Syrmaticus* pheasants

**Figure 4:**
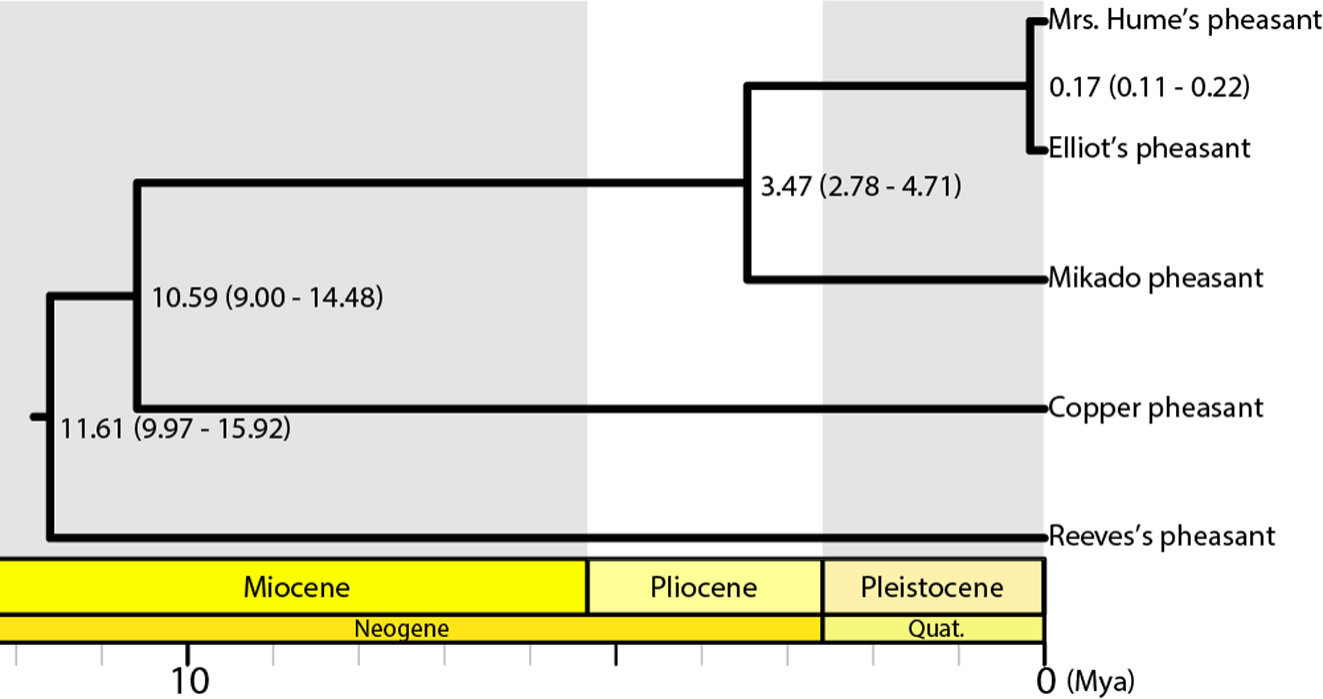
A phylogenetic tree of *Syrmaticus* pheasants. The divergence times and associated 95% confidence intervals shown in parentheses are given at the branch nodes of the tree in million years ago.

The mitochondrial genome of the Mikado pheasant was assembled based on the short-read libraries. The circular complete genome had a total length of 16,680 bp, including 13 protein-coding genes, 2 rRNAs, 22 tRNAs, and a control region (Additional file 1: Table S16). The average nucleotide composition was 30.52% A, 31.20% C, 13.44% G, and 24.84% T. To investigate the evolutionary history of the genus *Syrmaticus*, which includes five long-tailed pheasants, the phylogeny was reconstructed, and the divergence times were estimated using the mitochondrial genomes. According to molecular clock analysis, the genetic divergence of the Mikado pheasant began approximately 3.47 (2.78–4.71) Mya (Fig. 4). The tree topology is consistent with previous studies [12, 37], and the divergence time suggests that the Mikado pheasant might have originated in the late Pliocene.

### Amino acid substitution analysis in Mikado pheasant hemoglobin genes

Living at high elevations directly incurs the challenge of low oxygen availability. Additionally, exposure to low-pressure environments causes oxygen saturation in the arterial blood, thus decreasing and restricting oxygen supplementation to tissues [38]. Certain birds show an increased combined affinity between blood and oxygen via amino acid substitutions in the major hemoglobin [39-41]. To investigate their role in adaptation to high-altitude environments, amino acid substitutions were examined in the Mikado pheasant hemoglobin sequences. By comparing six avian species, an amino acid substitution with different consensus residues was found in the Mikado pheasant (Additional file 1: Fig. S10), and the substitution of alanine with threonine occurred at residue 78 of the alpha-A subunit—the major component of hemoglobin isoforms. The Andean goose, a kind of waterfowl that lives at more than 3,000 meters in the Andes, has been reported to carry the identical substitution [42].

## Discussion

In this study, experimental data and statistical approaches were used to evaluate the genome assembly of the Mikado pheasant. Notably, the genome sequence of this species was previously unknown, and this study provides a comparative analysis of various genomes using a large number of tools at different stages for the assembly of the Mikado pheasant genome. While conducting the genome assembly, we used not only MaSuRCA but also assembly tools such as ALLPATHS-LG [43], JR [44], Newbler [45], SGA [46], and SOAPdenovo [47]. All of these assembly tools produced similar draft genome sizes, and MaSuRCA and SGA also showed similar results in terms of the N50 value and the scaffold number (Additional file 1: Table S17). To facilitate the downstream analysis, we used several methods to compare these assembly sets. However, no single assembly tool outperformed the others in terms of the number of annotations for the predicted genes, the quality of the genome compared to that of other birds, and the BUSCO benchmark. In this study, the draft genome assembled using MaSuRCA was selected because it generated dramatically longer scaffolds that displayed a decent score on the BUSCO benchmark and produced proper annotations for the predicted genes. Although scaffolds of the draft genome displayed some degree of fragmentation (Fig. 1A) and showed translocation (Fig. 1B) in certain chicken chromosomes, our approach still provides a practical strategy for whole-genome assembly using only short-read sequencing technology. We assert that the high coverage of our sequencing data, differing library insert sizes, and the use of a combination of tools, such as MaSuRCA and SSPACE for assembly and scaffolding, respectively, contributed to high-quality *de novo* assembly of the Mikado pheasant genome with a genome length of approximately 1 Gb.

Recent studies have reported phylogenetic tree topologies for the Mikado pheasant and other Galliformes birds [37, 48, 49]; however, these studies relied on small amounts of genomic DNA as supporting evidence. To obtain a highly accurate phylogenetic inference, long DNA sequences are necessary for the reconstruction of a high-resolution tree [50-52]. This study used whole-exome information, with 5,287 single-copy orthologs totaling approximately 8 Mb of coding sequence, to reconstruct the phylogeny and estimate the divergence time among the Mikado pheasant and other birds (Fig. 2). Our results strongly suggest that the Mikado pheasant is more similar to the turkey than the chicken in the Galliformes clade, which is consistent with previous studies [37, 48, 49].

We additionally implemented a comprehensive phylogenetic analysis strategy to obtain information regarding the adaptive mechanisms of the Mikado pheasant to high elevations. Compared to birds that live at low altitudes, both the positive gene selection and gene expansion analyses showed a significant enrichment of genes relevant to energy metabolism (Additional file 2: Tables S8 and S11). This finding was consistent with the prior study that identified similar genes in other species that inhabit the highlands [53]. Moreover, the four metabolism-related PSGs (i.e., *INPP5A*, *INPP5J*, *PI4KB*, and *PLCE1*) that were involved in the inositol phosphate metabolism and phosphatidylinositol signaling system (Additional file 1: Table S12) were previously reported to be enriched in Tibetan pigs living at high altitudes [54]. Of these genes, *INPP5A* and *INPP5J* play a role in the hydrolysis of inositol polyphosphates [55], *PI4KB* is a phosphatidylinositol kinase that induces phosphorylation reactions [56], and *PLCE1*, which is a phospholipase enzyme, regulates gene expression, cell growth, and differentiation [57]. Another robust signal of its adaptation to high altitude was obtained from genes significantly associated with expansion of and positive selection for the enhancement of hemoglobin binding and oxygen transport (Additional file 2: Tables S8 and S13). Furthermore, for both the Mikado pheasant and Andean goose, an amino acid substitution was identified in the hemoglobin alpha-A subunit (Additional file 1: Fig. S10). The substitution of threonine at this position has recently been shown to cause an increase in the molecular volume, which might enhance the solubility of hemoglobin and facilitate adaptation to desiccating and high-altitude environments [42]. Through gene expansion, the genes of the Mikado pheasant that are involved in skeletal and cardiac muscle fiber development (Additional file 2: Table S8) and the enhanced functions of the additional GO terms implied that the biomass of the Mikado pheasant could be effectively produced in mountainous regions without nourishment, hence strongly suggesting the existence of an adaptive mechanism for high altitudes [58]. Finally, the PSGs in the radiation response, immune response, and DNA repair categories (Additional file 2: Tables S13 and S14) may reflect the increased resistance of the Mikado pheasant to long-term ultraviolet radiation exposure through the induction of cytokine production [59] and lymphocyte activation [60] and DNA repair processes. Some of these PSGs were also involved in the Jak-STAT signaling pathway (Additional file 1: Table S15), which participates in chemical signal transmission and induces cellular stress responses, such as immunity, apoptosis [61, 62], and hypoxia [63]. All of these results provide wider support for the adaptive evolution of the Mikado pheasant. To sum up, this study reveals the high-altitude adaptation mechanisms of the Mikado pheasant at the genomic level. However, there are some adaptive mechanisms for high altitude that happen via changes in regulatory regions that modulate the levels of gene expression [64-66]. We believe that this is an intriguing topic and worthy of further research to be undertaken in the future.

In this work, we annotated and curated the MHC-B gene loci in the Mikado pheasant, which is important for assessing the adaptive mechanisms associated with endangered species, because variations in gene number in the MHC cluster could be caused by exposure to pathogens or diseases [67, 68]. The genome of the Mikado pheasant contains a number of MHC-B genes, and inversions were observed in the *TAPBP* locus and the *TAP1*-*TAP2* block (Fig. 3) compared to the chicken genome; an inverse orientation of the *TAP1*-*TAP2* block was also detected compared to the turkey genome (Additional file 1: Fig. S11). A similar conversion at the MHC locus in Galliformes has been reported in previous studies [29, 34, 69]. We further observed a Blec2-like sequence with an inverse orientation located within the *BG1*-*Blec2* region in the Mikado pheasant. We inferred that this region is likely similar to the *Blec4* pseudogene of the chicken and highly similar to *Blec2* [32].

In this study, we not only sequenced the whole genome of a bird of the *Syrmaticus* genus but also completed the full mitochondrial genome. Before whole-genome sequences were available, mitochondrial sequences were widely utilized in molecular phylogenetic analyses of the genus of *Gallus* [70, 71]. Based on the assembly of the Mikado pheasant and the other four available sequences, we reconstructed a phylogenetic tree and provide a completely sequenced mitochondrial genome for five long-tailed pheasants. The topology of our reconstructed tree (Fig. 4) is consistent with results from a previous study [12]. However, the time of divergence was estimated to be earlier than the previously reported time [12] for the Mikado pheasant, which might have been due to the use of a few mitochondrial or nuclear genes rather than the complete mitochondrial genome. The reconstructed tree showed a potential migration pathway of these pheasants. The ancestors of the Mikado pheasant, which have been described to have migrated to the island of Taiwan, separated from the lineage of the copper pheasant (*Syrmaticus soemmerringii ijimae*). The copper pheasant is a pheasant indigenous to Japan, whose ancestors might have separated from the lineage of the Reeves's pheasant (*Syrmaticus reevesii*) that has inhabited northern China. The ancestors of Elliot's pheasant (*Syrmaticus ellioti*) and Mrs. Hume's pheasant (*Syrmaticus humiae*) have branched from the Mikado pheasant, then separated into two present kinds of pheasants that have alternatively roosted in the mountainous forests of southeastern and southwestern China, respectively. According to paleogeographical reports, Taiwan was formed approximately 4–5 Mya and attained its modern topography approximately 3 Mya [72]. The sea level was lower during the glacial periods, and Taiwan might have been connected to the mainland [73]. Our results suggest that the evolutionary history of the Mikado pheasant might have included ancestors that migrated from the north toward Taiwan approximately 3.47 Mya and consequently were isolated by the Taiwan Strait during the warm interglacial periods during the early Pleistocene.

Currently, there is no nuclear genome data available for the copper pheasant, so unfortunately, incorporating all five long-tailed pheasants into our analysis using nuclear genomes is impossible at present. For the other four pheasants, however, Wang et al. [37] used six nuclear intron and two mitochondrial gene sequences to construct a phylogenetic tree, and its topology was consistent with our result. Our estimate of the divergence time was more precise, considering that we employed complete mitochondrial genomes in the reconstruction of a high-resolution tree for the *Syrmaticus* genus instead of a few mitochondrial genes. Our estimated divergence time is also supported by the paleogeographical report of Taiwan island formation. Despite these corroborations of the proposed tree topology and estimated divergence time, the use of only mitochondrial data may be considered as a potential limitation. Going forward, it will be necessary to analyze the nuclear genome to obtain further insights into the evolution history of long-tailed pheasants.

## Materials and Methods

### Sample preparation and sequencing

Blood samples were collected from a single female Mikado pheasant living in central Taiwan; then, genomic DNA was extracted, and two paired-end libraries (280 bp and 480 bp; average read length, 151 bp) and five mate pair libraries (1, 3, 5, 7, and 10 kb; average read length, 101 bp) were constructed according to the manufacturer's protocol. In addition, two RNA-seq libraries from two male Mikado pheasants’ blood samples were prepared for the purpose of draft genome assessment and gene prediction (Additional file 1: Table S1). The DNA libraries were sequenced using the HiSeq platform (Illumina Inc., San Diego, CA, USA), and the RNA libraries were sequenced using the HiScanSQ and HiSeq platforms.

### *De novo* genome assembly

The quality of the raw reads was examined using FastQC (FastQC, RRID:SCR_014583), version 0.10.1. Trimmomatic (Trimmomatic, RRID:SCR_011848), version 0.30 (parameters: “ILLUMINACLIP: TruSeq3-PE.fa:2:30:15 SLIDINGWINDOW:4:20 MINLEN:100”) [74] and NextClip (version 1.3.1) [75] with default parameters were used to trim sequencing reads. Genome assembly into contigs was performed using MaSuRCA (version 2.3.2) [15] with settings based on the instruction manual. ALLPATHS-LG (ALLPATHS-LG, RRID:SCR_010742, version 49722) [43], Newbler (version 2.9) [45] both with default parameters, JR (version 1.0.4; parameters: “-minOverlap 60 -maxOverlap 90 -ratio 0.3”) [44], SGA (version 0.10.13; parameters: “assemble -m 125 -d 0.4 -g 0.1 -r 10 -l 200”) [46], and SOAPdenovo (version 2.04; parameters: “-K 47 -R”) [47] were also used to assemble contigs. We employed SSPACE (SSPACE, RRID:SCR_005056, version 3.0; parameter: “-z 300”) [76] to construct scaffolds for the draft genome. In this step, mate pair libraries with 35 bases from the 5' end of both reads were used for scaffolding. Scaffold sequences shorter than 300 bp were then excluded from the final assembly. The statistical results of the assembly were estimated using QUAST (version 3.2) [77].

To examine sequencing reads for potential contamination, we used Kraken (version 1.0) [78] with the standard Kraken database to check the paired-end DNA libraries. Classified reads reported by Kraken were further examined using our proposed pipeline (Additional file 1: Fig. S12). Briefly, we employed Bowtie 2 (Bowtie, RRID:SCR_005476; version 2.3.0) [79] to align these classified reads against the chicken genome reference (Galgal 5.0) downloaded from Ensembl (release 90), collecting unmapped reads and using Bowtie 2 again to align them against the assembled genome of the Mikado pheasant. We then took those reads mapped onto the Mikado pheasant genome and performed Basic Local Alignment Search Tool N (BLASTN) alignment against the nonredundant nucleotide sequences database, downloaded from NCBI's FTP site (on Nov. 16, 2017), using parameters “-outfmt “6 std staxids” -max_target_seqs 1 -evalue 1E-10.” Next, we collected reads with alignment length ≥100 bp (i.e., two thirds of read length), filtering out the reads that matched an avian species or with a read count <50 in a species. The remaining reads were counted and the contaminated scaffolds calculated by applying a cutoff of a read count >20 on a given scaffold. Finally, we removed 31 contaminated scaffolds with 12,587 bp (∼0.001% of the total length) from the assembled genome.

### Evaluation of assembly quality

Several metrics were used to evaluate the assembly quality, including the number and length distribution of the scaffold sequences, the mapping rate of the paired-end DNA reads and RNA reads, the per-base coverage of the DNA read mapping, and the coverage of universal single-copy orthologs provided by BUSCO (version 1.21). To evaluate the mapping rate of the reads and per-base coverage, the paired-end DNA reads and RNA reads were aligned against the assembled scaffolds using Bowtie 2 (version 2.2.4) and STAR [80], respectively. Briefly, scaffolds were mainly assembled from the paired-end DNA reads; the higher mapping rate of the paired-end DNA reads suggests a higher degree of the final assembly covering the raw reads. Taking the RNA sequencing reads from two individual Mikado pheasants and observing the mapping rate is another approach for assessing the completeness of the assembly. The per-base DNA read coverage was calculated using BEDTools (BEDTools, RRID:SCR_006646), version 2.23.0 [81]. For each base, the expected coverage should be close to the sequencing depth of the paired-end reads (approximately 98.7x). The BUSCO benchmark is a single-copy ortholog set derived from the species of a major lineage. The gene models predicted from the draft genome in the Mikado pheasant were compared with the lineage of vertebrates (3,023 single-copy orthologs in total) provided by BUSCO. Protein sequences from the chicken, duck, turkey, and zebra finch were also evaluated for comparison.

### Genome comparison

To compare the genome of the Mikado pheasant with that of other avian species, we retrieved the whole-genome sequences of the chicken (Galgal4), turkey (UMD2), and zebra finch (taeGut3.2.4) from the Ensembl database. Using the genome-wide sequence aligner MUMmer (version 3.23), the chromosome-level differences and similarities among the species were investigated and visualized. The structural variants among the species were further reported using the “show-diff” utility in MUMmer. The chord diagrams of the alignment were generated using Circos [82].

### Gene prediction and annotation

First, RepeatMasker (version 4.0.5; parameter: “-species chicken”), including rmblastn (version 2.2.23+) as the search engine, RepBase (version 20140131), and RM database (version 20140131), were applied to screen the scaffolds for interspersed repeats and low-complexity regions in the DNA sequences, and the masked genome was used for further gene prediction. Then, homology-based, RNA-seq, and *ab initio* prediction approaches were used to identify protein-coding genes and build a consensus gene set that included all predicted genes. For the homology protein sequence alignment, the protein sequences of the chicken (Galgal4), turkey (UMD2), duck (BGI_duck_1.0), and zebra finch (taeGut3.2.4) were collected from Ensembl. The protein sequence alignments were performed using Exonerate (version 2.2.0) [83]. All RNA-seq reads were aligned against the repeat-masked genome using TopHat2 [84], which generated evidence of splice sites, introns, and exons. Additionally, Trinity (Trinity, RRID:SCR_013048), version 2.0.6, [85], was utilized to assemble transcripts, and PASA (version 2.0.0) [86] was used to group alternatively spliced isoforms. For the *ab initio* gene prediction, the standard Augustus (version 3.0.3) pipeline was used to yield potentially predicted genes with evidence from both homologous proteins and RNA-seq. Next, the consensus gene set was determined by consolidating the three types of gene prediction using EVidenceModeler (version 1.1.1). Finally, the gene annotations were defined based on the best sequence alignment against NCBI NR proteins in Aves and Reptilians using BLASTP (version 2.2.29+), with the following criteria: identity ≥ 30%,alignment length ≥80 bp, and E-value ≤ 1e−5. For the protein domain identification, we annotated the domains using HMMER (version 3.1b2) [87] by scanning the Pfam database (version 30.0).

For MHC-B annotation and curation, we first took the scaffold208 sequence and used MAKER (version 2.31.8) [88] to predict the potential gene structures of MHC-B genes. Next, the RNA-seq libraries from the Mikado pheasant and the homologous protein sequences from chicken and turkey were aligned to these predicted regions. Finally, we used Web Apollo (version 2.0.3), a web-based and visualization tool for curation and annotation, to manually curate these genes according to the alignment evidence.

### Gene families

To identify gene families, the protein-coding genes of five birds (i.e., *Gallus gallus, Meleagris gallopavo, Anas platyrhynchos, Taeniopygia guttata*, and *Ficedula albicollis*) and four additional species (*Anolis carolinensis, Pelodiscus sinensis, Homo sapiens*, and *Mus musculus*) were downloaded from Ensembl (release 82). The sequence of the longest isoform was selected to represent the gene for each species, despite the presence of protein isoforms. The all-vs-all BLASTP was applied to align all protein sequences (including those of the Mikado pheasant) of the 10 species and 5 birds (excluding flycatcher) with E-value thresholds less than 1e−5 and 1e−20, respectively. Then, 18,220 gene families (including 5,287 single-copy orthologs) were obtained from the 10 species, and 13,436 gene families (including 7,132 single-copy orthologs) were obtained from the 5 birds by OrthoMCL (version 2.0.9) using default parameters. In the analysis of the 10 species, 15,161 genes of the Mikado pheasant were grouped into 12,549 gene families. In the analysis of the five avian species, 14,375 Mikado pheasant genes were grouped into 12,078 gene families. Next, MUSCLE (MUSCLE, RRID:SCR_011812), version 3.8.1551 [89], was used with default parameters for the multiple sequence alignment of the converted coding DNA sequences from single-copy orthologs, and Gblocks (version 0.91b; parameters: “-t = d -b4 = 5 -b5 = h -e = _cln”) [90] was used to remove the poorly aligned regions. After trimming, the genes from each species were concatenated using the same order to reconstruct the phylogenies and evaluate the divergence time. The concatenated sequences were used to build a phylogenetic tree using RAxML (RAxML, RRID:SCR_006086), version 8.2.4 [91], via a maximum likelihood search with 500 bootstrap replicates; then, the divergence time was analyzed using BEAST (BEAST, RRID:SCR_010228), version 2.3.2, with the GTR+I+Γ model, which is the best substitution model selected by Modeltest (version 3.7) and PAUP* (version 4.0a150) [92]. Four nodes were chosen as the fossil calibration points from the TimeTree database [93], including human-chicken (311.9 Mya), anole lizard-chicken (279.7 Mya), Chinese softshell turtle-chicken (253.7 Mya), and human-mouse (89.8 Mya). The phylogenetic tree was generated using the Strap R package [94]. To identify the gene families with an expansion or contraction between the Mikado pheasant and other species, CAFE (version 3.1) [95] was used to estimate the rates of gene family evolution from the observed gene numbers in each family and the given phylogenetic tree. A *p*-value <0.05 was used to indicate significant changes in the gene family size.

### Examination of genes under positive selection and enrichment analysis

To determine the genes that underwent positive natural selection in the Mikado pheasant, CODEML from PAML (PAML, RRID:SCR_014932), version 4.8 [96], was applied to the branch-site model to investigate the genes in positively selected sites of the Mikado pheasant. For the branch-site model, we implemented likelihood ratio tests to determine the statistical significance of positive selection for testing a null model (model = 2, NSsites = 2, fix_omega = 1, and omega = 1) against an alternative model (model = 2, NSsites = 2, and fix_omega = 0). Consequently, the false discovery rates (FDRs) were computed with a cutoff of 0.05 to adjust for multiple testing using the Benjamini-Hochberg procedure.

The GO annotations of four birds (i.e., chicken, duck, turkey, and zebra finch) retrieved from the Ensembl BioMart were used to characterize the functions of the identified orthologs. A hypergeometric test was performed to identify significant GO functions in these orthologs. However, the raw *p*-values of the hypergeometric tests can easily be affected by the number of genes [97]; therefore, to address the underlying bias of the hypergeometric distribution, we further calculated empirical *p*-values [98]. The empirical *p*-values were determined through 100K simulated datasets by ranking the hypergeometric probability of enriched functional categories compared with the null baseline probabilities. The null baseline probability was established by randomly selecting a group of genes containing an equal number of PSGs with an FDR <0.05 for the branch-site model. For massively enriched GO terms with similar functions, CateGOrizer [99] was used to classify the genes into basic categories. ClueGO [100] with the hypergeometric test and a Bonferroni adjustment were performed to enrich the KEGG pathways [101].

### Mitochondrial genome assembly

Geneious (version 8.1.5) [102] was utilized with the default settings to assemble the whole mitochondrial genome. First, the reads were mapped to the four available *Syrmaticu*s mitochondrial genomes from GenBank (AB164622.1 - AB164625.1). The mapped reads were collected and then used for the further assembly of the mitochondrial genome of the Mikado pheasant. The genes were identified using MITOS [103] and curated by comparison with known sequences of other long-tailed pheasants from GenBank. The phylogenetic reconstruction and estimation of the divergence times among the five long-tailed pheasants were achieved using BEAST with the GTR+G model, which was selected as the best nucleotide substitution model by Modeltest and PAUP*. We added two nodes as the fossil calibration points according to the TimeTree database, including Elliot's pheasant-Reeves's pheasant (11.1 Mya) and Elliot's pheasant-Mrs. Hume's pheasant (0.2 Mya). A calibrated Yule speciation process was implemented in the analysis using BEAST. In the Markov chain Monte Carlo analysis, the chain length utilized 10 million generations.

## Availability of supporting data

Data for the *S. mikado* genome has been deposited in the GenBank/EMBL/DDBJ Bioproject database under the project number PRJNA389983. Raw genomic and transcriptomic sequence datasets were deposited in the Sequence Read Archive under the accession number SRP108966. Other supporting data, including the draft genome, annotations, alignments, phylogenetic trees, and scripts, are available via the *GigaScience* repository, GigaDB [104].

## Ethics approval and consent to participate

All experimental procedures and sample collection methods in this study involving Mikado pheasants were conducted according to the Wildlife Conservation Act (amendment on July 8, 2009, Taiwan) and were approved by the Council of Agriculture, Executive Yuan, Taipei, Taiwan with issue 1021700417.

## Additional files

Additional file 1: Supplementary Figures S1-S12 and supplementary Tables S1-S7, S9, S12, and S15-S17. Additional file 2: Supplementary Tables S8, S10-S11, and S13-S14.

## Abbreviations

BLAST: Basic Local Alignment Search Tool; BUSCO: Benchmarking Universal Single-Copy Orthologs; FDR: false discovery rate; GO: gene ontology; KEGG: Kyoto Encyclopedia of Genes and Genomes; MHC: major histocompatibility complex; Mya: million years ago; NCBI: National Center for Biotechnology Information; NR: nonredundant; PSG: positively selected gene; RNA-seq: RNA-sequencing.

## Supplementary Material

GIGA-D-17-00220_Original_Submission.pdf

GIGA-D-17-00220_Revision_1.pdf

Response_to_Reviewer_Comments_Original_Submission.pdf

Reviewer_1_Report_(Original_Submission) -- Matthew Greenwold 10/11/2017 Reviewed

Reviewer_2_Report_(Original_Submission) -- Aurelie Kapusta, Ph.D. 10/30/2017 Reviewed

Reviewer_3_Report_(Original_Submission) -- Lél Eöry 11/1/2017 Reviewed

Reviewer_3_Report_(Revision_1) -- Lél Eöry 3/13/2018 Reviewed

Additional Files
