# Supplementary material for: Whole-genome de novo sequencing reveals unique genes that contributed to the adaptive evolution of the Mikado pheasant
Source: Gigascience. 2018 May 2;7(5):giy044. doi: 10.1093/gigascience/giy044 (PMC5941149; doi:10.1093/gigascience/giy044)
Supplement: GIGA-D-17-00220_Original_Submission.pdf [file giy044_giga-d-17-00220_original_submission.pdf]

GigaScience

Whole-Genome De Novo Sequencing Reveals Unique Genes that Contributed to the Adaptive Evolution of the Mikado Pheasant

--Manuscript Draft--

**Manuscript Number:**	GIGA-D-17-00220
**Full Title:**	Whole-Genome De Novo Sequencing Reveals Unique Genes that Contributed to the Adaptive Evolution of the Mikado Pheasant
**Article Type:**	Research
**Funding Information:**	Taipei Zoo (No. 13, 2015 Animal Adoption Programs of Taipei Zoo) — Dr. Eric Y. Chuang
**Abstract:**	Background: The Mikado pheasant (Syrmaticus mikado) is a nearly endangered species indigenous to high-altitude regions of Taiwan. It possesses a unique position in evolution because of its geographic isolation. Currently, the genetic background and adaptive behaviors of the Mikado pheasant remain unclear.<br>Results: We present the draft genome of the Mikado pheasant, which consists of 1.04 Gb of DNA and 15 972 annotated protein-coding genes. The Mikado pheasant displays expansion and positive selection of genes related to features that contribute to its adaptive evolution, such as energy metabolism, oxygen transport, hemoglobin binding, radiation response, immune response, and DNA repair. The major histocompatibility complex (MHC) region contains 39 putative genes within 227 kb of DNA. Compared with the chicken MHC, we not only found that TAPBP and the TAP1-TAP2 block are in inverse orientation, but also identified some genes undergoing rapid evolution. The complete mitochondrial genome was further sequenced, assembled, and compared against 4 other long-tailed pheasants. The results from molecular clock analysis suggest that ancestors of the Mikado pheasant migrated from the north to Taiwan about 3.47 million years ago.<br>Conclusions: This study provides a valuable genomic resource for the Mikado pheasant, insights into its adaptation to high altitude, and the evolutionary history of the genus Syrmaticus, which could potentially be useful for future studies investigating molecular evolution, genomics, and immunogenetics.
**Corresponding Author:**	Eric Y. Chuang<br><br>TAIWAN
**Corresponding Author Secondary Information:**	
**Corresponding Author's Institution:**	
**Corresponding Author's Secondary Institution:**	
**First Author:**	Chien-Yueh Lee
**First Author Secondary Information:**	
**Order of Authors:**	Chien-Yueh Lee<br>Ping-Han Hsieh<br>Li-Mei Chiang<br>Amrita Chattopadhyay<br>Kuan-Yi Li<br>Yi-Fang Lee<br>Tzu-Pin Lu<br>Liang-Chuan Lai<br>En-Chung Lin

Powered by Editorial Manager® and ProduXion Manager® from Aries Systems Corporation

	Hsinyu Lee
	Shih-Torng Ding
	Mong-Hsun Tsai
	Chien-Yu Chen
	Eric Y. Chuang
**Order of Authors Secondary Information:**	
**Opposed Reviewers:**	
**Additional Information:**	
**Question**	**Response**
Are you submitting this manuscript to a special series or article collection?	No
**Experimental design and statistics**<br><br>Full details of the experimental design and statistical methods used should be given in the Methods section, as detailed in our Minimum Standards Reporting Checklist. Information essential to interpreting the data presented should be made available in the figure legends.<br><br>Have you included all the information requested in your manuscript?	Yes
**Resources**<br><br>A description of all resources used, including antibodies, cell lines, animals and software tools, with enough information to allow them to be uniquely identified, should be included in the Methods section. Authors are strongly encouraged to cite Research Resource Identifiers (RRIDs) for antibodies, model organisms and tools, where possible.<br><br>Have you included the information requested as detailed in our Minimum Standards Reporting Checklist?	Yes
**Availability of data and materials**<br><br>All datasets and code on which the conclusions of the paper rely must be either included in your submission or deposited in publicly available repositories (where available and ethically appropriate), referencing such data using a unique identifier in the references and in the “Availability of Data and Materials”	Yes

section of your manuscript.<br><br>Have you have met the above requirement as detailed in our Minimum Standards Reporting Checklist?	

Manuscript Click here to download Manuscript Manuscript.docx

Whole-Genome *De Novo* Sequencing Reveals Unique Genes that Contributed to the Adaptive Evolution of the Mikado Pheasant

Short title: The Mikado Pheasant Genome and Adaptive Evolution

Chien-Yueh Lee[1†], Ping-Han Hsieh[1†], Li-Mei Chiang[1], Amrita Chattopadhyay[2], Kuan-Yi Li[3,4], Yi-Fang Lee[1], Tzu-Pin Lu[5], Liang-Chuan Lai[6], En-Chung Lin[7], Hsinyu Lee[1,8,9], Shih-Torng Ding[7,9], Mong-Hsun Tsai[2,9,10,11*], Chien-Yu Chen[3,9,12*], and Eric Y. Chuang[1,2,5,9,13*]

[1]Graduate Institute of Biomedical Electronics and Bioinformatics, National Taiwan University, Taipei 10617, Taiwan

[2]Bioinformatics and Biostatistics Core, Center of Genomic Medicine, National Taiwan University, Taipei 10055, Taiwan

[3]Department of Bio-Industrial Mechatronics Engineering, National Taiwan University, Taipei 10617, Taiwan

[4]Institute of Plant and Microbial Biology, Academia Sinica, Taipei, 11529, Taiwan

[5]Institute of Epidemiology and Preventive Medicine, National Taiwan University, Taipei 10055, Taiwan

[6]Graduate Institute of Physiology, National Taiwan University, Taipei 10051, Taiwan

[7]Department of Animal Science and Technology, National Taiwan University, Taipei 10617, Taiwan

[8]Department of Life Science, National Taiwan University, Taipei 10617, Taiwan

[9]Center for Biotechnology, National Taiwan University, Taipei 10672, Taiwan

[10]Institute of Biotechnology, National Taiwan University, Taipei 10672, Taiwan

[11]Agricultural Biotechnology Research Center, Academia Sinica, Taipei 11529, Taiwan University, Taipei, Taiwan

[12]Center for Systems Biology, National Taiwan University, Taipei 10672, Taiwan

[13]Graduate Institute of Chinese Medical Science, China Medical University, Taichung 40402, Taiwan

[†] These authors contributed equally to the work.

[*] Corresponding authors
Eric Y. Chuang
Department of Electrical Engineering, Graduate Institute of Biomedical Electronics and Bioinformatics, National Taiwan University, Taipei 10617, Taiwan
Phone: +886-2-3366-3660, Fax: +886-2-3366-3682, E-mail: chuangey@ntu.edu.tw

Chien-Yu Chen
Department of Bio-Industrial Mechatronics Engineering, National Taiwan University, Taipei 10617, Taiwan
Phone: +886-2-3366-5334, E-mail: chienyuchen@ntu.edu.tw

Mong-Hsun Tsai
Institute of Biotechnology, National Taiwan University, Taipei, Taiwan
Phone: +886-2-3366-6009, E-mail: motiont@ntu.edu.tw

E-mail addresses

Chien-Yueh Lee: d00945006@ntu.edu.tw
Ping-Han Hsieh: r04945025@ntu.edu.tw
Li-Mei Chiang: dytk2134@gmail.com
Amrita Chattopadhyay: amrita@ntu.edu.tw
Kuan-Yi Li: kyli.tw@gmail.com
Yi-Fang Lee: b01901110@ntu.edu.tw
Tzu-Pin Lu: tbenlu@gmail.com
Liang-Chuan Lai: llai@ntu.edu.tw
En-Chung Lin: eclin@mail2000.com.tw
Hsinyu Lee: hsinyu@ntu.edu.tw
Shih-Torng Ding: sding@ntu.edu.tw
Mong-Hsun Tsai: motiont@ntu.edu.tw
Chien-Yu Chen: chienyuchen@ntu.edu.tw
Eric Y. Chuang: chuangey@ntu.edu.tw

Abstract

Background: The Mikado pheasant (*Syrmaticus mikado*) is a nearly endangered species indigenous to high-altitude regions of Taiwan. It possesses a unique position in evolution because of its geographic isolation. Currently, the genetic background and adaptive behaviors of the Mikado pheasant remain unclear.

Results: We present the draft genome of the Mikado pheasant, which consists of 1.04 Gb of DNA and 15 972 annotated protein-coding genes. The Mikado pheasant displays expansion and positive selection of genes related to features that contribute to its adaptive evolution, such as energy metabolism, oxygen transport, hemoglobin binding, radiation response, immune response, and DNA repair. The major histocompatibility complex (MHC) region contains 39 putative genes within 227 kb of DNA. Compared with the chicken MHC, we not only found that *TAPBP* and the *TAP1-TAP2* block are in inverse orientation, but also identified some genes undergoing rapid evolution. The complete mitochondrial genome was further sequenced, assembled, and compared against 4 other long-tailed pheasants. The results from molecular clock analysis suggest that ancestors of the Mikado pheasant migrated from the north to Taiwan about 3.47 million years ago.

Conclusions: This study provides a valuable genomic resource for the Mikado pheasant, insights into its adaptation to high altitude, and the evolutionary history of the genus *Syrmaticus*, which could potentially be useful for future studies investigating molecular evolution, genomics, and immunogenetics.

Keywords: Mikado pheasant, *Syrmaticus mikado,* long-tailed pheasant, whole-genome sequencing, *de novo* genome assembly, adaptive evolution

Background

The Mikado pheasant (*Syrmaticus mikado*), which is a long-tailed pheasant indigenous to Taiwan, belongs to the family *Phasianidae* in the order Galliformes (Additional file 1: Fig. S1A, B). The Mikado pheasant is known to inhabit a variety of habitats in the mountainous regions of Central and Southern Taiwan at very high elevations ranging from 1600 to 3500 meters [1, 2]. The Mikado pheasant faced endangerment due to hunting pressure and habitat destruction [3, 4] until it became protected under the Wildlife Conservation Act. Currently, the International Union for Conservation of Nature (IUCN) Red List has classified the Mikado pheasant as a nearly threatened species, showing a decreasing trend in the overall population with a total estimate of approximately 15 000 mature birds. The rare and precious Mikado pheasant is a national icon in Taiwan and is depicted on its 1000 dollar banknote.

The *de novo* genome assembly of endangered species is an effective approach for the identification of genomic signatures associated with environmental adaptation and behavioral attributes. Genome studies have assisted in the discovery of genetic defects and deleterious mutations, and phylogenetic reconstruction can reveal genetic relationships and evolutionary history, thus leading to the conservation and rescue of endangered species [5-9]. The Mikado pheasant possesses a unique position in evolution because of its geographic isolation. It is one of 5 long-tailed pheasants in the *Syrmaticus* genus and belongs to a monophyletic group [10]. Due to limited molecular data, very few studies have been conducted to investigate the phylogenetic relationships and divergence time of species within the genus. Moreover, the Mikado pheasant is mainly found in Yushan National Park [11], which has numerous extremely high mountains exceeding an altitude of 3000 meters (Additional file 1: Fig. S2). As high altitudes are associated with extremely cold climates and lower

concentrations of oxygen, hypoxic stress is observed in the pheasant. Considering its importance as a species facing endangerment, the present unavailability of genetic information regarding the Mikado pheasant motivated the *de novo* assembly of its genome, followed by a detailed study of its genetic background and subsequent adaptive behaviors.

Here we report the whole-genome assembly of the Mikado pheasant and provide insights into the adaptive mechanisms of the pheasant. This genome-wide study reveals the evolutionary adaptation of the Mikado pheasant to high altitudes, including changes in gene family size and/or molecular signatures of positive selection associated with energy metabolism, oxygen transport, hemoglobin binding, radiation response, immune response, and DNA repair. The estimated time of divergence among the 5 long-tailed pheasant species reconstructs the evolutionary history of the lineage and allows us to propose a hypothesis for the biogeographical speciation of the Mikado pheasant. Additionally, in comparison with the chicken, the curated major histocompatibility complex gene loci of the Mikado pheasant display a high level of synteny, mainly across inverse regions in 2 gene blocks, and several rapidly evolving genes.

Data Description

Genome assembly and annotation

In total, 171.7 Gb of raw DNA sequence reads (Additional file 1: Table S1) were generated, resulting in an approximately 160-fold sequencing coverage based on the 1.07 Gb genome size estimated by KmerGenie [12]. After filtering the low-quality reads and removing the read adapters, the high-quality reads were used to build contigs. The contigs were assembled into a 1.04 Gb sequence of the draft genome.

The N50 lengths of the contigs and scaffolds were 13.46 kb and 11.32 Mb, respectively. The overall GC content of the Mikado pheasant genome was 41.13%, which is similar to that of the chicken, duck, turkey, and zebra finch (Additional file 1: Fig. S3). The size of the longest assembled sequence was 50.28 Mb, and 928 scaffolds were longer than 10 kb. The basic statistics of both the contigs and scaffolds assembled using MaSuRCA [13] are shown in Table 1. The cumulative length plots (Additional file 1: Fig. S4A, B) and the Nx plot for the scaffolds (Additional file 1: Fig. S5) showed that almost all the scaffolds were assembled with very long lengths, but only a small number of short sequences were present in the scaffolds.

Before performing the gene prediction and annotation, the interspersed and low complexity regions were first masked using RepeatMasker [14]. Approximately 8.91% of the sequences were identified as interspersed repeats, 1.32% of the sequences were identified as long tandem repeat (LTR) elements, and overall 11.46% of the total bases were identified (Additional file 1: Table S2). After masking the repeats and extrinsic data, an *ab initio* gene prediction was performed using Augustus [15], followed by EVidenceModeler [16]. The final gene models comprised 27 255 transcripts (proteins). Of the predicted proteins, 15 972 (58.6%) could be strictly aligned to the National Center for Biotechnology Information (NCBI) non-redundant (NR) protein database for Aves and Reptilians. The statistics of annotated genes in the Mikado pheasant averaged 19.9 kb per gene, 1625 bp per coding DNA sequence (CDS), 164.1 bp per exon, and 2053 bp per intron (Additional file 1: Table S3), which are similar composition in length to other avian species [17]. Out of the 15 972 NR annotated proteins, 14 124 proteins were well annotated to the Pfam domains. A total of 5626 Pfam domains were identified based on our predictions.

Results

Assessment of the assembly quality

The overall DNA mapping rate of the paired-end libraries was >90% for the concordant paired read alignment and >96% for both paired and single read alignment (Additional file 1: Table S4). Thus, the assembly utilized most of the DNA reads. We further examined the per-base alignment coverage. The results (Additional file 1: Fig. S6) showed that most of the genome positions had a coverage between approximately 57- and 121-fold and an average sequence coverage of 88-fold, which is very similar to the sequencing depth of DNA paired-end libraries (98.7x). Thus, our draft genome is well assembled.

To evaluate the quality of the assembled genome [18], the RNA reads were mapped onto the draft genome. The overall alignment rate of both RNA libraries showed that approximately 80% of the reads could be concordantly aligned to the scaffolds, indicating that most of the expressed protein-coding genes could be found in the draft genome (Additional file 1: Table S5). Moreover, the BUSCO [19] benchmark was used to evaluate the genes predicted from the genome assembly (Additional file 1: Table S6). Of the 3023 single-copy orthologs in the vertebrate lineage, approximately 88.6% of the orthologs were found in our assembly, which is similar to the results obtained in duck (88.6%), turkey (87.5%), and zebra finch (88.8%). These results suggested that a potentially large number of genes, along with their complete structure, could be predicted from the genome.

Genome comparison

To understand the similarities between the Mikado pheasant and the chicken at the genomic level, assembled scaffolds that were longer than 0.25% of the aligned

chicken chromosome were selected and plotted onto a syntenic map with an alignment length of at least 3 kb using MUMmer [20]. The identities of each chicken chromosome were between 86.24% and 89.98%, and the overall coverage was 85.28% (i.e., 855.35 Mb of the assembled scaffolds could be mapped onto the chicken genome; Additional file 1: Table S7). The syntenic relationships between the Mikado pheasant scaffolds and the chicken chromosomes were highly conserved, but a few of the chromosomes could be only partially aligned. In particular, 3 well-assembled scaffolds, i.e., scaffold14, scaffold69, and scaffold46, were mapped to nearly the full length of chicken chromosomes 15, 23, and 24, respectively. Notably, compared to the scaffolds of the Mikado pheasant, the chicken chromosomes, including chromosomes 6, 11, 18, and 21, were properly aligned, but with obvious inversions (Fig. 1A). More stringent conditions were then considered to evaluate the alignment of certain scaffolds to multiple chromosomes (e.g., scaffold1 and scaffold45; Fig. 1B); however, further confirmation is required to determine whether this finding represents the actual presence of intrachromosomal translocations in the Mikado pheasant genome. Additionally, the alignment between the Mikado pheasant scaffolds and the turkey chromosomes provided similar results (Additional file 1: Fig. S7A), but the Mikado pheasant scaffolds were poorly aligned with the zebra finch chromosomes (Additional file 1: Fig. S7B). In general, the Mikado scaffolds not only displayed a high level of synteny with the chicken and turkey scaffolds but also displayed a high frequency of potentially highly conserved regions, chromosomal rearrangements, and translocations.

Phylogenetic relationships of the Mikado pheasant

To compare the protein sequences of the Mikado pheasant against homologous protein families of other birds and organisms, OrthoMCL [21] was used to define the

orthologous gene families in 10 species. Proteins with sequences that were similar to those of the Mikado pheasant—5 birds (i.e., chicken, duck, flycatcher, turkey, and zebra finch), 2 reptiles (anole lizard and Chinese softshell turtle), and 2 mammals (human and mouse)—were classified into each gene family. First, 15 161 Mikado pheasant genes were identified in 18 220 families, and 5287 single-gene families that were common across the 10 species were then used to construct a Bayesian maximum clade credibility phylogenetic tree to estimate the time of divergence [22] (Fig. 2). The estimated time of divergence among the 3 *Phasianidae* was between 21.4 and 28.9 million years ago (Mya), and the Mikado pheasant was found to be more closely related to the turkey than to the chicken. The branches of the Galliformes and duck (76.4 Mya), Passeriformes and Galliformes (105.3 Mya), and anole lizard and Aves (266.3 Mya) displayed divergence times that were similar to those reported in the literature [23-25].

Gene family evolution

To assess the changes in the gene family sizes, a likelihood model was used to examine significant expansions and contractions of gene families, particularly in the Mikado pheasant lineage. Expansions or contractions in gene families indicate that total number of genes in a gene family are increasing or decreasing, respectively. The results revealed 311 expanded and 15 contracted gene families compared with the common ancestor of the Mikado pheasant and turkey (Fig. 2). In total, 86 gene ontology (GO) categories were significantly enriched ($p < 0.05$, empirical test) among the 311 expanded genes. Fifty of these GO categories were further classified into 8 main categories, including actin cytoskeleton, morphogenesis, catalytic activity, cell differentiation, binding, metabolism, cytoplasm, and organelle organization and biogenesis (Additional file 2: Table S8). In particular, the gene families involved in

oxygen and heme binding (GO:0019825 and GO:0020037, respectively), monooxygenase activity (GO:0004497), and energy metabolism (GO:0046034, ATP metabolic process; GO:0005977, glycogen metabolic process) were substantially expanded in the Mikado pheasant. Conversely, 7 of the 25 GO categories in the contracted gene families were significantly enriched in immune system processes and apoptosis (Additional file 1: Table S9). Moreover, 8 of the 75 expanded gene families were annotated as olfactory receptors (Additional file 2: Table S10) by the Pfam database [26].

Positive selection

To detect the genes that evolved rapidly under positive selection from the avian species, because of living at and between high and low elevation, 7132 single-copy orthologs were analyzed from 9038 genes common across the Mikado pheasant, chicken, turkey, duck, and zebra finch (Additional file 1: Fig. S8). According to the branch-site model and the likelihood ratio test, the positively selected genes (PSGs) identified in the Mikado pheasant were mainly enriched in functions such as metabolism (GO:0008152), cell (GO:0005623), and binding (GO:0005488) that belong to biological process, cellular component, and molecular function ontology terms, respectively (Additional file 1: Fig. S9). We further examined the PSGs involved in metabolism. The 45 PSGs enriched in metabolism-related functions (p-values < 0.05) were classified according to the GOSlim categories into lipid metabolism (GO:0006629), carbohydrate metabolic processes (GO:0005975), and generation of precursor metabolites and energy (GO:0006091), which included 13, 3, and 2 GO functions, respectively (Additional file 2: Table S11). Of these metabolism-related PSGs, 4 genes were found to be involved in the inositol phosphate metabolism (map00562; p-value < 0.01) and phosphatidylinositol signaling system

(map04070; *p*-value < 0.05) through a functional enrichment analysis from the Kyoto Encyclopedia of Genes and Genomes (KEGG) database (Additional file 1: Table S12).

In addition to metabolism, other high-altitude adaptations were observed, such as response to radiation (GO:0010212, response to ionizing radiation; GO:0010332, response to gamma radiation; GO:0034644, cellular response to UV; and GO:0071480, cellular response to gamma radiation), DNA repair (GO:0000731, DNA synthesis involved in DNA repair; GO:0045739, positive regulation of DNA repair; and GO:0006284, base-excision repair), and oxygen transport (GO:0016706, oxidoreductase activity; GO:0072593, reactive oxygen species metabolic process; GO:0019825, oxygen binding; and GO:2000377, regulation of reactive oxygen species metabolic process; Additional file 2: Table S13). Moreover, 43 PSGs in the Mikado pheasant were significantly enriched in the categories of lymphocyte activation (GO:0046649; including 8 GO terms) and cytokine production (GO:0001816; including 8 GO terms) (Additional file 2: Table S14). We also identified the janus kinase/signal transducer and activator of transcription (Jak-STAT) signaling pathway (map04630; *p*-value < 0.05), which was enriched in 5 PSGs (i.e., *BCL2*, *CCND3*, *IL12RB2*, *IL23R*, and *IL7*), in the KEGG analysis (Additional file 1: Table S15).

To further identify the PSGs that were exclusively present in the Mikado pheasant in comparison with the existing avian genomes, additional protein sequences were retrieved from 44 birds using the GigaScience database [17], in addition to the 5 birds (i.e., chicken, turkey, duck, zebra finch, and flycatcher) available in Ensembl. In total, 5287 single-copy orthologs from 50 avian species were identified, and branch model tests were performed to identify the PSGs for further GO enrichment analysis. We identified 149 genes that were positively selected in the Mikado pheasant in

comparison to the other 49 birds. Among these genes, 4 of the 39 enriched GO terms were related to ubiquitin activity (GO:0006511, ubiquitin-dependent protein catabolic process; GO:0019787, ubiquitin-like protein transferase activity; GO:0061631, ubiquitin conjugating enzyme activity; and GO:0061630, ubiquitin protein ligase activity; all p-values < 0.05). Additionally, the PSGs were found to be involved in the immune response, such as the positive regulation of defense response to virus by host (GO:0002230) and T cell differentiation in thymus (GO:0033077; all p-values < 0.05; Additional file 2: Table S16).

Identification of the MHC-B region of the Mikado pheasant

The major histocompatibility complex (MHC) is a cluster of genes that is associated with functions such as infectious disease resistance and immune responses in all jawed vertebrates [27]. The MHC B-locus (MHC-B) performs the main MHC functions in the chicken [28, 29]. Based on the above analysis, an assembled scaffold (scaffold208) was almost able to cover the known MHC-B contiguous region [30] on chicken chromosome 16. To understand the evolution of the MHC-B genes between the Mikado pheasant and the chicken, the predicted gene loci were manually curated by incorporating evidence from the aligned RNA-Seq data and homologous genes from chicken and turkey using Web Apollo software [31]. After the curation, 39 putative MHC genes of the Mikado pheasant were identified within a 227 kb sequence (Table 2), including 7 MHC class II loci (*BLB1*, *TAPBP*, *BLB2*, *BRD2*, *DMA*, *DMB1*, and *DMB2*), 4 MHC class I loci (*BF1*, *TAP1*, *TAP2*, and *BF2*), and 5 MHC class III loci (*C4*, *CenpA*, *CYP21*, *TNXB*, and *LTB4R1*).

Recently, gene loci involved in immunity have been shown to have a higher ratio of nonsynonymous (d_N) to synonymous (d_S) amino acid substitutions due to interactions with rapidly evolving pathogens under selective pressures [32]. *KIFC1*,

BTN1, *Blec2*, *BLB1*, *BLB2*, and *BF2* had comparatively high d_N/d_S ratios between the Mikado pheasant and the chicken (Table 2). Conversely, the genes with comparatively lower d_N/d_S ratios included *TRIM7.2*, *TRIM41*, *BRD2*, and *CenpA*. As shown in Fig. 3, the Mikado pheasant and the chicken displayed similarity in the MHC-B region and shared an almost perfect syntenic gene order. Based on the RNA-Seq results, 2.54 million reads were mapped onto 38 MHC-B genes (except for *BLB2*) of the Mikado pheasant, 27 of which had at least a 1-fold average coverage per nucleotide. Furthermore, 15 genes possessed more than 100-fold average coverage per nucleotide, providing concrete evidence of a reliable prediction. Notably, 2 gene loci, i.e., *TAPBP* and the *TAP1-TAP2* block, were inversely oriented compared to the chicken sequence.

Evolutionary history of *Syrmaticus* pheasants

The mitochondrial genome of the Mikado pheasant was assembled based on the short-read libraries. The circular complete genome had a total length of 16 680 bp, including 13 protein-coding genes, 2 rRNAs, 22 tRNAs, and a control region (Additional file 1: Table S17). The average nucleotide composition was 30.52% A, 31.20% C, 13.44% G, and 24.84% T. To investigate the evolutionary history of the genus *Syrmaticus*, which includes 5 long-tailed pheasants, the phylogeny was reconstructed, and the divergence times were estimated using the mitochondrial genomes. According to molecular clock analysis, the genetic divergence of the Mikado pheasant began approximately 3.47 (2.78-4.71) Mya (Fig. 4). The tree topology is consistent with previous studies [10, 33], and the divergence time suggests that the Mikado pheasant might have originated in the late Pliocene.

Amino acid substitution analysis in Mikado pheasant hemoglobin genes

Living at high elevations directly incurs the challenge of low oxygen availability. Additionally, exposure to low-pressure environments causes oxygen saturation in the arterial blood, thus decreasing and restricting oxygen supplementation to tissues [34]. Certain birds show an increased combined affinity between blood and oxygen via amino acid substitutions in the major hemoglobin [35-37]. To investigate their role in adaptation to high-altitude environments, amino acid substitutions were examined in the Mikado pheasant hemoglobin sequences. By comparing 6 avian species, an amino acid substitution with different consensus residues was found in the Mikado pheasant (Additional file 1: Fig. S10), and the substitution of alanine with threonine occurred at residue 78 of the alpha-A subunit—the major component of hemoglobin isoforms. The Andean goose, a kind of waterfowl living at over 3000 meters in the Andes, has been reported to carry the identical substitution [38].

Discussion

In this study, experimental data and statistical approaches were used to evaluate the genome assembly of the Mikado pheasant. Notably, the genome sequence of this species was previously unknown, and this study provides a comparative analysis of various genomes using a large number of tools at different stages for the assembly of the Mikado pheasant genome. While conducting the genome assembly, we used not only MaSuRCA but also assembly tools, such as ALLPATHS-LG [39], JR [40], Newbler [41], SGA [42], and SOAPdenovo [43]. All these assembly tools produced similar draft genome sizes, and MaSuRCA and SGA also showed similar results in terms of the N50 value and the scaffold number (Additional file 1: Table S18). To facilitate the downstream analysis, we used several methods to compare these

assembly sets. However, no single assembly tool outperformed the others in terms of the number of annotations for the predicted genes, the quality of the genome compared to that of other birds, and the BUSCO benchmark. In this study, the draft genome assembled using MaSuRCA was selected because it generated dramatically longer scaffolds that displayed a decent score on the BUSCO benchmark and produced proper annotations for the predicted genes. However, although a small number of misassembled or fragmented sequences was present in our proposed assembly (Fig. 1A, B), our approach still provides a practical strategy for whole-genome assembly using only short-read sequencing technology. We assert that the high coverage of our sequencing data, differing library insert sizes, and the use of a combination of tools, such as MaSuRCA and SSPACE for assembly and scaffolding, respectively, contributed to high-quality *de novo* assembly of the Mikado pheasant genome with a genome length of approximately 1 Gb.

Recent studies have reported phylogenetic tree topologies for the Mikado pheasant and other Galliformes birds [33, 44, 45]; however, these studies relied on small amounts of genomic DNA as supporting evidence. To obtain a highly accurate phylogenetic inference, long DNA sequences are necessary for the reconstruction of a high-resolution tree [46-48]. This study used whole-exome information, with 5287 single-copy orthologs totaling approximately 8 Mb of coding sequence, to reconstruct the phylogeny and estimate the divergence time among the Mikado pheasant and other birds (Fig. 2). Our results strongly suggest that the Mikado pheasant is more similar to the turkey than the chicken in the Galliformes clade, which is consistent with previous studies [33, 44, 45].

We additionally implemented a comprehensive phylogenetic analysis strategy to obtain information regarding the adaptive mechanisms of the Mikado pheasant to high elevations. Compared to birds living at low altitudes, both the positive gene selection

and gene expansion analyses showed a significant enrichment of genes relevant to energy metabolism (Additional file 2: Tables S8 and S11). This finding was consistent with the prior study that identified similar genes in other species inhabiting the highlands [49]. Moreover, the 4 metabolism-related PSGs (i.e., *INPP5A*, *INPP5J*, *PI4KB*, and *PLCE1*) that were involved in the inositol phosphate metabolism and phosphatidylinositol signaling system (Additional file 1: Table S12) were previously reported to be enriched in Tibetan pigs living at high altitudes [50]. Of these genes, *INPP5A* and *INPP5J* play a role in the hydrolysis of inositol polyphosphates [51], *PI4KB* is a phosphatidylinositol kinase that induces phosphorylation reactions [52], and *PLCE1*, which is a phospholipase enzyme, regulates gene expression, cell growth, and differentiation [53]. Another robust signal of its adaptation to high altitude was obtained from genes significantly associated with expansion of and positive selection for the enhancement of hemoglobin binding and oxygen transport (Additional file 2: Tables S8 and S13). Furthermore, for both the Mikado pheasant and Andean goose, an amino acid substitution was identified in the hemoglobin alpha-A subunit (Additional file 1: Fig. S10). The substitution of threonine at this position has recently been shown to cause an increase in the molecular volume, which might enhance the solubility of hemoglobin and facilitate adaptation to desiccating and high-altitude environments [38]. Through gene expansion, the genes of the Mikado pheasant that are involved in skeletal and cardiac muscle fiber development (Additional file 2: Table S8) and the enhanced functions of the additional GO terms implied that the biomass of the Mikado pheasant could be effectively produced in mountainous regions without nourishment, hence strongly suggesting the existence of an adaptive mechanism for high altitudes [54]. Finally, the PSGs in the radiation response, immune response, and DNA repair categories (Additional file 2: Tables S13 and S14) may reflect the increased resistance of the Mikado pheasant to long-term ultraviolet

radiation exposure through the induction of cytokine production [55] and lymphocyte activation [56] and DNA repair processes.

In this work, we annotated and curated the MHC-B gene loci in the Mikado pheasant, which is important for assessing the adaptive mechanisms associated with endangered species, because variations in gene number in the MHC cluster could be caused by exposure to pathogens or diseases [57, 58]. The genome of the Mikado pheasant contains a number of MHC-B genes, and inversions were observed in the *TAPBP* locus and the *TAP1-TAP2* block (Fig. 3) compared to the chicken genome; an inverse orientation of the *TAP1-TAP2* block was also detected compared to the turkey genome (Additional file 1: Fig. S11). A similar conversion at the MHC locus in Galliformes has been reported in previous studies [27, 32, 59]. We further observed a Blec2-like sequence with an inverse orientation located within the *BG1-Blec2* region in the Mikado pheasant. We inferred that this region is likely similar to the *Blec4* pseudogene of the chicken and highly similar to *Blec2* [30]. No *BLB2* genes were predicted between the *TAPBP* and *BRD2* intergenic regions in the Mikado pheasant MHC-B locus; however, these regions could be detected among the transcripts of our RNA-Seq data. A likely explanation for the absence of a prediction of the BLB2-like gene might be the existence of 2 unsequenced gap regions with a size of 1098 bp within the *TAPBP-BRD2* block (5931 bp). Since *BLB2* is only 792 bp in length, it could reside within the missing sequence.

In this study, we not only sequenced the whole genome of a genus of *Syrmaticus* but also completed the full mitochondrial genome. Before whole-genome sequences were available, mitochondrial sequences were widely utilized in molecular phylogenetic analyses of the genus of *Gallus* [60, 61]. Based on the assembly of the Mikado pheasant and the other 4 available sequences, we reconstructed a phylogenetic tree and provide a completely sequenced mitochondrial genome for 5

long-tailed pheasants. The topology of our reconstructed tree (Fig. 4) is consistent with results from a previous study [10]. However, the time of divergence was estimated to be earlier than the previously reported time [10] for the Mikado pheasant, which might have been due to the use of a few mitochondrial or nuclear genes rather than the complete mitochondrial genome. The reconstructed tree showed a potential migration pathway of these pheasants. The ancestors of the Mikado pheasant, which have been described to have migrated to the island of Taiwan, separated from the lineage of the copper pheasant (*S. soemmerringii ijimae*). The copper pheasant is a pheasant indigenous to Japan, whose ancestors might have separated from the lineage of the Reeves's pheasant (*S. reevesii*) that has inhabited in Northern China. The ancestors of Elliot's pheasant (*S. ellioti*) and Mrs. Hume's pheasant (*S. humiae*) have branched from the Mikado pheasant, then separated into two present kinds of pheasants that have alternatively roosted in the mountainous forests of Southeastern and Southwestern China, respectively. According to paleogeographical reports, Taiwan was formed approximately 4-5 Mya and attained its modern topography approximately 3 Mya [62]. The sea level was lower during the glacial periods, and Taiwan might have been connected to the mainland [63]. Our results suggest that the evolutionary history of the Mikado pheasant might have included ancestors that migrated from the north towards Taiwan approximately 3.47 Mya and consequently were isolated by the Taiwan Strait during the warm interglacial periods during the early Pleistocene.

Materials and Methods

De novo genome assembly

Blood samples were collected from a single female Mikado pheasant living in Central Taiwan; then, genomic DNA was extracted, and 2 paired-end libraries (280 bp and 480 bp; average read length: 151 bp) and 5 mate pair libraries (1, 3, 5, 7, and 10 kb; average read length: 101 bp) were constructed according to the manufacturer's protocol. In addition, 2 RNA-Seq libraries from 2 male Mikado pheasants were prepared for the draft genome assessment and gene prediction (Additional file 1: Table S1). The DNA libraries were sequenced using the HiSeq platform (Illumina Inc., San Diego, CA, USA), and the RNA libraries were sequenced using the HiScanSQ and HiSeq platforms.

The genome assembly performed in this study was divided into the following 3 stages: 1) quality control and preprocessing, 2) contig assembly, and 3) scaffolding. First, the quality of the raw reads was examined using FastQC. Read trimming and adapter removal were performed as needed. For the mate pair libraries, only 35 bases from the 5'end of both reads were used in the scaffolding step. Next, contig assembly was performed using MaSuRCA (version 2.3.2). In this step, both the paired-end and mate pair libraries were employed. Finally, SSPACE (version 3.0) [64] was used to concatenate the contigs into scaffolds based on the 5 mate pair libraries. Scaffold sequences shorter than 300 bp were then excluded from the final assembly. The statistical results of the assembly were estimated using QUAST (version 3.2) [65].

Evaluation of assembly quality

Several metrics provided by BUSCO (version 1.21) were used to evaluate the assembly quality, including the number and length distribution of the scaffold

sequences, the mapping rate of the paired-end DNA reads and RNA reads, the per-base coverage of the DNA read mapping, and the coverage of universal single-copy orthologs. To evaluate the mapping rate of the reads and per-base coverage, the paired-end DNA reads and RNA reads were aligned against the assembled scaffolds using Bowtie 2 [66] and TopHat2 [67], respectively. The scaffolds were mainly assembled from the paired-end DNA reads, and the higher mapping rate of the paired-end DNA reads suggests a higher degree of the final assembly covering the raw reads. The per-base DNA read coverage was calculated using BEDTools [68]. For each base, the expected coverage should be close to the sequencing depth of the paired-end reads (approximately 98.7x). Regarding the RNA reads, the mapping rate showed the completeness of the final assembly with respect to the independent sequencing data from the transcriptomes of the Mikado pheasant. The BUSCO benchmark is a single-copy ortholog set derived from the species of a major lineage. The gene models predicted from the draft genome in the Mikado pheasant were compared with the lineage of vertebrates (3023 orthologs in total) provided by BUSCO. Protein sequences from the chicken, duck, turkey, and zebra finch were also evaluated for comparison.

Genome comparison

To compare the genome of the Mikado pheasant with that of other avians, we retrieved the whole-genome sequences of the chicken (Galgal4), turkey (UMD2) and zebra finch (taeGut3.2.4) from the Ensembl database. Using the genome-wide sequence aligner MUMmer (version 3.23), the chromosome-level differences and similarities among the species were investigated and visualized. The structural variants among the species were further reported using the "show-diff" utility in MUMmer. The chord diagrams of the alignment were generated using Circos [69].

Gene prediction and annotation

First, RepeatMasker was applied to screen the scaffolds for interspersed repeats and low-complexity regions in the DNA sequences, and the masked genome was used for further gene prediction. Then, homology-based, RNA-Seq, and *ab initio* prediction approaches were used to identify protein-coding genes and build a consensus gene set that included all predicted genes. For the homology protein sequence alignment, the protein sequences of the chicken (Galgal4), turkey (UMD2), duck (BGI_duck_1.0) and zebra finch (taeGut3.2.4) were collected from Ensembl. The protein sequence alignments were performed using Exonerate (version 2.2.0) [70]. All RNA-Seq reads were aligned against the repeat-masked genome using TopHat2, which generated evidence of splice sites, introns, and exons. Additionally, Trinity (version 2.0.6) [71] was utilized to assemble transcripts, and PASA (version 2.0.0) [72] was used to group alternatively spliced isoforms. For the *ab initio* gene prediction, the standard Augustus (version 3.0.3) pipeline was used to yield potentially predicted genes with evidence from both homologous proteins and RNA-Seq. Next, the consensus gene set was determined by consolidating the 3 types of gene prediction using EVidenceModeler (version 1.1.1). Finally, the gene annotations were defined based on the best sequence alignment against NCBI NR proteins in Aves and Reptilians using BLASTP (version 2.2.29+), with the following criteria: identity $\geq$ 30%, alignment length $\geq$ 80 bp, and E-value $\leq$ 1e–5. For the protein domain identification, we annotated the domains using HMMER (version 3.1b2) [73] by scanning the Pfam database (version 30.0). In addition, we used MAKER [74] (version 2.31.8) to predict gene loci in the MHC region.

Gene families

To identify orthologs, the protein-coding genes of 5 birds (i.e., *Gallus gallus, Meleagris gallopavo, Anas platyrhynchos, Taeniopygia guttata,* and *Ficedula albicollis*) and 4 additional species (*Anolis carolinensis, Pelodiscus sinensis, Homo sapiens,* and *Mus musculus*) were downloaded from Ensembl (release 82). The sequence of the longest isoform was selected to represent the gene for each species, despite the presence of protein isoforms. The all-vs-all BLASTP was applied to align all protein sequences (including those of the Mikado pheasant) of the 10 species and 5 birds (excluding flycatcher) with E-value thresholds less than 1e–5 and 1e–20, respectively. To examine the orthologous genes in the species of interest, OrthoMCL (version 2.0.9) was used to classify the gene families, and 5287 single-gene families were obtained from the 10 species, and 7132 single-gene families were obtained from the 5 birds. Furthermore, MUSCLE (version 3.8.1551) [75] was used for the multiple sequence alignment of the converted coding DNA sequences, and Gblocks (version 0.91b) [76] was used to remove the poorly aligned regions. After trimming, the genes from each species were concatenated using the same order to reconstruct the phylogenies and evaluate the divergence time. The concatenated sequences were used to build a phylogenetic tree using RAxML (version 8.2.4) [77] via a maximum likelihood search; then, the divergence time was analyzed using BEAST (version 2.3.2) with the GTR+I+Γ model, which is the best substitution model selected by Modeltest (version 3.7) and PAUP* (version 4.0a150) [78]. Four nodes were chosen as the fossil calibration points from the TimeTree database [79], including human-chicken (311.9 Mya), anole lizard-chicken (279.7 Mya), Chinese softshell turtle-chicken (253.7 Mya), and human-mouse (89.8 Mya). The phylogenetic tree was generated using the Strap R package [80]. To identify the gene families with a expansion or contraction between the Mikado pheasant and other species, CAFE

(version 3.1) [81] was used to estimate the rates of gene family evolution from the observed gene numbers in each family and the given phylogenetic tree. A p-value < 0.05 was used to indicate significant changes in the gene family size.

Examination of genes under positive selection and enrichment analysis

To determine the genes that underwent positive natural selection in the Mikado pheasant, CODEML from PAML (version 4.8) [82] was applied to both the branch-site and branch models to investigate the genes in positively selected sites and the genes with increased d_N/d_S values within the considered lineage of the Mikado pheasant, respectively. For the branch-site model, we implemented likelihood ratio tests to determine the statistical significance of positive selection for testing a null model (model = 2, NSsites = 2, fix_omega = 1, and omega = 1) against an alternative model (model = 2, NSsites = 2, and fix_omega = 0). Consequently, the false discovery rates (FDRs) were computed with a cutoff of 0.05 to adjust for multiple testing using the Benjamini-Hochberg procedure. Similarly, for the branch model tests, the 2-ratio branch model (model = 2 and NSsites = 0) for the foreground (Mikado pheasant) and background (other birds) branches and the one-ratio model (model = 0 and NSsites = 0) as the null model for all branches were implemented and compared via likelihood ratio tests with respect to the FDR to evaluate the significance of genes associated with accelerated evolution.

The GO annotations of 4 birds (i.e., chicken, duck, turkey, and zebra finch) retrieved from the Ensembl BioMart were used to characterize the functions of the identified orthologs. A hypergeometric test was performed to identify significant GO functions in these orthologs. However, the raw p-values of the hypergeometric tests can easily be affected by the number of genes [83]; therefore, to address the underlying bias of the hypergeometric distribution, we further calculated empirical

p-values [84]. The empirical *p*-values were determined through 100K simulated datasets by ranking the hypergeometric probability of enriched functional categories compared with the null baseline probabilities. The null baseline probability was established by randomly selecting a group of genes containing an equal number of PSGs with an FDR < 0.05 for both the branch and branch-site models. For massively enriched GO terms with similar functions, CateGOrizer [85] was used to classify the genes into basic categories. ClueGO [86] with the hypergeometric test and a Bonferroni adjustment were performed to enrich the KEGG pathways [87].

Mitochondrial genome assembly

Geneious (version 8.1.5) [88] was utilized with the default settings to assemble the whole mitochondrial genome. First, the reads were mapped to the 4 available *Syrmaticus* mitochondrial genomes from GenBank (AB164622.1 - AB164625.1). The mapped reads were collected and then used for the further assembly of the mitochondrial genome of the Mikado pheasant. The genes were identified using MITOS [89] and curated by comparison with known sequences of other long-tailed pheasants from GenBank. The phylogenetic reconstruction and estimation of the divergence times among the 5 long-tailed pheasants were achieved using BEAST with the GTR+G model, which was selected as the best nucleotide substitution model by Modeltest and PAUP*. We added 2 nodes as the fossil calibration points according to the TimeTree database, including Elliot's pheasant-Reeves's pheasant (11.1 Mya) and Elliot's pheasant-Mrs. Hume's pheasant (0.2 Mya). A calibrated Yule speciation process was implemented in the analysis using BEAST. In the Markov chain Monte Carlo analysis, the chain length utilized 10 million generations.

Additional files

Additional file 1: Supplementary figures S1-S11 and supplementary tables S1-S7, S9, S12, S15, and S17-S18.

Additional file 2: Supplementary tables S8, S10-S11, S13-S14, and S16.

List of abbreviations

FDR: false discovery rate; GO: Gene Ontology; IUCN: International Union for Conservation of Nature; LRT: likelihood ratio test; MHC: major histocompatibility complex; Mya: million years ago; NR: non-redundant; PSG: positively selected gene.

Availability of supporting data

Data for the *Syrmaticus mikado* genome has been deposited in the GenBank/EMBL/DDBJ Bioproject database under the project number PRJNA389983. Raw genomic and transcriptomic sequence datasets were deposited in Sequence Read Archive (SRA) under the accession code SRP108966.

Competing interests

The authors declare that they have no competing interests.

Funding

This work was supported by grant from Taipei Zoo (No. 13, 2015 Animal Adoption Programs of Taipei Zoo). The funder had no role in the design, collection, analysis, or interpretation of the data; writing the manuscript; or the decision to submit the manuscript for publication.

Acknowledgements

We thank Dr. Mei-Yeh Lu and the High Throughput Genomics Core at the Biodiversity Research Center, Academia Sinica, for the NGS library constructions and Illumina sequencing. We are also grateful to the Computer and Information Networking Center, National Taiwan University, and the National Center for High-performance Computing for the support of the high-performance computing facilities. We thank Dr. Chih-Ming Hung and Dr. Chen Siang Ng for discussion and comments on the phylogenetic analysis, Dr. Chia-Yang Cheng for assistance in data analyses, Dr. Melissa Stauffer and Dr. Yao-Yin Chang for assistance with editing the manuscript, and Yu-Cheng Hsieh for providing photos of the Mikado pheasant.

Author Contributions

E.Y.C., C.-Y.C., M.-H.T., S.-T.D., and H.L. conceived the project. E.Y.C., C.-Y.C., M.-H.T., and E.-C.L. managed and coordinated the project. M.-H.T., S.-T.D., and E.-C.L. performed animal work and prepared biological samples. T.-P.L. and L.-C.L. designed bioinformatics and evolutionary analyses. C.-Y.L., P.-H.H., and K.-Y.L. performed genome assembly. P.-H.H. performed assessment of the assembly quality. C.-Y.L. and P.-H.H. performed gene prediction and annotation. C.-Y.L. and L.-M.C. performed evolutionary analysis. C.-Y.L. performed mitochondrial genome assembly and gene annotation, and curated the MHC-B gene loci. Y.-F.L. wrote a visualization program for displaying MHC-B genes. C.-Y.L., P.-H.H., and A.C. wrote the manuscript. A.C., M.-H.T., and C.-Y.C. commented on the draft and revised the manuscript. All authors read and approved the final manuscript.

References

1. Bridgman CL. Habitat use, distribution and conservation status of the mikado pheasant (Syrmaticus mikado) in Taiwan. The University of Tennessee; 2002.
2. Severinghaus SR. A study of the Swinhoe's and Mikado pheasants in Taiwan with recommendations for their conservation. Cornell University, May; 1977.
3. McGowan PJ and Garson PJ. Pheasants: status survey and conservation action plan 1995-1999. IUCN; 1995.
4. Fuller RA. Pheasants: status survey and conservation action plan 2000-2004. IUCN; 2000.
5. Li R, Fan W, Tian G, Zhu H, He L, Cai J, et al. The sequence and de novo assembly of the giant panda genome. Nature. 2010;463 7279:311-7. doi:10.1038/nature08696.
6. Hung CM, Shaner PJ, Zink RM, Liu WC, Chu TC, Huang WS, et al. Drastic population fluctuations explain the rapid extinction of the passenger pigeon. Proceedings of the National Academy of Sciences of the United States of America. 2014;111 29:10636-41. doi:10.1073/pnas.1401526111.
7. Li S, Li B, Cheng C, Xiong Z, Liu Q, Lai J, et al. Genomic signatures of near-extinction and rebirth of the crested ibis and other endangered bird species. Genome biology. 2014;15 12:557. doi:10.1186/s13059-014-0557-1.
8. Le Duc D, Renaud G, Krishnan A, Almen MS, Huynen L, Prohaska SJ, et al. Kiwi genome provides insights into evolution of a nocturnal lifestyle. Genome biology. 2015;16:147. doi:10.1186/s13059-015-0711-4.
9. Yu L, Wang GD, Ruan J, Chen YB, Yang CP, Cao X, et al. Genomic analysis of snub-nosed monkeys (Rhinopithecus) identifies genes and processes related to high-altitude adaptation. Nat Genet. 2016;48 8:947-52. doi:10.1038/ng.3615.
10. Zhan XJ and Zhang ZW. Molecular phylogeny of avian genus Syrmaticus based on the mitochondrial cytochrome B gene and control region. Zoolog Sci. 2005;22 4:427-35. doi:10.2108/zsj.22.427.
11. Lee P, Lue K, Hsieh J, Lee Y, Pan Y, Chen H, et al. A wildlife distribution database in Taiwan. Council of Agriculture, Taipei. 1998.
12. Chikhi R and Medvedev P. Informed and automated k-mer size selection for genome assembly. Bioinformatics. 2014;30 1:31-7. doi:10.1093/bioinformatics/btt310.
13. Zimin AV, Marcais G, Puiu D, Roberts M, Salzberg SL and Yorke JA. The MaSuRCA genome assembler. Bioinformatics. 2013;29 21:2669-77. doi:10.1093/bioinformatics/btt476.

14. Smit AF, Hubley R and Green P. RepeatMasker Open-3.0. 1996.
15. Stanke M, Diekhans M, Baertsch R and Haussler D. Using native and syntenically mapped cDNA alignments to improve de novo gene finding. Bioinformatics. 2008;24 5:637-44.
16. Haas BJ, Salzberg SL, Zhu W, Pertea M, Allen JE, Orvis J, et al. Automated eukaryotic gene structure annotation using EVidenceModeler and the Program to Assemble Spliced Alignments. Genome biology. 2008;9 1:R7. doi:10.1186/gb-2008-9-1-r7.
17. Zhang G, Li B, Li C, Gilbert MT, Jarvis ED, Wang J, et al. Comparative genomic data of the Avian Phylogenomics Project. Gigascience. 2014;3 1:26. doi:10.1186/2047-217X-3-26.
18. Lee C-Y, Chiu Y-C, Wang L-B, Kuo Y-L, Chuang EY, Lai L-C, et al. Common applications of next-generation sequencing technologies in genomic research. Translational cancer research. 2013;2 1:33-45.
19. Simao FA, Waterhouse RM, Ioannidis P, Kriventseva EV and Zdobnov EM. BUSCO: assessing genome assembly and annotation completeness with single-copy orthologs. Bioinformatics. 2015;31 19:3210-2. doi:10.1093/bioinformatics/btv351.
20. Kurtz S, Phillippy A, Delcher AL, Smoot M, Shumway M, Antonescu C, et al. Versatile and open software for comparing large genomes. Genome biology. 2004;5 2:R12. doi:10.1186/gb-2004-5-2-r12.
21. Li L, Stoeckert CJ, Jr. and Roos DS. OrthoMCL: identification of ortholog groups for eukaryotic genomes. Genome research. 2003;13 9:2178-89. doi:10.1101/gr.1224503.
22. Drummond AJ, Suchard MA, Xie D and Rambaut A. Bayesian phylogenetics with BEAUti and the BEAST 1.7. Mol Biol Evol. 2012;29 8:1969-73. doi:10.1093/molbev/mss075.
23. Lu L, Chen Y, Wang Z, Li X, Chen W, Tao Z, et al. The goose genome sequence leads to insights into the evolution of waterfowl and susceptibility to fatty liver. Genome biology. 2015;16:89. doi:10.1186/s13059-015-0652-y.
24. Jiang L, Wang G, Peng R, Peng Q and Zou F. Phylogenetic and molecular dating analysis of Taiwan Blue Pheasant (Lophura swinhoii). Gene. 2014;539 1:21-9. doi:10.1016/j.gene.2014.01.067.
25. Cai Q, Qian X, Lang Y, Luo Y, Xu J, Pan S, et al. Genome sequence of ground tit Pseudopodoces humilis and its adaptation to high altitude. Genome biology. 2013;14 3:R29. doi:10.1186/gb-2013-14-3-r29.

26. Finn RD, Mistry J, Tate J, Coggill P, Heger A, Pollington JE, et al. The Pfam protein families database. Nucleic Acids Res. 2010;38 Database issue:D211-22. doi:10.1093/nar/gkp985.

27. Wang B, Ekblom R, Strand TM, Portela-Bens S and Hoglund J. Sequencing of the core MHC region of black grouse (Tetrao tetrix) and comparative genomics of the galliform MHC. BMC Genomics. 2012;13:553. doi:10.1186/1471-2164-13-553.

28. Kaufman J, Milne S, Gobel TW, Walker BA, Jacob JP, Auffray C, et al. The chicken B locus is a minimal essential major histocompatibility complex. Nature. 1999;401 6756:923-5. doi:10.1038/44856.

29. Kaufman J, Volk H and Wallny HJ. A "minimal essential Mhc" and an "unrecognized Mhc": two extremes in selection for polymorphism. Immunol Rev. 1995;143:63-88.

30. Shiina T, Briles WE, Goto RM, Hosomichi K, Yanagiya K, Shimizu S, et al. Extended gene map reveals tripartite motif, C-type lectin, and Ig superfamily type genes within a subregion of the chicken MHC-B affecting infectious disease. J Immunol. 2007;178 11:7162-72.

31. Lee E, Helt GA, Reese JT, Munoz-Torres MC, Childers CP, Buels RM, et al. Web Apollo: a web-based genomic annotation editing platform. Genome biology. 2013;14 8:R93. doi:10.1186/gb-2013-14-8-r93.

32. Chaves LD, Krueth SB and Reed KM. Defining the turkey MHC: sequence and genes of the B locus. J Immunol. 2009;183 10:6530-7. doi:10.4049/jimmunol.0901310.

33. Wang N, Kimball RT, Braun EL, Liang B and Zhang Z. Assessing phylogenetic relationships among galliformes: a multigene phylogeny with expanded taxon sampling in Phasianidae. PLoS One. 2013;8 5:e64312. doi:10.1371/journal.pone.0064312.

34. Powell FL, Shams H, Hempleman SC and Mitchell GS. Breathing in thin air: acclimatization to altitude in ducks. Respir Physiol Neurobiol. 2004;144 2-3:225-35. doi:10.1016/j.resp.2004.07.021.

35. Monge C and Leon-Velarde F. Physiological adaptation to high altitude: oxygen transport in mammals and birds. Physiol Rev. 1991;71 4:1135-72.

36. Weber RE, Jessen TH, Malte H and Tame J. Mutant hemoglobins (alpha 119-Ala and beta 55-Ser): functions related to high-altitude respiration in geese. J Appl Physiol (1985). 1993;75 6:2646-55.

37. Jessen TH, Weber RE, Fermi G, Tame J and Braunitzer G. Adaptation of bird hemoglobins to high altitudes: demonstration of molecular mechanism by

protein engineering. Proceedings of the National Academy of Sciences of the United States of America. 1991;88 15:6519-22.

38. McCracken KG, Barger CP and Sorenson MD. Phylogenetic and structural analysis of the HbA (alphaA/betaA) and HbD (alphaD/betaA) hemoglobin genes in two high-altitude waterfowl from the Himalayas and the Andes: Bar-headed goose (Anser indicus) and Andean goose (Chloephaga melanoptera). Mol Phylogenet Evol. 2010;56 2:649-58. doi:10.1016/j.ympev.2010.04.034.

39. Gnerre S, Maccallum I, Przybylski D, Ribeiro FJ, Burton JN, Walker BJ, et al. High-quality draft assemblies of mammalian genomes from massively parallel sequence data. Proceedings of the National Academy of Sciences of the United States of America. 2011;108 4:1513-8. doi:10.1073/pnas.1017351108.

40. Chu TC, Lu CH, Liu T, Lee GC, Li WH and Shih AC. Assembler for de novo assembly of large genomes. Proceedings of the National Academy of Sciences of the United States of America. 2013;110 36:E3417-24. doi:10.1073/pnas.1314090110.

41. Margulies M, Egholm M, Altman WE, Attiya S, Bader JS, Bemben LA, et al. Genome sequencing in microfabricated high-density picolitre reactors. Nature. 2005;437 7057:376-80. doi:10.1038/nature03959.

42. Simpson JT and Durbin R. Efficient de novo assembly of large genomes using compressed data structures. Genome research. 2012;22 3:549-56. doi:10.1101/gr.126953.111.

43. Luo R, Liu B, Xie Y, Li Z, Huang W, Yuan J, et al. SOAPdenovo2: an empirically improved memory-efficient short-read de novo assembler. Gigascience. 2012;1 1:18. doi:10.1186/2047-217X-1-18.

44. Eo SH, Bininda-Emonds OR and Carroll JP. A phylogenetic supertree of the fowls (Galloanserae, Aves). Zoologica Scripta. 2009;38 5:465-81.

45. Kimball RT, Mary CM and Braun EL. A macroevolutionary perspective on multiple sexual traits in the phasianidae (galliformes). Int J Evol Biol. 2011;2011:423938. doi:10.4061/2011/423938.

46. Dunn CW, Hejnol A, Matus DQ, Pang K, Browne WE, Smith SA, et al. Broad phylogenomic sampling improves resolution of the animal tree of life. Nature. 2008;452 7188:745-9. doi:10.1038/nature06614.

47. Naylor GJ and Brown WM. Structural biology and phylogenetic estimation. Nature. 1997;388 6642:527-8. doi:10.1038/41460.

48. Rosenberg MS and Kumar S. Incomplete taxon sampling is not a problem for phylogenetic inference. Proceedings of the National Academy of Sciences of

the United States of America. 2001;98 19:10751-6. doi:10.1073/pnas.191248498.

49. Li M, Tian S, Jin L, Zhou G, Li Y, Zhang Y, et al. Genomic analyses identify distinct patterns of selection in domesticated pigs and Tibetan wild boars. Nat Genet. 2013;45 12:1431-8. doi:10.1038/ng.2811.

50. Zhang B, Qiangba Y, Shang P, Wang Z, Ma J, Wang L, et al. A Comprehensive MicroRNA Expression Profile Related to Hypoxia Adaptation in the Tibetan Pig. PLoS One. 2015;10 11:e0143260. doi:10.1371/journal.pone.0143260.

51. Hsu F and Mao Y. The structure of phosphoinositide phosphatases: Insights into substrate specificity and catalysis. Biochim Biophys Acta. 2015;1851 6:698-710. doi:10.1016/j.bbalip.2014.09.015.

52. Rapoport SI, Primiani CT, Chen CT, Ahn K and Ryan VH. Coordinated Expression of Phosphoinositide Metabolic Genes during Development and Aging of Human Dorsolateral Prefrontal Cortex. PLoS One. 2015;10 7:e0132675. doi:10.1371/journal.pone.0132675.

53. Tan J, Yu CY, Wang ZH, Chen HY, Guan J, Chen YX, et al. Genetic variants in the inositol phosphate metabolism pathway and risk of different types of cancer. Sci Rep. 2015;5:8473. doi:10.1038/srep08473.

54. Zhu L, Li M, Li X, Shuai S, Liu H, Wang J, et al. Distinct expression patterns of genes associated with muscle growth and adipose deposition in tibetan pigs: a possible adaptive mechanism for high altitude conditions. High Alt Med Biol. 2009;10 1:45-55. doi:10.1089/ham.2008.1042.

55. Ullrich SE and Schmitt DA. The role of cytokines in UV-induced systemic immune suppression. Journal of dermatological science. 2000;23 Suppl 1:S10-2.

56. Baadsgaard O, Fox DA and Cooper KD. Human epidermal cells from ultraviolet light-exposed skin preferentially activate autoreactive CD4+2H4+ suppressor-inducer lymphocytes and CD8+ suppressor/cytotoxic lymphocytes. J Immunol. 1988;140 6:1738-44.

57. Bollmer JL, Vargas FH and Parker PG. Low MHC variation in the endangered Galapagos penguin (Spheniscus mendiculus). Immunogenetics. 2007;59 7:593-602. doi:10.1007/s00251-007-0221-y.

58. Wan QH, Zhu L, Wu H and Fang SG. Major histocompatibility complex class II variation in the giant panda (Ailuropoda melanoleuca). Mol Ecol. 2006;15 9:2441-50. doi:10.1111/j.1365-294X.2006.02966.x.

59. Zeng QQ, Zhong GH, He K, Sun DD and Wan QH. Molecular characterization of classical and nonclassical MHC class I genes from the

golden pheasant (Chrysolophus pictus). Int J Immunogenet. 2016;43 1:8-17. doi:10.1111/iji.12245.

60. Kan XZ, Yang JK, Li XF, Chen L, Lei ZP, Wang M, et al. Phylogeny of major lineages of galliform birds (Aves: Galliformes) based on complete mitochondrial genomes. Genet Mol Res. 2010;9 3:1625-33. doi:10.4238/vol9-3gmr898.

61. Nishibori M, Shimogiri T, Hayashi T and Yasue H. Molecular evidence for hybridization of species in the genus Gallus except for Gallus varius. Anim Genet. 2005;36 5:367-75. doi:10.1111/j.1365-2052.2005.01318.x.

62. Liu T-K, Chen Y-G, Chen W-S and Jiang S-H. Rates of cooling and denudation of the Early Penglai Orogeny, Taiwan, as assessed by fission-track constraints. Tectonophysics. 2000;320 1:69-82.

63. Osozawa S, Shinjo R, Armid A, Watanabe Y, Horiguchi T and Wakabayashi J. Palaeogeographic reconstruction of the 1.55 Ma synchronous isolation of the Ryukyu Islands, Japan, and Taiwan and inflow of the Kuroshio warm current. International Geology Review. 2012;54 12:1369-88.

64. Boetzer M, Henkel CV, Jansen HJ, Butler D and Pirovano W. Scaffolding pre-assembled contigs using SSPACE. Bioinformatics. 2011;27 4:578-9. doi:10.1093/bioinformatics/btq683.

65. Gurevich A, Saveliev V, Vyahhi N and Tesler G. QUAST: quality assessment tool for genome assemblies. Bioinformatics. 2013;29 8:1072-5. doi:10.1093/bioinformatics/btt086.

66. Langmead B and Salzberg SL. Fast gapped-read alignment with Bowtie 2. Nat Methods. 2012;9 4:357-9. doi:10.1038/nmeth.1923.

67. Kim D, Pertea G, Trapnell C, Pimentel H, Kelley R and Salzberg SL. TopHat2: accurate alignment of transcriptomes in the presence of insertions, deletions and gene fusions. Genome biology. 2013;14 4:R36. doi:10.1186/gb-2013-14-4-r36.

68. Quinlan AR and Hall IM. BEDTools: a flexible suite of utilities for comparing genomic features. Bioinformatics. 2010;26 6:841-2. doi:10.1093/bioinformatics/btq033.

69. Krzywinski M, Schein J, Birol I, Connors J, Gascoyne R, Horsman D, et al. Circos: an information aesthetic for comparative genomics. Genome Res. 2009;19 9:1639-45. doi:10.1101/gr.092759.109.

70. Slater GS and Birney E. Automated generation of heuristics for biological sequence comparison. BMC Bioinformatics. 2005;6:31. doi:10.1186/1471-2105-6-31.

71. Grabherr MG, Haas BJ, Yassour M, Levin JZ, Thompson DA, Amit I, et al. Full-length transcriptome assembly from RNA-Seq data without a reference genome. Nature biotechnology. 2011;29 7:644-52. doi:10.1038/nbt.1883.

72. Haas BJ, Delcher AL, Mount SM, Wortman JR, Smith RK, Jr., Hannick LI, et al. Improving the Arabidopsis genome annotation using maximal transcript alignment assemblies. Nucleic Acids Res. 2003;31 19:5654-66.

73. Eddy SR. Profile hidden Markov models. Bioinformatics. 1998;14 9:755-63.

74. Holt C and Yandell M. MAKER2: an annotation pipeline and genome-database management tool for second-generation genome projects. BMC Bioinformatics. 2011;12:491. doi:10.1186/1471-2105-12-491.

75. Edgar RC. MUSCLE: multiple sequence alignment with high accuracy and high throughput. Nucleic Acids Res. 2004;32 5:1792-7. doi:10.1093/nar/gkh340.

76. Castresana J. Selection of conserved blocks from multiple alignments for their use in phylogenetic analysis. Mol Biol Evol. 2000;17 4:540-52.

77. Stamatakis A. RAxML version 8: a tool for phylogenetic analysis and post-analysis of large phylogenies. Bioinformatics. 2014;30 9:1312-3. doi:10.1093/bioinformatics/btu033.

78. Posada D. Using MODELTEST and PAUP* to select a model of nucleotide substitution. Current protocols in bioinformatics / editoral board, Andreas D Baxevanis [et al]. 2003;Chapter 6:Unit 6 5. doi:10.1002/0471250953.bi0605s00.

79. Hedges SB, Dudley J and Kumar S. TimeTree: a public knowledge-base of divergence times among organisms. Bioinformatics. 2006;22 23:2971-2. doi:10.1093/bioinformatics/btl505.

80. Bell MA and Lloyd GT. Strap: an R package for plotting phylogenies against stratigraphy and assessing their stratigraphic congruence. Palaeontology. 2015;58 2:379-89.

81. Han MV, Thomas GW, Lugo-Martinez J and Hahn MW. Estimating gene gain and loss rates in the presence of error in genome assembly and annotation using CAFE 3. Mol Biol Evol. 2013;30 8:1987-97. doi:10.1093/molbev/mst100.

82. Yang Z. PAML 4: phylogenetic analysis by maximum likelihood. Mol Biol Evol. 2007;24 8:1586-91. doi:10.1093/molbev/msm088.

83. Bleazard T, Lamb JA and Griffiths-Jones S. Bias in microRNA functional enrichment analysis. Bioinformatics. 2015;31 10:1592-8. doi:10.1093/bioinformatics/btv023.

84. Lu TP, Lee CY, Tsai MH, Chiu YC, Hsiao CK, Lai LC, et al. miRSystem: an integrated system for characterizing enriched functions and pathways of microRNA targets. PLoS One. 2012;7 8:e42390. doi:10.1371/journal.pone.0042390.

85. Bao J and Reecy JM. CateGOrizer: a web-based program to batch analyze gene ontology classification categories. Online Journal of Bioinformatics. 2008;9 2:108-12.

86. Bindea G, Mlecnik B, Hackl H, Charoentong P, Tosolini M, Kirilovsky A, et al. ClueGO: a Cytoscape plug-in to decipher functionally grouped gene ontology and pathway annotation networks. Bioinformatics. 2009;25 8:1091-3. doi:10.1093/bioinformatics/btp101.

87. Kanehisa M and Goto S. KEGG: kyoto encyclopedia of genes and genomes. Nucleic Acids Res. 2000;28 1:27-30.

88. Kearse M, Moir R, Wilson A, Stones-Havas S, Cheung M, Sturrock S, et al. Geneious Basic: an integrated and extendable desktop software platform for the organization and analysis of sequence data. Bioinformatics. 2012;28 12:1647-9. doi:10.1093/bioinformatics/bts199.

89. Bernt M, Donath A, Juhling F, Externbrink F, Florentz C, Fritzsch G, et al. MITOS: improved de novo metazoan mitochondrial genome annotation. Mol Phylogenet Evol. 2013;69 2:313-9. doi:10.1016/j.ympev.2012.08.023.

Figure Legends

Figure 1: A chromosome-level comparison of the Mikado pheasant and the chicken. **(A)** A syntenic map of the Mikado pheasant and chicken genomes. The x-axis specifies the chromosome position in the chicken, whereas the y-axis specifies the scaffold position in the Mikado pheasant. The red dots (or lines) indicate that the sequences were aligned in the same orientation, and the blue dots indicate an alignment with a reverse complement. **(B)** A chord diagram of scaffolds with a total length greater than 500 kb and an alignment length greater than 10 kb. The orange perimeters specify the chromosomes of the chicken, whereas the purple perimeters specify the scaffolds of the Mikado pheasant. The red links represent the sequences aligned in the same orientation, and the blue links represent an alignment with a reverse complement.

Figure 2: Evolution of gene families among various animal species. A phylogenetic tree was reconstructed based on 5287 single-copy orthologs of 10 species. The most recent common ancestor (MRCA) contains 18 220 gene families that were used to examine gene families with expansions or contractions. The numbers of gene families with significant expansions and contractions are shown in red and blue, respectively, at each branch. The divergence times and associated 95% confidence intervals (in parentheses) are indicated at the nodes of the tree in Mya. All nodes had 100% support in 500 bootstrap replicates.

Figure 3: An identity plot of the MHC regions of the Mikado pheasant and the chicken. The chicken MHC sequence was downloaded from GenBank (AB268588). Its nucleotide sequence from 17 978 to 241 251 was aligned against the Mikado pheasant MHC sequence from 2615 to 229 500 in scaffold208. The boxes on the horizontal and vertical axes, respectively, represent the gene loci in the Mikado

pheasant and the chicken. Boxes with different sizes exhibit different gene locus sizes, and red/blue coloring indicate genes in forward/reverse orientation. The red dots (or lines) on the diagonal indicate that the sequences were aligned in the same orientation, whereas the blue dots indicate alignments with reverse complements. The green dotted lines highlight the sequence of the inverted *TAPBP* locus and *TAP1-TAP2* block. The orange peaks show the read counts of the gene expression based on our RNA-Seq data. Those with more than 10 000 read counts were scaled to 10 000.

Figure 4: A phylogenetic tree of *Syrmaticus* pheasants. The divergence times and associated 95% confidence intervals shown in parentheses are given at the branch nodes of the tree in Mya.

Tables

Table 1: DNA contigs and scaffolds from the genomic data of the Mikado pheasant.

	**Contigs**	**Scaffolds**
Total length	1 054 607 905	1 035 950 077
Maximum length	195 342	50 275 205
Number of Ns	0	19 577 507
Average length	5050	110 690
N50[*]	13 461	11 324 524
N75[*]	6528	5 708 287
L50[†]	22 195	28
L75[†]	50 081	59
Counts > 300 bp	208 810	-
Counts > 1 kb	123 006	9359
Counts > 5 kb	61 237	1489
Counts > 10 kb	32 868	928

[*] The N50/N75 length is defined as the shortest sequence length at 50%/75% of the genome.

[†] The L50/L75 count is defined as the smallest number of contigs (or scaffolds) that those length sum produces N50/N75.

Table 2: Coding sequences of MHC-B genes in the Mikado pheasant and comparisons with the chicken.

	Mikado pheasant					Chicken					
**Gene**	**Position**	**Strand**	**Gene length**	**Amino acid length**	**Exon**	**Aligned base**	**Nucleotide identity (%)**	**Aligned amino acid**	**Amino acid identity (%)**	**Amino acid substitutions**	**d_N/d_S***
KIFC1	2615-5304	+	1140	380	7	1131	91.76	377	90.53	33	0.8669
Blec3	8997-11221	-	552	183	5	507	82.43	168	78.14	25	0.3821
Bzfp3	12126-18213	+	1449	482	13	1569	85.8	522	83.62	40	0.1884
TRIM7.2	19507-24562	-	1518	505	7	1518	95.98	505	98.61	7	0.0391
Bzfp2†	27027-29946	+	1368	455	4	1396	70.41	N/A	N/A	N/A	0.2438
Bzfp1	31049-33298	-	1425	474	2	1426	88.23	471	86.79	54	0.1900
44G24.1	37266-37673	-	408	136	1	408	85.78	136	80.15	27	0.2762
IL4I1	42730-46759	+	1578	525	6	1572	92.25	523	93.75	25	0.1011
TRIM7.1	51325-62131	-	1758	585	8	1767	92.49	588	92.69	40	0.1545
HEP21	63362-64247	-	324	107	3	324	93.52	107	91.59	9	0.2148
TRIM39.2	70980-74640	-	1392	464	6	1389	93.68	463	94.61	24	0.1167
TRIM27.2	76988-80522	+	1431	476	7	1431	94.13	476	92.23	37	0.2415
TRIM39.1	81560-85449	-	798	266	5	798	93.23	266	91.35	23	0.2753
TRIM27.1	86518-90228	-	1485	495	7	1485	94.48	495	94.34	28	0.1715
TRIM41	91918-96605	+	1656	551	7	1770	89.58	589	91.71	7	0.0375
GNB2L1	98038-101512	-	954	317	8	954	96.86	317	100	0	N/A
BTN1	103411-114264	+	930	309	8	939	74.64	339	57.26	96	0.8357
BTN2	117466-120157	+	1461	487	7	1481	90.41	469	83.37	52	0.3996
BG1	124105-125436	-	549	183	3	546	91.99	182	87.98	21	0.5591
Blec2	131358-133021	-	579	192	5	579	86.32	190	71.88	52	0.9375
Blec1	135818-137846	+	567	188	5	567	92.59	188	88.3	22	0.4683
BLB1	138411-139729	-	339	112	3	345	83.38	113	42.98	62	0.7904
TAPBP	140657-144216	+	1293	430	8	1293	92.19	430	89.77	44	0.3179
BLB2‡	N/A	N/A	792	263	N/A	792	92.93	263	85.93	37	1.4489
BRD2	150146-156295	-	2976	991	13	3078	86.85	776	75.28	30	0.0306
DMA	160545-162778	+	789	263	4	789	92.65	263	89.73	27	0.4528
DMB1	163010-165184	+	930	310	6	930	91.29	310	86.45	42	0.4978
DMB2	165617-168363	+	768	256	5	768	92.71	256	92.58	19	0.1622
BF1	169254-170740	+	996	331	5	1001	83.66	345	64.12	95	0.7116
TAP2	172793-176021	-	2100	700	9	2100	92.48	700	93.14	48	0.1675
TAP1	176574-180981	+	1752	584	11	1739	93.21	580	92.81	38	0.2191
BF2	181900-184038	-	1530	509	6	1213	62.28	326	57.39	119	0.8157
C4	185102-199258	+	5031	1676	40	4998	93.33	1665	93.2	101	0.1974
CenpA	199593-200795	+	396	131	4	396	96.72	131	99.24	1	0.0324
CYP21	201291-205141	+	1431	477	11	1431	92.67	477	94.13	28	0.7109
TNXB	209524-215604	-	2472	824	10	2496	92.14	832	92.34	50	0.2002
LTB4R1	221450-222538	+	1089	363	1	1089	94.12	363	94.49	20	0.1954
CD1A2	223740-225788	-	1044	348	6	1044	92.24	348	87.93	42	0.3796
CD1A1	227030-229500	-	1122	374	6	1122	93.4	374	90.64	35	0.3294

KIFC1, kinesin family member C1; Blec, C-type lectin-like receptor; Bzfp, B-locus zinc finger-like protein; TRIM, tripartite motif containing protein; 44G24.1, histone H2B-like protein; IL4I1, interleukin 4 induced 1; HEP21, hen egg protein 21 kDa; GNB2L1, guanine nucleotide binding-like protein; BTN, B-butyrophilin protein; BG1, BG-like antigen; CD1A1/A2, CD1-like proteins

[*] d_N/d_S = ratio of nonsynonymous (d_N) to synonymous (d_S) substitutions.

[†] Defined as a pseudogene in chicken.

[‡] No predicted result was identified from the DNA assembly. The transcript sequence was alternatively derived from the transcriptome assembly by RNA-Seq.

Figure 1

Click here to download Figure Fig1.tif

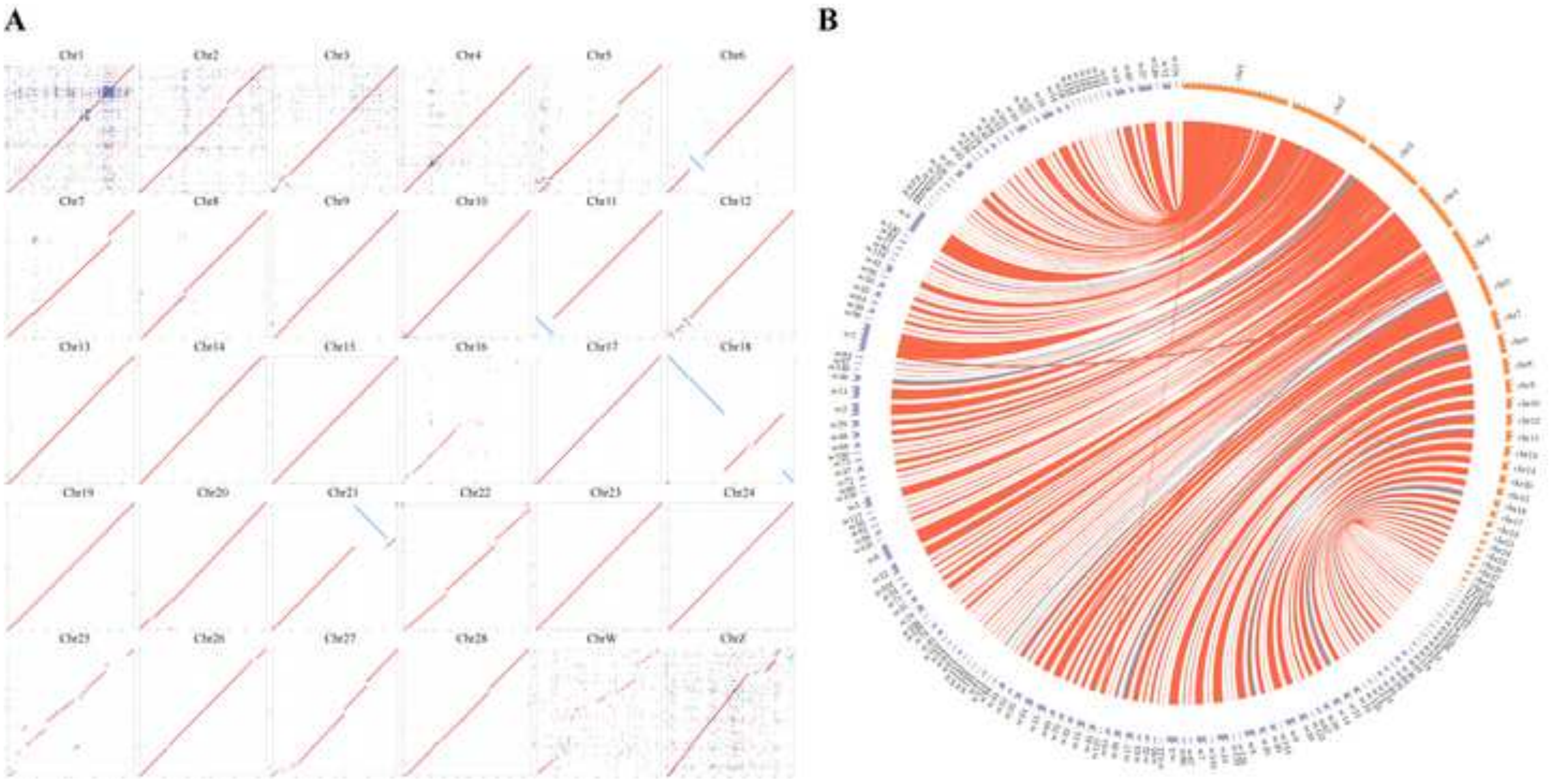

Figure 2

Click here to download Figure Fig2.tif

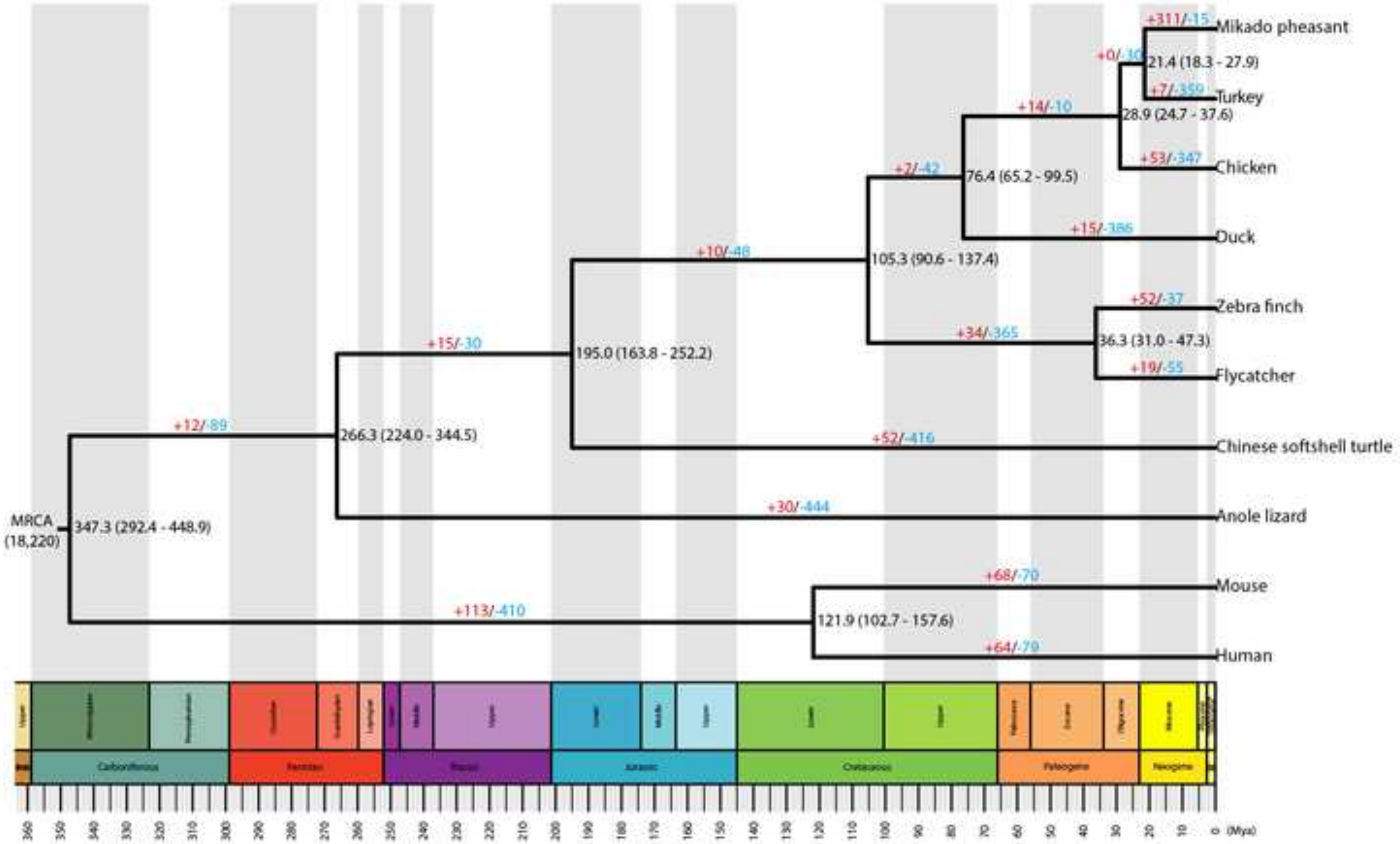

Figure 3

Click here to download Figure Fig3.tif

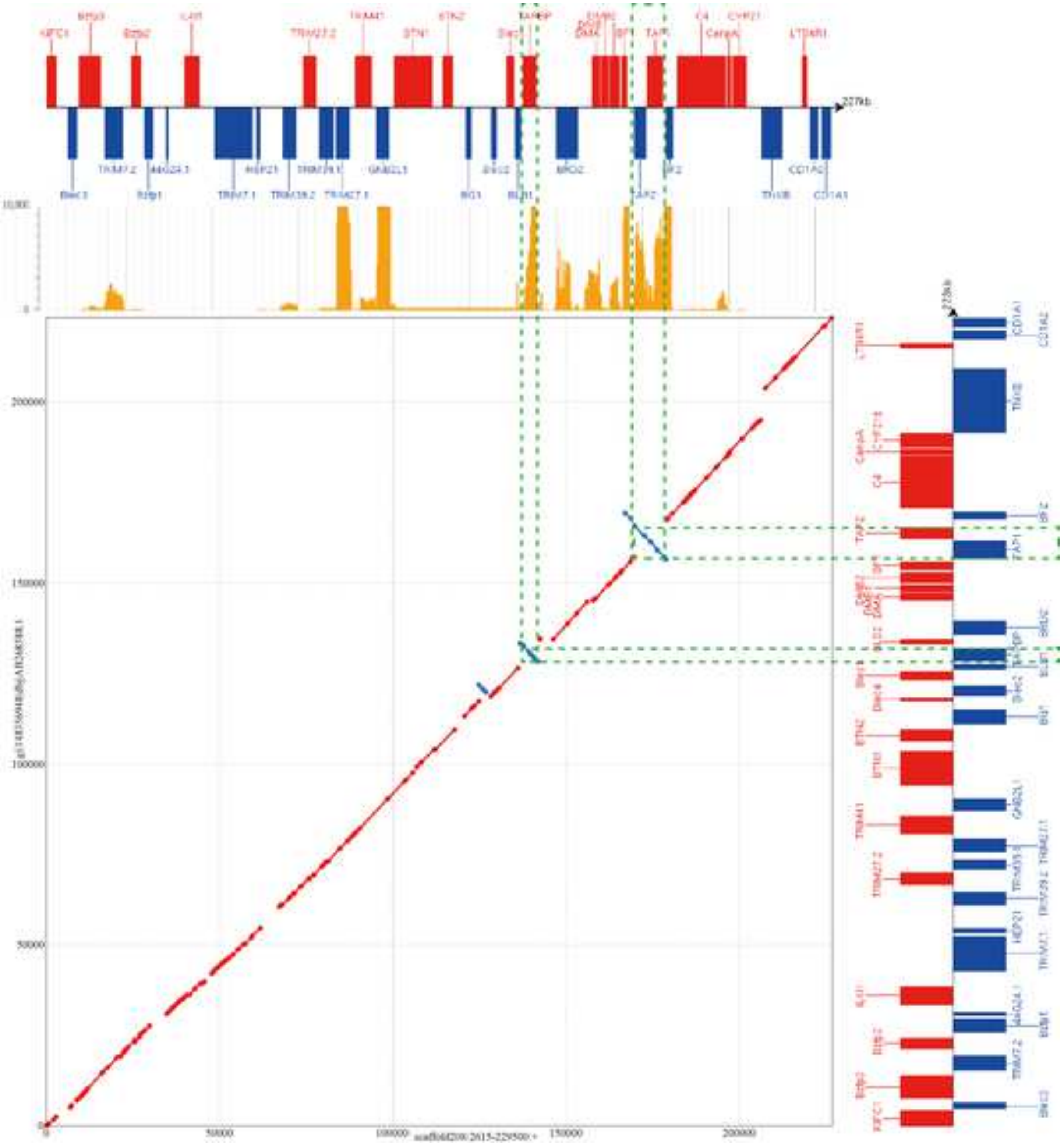

Figure 4

Click here to download Figure Fig4.tif

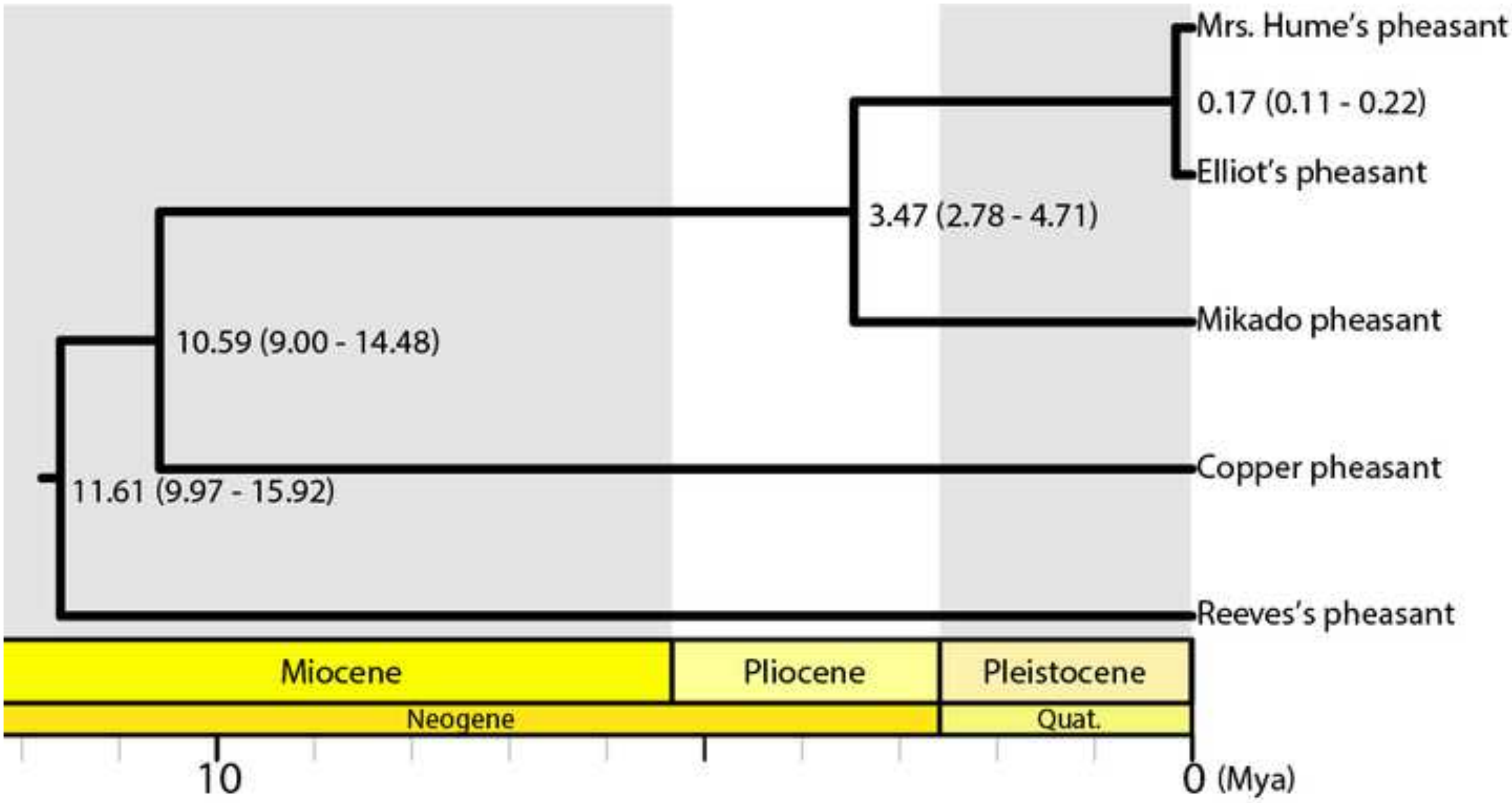

Additional file 1

Click here to access/download
Supplementary Material
Additional file 1.docx

Additional file 2

Click here to access/download
Supplementary Material
Additional file 2.xlsx
