# Supplementary material for: Whole-genome de novo sequencing reveals unique genes that contributed to the adaptive evolution of the Mikado pheasant
Source: Gigascience. 2018 May 2;7(5):giy044. doi: 10.1093/gigascience/giy044 (PMC5941149; doi:10.1093/gigascience/giy044)
Supplement: GIGA-D-17-00220_Revision_1.pdf [file giy044_giga-d-17-00220_revision_1.pdf]

GigaScience

Whole-Genome De Novo Sequencing Reveals Unique Genes that Contributed to the Adaptive Evolution of the Mikado Pheasant

--Manuscript Draft--

**Manuscript Number:**	GIGA-D-17-00220R1
**Full Title:**	Whole-Genome De Novo Sequencing Reveals Unique Genes that Contributed to the Adaptive Evolution of the Mikado Pheasant
**Article Type:**	Research
**Funding Information:**	Taipei Zoo (No. 13, 2015 Animal Adoption Programs of Taipei Zoo) — Dr. Eric Y. Chuang
**Abstract:**	Background: The Mikado pheasant (Syrmaticus mikado) is a nearly endangered species indigenous to high-altitude regions of Taiwan. This pheasant provides an opportunity to investigate evolutionary processes following geographic isolation. Currently, the genetic background and adaptive evolution of the Mikado pheasant remain unclear.<br>Results: We present the draft genome of the Mikado pheasant, which consists of 1.04 Gb of DNA and 15 972 annotated protein-coding genes. The Mikado pheasant displays expansion and positive selection of genes related to features that contribute to its adaptive evolution, such as energy metabolism, oxygen transport, hemoglobin binding, radiation response, immune response, and DNA repair. To investigate the molecular evolution of the major histocompatibility complex (MHC) across several avian species, 39 putative genes spanning 227 kb on a contiguous region were annotated and manually curated. The MHC loci of the pheasant revealed a high level of synteny, several rapidly evolving genes, and inverse regions compared to the same loci in the chicken. The complete mitochondrial genome was also sequenced, assembled, and compared against 4 other long-tailed pheasants. The results from molecular clock analysis suggest that ancestors of the Mikado pheasant migrated from the north to Taiwan about 3.47 million years ago.<br>Conclusions: This study provides a valuable genomic resource for the Mikado pheasant, insights into its adaptation to high altitude, and the evolutionary history of the genus Syrmaticus, which could potentially be useful for future studies investigating molecular evolution, genomics, ecology, and immunogenetics.
**Corresponding Author:**	Eric Y. Chuang<br><br>TAIWAN
**Corresponding Author Secondary Information:**	
**Corresponding Author's Institution:**	
**Corresponding Author's Secondary Institution:**	
**First Author:**	Chien-Yueh Lee
**First Author Secondary Information:**	
**Order of Authors:**	Chien-Yueh Lee<br>Ping-Han Hsieh<br>Li-Mei Chiang<br>Amrita Chattopadhyay<br>Kuan-Yi Li<br>Yi-Fang Lee<br>Tzu-Pin Lu

Powered by Editorial Manager® and ProduXion Manager® from Aries Systems Corporation

	Liang-Chuan Lai
	En-Chung Lin
	Hsinyu Lee
	Shih-Torng Ding
	Mong-Hsun Tsai
	Chien-Yu Chen
	Eric Y. Chuang
**Order of Authors Secondary Information:**	

Response to Reviewers:

Editor suggestions:

Please check for potential contamination, along the lines suggested by reviewer 2, and describe this step in the manuscript. Among other points, reviewer 1 also remarks that only mitochondrial data has been used for divergence time estimates - this should be discussed as a potential limitation in the revised manuscript.
Reviewer 3 has an important point regarding errors in the gal4 assembly - please carefully address this point, as it may affect your conclusions.

Response: Thank you for returning the constructive and useful comments from the three reviewers. Following the suggestion from Reviewer #2, contamination testing for sequencing reads was performed, and 31 contaminated scaffolds were identified and removed from the draft genome. Please refer to response 2-11 for the details. On Reviewer #1’s suggestion, since nuclear genome data of the copper pheasant is not available now, we could only incorporate the five long-tailed pheasants into our analysis on the mitochondrial level. Discussion about the potential impact of this on the divergence time estimate has been added to the revised manuscript. Please refer to response 1-2 for the details. On Reviewer #3’s point, instead of the chr16 sequence from the gal4 or gal5 genome references, we used the chicken MHC-B sequence in our manuscript. The sequence was obtained from GenBank (AB268588.1) and it was produced by Sanger sequencing technology. We apologize for only mentioning it in the legend of Figure 3, but not clearly noting it in the manuscript. The sentence has been rephrased in the revised manuscript to clarify this point. Also, a result identical to that of our previous analysis was obtained after we realigned the MHC region between the Mikado pheasant and chr16 of gal5 (NC_006103.4). Please refer to response 3-2 for the details. All these points are fully addressed, and they do not affect our conclusions.

Overall, the reviewers point out quite a large number of minor inaccuracies or places where further information is needed - please make an effort to address all of these points, as it will help to make your work more clear and more reproducible.

Response: We are grateful for the editor’s and reviewers’ helpful suggestions on our manuscript. All points have been fully addressed as described below.

Another minor issue: In the first paragraph of the abstract, you write "... adaptive behaviors of the Mikado pheasant .." - do you actually mean animal behaviour here? Or rather something like "patterns of adaptive evolution"?

Response: We apologize for the confusion and we have replaced all instances of “behavior” with “evolution” in the revised manuscript.

Please also clarify in the manuscript whether you had approval of an ethics committee and /or , if applicable, permission of wildlife authorities (if the bird was caught in the wild for this study?) for the animal research.

Response: We thank you for your reminding us of this requirement. We have added a statement on ethics approval and consent in the Declarations section.

Reviewer reports:

Powered by Editorial Manager® and ProduXion Manager® from Aries Systems Corporation

Reviewer #1: The manuscript entitled "Whole-Genome De Nova Sequencing Reveals Unique Genes that Contributed to the Adaptive Evolution of the Mikado Pheasant" utilizes the nuclear and mitochondrial genomes to identify genes related to adaptation and immunity. Furthermore, they use nuclear genome genes to reconstruct the phylogenetic position of the Mikado Pheasant among birds with sequenced genomes and estimate the divergence time using mitochondrial genomes of long-tailed pheasants. The manuscript is in very good shape and I have mostly minor comments (see below). Generally, I thought the Results section could be better presented, but the Discussion section was very well written and really brings the significance of these findings to light.

Response: We appreciate the favorable comments and have addressed each issue as described below.

Major comments

1-1: One critical issue I had with the results was the use of separate analyses for the identification of PSGs - 5 vs. 50 species. Can you remove one of these analyses? Or if you decide to retain both, I think a couple of statements about how many PSGs and GO terms overlap and a explanation for their use is required.

Response: We appreciate the suggestion and agree with the reviewer that the identification of PSGs from 50 avian species here may cause confusion. To emphasize the main purpose of the manuscript, we have removed the analysis of PSGs in 50 avian species, including the description of the branch model used for this analysis in the “Examination of genes under positive selection and enrichment analysis” paragraph in the Materials and Methods section, from the revised manuscript.

1-2: Also, for the divergence time estimate using mitochondrial genomes - Is there any nuclear genome data for the other long-tailed pheasants that can be incorporated into this analysis? If not, please discuss how the use of only mitochondrial data may affect the divergence time estimates.

Response: We appreciate the suggestion. Currently, there is no nuclear genome data available for the copper pheasant, so unfortunately, incorporating all five long-tailed pheasants into our analysis using nuclear genomes is impossible at present. For the other four pheasants, however, Wang N. et al. [1] used six nuclear intron and two mitochondrial gene sequences to construct a phylogenetic tree, and its topology was consistent with our result. Our estimate of the divergence time was more precise, considering that we employed complete mitochondrial genomes in the reconstruction of a high-resolution tree for the Syrmaticus genus instead of a few mitochondrial genes. Our estimated divergence time is also supported by the paleogeographical report of Taiwan island formation. Despite these corroborations of the proposed tree topology and estimated divergence time, the use of only mitochondrial data may be considered as a potential limitation. Going forward, it will be necessary to analyze the nuclear genome to obtain further insights into the evolution history of long-tailed pheasants. We have added the paragraph above into the Discussion section.

Minor comments

Abstract

1-3: Line 78: The second sentence in abstract Background is oddly worded. Please revise. A variant of this sentence is also in Background (line 117).

Response: We apologize for the confusion. We have rephrased the sentence in the abstract section as: “This pheasant provides an opportunity to investigate evolutionary processes following geographic isolation.” and the sentence in the background section as: “The Mikado pheasant possesses ideal characteristics for evolutionary research because of its flightlessness and habitat isolation.”

1-4: Line 89: "mitochondrial genome was further" would sound better written as "mitochondrial genome was ALSO"

Response: The correction was made.

Background

1-5: Lines 112-116: I think that genome resources for endangered species can provide great insight into effective population size. This should be added here.

Response: We appreciate the suggestion, and the suggested text has been added.

1-6: Line 119: "the Syrmaticus genus and belongs" should be written as "the Syrmaticus genus and FORMS (or comprises)"

Response: The correction was made.

1-7: Line 131: change to "insights into its adaptive mechanisms." Remove "of the pheasant".

Response: The correction was made.

Results

1-8: Lines 233-234: "between 21.4 and 28.9 million years ago" The Figure has different values - 18.3-27.9. Is the text or figure correct?

Response: We apologize for the confusion. The age between 21.4 and 28.9 million years ago indicates the divergence time of the Phasianidae lineage including 3 birds, Mikado pheasant, turkey, and chicken. The values (18.3-27.9 Mya) in Figure 2 in the revised manuscript represents 95% confidence intervals of the divergence time between the Mikado pheasant and turkey. To avoid the confusion, the sentence was revised to “The estimated time of the Mikado pheasant-turkey divergence was 21.4 million years ago (Mya); the divergence time between chicken and the sister clade of the Mikado pheasant-turkey was estimated at 28.9 Mya.”

1-9: Lines 262-265: Please rewrite this first sentence as it is awkward.

Response: We apologize for the confusion. We have rephrased the sentence as: “To detect the genes that evolved rapidly due to positive selection under the influence of high elevation (Mikado pheasant) as opposed to low elevation (chicken, turkey, duck, and zebra finch), …”

1-10: Line 266: How many PSGs were identified? Please list the number in the text.

Response: We apologize for the confusion. There were 889 PSGs identified and enriched in GO functions. We have added the number in the text.

1-11: Lines 270-272: It should be noted here that PSGs enriched for metabolism constituted the highest number of PSGs as that information is buried in the supplemental files.

Response: A correction was made.

1-12: Lines 327-329, first sentence. The use of "Recently" seems odd as there has been significant work looking at dN/dS ratios in relationship to MHC. Please consider adding more references here and removing "Recently".

Response: We appreciate the suggestion. A correction was made and two references from Harmit Malik's work have been added to strengthen this point.

Reviewer #2: In their study titled "Whole-Genome De Novo Sequencing Reveals Unique Genes that Contributed to the Adaptive Evolution of the Mikado Pheasant", Lee, Hsieh et al. describe a newly sequenced bird genome - which is always a good resource - including some comparative genomics studies. I believe that this work is solid and clearly explained, and as such is of interest and in the scope of GigaScience. I do have some (mostly minor) comments detailed below that I believe would increase

the quality and clarity of the manuscript.

Response: We appreciate the favorable comments.

GENERAL COMMENTS:

2-1: - The use of the word "behavior" (adaptive behavior) is misleading. It seems that the authors mean it in the context of adaptive evolutionary history, and I would suggest to reformulate for accuracy (Abstract and Introduction p5 l129).

Response: We apologize for the confusion and have changed the word “behavior” to “evolution” both in the abstract and the introduction (background).

2-2: - some figures could be improved by having more information on the figure instead of in the legend (mostly Figure 3, see detailed comments below)
2-3: - The significance of some data could be improved at a few locations (see detailed comments below)

Responses to 2-2 & 2-3: We appreciate these suggestions and have addressed each issue as described below.

DETAILED COMMENTS:

ABSTRACT:
2-4: - The authors emphasize in the abstract the details of their data about the MHC & comparison with chicken: having less details but more scope / significance would improve the abstract.

Response: We appreciate the suggestion and have rephrased the sentence as: “To investigate the molecular evolution of the major histocompatibility complex (MHC) across several avian species, 39 putative genes spanning 227 kb on a contiguous region were annotated and manually curated. The MHC loci of the pheasant revealed a high level of synteny, several rapidly evolving genes, and inverse regions compared to the same loci in the chicken.”

INTRODUCTION:
2-5: - p4 l114: what does "behavioral attributes" means here?

Response: We apologize for the confusion. According to the reference paper, Diana Le Duc et al. reported a nocturnal lifestyle in kiwi [2]. We believe that this is an example of a behavioral attribute successfully identified by the genome assembly approach.

2-6: - p4 l114-117: consider splitting this sentence.

Response: We appreciate the suggestion and have made the correction.

2-7: - p5 l1: how was hypoxic stress observed? Is there any citation? Or is this an expectation/extrapolation?

Response: We apologize for the confusion, and this is our expectation/extrapolation. We have removed the word “is” and replaced it with “may be.”

2-8: - p5 l138-141: same comment as abstract.

Response: The correction was made.

DATA DESCRIPTION:
2-9: - Refer to the Method section at least one time at the beginning of this section.

Response: We appreciate the suggestion and have added the following descriptive text at the beginning of the Data Description section.
‘The details about sample collection, library construction, sequencing, assembly, gene

prediction, and annotation can be found in the “Materials and Methods” section.’

2-10: - p6 l156-158: please revise the formulation of this sentence for clarity. Fig S4 shows that there are in fact a lot of scaffolds with short length, even if indeed most of the genome size is assembled in large scaffolds.

Response: We apologize for the confusion. We have rephrased the sentence as: “… showed that most of the draft genome consisted of large scaffolds; though many short scaffolds were present, they only contributed a small portion of the genome size.”

RESULTS:
2-11: - p7 l 176-180: was there a step to verify that the sequencing samples were not contaminated? For example, the bald eagle genome assembly (file from Zhang et al. (2014), Science) has hundreds of bacterial contigs in it (absent from the refseq version because very short), coming from 2 samples contaminated with Yersinia (SRR1176808 and SRR1176809). This can be checked quickly with some software such as Kraken or Taxonomer (with www.taxonomer.com - note that for this website, for a bird genome reads would be nearly all unknown or ambiguous). I could not find the data on the SRA at the time of reviewing to look myself.

Response: We appreciate the suggestion. We used Kraken, combining the approaches of aligning reads against both the chicken genome and the assembled genome, as well as alignment against BLAST’s non-redundant nucleotide sequences (NT) database, to perform the post-check for contamination in our assembled genome (Fig. R1 below; S12 in the revised manuscript). In this way, we obtained 31 contaminated scaffolds with 12 587 bp (~0.001% of the total length) including 290 649 (0.088% of total reads) and 300 871 (0.095% of total reads) reads in the 280-bp and 480-bp libraries, respectively. The major contaminating species were phiX174 and E. coli. We then removed these 31 contaminated scaffolds and a related gene, which had neither an annotation nor a classified gene family. Thus, our conclusions in this study were robust and they were not affected by the contamination problem. We have added a new paragraph to describe the details at the end of the “De novo genome assembly” paragraph in the Materials and Methods section.
‘To examine sequencing reads for potential contamination, we used Kraken (version 1.0) [78] with the standard Kraken database to check the paired-end DNA libraries. Classified reads reported by Kraken were further examined using our proposed pipeline (Additional file 1: Fig. S12). Briefly, we employed Bowtie 2 (version 2.3.0) [79] to align these classified reads against the chicken genome reference (Galgal 5.0) downloaded from Ensembl (release 90), collecting unmapped reads and using Bowtie 2 again to align them against the assembled genome of the Mikado pheasant. We then took those reads mapped onto the Mikado pheasant genome and performed BLASTN alignment against the non-redundant nucleotide sequences (NT) database, downloaded from NCBI’s FTP site (on Nov. 16, 2017), using parameters “-outfmt '6 std staxids' -max_target_seqs 1 -evalue 1E-10.” Next, we collected reads with alignment length ≥100 bp (i.e., two thirds of read length), filtering out the reads matching an avian species or with a read count <50 in a species. The remaining reads were counted and the contaminated scaffolds calculated by applying a cutoff of a read count >20 on a given scaffold. Finally, we removed 31 contaminated scaffolds with 12 587 bp (~0.001% of the total length) from the assembled genome.’

2-12: - p7 l180-184: unless reads were excluded when mapping at multiple locations, do (some) high coverage regions correspond to repeats?

Response: We appreciate the question. To minimize the effect of repeat sequences, we performed Bowtie 2 alignment with the best alignment of each read to calculate the per-base alignment coverage. (Please refer to response 2-41 for more details.)

2-13: - p8 l202: This sentence would be more clear if "with pheasant scaffolds" was added after "The identities of each chicken chromosome"

Response: We appreciate the suggestion, and the sentence has been rephrased as: “The identities of each chicken chromosome with the scaffolds of Mikado pheasant …”

2-14: - p8 l208: if this is notable, what is the significance?

Response: Intrachromosomal inversions occur frequently within avian genomes [3-5]. Despite the concrete mechanism being unclear, inversion is thought to play an important role in avian genome evolution, serving as a driver of speciation [6, 7]. For example, a recent study reported that some intrachromosomal inversions in the white-throated sparrow were related to behavioral attributes and feather features [8]. Our manuscript describes the first genome-wide analysis to identify multiple intrachromosomal inversions between the Mikado pheasant and chicken genomes.

2-15: - p8 l217: as expected?

Response: Yes, according to the phylogenetic tree (Fig. 2 in the revised manuscript) and molecular evidence from recent reports [9, 10], the Mikado pheasant is more closely related to the Galliformes order than to the Passeriformes order. Therefore, we can expect that the Mikado pheasant scaffolds were poorly aligned with the zebra finch genome.

2-16: - p8 l218-221: consider having the mention of "high frequency of potentially highly conserved regions" before the "but", to contrast conservation and dynamics.

Response: We appreciate the suggestion, and the sentence has been rephrased as: "In general, the Mikado scaffolds displayed high conservation with the genomes of chicken and turkey. We also observed several intrachromosomal inversions and chromosomal translocations. This is the first genome-wide analysis to identify multiple intrachromosomal inversions between the Mikado pheasant and chicken genomes."

2-17: - p8 l 220: what are the "high frequency" numbers? How does this compare to the literature, if any similar other research?

Response: We apologize for the confusion. Originally, the "high frequency" denoted that the Mikado genome showed high conservation with the genomes of chicken and turkey. To avoid the confusion, we have rephrased the sentence as described above (please refer to response 2-16).

2-18: - p9 l229-230: The formulation here is confusing and should be revised to illustrate better that the 18 220 gene families (as mentioned in the legend of Figure 2) are for all species considered (and not just the Mikado pheasant) - since Figure S8 shows different numers. Additionally, the number of genes is lower than the number of annotated genes mentioned in the manuscript or than the one in Figure S8; why these three different values?

Response: We apologize for the confusion. The sentence has been rephrased to match the legend of Fig. 2. There are two possible reasons for the different gene numbers from these analyses in the Mikado pheasant. First, the gene families from 10 species (Fig. 2 in the revised manuscript) or 5 birds (Fig. S8) were classified by OrthoMCL using the protein sequences from Ensembl. Considering the phylogenetic relationship, the E-value cutoff for running all-vs-all BLASTP was stricter in the analysis of 5 birds (1e-20) than in that of 10 species (1e-5) (please refer to Gene families in the Materials and Methods section). Thus, the number of genes in the Mikado pheasant was less in the analysis of 5 birds (14 375 genes) than in the 10 species (15 161 genes). Second, we used a completely different source—the Aves and Reptilians protein sequences from the NCBI NR database—to annotate 15 972 genes in the Mikado pheasant. Although these methods produced different numbers of genes, we believe that the numbers are in a reasonable range for the avian genome, based on a previous study [11].

2-19: - p9 l245: are fragmented annotations a possible issue here? i.e. are longer genes enriched or not in expanded families?

Response: Yes, we believe that fragmented annotations of longer genes may cause the evolutionary rates of expanded families to be overestimated. To reduce the potential errors, we used CAFE 3 to identify expanded and contracted genes in the study (see Gene families in the Materials and Methods section). The authors of CAFE 3 claim that they applied phylogenetic tree information to model the observed family

sizes in the algorithm, which could recover accurate evolutionary rates of gene families with fragmented annotations [12].

2-20: - p9 l246: Are the numbers / rates surprising or not based on the literature?

Response: We did not expect so many gene ontology (GO) categories to be identified. However, the identified GO functions provided straightforward evidence to explain the Mikado pheasant's adaptation to high altitude.

2-21: - p9 l248-259: what about the ones in the chicken for example? And other birds?

Response: Analyzing genes with expansion and contraction is an approach to identify the gene number changes in each gene family. To infer these changes for a specific combination of interest, for example the Mikado pheasant versus chicken, would be ill-advised, due to the limitations of the statistical test provided by CAFE 3. The expansion and contraction can only be identified significantly between a specific species and its common ancestor. Based on the tree topology in Figure 2 in the revised manuscript, for example, chicken can only be compared with the node (labeled 28.9 Mya) which is the common ancestor of chicken and the other node (labeled 21.4 Mya; the common ancestor of the Mikado pheasant and turkey)—neither the Mikado pheasant, nor the turkey itself. For this reason, we cannot directly identify expanded/contracted genes between the Mikado pheasant and chicken/other birds.

2-22: - p9 l258: is 8/75 surprising? What is the fraction of all olfactory receptors among all gene families? Were there more olfactory receptors annotated in the pheasant than other birds? E.g. discuss based on the data from Steiger et al 2008 (DOI: 10.1098/rspb.2008.0607), or other literature if any.

Response: 1) Among the 75 expanded gene families of Mikado pheasant, 8 gene families were annotated as olfactory receptors (ORs). Since the proportion exceeds ten percent, we mentioned this finding in the manuscript to provide the result as a numeric basis for possible comparisons in future studies.
2) There were 12 549 gene families in the Mikado pheasant (total 18 220 gene families in the 10 species). Of these gene families, 44 were OR-related (with 65 genes predicted to be ORs).
3) Steiger et al. compared nine bird species from seven orders (blue tit, black coucal, brown kiwi, canary, galah, red jungle fowl, kakapo, mallard, and snow petrel) and drew the conclusion that the estimated total number of OR genes correlates positively with olfactory capability. However, some of the birds did not have an assembled draft genome at the time of the paper's publication, and some of the OR gene numbers might be overestimated by the authors. For instance, the paper displayed 600 estimated OR genes in the brown kiwi, but there were only 141 presented (82 OR genes were identified from the initial prediction) in a subsequent study when the genome sequence was available [2]. Despite these limitations, we can still compare our result with the kiwi [2]. There were more genes predicted to be ORs in the kiwi (N=82) than in the Mikado pheasant (N=65). However, this difference should not be overinterpreted, since ORs are highly duplicated across the genome, which may produce more overcollapsed contigs during the assembly process. This is a general problem in the short-read sequencing technology.

2-23: - p10 l262: this formulation is unclear: "because of living at and between high and low elevation".

Response: We apologize for the confusion. We have removed the unclear sentence and rephrased as: "To detect the genes that evolve rapidly due to positive selection under the influence of high elevation (Mikado pheasant) as opposed to low elevation (chicken, turkey, duck, and zebra finch), …"

2-24: - p10 l264: Since these 7132 orthologues seem to be the same as the 7132 single-gene families mentioned in Methods, the change of terminology (gene family v.s. orthologs) is confusing (maybe use orthologs for single-gene families that were also annotated as orthologs by OrthoMCL, and gene families for the others?).

Response: We apologize for the confusion. Gene families contain orthologs and

paralogs. Orthologs (or orthologous genes) indicate genes with similar sequences in different species, whereas paralogs (not part of this study) indicate genes with similar sequences from within the same species. Specifically, orthologs from within a gene family having one gene for each species are called single-copy orthologs (or single-gene families). In the manuscript, we classified gene families using OrthoMCL, and further identified single-copy orthologs from these gene families to construct a phylogenetic tree and analyze positively selected genes. To avoid confusion, we have unified the terminology and used “gene families” and “orthologs” in the revised manuscript.

2-25: - p11 l293: since the Jak-STAT pathway is not mentioned again in discussion, please add why this is worth noticing.

Response: We appreciate the suggestion. The following sentence for discussing the Jak-STAT pathway has been added in the Discussion section.
“Some of these PSGs were also involved in the Jak-STAT signaling pathway (Additional file 1: Table S15), which participates in chemical signal transmission and induces cellular stress responses, such as immunity, apoptosis, [61, 62], and hypoxia [63]. All these results provide wider support for the adaptive evolution of the Mikado pheasant.”

2-26: - p11 l301: this number of 5287 orthologs between 48 birds is identical to the one of orthologs identified in 10 species (with mammals) - please check that this is accurate.

Response: We apologize for the mistake. The correct number of orthologs is 2209.

2-27: - p12 l305: the ubiquitin activity is not mentioned in discussion: what would be the significance of having expanded gene families associated with this GO term?

Response: The GO term associated with ubiquitin activity is associated with the degradation of proteins. The ubiquitin will mark the target protein by forming an isopeptide bond to the lysine residues on the protein. The complex will be sent to the proteasome, and the proteins will be subsequently degraded. Currently, few studies have reported the relationship between ubiquitin activity and phenotype in avian species. Thus, we have insufficient evidence to explain the enrichment of ubiquitin activity in the Mikado pheasant. To emphasize the main purpose of the manuscript, in response to Reviewer #1’s suggestion, we have removed the results of the analysis of positively selected genes in 50 avian species from the Results section.

2-28: - p12 l320: Methods says MAKER, not manual curation; was MAKER used and then the annotations manually curated?

Response: Yes, MAKER was used to predict potential MHC-B genes, and then these genes were manually curated. We apologize for the confusion and have added a new paragraph to describe the details at the end of the “Gene prediction and annotation” paragraph in the Materials and Methods section.
“For MHC-B annotation and curation, we first took the scaffold208 sequence and used MAKER (version 2.31.8) [88] to predict the potential gene structures of MHC-B genes. Next, the RNA-Seq libraries from the Mikado pheasant and the homologous protein sequences from chicken and turkey were aligned to these predicted regions. Finally, we used Web Apollo (version 2.0.3), a web-based and visualization tool for curation and annotation, to manually curate these genes according to the alignment evidence.”

2-29: - p12 l329: there is more general literature on this question (e.g. Harmit Malik's work and others); adding one or two references would strengthen this point.

Response: We appreciate the suggestion. The two references from Harmit Malik's work have been added to strengthen this point.

2-30: - p13 l330: BLB2 is mentioned here (probably because found in RNAseq data?), but it is is missing from the Figure and afterwards said missing from the Mikado pheasant assembly, which is confusing. Maybe the lines 445 to 451 should be part of this result section instead?

Response: We appreciate the suggestion. We have moved this part to the Results section.

2-31: - p13 l331: see comment about Figure 3

Response: A correction was made (please refer to response 2-48).

2-32: - p13 l240: significance of inversions?

Response: Yes, there are several MHC-related studies reporting that, in the Galliformes order, the TAPBP and/or TAP1-TAP2 blocks are in inverse orientation [13-15]. Wang B. et al. even proposed a hypothesis of MHC evolutionary history in black grouse based on these inversions [16]. In our study, it is the first time to observe them in the Mikado pheasant, and we believe that this finding will have a profound influence on studies of the evolutionary history of the avian MHC.

DISCUSSION:
2-33: - p14 l375: since these extra steps are not detailed in the Method section, the parameters and versions should figure in Table S18 or in additional info.

We apologize for the unclear statement. We have rephrased the paragraph in the Materials and Methods section as follows.
"The quality of the raw reads was examined using FastQC (version 0.10.1). Trimmomatic (version 0.30; parameters: "ILLUMINACLIP:TruSeq3-PE.fa:2:30:15 SLIDINGWINDOW:4:20 MINLEN:100") [76] and NextClip (version 1.3.1) [77] with default parameters were used to trim sequencing reads. Genome assembly into contigs was performed by MaSuRCA (version 2.3.2) [15] with settings based on the instruction manual. ALLPATHS-LG (version 49722) [43], Newbler (version 2.9) [45] both with default parameters, JR (version 1.0.4; parameters: "-minOverlap 60 -maxOverlap 90 -ratio 0.3") [44], SGA (version 0.10.13; parameters: "assemble -m 125 -d 0.4 -g 0.1 -r 10 -l 200") [46], and SOAPdenovo (version 2.04; parameters: "-K 47 -R") [47] were also used to assemble contigs. We employed SSPACE (version 3.0; parameter: "-z 300") [74] to construct scaffolds for the draft genome. In this step, mate pair libraries with 35 bases from the 5'end of both reads were used for scaffolding. Scaffold sequences shorter than 300 bp were then excluded from the final assembly. The statistical results of the assembly were estimated using QUAST (version 3.2) [75]."

2-34: - p15 l387: how were the number of misassembled or fragmented sequences estimated and distinguished from real differences with the chicken genome (since Fig1 is referred to)?

Response: We apologize for the carelessness. We realize that it is difficult to estimate the number of misassembled or fragmented sequences from the information in Fig. 1 only. The degree of fragmented sequences may be distinguished by the composition of lines and points from Fig. 1A; on the other hand, we can expect that the more points on a syntenic map, the more fragmented sequences exist. To clarify the statement, we have rephrased the sentence as: "Although scaffolds of the draft genome displayed some degree of fragmentation (Fig. 1A) and showed translocation (Fig. 1B) in certain chicken chromosomes, …"

2-35: - p17 l438-441: is there any evidence that these inversions affect the expression of these genes...?

Response: No, there is no study reporting a correlation between these inversions and their gene expression.

2-36: - p17 l445-451: see comment for p13 l330.

Response: A correction was made (please refer to response 2-30).

2-37: - p17 l452: "the whole genome of a genus" would read better as "the whole genome of a bird of the genus"

Powered by Editorial Manager® and ProduXion Manager® from Aries Systems Corporation

Response: A correction was made.

MATERIAL AND METHODS:
2-38: - p19 l486: were both experiments done on pooled RNA from the 2 males, or was there one male per RNAseq experiment?

Response: We apologize for the confusion. There was one male individual per RNA-Seq experiment.

2-39: - p19 l494: FastQC version and exact tools and parameters used to trim reads and remove adapters?

Response: FastQC version is 0.10.1. The paired-end and mate pair reads were trimmed and adapters removed by Trimmomatic (version 0.30; parameters: “ILLUMINACLIP:TruSeq3-PE.fa:2:30:15 SLIDINGWINDOW:4:20 MINLEN:100”) and NextClip (version 1.3.1) with default parameters, respectively. This information is included in the revised text.

2-40: - p19 l496: MaSuRCA reference missing here (even if elsewhere in the ms): Zimin, A. et al. Bioinformatics (2013). doi:10.1093/bioinformatics/btt476

Response: We appreciate the information and a correction was made.

2-41: - p20 l509: were the software's default parameters used? What were the parameters regarding non uniquely mapping reads?

Response: Yes, default parameters were used for both Bowtie 2 and TopHat2. When evaluating per-base alignment coverage and mapping rates for DNA reads, only the best alignment for each read was taken into account. However, when evaluating mapping rates for RNA reads, the non-uniquely mapped reads were considered by TopHat2. Table R1 shows the detailed information for the multi-read alignment. In addition, to improve RNA-Seq mapping rates at Reviewer #3’s suggestion, we have replaced Table S5 in Additional file 1 with the results using the STAR alignment program in the revised manuscript.

Table R1: Summary of RNA read mapping rates using TopHat2.

	RNA Sample 1	RNA Sample 2
Reads		
Overall mapping rate (left)†	92.5%	84.4%
Multiple mapping rate (left)	6.6%	7.5%
Overall mapping rate (right)†	91.4%	77.3%
Multiple mapping rate (right)	6.6%	7.6%
Pairs		
Concordant mapping rate*	88.1%	72.4%
Multiple mapping rate	6.7%	7.7%

† Overall mapping rate stands for the ratio of total mapped reads to total reads.
* Mapped concordantly means the read pairs were aligned to the genome with the expected distances and orientation.

2-42: - p10 l513: Version of BEDTools is missing.

Response: The BEDTools version was 2.23.0. This information is included in the revised text.

2-43: - p21 l533: RepeatMasker version, parameters and library used are missing.

Response: We used RepeatMasker (version 4.0.5, parameter: “-species chicken”), including rmblastn (version 2.2.23+) as the search engine, RepBase (version 20140131), and RM database (version 20140131), to identify repeat regions. This information is included in the revised text.

2-44: - p22 l565: consider adding the numbers of genes and gene families identified that are not single genes before switching to the Method of ortholog identification.

Powered by Editorial Manager® and ProduXion Manager® from Aries Systems Corporation

Response: We appreciate the suggestion. We have added gene and gene family numbers in the sentence as follows.
“Then, 18 220 gene families (including 5287 single-copy orthologs) were obtained from the 10 species, and 13 436 gene families (including 7132 single-copy orthologs) were obtained from the 5 birds by OrthoMCL (version 2.0.9) using default parameters. In the analysis of the 10 different species, 15 161 genes of the Mikado pheasant were grouped into 12 549 gene families. In the analysis of the 5 avian species, 14 375 Mikado pheasant genes were grouped into 12 078 gene families.”

2-45: - p22 l573: bootstraps?

Response: We performed RAxML with 500 bootstrap replicates. This information is included in the revised text.

FIGURES:
FIGURE 1B:
2-46: - point/label differently on the figure scaffolds 1 and 45 (since mentioned in the text), and maybe also the ones that fully align to chicken chromosomes?

Response: We appreciate the suggestion. We have added arrows colored in yellow to indicate scaffolds that fully aligned to the chicken chromosomes; grey arrows are added to point out the multiple alignment ones.

FIGURE2:
2-47: - consider adding numbers per My, to facilitate the comparison between branches.

Response: A correction was made.

FIGURE3:
2-48: - it would help the reader a lot if instead of having a color code for forward/reverse (that information is already coded by the position above or under the bar), the genes could be color coded based on their dN/dS ratios.

Response: We appreciate the suggestion. A dN/dS ratio bar chart has been added under the gene structure boxes to enhance the readability of Figure 3. At the same time, the forward/reverse color codes are retained, since it clearly visualized the gene orientation.

2-49: - the coverage scale does not allow to see lower coverage genes; consider using a log scale?

Response: A correction was made.

SUPP DATA:
2-50: FIGURE S2: does 'habitats available' correspond to where they are found generally, or a protected habitat?

Response: The primary habitats available for the Mikado pheasant include both areas where they are generally found and protected habitats. Regions in national parks are protected habitats, but Shuanggueihu and Tawushan are areas where they are generally found.

2-51: FIGURE S8: see comment for Results p9 l230.

Response: Values in the figure are correct. Please refer to responses 2-18 and 2-44 for more details.

2-52: TABLE S1: which RNAseq was HiSeq and which was HiSanSQ?

Response: We apologize for the confusion and we have added platform information in Table S1.

Powered by Editorial Manager® and ProduXion Manager® from Aries Systems Corporation

	2-53: TABLE S9: for ex. for the GO:0002504 line, could these annotations be missing from the assembly or be fragmented? Since there are only 2 genes in this family, it sounds possible.<br><br>Response: We apologize for the confusion. There were 2 gene families involved in the GO:0002504 function rather than 2 genes in this family. These gene families contained genes in other species instead of the Mikado pheasant. One of these gene families showed significant change in its size, which met our expectation of gene contraction in the Mikado pheasant.<br><br>2-54: TABLE S18: see comment about Discussion p14 l375.<br><br>Response: We have added detailed steps, parameters, and versions in the Materials and Methods section, so the table is not changed (please refer to response 2-33).<br><br><br>Reviewer #3: The manuscript presents the genome and gene annotation of the Mikado pheasant (MP), a protected species living in geographical isolation and adapted to high altitude habitats. The genome was assemble into 208.8k contigs (>300 bp) and 9,359 scaffolds (>1 kb) using Illumina short read technology of paired-end and mate-pair libraries. Annotation was generated by ab initio and homology based gene predictions and from short-read RNA-seq data which was followed by defining the phylogenetic position of the species and analyses of gene and gene-family evolution. The study provides a genome resource and annotation for the species and contributes to the understanding of gene family evolution for adaptation to high altitude and immunity in birds.<br><br>3-1: One of the main aims of the authors was to provide a genomic resource for the MP to support future studies of the species and this work fulfils this aim. Properties of the genome sequence (contig/scaffold N50, coverage, repeat content) is very similar to the medium quality bird genome assemblies released by the Avian Phylogenetic Consortium (Zhang G et al. 2014. Science 346: 1311-1320). The annotation approach should be sufficient and the methods used adequate to define the place of the species in the phylogeny of pheasants as it is built on orthologous peptide regions. Nevertheless the fragmented genome assembly will limit the scope of future analyses which can be done with the assembly. Also, the annotation chiefly relies on annotation from orthologous peptides with only limited information coming from transcriptome sequencing. While it is possible to find gene family expansions and contraction events and infer adaptively evolving regions in key genes, many of the adaptations to high altitude can be assumed to happen at changes in regulatory regions modulating levels of gene expressions, neither of which is even mentioned in the study.<br><br>Response: We appreciate the favorable comments. As the reviewer pinpointed, the relation between adaptations to high altitude and changes in regulatory regions modulating levels of gene expression is not mentioned in our manuscript. In this study, we set our sights on the de novo genome assembly of the Mikado pheasant and identification of high-altitude adaptation based on genomic information. Identifying genes related to adaption from the perspective of gene expression and biologically verifying the findings of this study are all potentially interesting topics and can be set as one of our long-term goals in the research of Mikado pheasants. The following paragraph was added into the Discussion section.<br>"To sum up, this study reveals the high-altitude adaptation mechanisms of the Mikado pheasant at the genomic level. However, there are some adaptive mechanisms for high altitude that happen via changes in regulatory regions modulating the levels of gene expression [64-66]. We believe that this is an intriguing topic and worthy of further research to be undertaken in the future."<br><br>3-2: My main concern is with the part of the paper which describes the observed differences in the MHC region between the MP and the gal4 chicken assembly. It is known that chr16 of the gal4 assembly contained errors. Unfortunately the authors failed to mention the presence of these errors and how these would affect their results. Chr 16 has got improved in the gal5 assembly (Warren WC et al. 2016 G3 (Bethesda) 7: 109-117.) and the improved sequence would/could have provided a much better reference for this comparison. If, for this part of the work, the authors would realign the

MHC region between MP and chr16 of gal5 that would make their results more reliable and relevant for the bird communities.

Response: We appreciate the suggestion and apologize for the confusion. Instead of the chr16 sequence from the gal4 or gal5 genome references, the chicken MHC-B sequence that we used to compare with the Mikado pheasant was obtained from GenBank (AB268588.1). The sequence was published by Shiina et al. and analyzed DNA molecules from constructed bacterial artificial chromosome (BAC) clones and long-PCR products by Sanger sequencing technology [17]. To avoid the confusion, we have rephrased the sentence as "…, an assembled scaffold (scaffold208) was almost able to cover the known chicken sequence of the MHC-B contiguous region published by Shiina et al. (GenBank Accession: AB268588.1)."
However, out of respect for the reviewer's comment we also realigned the MHC region between the Mikado pheasant and chr16 of gal5 (NC_006103.4). As shown in Fig. R2, except for the strand orientation, the alignment showed identical results between the AB268588.1 sequence and chr16 of gal5, which proved that our results are reliable.

Apart from the above I found the manuscript generally well written and I only have a few small comments:

3-3: I assumed to find tissue information for the samples from which the genomic short read and RNA-seq data was generated, but could not find it in the materials and methods section (MM).

Response: We apologize for the confusion. Originally, the information on tissue samples was at the beginning of the "De novo genome assembly" paragraph in the Materials and Methods section. To avoid the confusion, the paragraph has been split into an independent paragraph and titled "Sample preparation and sequencing."

3-4: A technical note: TopHat2 was shown to underperform most of the other RNA-seq read mapping softwares (e.g. STAR). As the RNA-seq data is limited and the genome is fragmented the limitations coming from the usage of a "weaker" aligner is probably not that significant for this study.

Response: We appreciate the suggestion. Compared with TopHat2, the mapping rates using STAR (version 2.4.0) with default settings were significantly improved in both of the RNA-Seq samples (Table R2). We have replaced the TopHat2 results with STAR results for the assembly assessment in the revised manuscript.

Table R2: Comparison of RNA read mapping rates using STAR and TopHat2.

	RNA Sample 1	RNA Sample 2
STAR		
Total mapped	95.8%	93.1%
Multiple Mapped	2.04%	2.04%
Uniquely Mapped	93.8%	91.1%
TopHat2		
Mapped concordantly	88.1%	72.4%
Overall mapping rate	91.9%	80.9%

There were a few sentences which I found hard to understand:

3-5: P9: L229. "First, 15 161 Mikado pheasant genes were identified in 18 220 families, and 5287 single-gene families that were common across the 10 species were then used to construct a Bayesian maximum clade credibility phylogenetic tree to estimate the time of divergence"
Do you mean 15,161 genes in 18,220 families? Did you have genes belonging to multiple gene families?

Response: 1) We apologize for the confusion, the 18 220 gene families in total were obtained from the 10 species. There were 15 161 genes of the Mikado pheasant were grouped into 12 549 gene families in the analysis of the 10 different species (please refer to Gene families in the Materials and Methods section). To clarify the statement, we have rephrased the sentence at Reviewer #2's suggestion.

2) No, we performed OrthoMCL to classify gene families. The tool assigned a gene to a gene family.

3-6: P20: L514." Regarding the RNA reads, the mapping rate showed the completeness of the final assembly with respect to the independent sequencing data from the transcriptomes of the Mikado pheasant."

Response: We apologize for the confusion. The sentence has been rephrased as: “Taking the RNA sequencing reads from two individual Mikado pheasants and observing the mapping rate is another approach for assessing the completeness of the assembly.”

References:

1.Wang N, Kimball RT, Braun EL, Liang B and Zhang Z. Assessing phylogenetic relationships among galliformes: a multigene phylogeny with expanded taxon sampling in Phasianidae. PLoS One. 2013;8 5:e64312. doi:10.1371/journal.pone.0064312.
2.Le Duc D, Renaud G, Krishnan A, Almen MS, Huynen L, Prohaska SJ, et al. Kiwi genome provides insights into evolution of a nocturnal lifestyle. Genome biology. 2015;16:147. doi:10.1186/s13059-015-0711-4.
3.Hooper DM and Price TD. Chromosomal inversion differences correlate with range overlap in passerine birds. Nat Ecol Evol. 2017;1 10:1526-34. doi:10.1038/s41559-017-0284-6.
4.Aslam ML, Bastiaansen JW, Crooijmans RP, Vereijken A, Megens HJ and Groenen MA. A SNP based linkage map of the turkey genome reveals multiple intrachromosomal rearrangements between the turkey and chicken genomes. BMC Genomics. 2010;11:647. doi:10.1186/1471-2164-11-647.
5.Zhang G, Li C, Li Q, Li B, Larkin DM, Lee C, et al. Comparative genomics reveals insights into avian genome evolution and adaptation. Science. 2014;346 6215:1311-20. doi:10.1126/science.1251385.
6.Volker M, Backstrom N, Skinner BM, Langley EJ, Bunzey SK, Ellegren H, et al. Copy number variation, chromosome rearrangement, and their association with recombination during avian evolution. Genome research. 2010;20 4:503-11. doi:10.1101/gr.103663.109.
7.Ellegren H. Molecular evolutionary genomics of birds. Cytogenet Genome Res. 2007;117 1-4:120-30. doi:10.1159/000103172.
8.Davis JK, Mittel LB, Lowman JJ, Thomas PJ, Maney DL, Martin CL, et al. Haplotype-based genomic sequencing of a chromosomal polymorphism in the white-throated sparrow (Zonotrichia albicollis). J Hered. 2011;102 4:380-90. doi:10.1093/jhered/esr043.
9.Prum RO, Berv JS, Dornburg A, Field DJ, Townsend JP, Lemmon EM, et al. A comprehensive phylogeny of birds (Aves) using targeted next-generation DNA sequencing. Nature. 2015;526 7574:569-73. doi:10.1038/nature15697.
10.Jarvis ED, Mirarab S, Aberer AJ, Li B, Houde P, Li C, et al. Whole-genome analyses resolve early branches in the tree of life of modern birds. Science. 2014;346 6215:1320-31. doi:10.1126/science.1253451.
11.Zhang G, Li B, Li C, Gilbert MT, Jarvis ED, Wang J, et al. Comparative genomic data of the Avian Phylogenomics Project. Gigascience. 2014;3 1:26. doi:10.1186/2047-217X-3-26.
12.Han MV, Thomas GW, Lugo-Martinez J and Hahn MW. Estimating gene gain and loss rates in the presence of error in genome assembly and annotation using CAFE 3. Mol Biol Evol. 2013;30 8:1987-97. doi:10.1093/molbev/mst100.
13.Ye Q, He K, Wu SY and Wan QH. Isolation of a 97-kb minimal essential MHC B locus from a new reverse-4D BAC library of the golden pheasant. PLoS One. 2012;7 3:e32154. doi:10.1371/journal.pone.0032154.
14.Chaves LD, Krueth SB and Reed KM. Defining the turkey MHC: sequence and genes of the B locus. J Immunol. 2009;183 10:6530-7. doi:10.4049/jimmunol.0901310.
15.Hosomichi K, Shiina T, Suzuki S, Tanaka M, Shimizu S, Iwamoto S, et al. The major histocompatibility complex (Mhc) class IIB region has greater genomic structural flexibility and diversity in the quail than the chicken. BMC Genomics. 2006;7:322. doi:10.1186/1471-2164-7-322.
16.Wang B, Ekblom R, Strand TM, Portela-Bens S and Hoglund J. Sequencing of the core MHC region of black grouse (Tetrao tetrix) and comparative genomics of the galliform MHC. BMC Genomics. 2012;13:553. doi:10.1186/1471-2164-13-553.

Powered by Editorial Manager® and ProduXion Manager® from Aries Systems Corporation

	17.Shiina T, Briles WE, Goto RM, Hosomichi K, Yanagiya K, Shimizu S, et al. Extended gene map reveals tripartite motif, C-type lectin, and Ig superfamily type genes within a subregion of the chicken MHC-B affecting infectious disease. J Immunol. 2007;178 11:7162-72.
**Additional Information:**	
**Question**	**Response**
Are you submitting this manuscript to a special series or article collection?	No
**Experimental design and statistics**<br><br>Full details of the experimental design and statistical methods used should be given in the Methods section, as detailed in our Minimum Standards Reporting Checklist. Information essential to interpreting the data presented should be made available in the figure legends.<br><br>Have you included all the information requested in your manuscript?	Yes
**Resources**<br><br>A description of all resources used, including antibodies, cell lines, animals and software tools, with enough information to allow them to be uniquely identified, should be included in the Methods section. Authors are strongly encouraged to cite Research Resource Identifiers (RRIDs) for antibodies, model organisms and tools, where possible.<br><br>Have you included the information requested as detailed in our Minimum Standards Reporting Checklist?	Yes
**Availability of data and materials**<br><br>All datasets and code on which the conclusions of the paper rely must be either included in your submission or deposited in publicly available repositories (where available and ethically appropriate), referencing such data using a unique identifier in the references and in the "Availability of Data and Materials" section of your manuscript.<br><br>Have you have met the above requirement as detailed in our Minimum Standards Reporting Checklist?	Yes

Powered by Editorial Manager® and ProduXion Manager® from Aries Systems Corporation

Manuscript Click here to download Manuscript MikadoPheasant13_4.docx

Whole-Genome *De Novo* Sequencing Reveals Unique Genes that Contributed to the Adaptive Evolution of the Mikado Pheasant

Chien-Yueh Lee[1†], Ping-Han Hsieh[1†], Li-Mei Chiang[1], Amrita Chattopadhyay[2], Kuan-Yi Li[3,4], Yi-Fang Lee[1], Tzu-Pin Lu[5], Liang-Chuan Lai[6], En-Chung Lin[7], Hsinyu Lee[1,8,9], Shih-Torng Ding[7,9], Mong-Hsun Tsai[2,9,10,11], Chien-Yu Chen[3,9,12*], and Eric Y. Chuang[1,2,5,9,13*]

[1]Graduate Institute of Biomedical Electronics and Bioinformatics, National Taiwan University, Taipei 10617, Taiwan

[2]Bioinformatics and Biostatistics Core, Center of Genomic Medicine, National Taiwan University, Taipei 10055, Taiwan

[3]Department of Bio-Industrial Mechatronics Engineering, National Taiwan University, Taipei 10617, Taiwan

[4]Institute of Plant and Microbial Biology, Academia Sinica, Taipei, 11529, Taiwan

[5]Institute of Epidemiology and Preventive Medicine, National Taiwan University, Taipei 10055, Taiwan

[6]Graduate Institute of Physiology, National Taiwan University, Taipei 10051, Taiwan

[7]Department of Animal Science and Technology, National Taiwan University, Taipei 10617, Taiwan

[8]Department of Life Science, National Taiwan University, Taipei 10617, Taiwan

[9]Center for Biotechnology, National Taiwan University, Taipei 10672, Taiwan

[10]Institute of Biotechnology, National Taiwan University, Taipei 10672, Taiwan

[11]Agricultural Biotechnology Research Center, Academia Sinica, Taipei 11529, Taiwan University, Taipei, Taiwan

[12]Center for Systems Biology, National Taiwan University, Taipei 10672, Taiwan

[13]Graduate Institute of Chinese Medical Science, China Medical University, Taichung 40402, Taiwan

[†] These authors contributed equally to the work.

[*] Corresponding authors:
Eric Y. Chuang
Department of Electrical Engineering, Graduate Institute of Biomedical Electronics and Bioinformatics, National Taiwan University, Taipei 10617, Taiwan
Phone: +886-2-3366-3660, Fax: +886-2-3366-3682, E-mail: chuangey@ntu.edu.tw

Chien-Yu Chen
Department of Bio-Industrial Mechatronics Engineering, National Taiwan University, Taipei 10617, Taiwan
Phone: +886-2-3366-5334, E-mail: chienyuchen@ntu.edu.tw

E-mail addresses

Chien-Yueh Lee: d00945006@ntu.edu.tw
Ping-Han Hsieh: r04945025@ntu.edu.tw
Li-Mei Chiang: dytk2134@gmail.com
Amrita Chattopadhyay: amrita@ntu.edu.tw
Kuan-Yi Li: kyli.tw@gmail.com
Yi-Fang Lee: b01901110@ntu.edu.tw
Tzu-Pin Lu: tbenlu@gmail.com
Liang-Chuan Lai: llai@ntu.edu.tw
En-Chung Lin: eclin@mail2000.com.tw
Hsinyu Lee: hsinyu@ntu.edu.tw
Shih-Torng Ding: sding@ntu.edu.tw
Mong-Hsun Tsai: motiont@ntu.edu.tw
Chien-Yu Chen: chienyuchen@ntu.edu.tw
Eric Y. Chuang: chuangey@ntu.edu.tw

Abstract

Background: The Mikado pheasant (*Syrmaticus mikado*) is a nearly endangered species indigenous to high-altitude regions of Taiwan. This pheasant provides an opportunity to investigate evolutionary processes following geographic isolation. Currently, the genetic background and adaptive evolution of the Mikado pheasant remain unclear.

Results: We present the draft genome of the Mikado pheasant, which consists of 1.04 Gb of DNA and 15 972 annotated protein-coding genes. The Mikado pheasant displays expansion and positive selection of genes related to features that contribute to its adaptive evolution, such as energy metabolism, oxygen transport, hemoglobin binding, radiation response, immune response, and DNA repair. To investigate the molecular evolution of the major histocompatibility complex (MHC) across several avian species, 39 putative genes spanning 227 kb on a contiguous region were annotated and manually curated. The MHC loci of the pheasant revealed a high level of synteny, several rapidly evolving genes, and inverse regions compared to the same loci in the chicken. The complete mitochondrial genome was also sequenced, assembled, and compared against 4 other long-tailed pheasants. The results from molecular clock analysis suggest that ancestors of the Mikado pheasant migrated from the north to Taiwan about 3.47 million years ago.

Conclusions: This study provides a valuable genomic resource for the Mikado pheasant, insights into its adaptation to high altitude, and the evolutionary history of the genus *Syrmaticus*, which could potentially be useful for future studies investigating molecular evolution, genomics, ecology, and immunogenetics.

Keywords: Mikado pheasant, *Syrmaticus mikado*, long-tailed pheasant, whole-genome sequencing, *de novo* genome assembly, adaptive evolution

Background

The Mikado pheasant (*Syrmaticus mikado*), which is a long-tailed pheasant indigenous to Taiwan, belongs to the family *Phasianidae* in the order Galliformes (Additional file 1: Fig. S1A, B). The Mikado pheasant is known to inhabit a variety of habitats in the mountainous regions of Central and Southern Taiwan at very high elevations ranging from 1600 to 3500 meters [1, 2]. The Mikado pheasant faced endangerment due to hunting pressure and habitat destruction [3, 4] until it became protected under the Wildlife Conservation Act. Currently, the International Union for Conservation of Nature (IUCN) Red List has classified the Mikado pheasant as a nearly threatened species, showing a decreasing trend in the overall population with a total estimate of approximately 15 000 mature birds. The rare and precious Mikado pheasant is a national icon in Taiwan and is depicted on its 1000 dollar banknote.

The *de novo* genome assembly of endangered species is an effective approach to identify genomic signatures associated with environmental adaptation and behavioral attributes [5, 6]. Genome resources can also provide great insights into effective population size, genetic defects, and deleterious mutations [7, 8]. Moreover, reconstruction of a phylogenetic tree can reveal genetic relationships and evolutionary history [9-11]. Together they can lead to the conservation and rescue of endangered species.

The Mikado pheasant possesses ideal characteristics for evolutionary research because of its flightlessness and habitat isolation. It is one of 5 long-tailed pheasants in the *Syrmaticus* genus, which forms a monophyletic group [12]. Due to limited molecular data, very few studies have been conducted to investigate the phylogenetic

relationships and divergence time of species within the genus. Moreover, the Mikado pheasant is mainly found in Yushan National Park [13], which has numerous extremely high mountains exceeding an altitude of 3000 meters (Additional file 1: Fig. S2). As high altitudes are associated with extremely cold climates and lower concentrations of oxygen, hypoxic stress may be observed in the pheasant. Considering its importance as a species facing endangerment, the present unavailability of genetic information regarding the Mikado pheasant motivated the *de novo* assembly of its genome, followed by a detailed study of its genetic background and subsequent adaptive evolution.

Here we report the whole-genome assembly of the Mikado pheasant and provide insights into its adaptive mechanisms. This genome-wide study reveals the evolutionary adaptation of the Mikado pheasant to high altitudes, including changes in gene family size and/or molecular signatures of positive selection associated with energy metabolism, oxygen transport, hemoglobin binding, radiation response, immune response, and DNA repair. The estimated time of divergence among the 5 long-tailed pheasant species reconstructs the evolutionary history of the lineage and allows us to propose a hypothesis for the biogeographical speciation of the Mikado pheasant. Additionally, the manually curated major histocompatibility complex (MHC) gene loci of the Mikado pheasant display evidence for molecular evolution with a high level of synteny, mainly across inverse regions in gene blocks, and several rapidly evolving genes in comparison with the chicken.

Data Description

The details about sample collection, library construction, sequencing, assembly, gene prediction, and annotation can be found in the “Materials and Methods” section.

Results

Assessment of the assembly quality

The overall DNA mapping rate of the paired-end libraries was >90% for the concordant paired read alignment and >96% for both paired and single read alignment (Additional file 1: Table S4). Thus, the assembly utilized most of the DNA reads. We further examined the per-base alignment coverage. The results (Additional file 1: Fig. S6) showed that most of the genome positions had a coverage between approximately 57- and 121-fold and an average sequence coverage of 88-fold, which is very similar to the sequencing depth of DNA paired-end libraries (98.7x). Thus, our draft genome is well assembled.

To evaluate the quality of the assembled genome [20], the RNA reads were mapped onto the draft genome. The overall alignment rate of both RNA libraries showed that approximately >93% of the reads could be aligned to the scaffolds, indicating that most of the expressed protein-coding genes could be found in the draft genome (Additional file 1: Table S5). Moreover, the BUSCO (BUSCO, RRID:SCR_015008) [21] benchmark was used to evaluate the genes predicted from the genome assembly (Additional file 1: Table S6). Of the 3023 single-copy orthologs in the vertebrate lineage, approximately 88.6% of the orthologs were found in our assembly, which is similar to the results obtained in duck (88.6%), turkey (87.5%), and zebra finch (88.8%). These results suggested that a potentially large number of genes, along with their complete structure, could be predicted from the genome.

Genome comparison

To understand the similarities between the Mikado pheasant and the chicken at the genomic level, assembled scaffolds that were longer than 0.25% of the aligned chicken chromosome were selected and plotted onto a syntenic map with an alignment length of at least 3 kb using MUMmer [22]. The identities of each chicken chromosome with the scaffolds of Mikado pheasant were between 86.24% and 89.98%, and the overall coverage was 85.28% (i.e., 855.35 Mb of the assembled scaffolds could be mapped onto the chicken genome; Additional file 1: Table S7). The syntenic relationships between the Mikado pheasant scaffolds and the chicken chromosomes were highly conserved, but a few of the chromosomes could be only partially aligned. In particular, 3 well-assembled scaffolds, i.e., scaffold14, scaffold69, and scaffold46, were mapped to nearly the full length of chicken chromosomes 15, 23, and 24, respectively. Notably, compared to the scaffolds of the Mikado pheasant, the chicken chromosomes, including chromosomes 6, 11, 18, and 21, were properly aligned, but with obvious inversions (Fig. 1A). More stringent conditions were then considered to evaluate the alignment of certain scaffolds to multiple chromosomes (e.g., scaffold1 and scaffold45; Fig. 1B); however, further confirmation is required to determine whether this finding represents the actual presence of chromosomal translocations in the Mikado pheasant genome. Additionally, the alignment between the Mikado pheasant scaffolds and the turkey chromosomes provided similar results (Additional file 1: Fig. S7A), but the Mikado pheasant scaffolds were poorly aligned with the zebra finch chromosomes (Additional file 1: Fig. S7B). In general, the Mikado scaffolds displayed high conservation with the genomes of chicken and turkey. We also observed several intrachromosomal inversions and chromosomal translocations. This is the first genome-wide analysis to identify multiple intrachromosomal inversions between the Mikado pheasant and chicken genomes.

Phylogenetic relationships of the Mikado pheasant

To compare the protein sequences of the Mikado pheasant against homologous protein families of other birds and organisms, OrthoMCL (OrthoMCL DB: Ortholog Groups of Protein Sequences, RRID:SCR_007839) [23] was used to define the gene families in 10 species. Proteins with sequences that were similar to those of the Mikado pheasant—5 birds (i.e., chicken, duck, flycatcher, turkey, and zebra finch), 2 reptiles (anole lizard and Chinese softshell turtle), and 2 mammals (human and mouse)—were classified into each gene family. In this way, we obtained 18 220 gene families in total from 10 species. Next, 5287 single-copy orthologs that were common across these species were used to construct a Bayesian maximum clade credibility phylogenetic tree and to estimate the time of divergence [24] (Fig. 2). The estimated time of the Mikado pheasant-turkey divergence was 21.4 million years ago (Mya); the divergence time between chicken and the sister clade of the Mikado pheasant-turkey was estimated at 28.9 Mya. In the Galliformes order, the Mikado pheasant was found to be more closely related to the turkey than to the chicken. The branches of the Galliformes and duck (76.4 Mya), Passeriformes and Galliformes (105.3 Mya), and anole lizard and Aves (266.3 Mya) displayed divergence times that were similar to those reported in the literature [25-27].

Gene family evolution

To assess the changes in the gene family sizes, a likelihood model was used to examine significant expansions and contractions of gene families, particularly in the Mikado pheasant lineage. Expansions or contractions in gene families indicate that total number of genes in a gene family are increasing or decreasing, respectively. The results revealed 311 expanded and 15 contracted gene families compared with the

common ancestor of the Mikado pheasant and turkey (Fig. 2). In total, 86 gene ontology (GO) categories were significantly enriched (p < 0.05, empirical test) among the 311 expanded genes. Fifty of these GO categories were further classified into 8 main categories, including actin cytoskeleton, morphogenesis, catalytic activity, cell differentiation, binding, metabolism, cytoplasm, and organelle organization and biogenesis (Additional file 2: Table S8). In particular, the gene families involved in oxygen and heme binding (GO:0019825 and GO:0020037, respectively), monooxygenase activity (GO:0004497), and energy metabolism (GO:0046034, ATP metabolic process; GO:0005977, glycogen metabolic process) were substantially expanded in the Mikado pheasant. Conversely, 7 of the 25 GO categories in the contracted gene families were significantly enriched in immune system processes and apoptosis (Additional file 1: Table S9). From the Pfam database [28], 8 of the 75 expanded gene families were annotated as olfactory receptors (Additional file 2: Table S10).

Positive selection

To detect the genes that evolved rapidly due to positive selection under the influence of high elevation (Mikado pheasant) as opposed to low elevation (chicken, turkey, duck, and zebra finch), 7132 single-copy orthologs were analyzed from 9038 genes common across the five species (Additional file 1: Fig. S8). According to the branch-site model and the likelihood ratio test, the 889 positively selected genes (PSGs) identified in the Mikado pheasant were mainly enriched in functions such as metabolism (GO:0008152), cell (GO:0005623), and binding (GO:0005488) that belong to biological process, cellular component, and molecular function ontology terms, respectively. We further examined the PSGs involved in metabolism, which constituted the largest number of PSGs and GO functions (Additional file 1: Fig. S9).

The 45 PSGs enriched in metabolism-related functions (*p*-values < 0.05) were classified according to the GOSlim categories into lipid metabolism (GO:0006629), carbohydrate metabolic processes (GO:0005975), and generation of precursor metabolites and energy (GO:0006091), which included 13, 3, and 2 GO functions, respectively (Additional file 2: Table S11). Of these metabolism-related PSGs, 4 genes were found to be involved in the inositol phosphate metabolism (map00562; *p*-value < 0.01) and phosphatidylinositol signaling system (map04070; *p*-value < 0.05) through a functional enrichment analysis from the Kyoto Encyclopedia of Genes and Genomes (KEGG (KEGG, RRID:SCR_012773)) database (Additional file 1: Table S12).

In addition to metabolism, other high-altitude adaptations were observed, such as response to radiation (GO:0010212, response to ionizing radiation; GO:0010332, response to gamma radiation; GO:0034644, cellular response to UV; and GO:0071480, cellular response to gamma radiation), DNA repair (GO:0000731, DNA synthesis involved in DNA repair; GO:0045739, positive regulation of DNA repair; and GO:0006284, base-excision repair), and oxygen transport (GO:0016706, oxidoreductase activity; GO:0072593, reactive oxygen species metabolic process; GO:0019825, oxygen binding; and GO:2000377, regulation of reactive oxygen species metabolic process; Additional file 2: Table S13). Moreover, 43 PSGs in the Mikado pheasant were significantly enriched in the categories of lymphocyte activation (GO:0046649; including 8 GO terms) and cytokine production (GO:0001816; including 8 GO terms) (Additional file 2: Table S14). We also identified the janus kinase/signal transducer and activator of transcription (Jak-STAT) signaling pathway (map04630; *p*-value < 0.05), which was enriched in 5 PSGs (i.e., *BCL2*, *CCND3*, *IL12RB2*, *IL23R*, and *IL7*), in the KEGG analysis (Additional file 1: Table S15).

Identification of the MHC-B region of the Mikado pheasant

The MHC is a cluster of genes that is associated with functions such as infectious disease resistance and immune responses in all jawed vertebrates [29]. The MHC B-locus (MHC-B) performs the main MHC functions in the chicken [30, 31]. Based on the above analysis, an assembled scaffold (scaffold208) was almost able to cover the known chicken sequence of the MHC-B contiguous region published by Shiina *et al.* (GenBank Accession: AB268588.1) [32] (Fig. 3). To understand the evolution of the MHC-B genes between the Mikado pheasant and the chicken, the predicted gene loci were manually curated by incorporating evidence from the aligned RNA-Seq data and homologous genes from chicken and turkey using Web Apollo software [33]. After the curation, 39 putative MHC genes of the Mikado pheasant were identified within a 227 kb sequence (Table 2), including 7 MHC class II loci (*BLB1*, *TAPBP*, *BLB2*, *BRD2*, *DMA*, *DMB1*, and *DMB2*), 4 MHC class I loci (*BF1*, *TAP1*, *TAP2*, and *BF2*), and 5 MHC class III loci (*C4*, *CenpA*, *CYP21*, *TNXB*, and *LTB4R1*).

Gene loci involved in immunity have been shown to have a higher ratio of nonsynonymous (d_N) to synonymous (d_S) amino acid substitutions due to interactions with rapidly evolving pathogens under selective pressures [34-36]. *KIFC1*, *BTN1*, *Blec2*, *BLB1*, *BLB2*, and *BF2* had comparatively high d_N/d_S ratios between the Mikado pheasant and the chicken (Table 2). Conversely, the genes with comparatively lower d_N/d_S ratios included *TRIM7.2*, *TRIM41*, *BRD2*, and *CenpA*. As shown in Fig. 3, the Mikado pheasant and the chicken displayed similarity in the MHC-B region and shared an almost perfect syntenic gene order. Notably, no *BLB2* genes were predicted between the *TAPBP* and *BRD2* intergenic regions in the Mikado pheasant MHC-B locus; however, these regions could be detected among the transcripts of our RNA-Seq data. A likely explanation for the absence of a prediction

of the BLB2-like gene might be the existence of 2 unsequenced gap regions with a size of 1098 bp within the *TAPBP-BRD2* block (5931 bp). Since *BLB2* is only 792 bp in length, it could reside within the missing sequence. Based on the RNA-Seq results, 2.54 million reads were mapped onto 38 MHC-B genes (except for *BLB2*) of the Mikado pheasant, 27 of which had at least a 1-fold average coverage per nucleotide. Furthermore, 15 genes possessed more than 100-fold average coverage per nucleotide, providing concrete evidence of a reliable prediction. Intriguingly, 2 gene loci, i.e., *TAPBP* and the *TAP1-TAP2* block, were inversely oriented compared to the chicken sequence.

Evolutionary history of *Syrmaticus* pheasants

The mitochondrial genome of the Mikado pheasant was assembled based on the short-read libraries. The circular complete genome had a total length of 16 680 bp, including 13 protein-coding genes, 2 rRNAs, 22 tRNAs, and a control region (Additional file 1: Table S16). The average nucleotide composition was 30.52% A, 31.20% C, 13.44% G, and 24.84% T. To investigate the evolutionary history of the genus *Syrmaticus*, which includes 5 long-tailed pheasants, the phylogeny was reconstructed, and the divergence times were estimated using the mitochondrial genomes. According to molecular clock analysis, the genetic divergence of the Mikado pheasant began approximately 3.47 (2.78-4.71) Mya (Fig. 4). The tree topology is consistent with previous studies [12, 37], and the divergence time suggests that the Mikado pheasant might have originated in the late Pliocene.

Amino acid substitution analysis in Mikado pheasant hemoglobin genes

Living at high elevations directly incurs the challenge of low oxygen availability. Additionally, exposure to low-pressure environments causes oxygen saturation in the

arterial blood, thus decreasing and restricting oxygen supplementation to tissues [38]. Certain birds show an increased combined affinity between blood and oxygen via amino acid substitutions in the major hemoglobin [39-41]. To investigate their role in adaptation to high-altitude environments, amino acid substitutions were examined in the Mikado pheasant hemoglobin sequences. By comparing 6 avian species, an amino acid substitution with different consensus residues was found in the Mikado pheasant (Additional file 1: Fig. S10), and the substitution of alanine with threonine occurred at residue 78 of the alpha-A subunit—the major component of hemoglobin isoforms. The Andean goose, a kind of waterfowl living at over 3000 meters in the Andes, has been reported to carry the identical substitution [42].

Genome assembly and annotation

In total, 171.7 Gb of raw DNA sequence reads (Additional file 1: Table S1) were generated, resulting in an approximately 160-fold sequencing coverage based on the 1.07 Gb genome size estimated by KmerGenie [14]. The contigs were built and assembled into a 1.04 Gb sequence of the draft genome. The N50 lengths of the contigs and scaffolds were 13.46 kb and 11.46 Mb, respectively. The overall GC content of the Mikado pheasant genome was 41.13%, which is similar to that of the chicken, duck, turkey, and zebra finch (Additional file 1: Fig. S3). The size of the longest assembled sequence was 50.28 Mb, and 928 scaffolds were longer than 10 kb. The basic statistics of both the contigs and scaffolds assembled using MaSuRCA [15] are shown in Table 1. The cumulative length plots (Additional file 1: Fig. S4A, B) and the Nx plot for the scaffolds (Additional file 1: Fig. S5) showed that most of the draft genome consisted of large scaffolds; though many short scaffolds were present, they only contributed a small portion of the genome size.

Before performing the gene prediction and annotation, the interspersed and low complexity regions were first masked using RepeatMasker (RepeatMasker, RRID:SCR_012954) [16]. Approximately 8.91% of the sequences were identified as interspersed repeats, 1.32% of the sequences were identified as long tandem repeat (LTR) elements, and overall 11.46% of the total bases were identified (Additional file 1: Table S2). After masking the repeats and extrinsic data, an *ab initio* gene prediction was performed using Augustus (Augustus: Gene Prediction, RRID:SCR_008417) [17], followed by EVidenceModeler [18]. The final gene models comprised 27 254 transcripts (proteins). Of the predicted proteins, 15 972 (58.6%) could be strictly aligned to the National Center for Biotechnology Information (NCBI) non-redundant (NR) protein database for Aves and Reptilians. The statistics of annotated genes in the Mikado pheasant averaged 19.9 kb per gene, 1625 bp per coding DNA sequence (CDS), 164.1 bp per exon, and 2053 bp per intron (Additional file 1: Table S3), which are similar composition in length to other avian species [19]. Out of the 15 972 NR annotated proteins, 14 124 proteins were well annotated to the Pfam domains. A total of 5626 Pfam domains were identified based on our predictions.

Discussion

In this study, experimental data and statistical approaches were used to evaluate the genome assembly of the Mikado pheasant. Notably, the genome sequence of this species was previously unknown, and this study provides a comparative analysis of various genomes using a large number of tools at different stages for the assembly of the Mikado pheasant genome. While conducting the genome assembly, we used not only MaSuRCA but also assembly tools, such as ALLPATHS-LG [43], JR [44], Newbler [45], SGA [46], and SOAPdenovo [47]. All these assembly tools produced

similar draft genome sizes, and MaSuRCA and SGA also showed similar results in terms of the N50 value and the scaffold number (Additional file 1: Table S17). To facilitate the downstream analysis, we used several methods to compare these assembly sets. However, no single assembly tool outperformed the others in terms of the number of annotations for the predicted genes, the quality of the genome compared to that of other birds, and the BUSCO benchmark. In this study, the draft genome assembled using MaSuRCA was selected because it generated dramatically longer scaffolds that displayed a decent score on the BUSCO benchmark and produced proper annotations for the predicted genes. Although scaffolds of the draft genome displayed some degree of fragmentation (Fig. 1A) and showed translocation (Fig. 1B) in certain chicken chromosomes, our approach still provides a practical strategy for whole-genome assembly using only short-read sequencing technology. We assert that the high coverage of our sequencing data, differing library insert sizes, and the use of a combination of tools, such as MaSuRCA and SSPACE for assembly and scaffolding, respectively, contributed to high-quality *de novo* assembly of the Mikado pheasant genome with a genome length of approximately 1 Gb.

Recent studies have reported phylogenetic tree topologies for the Mikado pheasant and other Galliformes birds [37, 48, 49]; however, these studies relied on small amounts of genomic DNA as supporting evidence. To obtain a highly accurate phylogenetic inference, long DNA sequences are necessary for the reconstruction of a high-resolution tree [50-52]. This study used whole-exome information, with 5287 single-copy orthologs totaling approximately 8 Mb of coding sequence, to reconstruct the phylogeny and estimate the divergence time among the Mikado pheasant and other birds (Fig. 2). Our results strongly suggest that the Mikado pheasant is more similar to the turkey than the chicken in the Galliformes clade, which is consistent with previous studies [37, 48, 49].

We additionally implemented a comprehensive phylogenetic analysis strategy to obtain information regarding the adaptive mechanisms of the Mikado pheasant to high elevations. Compared to birds living at low altitudes, both the positive gene selection and gene expansion analyses showed a significant enrichment of genes relevant to energy metabolism (Additional file 2: Tables S8 and S11). This finding was consistent with the prior study that identified similar genes in other species inhabiting the highlands [53]. Moreover, the 4 metabolism-related PSGs (i.e., *INPP5A*, *INPP5J*, *PI4KB*, and *PLCE1*) that were involved in the inositol phosphate metabolism and phosphatidylinositol signaling system (Additional file 1: Table S12) were previously reported to be enriched in Tibetan pigs living at high altitudes [54]. Of these genes, *INPP5A* and *INPP5J* play a role in the hydrolysis of inositol polyphosphates [55], *PI4KB* is a phosphatidylinositol kinase that induces phosphorylation reactions [56], and *PLCE1*, which is a phospholipase enzyme, regulates gene expression, cell growth, and differentiation [57]. Another robust signal of its adaptation to high altitude was obtained from genes significantly associated with expansion of and positive selection for the enhancement of hemoglobin binding and oxygen transport (Additional file 2: Tables S8 and S13). Furthermore, for both the Mikado pheasant and Andean goose, an amino acid substitution was identified in the hemoglobin alpha-A subunit (Additional file 1: Fig. S10). The substitution of threonine at this position has recently been shown to cause an increase in the molecular volume, which might enhance the solubility of hemoglobin and facilitate adaptation to desiccating and high-altitude environments [42]. Through gene expansion, the genes of the Mikado pheasant that are involved in skeletal and cardiac muscle fiber development (Additional file 2: Table S8) and the enhanced functions of the additional GO terms implied that the biomass of the Mikado pheasant could be effectively produced in mountainous regions without nourishment, hence strongly suggesting the existence of an adaptive

mechanism for high altitudes [58]. Finally, the PSGs in the radiation response, immune response, and DNA repair categories (Additional file 2: Tables S13 and S14) may reflect the increased resistance of the Mikado pheasant to long-term ultraviolet radiation exposure through the induction of cytokine production [59] and lymphocyte activation [60] and DNA repair processes. Some of these PSGs were also involved in the Jak-STAT signaling pathway (Additional file 1: Table S15), which participates in chemical signal transmission and induces cellular stress responses, such as immunity, apoptosis, [61, 62], and hypoxia [63]. All these results provide wider support for the adaptive evolution of the Mikado pheasant. To sum up, this study reveals the high-altitude adaptation mechanisms of the Mikado pheasant at the genomic level. However, there are some adaptive mechanisms for high altitude that happen via changes in regulatory regions modulating the levels of gene expression [64-66]. We believe that this is an intriguing topic and worthy of further research to be undertaken in the future.

In this work, we annotated and curated the MHC-B gene loci in the Mikado pheasant, which is important for assessing the adaptive mechanisms associated with endangered species, because variations in gene number in the MHC cluster could be caused by exposure to pathogens or diseases [67, 68]. The genome of the Mikado pheasant contains a number of MHC-B genes, and inversions were observed in the *TAPBP* locus and the *TAP1-TAP2* block (Fig. 3) compared to the chicken genome; an inverse orientation of the *TAP1-TAP2* block was also detected compared to the turkey genome (Additional file 1: Fig. S11). A similar conversion at the MHC locus in Galliformes has been reported in previous studies [29, 34, 69]. We further observed a Blec2-like sequence with an inverse orientation located within the *BG1-Blec2* region in the Mikado pheasant. We inferred that this region is likely similar to the *Blec4* pseudogene of the chicken and highly similar to *Blec2* [32].

In this study, we not only sequenced the whole genome of a bird of the *Syrmaticus* genus but also completed the full mitochondrial genome. Before whole-genome sequences were available, mitochondrial sequences were widely utilized in molecular phylogenetic analyses of the genus of *Gallus* [70, 71]. Based on the assembly of the Mikado pheasant and the other 4 available sequences, we reconstructed a phylogenetic tree and provide a completely sequenced mitochondrial genome for 5 long-tailed pheasants. The topology of our reconstructed tree (Fig. 4) is consistent with results from a previous study [12]. However, the time of divergence was estimated to be earlier than the previously reported time [12] for the Mikado pheasant, which might have been due to the use of a few mitochondrial or nuclear genes rather than the complete mitochondrial genome. The reconstructed tree showed a potential migration pathway of these pheasants. The ancestors of the Mikado pheasant, which have been described to have migrated to the island of Taiwan, separated from the lineage of the copper pheasant (*S. soemmerringii ijimae*). The copper pheasant is a pheasant indigenous to Japan, whose ancestors might have separated from the lineage of the Reeves's pheasant (*S. reevesii*) that has inhabited in Northern China. The ancestors of Elliot's pheasant (*S. ellioti*) and Mrs. Hume's pheasant (*S. humiae*) have branched from the Mikado pheasant, then separated into two present kinds of pheasants that have alternatively roosted in the mountainous forests of Southeastern and Southwestern China, respectively. According to paleogeographical reports, Taiwan was formed approximately 4-5 Mya and attained its modern topography approximately 3 Mya [72]. The sea level was lower during the glacial periods, and Taiwan might have been connected to the mainland [73]. Our results suggest that the evolutionary history of the Mikado pheasant might have included ancestors that migrated from the north towards Taiwan approximately 3.47

Mya and consequently were isolated by the Taiwan Strait during the warm interglacial periods during the early Pleistocene.

Currently, there is no nuclear genome data available for the copper pheasant, so unfortunately, incorporating all five long-tailed pheasants into our analysis using nuclear genomes is impossible at present. For the other four pheasants, however, Wang N. *et al.* [37] used six nuclear intron and two mitochondrial gene sequences to construct a phylogenetic tree, and its topology was consistent with our result. Our estimate of the divergence time was more precise, considering that we employed complete mitochondrial genomes in the reconstruction of a high-resolution tree for the *Syrmaticus* genus instead of a few mitochondrial genes. Our estimated divergence time is also supported by the paleogeographical report of Taiwan island formation. Despite these corroborations of the proposed tree topology and estimated divergence time, the use of only mitochondrial data may be considered as a potential limitation. Going forward, it will be necessary to analyze the nuclear genome to obtain further insights into the evolution history of long-tailed pheasants.

Materials and Methods

Sample preparation and sequencing

Blood samples were collected from a single female Mikado pheasant living in Central Taiwan; then, genomic DNA was extracted, and 2 paired-end libraries (280 bp and 480 bp; average read length: 151 bp) and 5 mate pair libraries (1, 3, 5, 7, and 10 kb; average read length: 101 bp) were constructed according to the manufacturer's protocol. In addition, 2 RNA-Seq libraries from 2 male Mikado pheasants' blood samples were prepared for the purpose of draft genome assessment and gene prediction (Additional file 1: Table S1). The DNA libraries were sequenced using the

HiSeq platform (Illumina Inc., San Diego, CA, USA), and the RNA libraries were sequenced using the HiScanSQ and HiSeq platforms.

De novo genome assembly

The quality of the raw reads was examined using FastQC (FastQC, RRID:SCR_014583), version 0.10.1. Trimmomatic (Trimmomatic, RRID:SCR_011848), version 0.30 (parameters: "ILLUMINACLIP:TruSeq3-PE.fa:2:30:15 SLIDINGWINDOW:4:20 MINLEN:100") [76] and NextClip (version 1.3.1) [77] with default parameters were used to trim sequencing reads. Genome assembly into contigs was performed by MaSuRCA (version 2.3.2) [15] with settings based on the instruction manual. ALLPATHS-LG (ALLPATHS-LG, RRID:SCR_010742, version 49722) [43], Newbler (version 2.9) [45] both with default parameters, JR (version 1.0.4; parameters: "-minOverlap 60 -maxOverlap 90 -ratio 0.3") [44], SGA (version 0.10.13; parameters: "assemble -m 125 -d 0.4 -g 0.1 -r 10 -l 200") [46], and SOAPdenovo (version 2.04; parameters: "-K 47 -R") [47] were also used to assemble contigs. We employed SSPACE (SSPACE, RRID:SCR_005056, version 3.0; parameter: "-z 300") [74] to construct scaffolds for the draft genome. In this step, mate pair libraries with 35 bases from the 5'end of both reads were used for scaffolding. Scaffold sequences shorter than 300 bp were then excluded from the final assembly. The statistical results of the assembly were estimated using QUAST (version 3.2) [75].

To examine sequencing reads for potential contamination, we used Kraken (version 1.0) [78] with the standard Kraken database to check the paired-end DNA libraries. Classified reads reported by Kraken were further examined using our proposed pipeline (Additional file 1: Fig. S12). Briefly, we employed Bowtie 2 (Bowtie, RRID:SCR_005476; version 2.3.0) [79] to align these classified reads

against the chicken genome reference (Galgal 5.0) downloaded from Ensembl (release 90), collecting unmapped reads and using Bowtie 2 again to align them against the assembled genome of the Mikado pheasant. We then took those reads mapped onto the Mikado pheasant genome and performed BLASTN alignment against the non-redundant nucleotide sequences (NT) database, downloaded from NCBI's FTP site (on Nov. 16, 2017), using parameters "-outfmt '6 std staxids' -max_target_seqs 1 -evalue 1E-10." Next, we collected reads with alignment length ≥100 bp (i.e., two thirds of read length), filtering out the reads matching an avian species or with a read count <50 in a species. The remaining reads were counted and the contaminated scaffolds calculated by applying a cutoff of a read count >20 on a given scaffold. Finally, we removed 31 contaminated scaffolds with 12 587 bp (~0.001% of the total length) from the assembled genome.

Evaluation of assembly quality

Several metrics were used to evaluate the assembly quality, including the number and length distribution of the scaffold sequences, the mapping rate of the paired-end DNA reads and RNA reads, the per-base coverage of the DNA read mapping, and the coverage of universal single-copy orthologs provided by BUSCO (version 1.21). To evaluate the mapping rate of the reads and per-base coverage, the paired-end DNA reads and RNA reads were aligned against the assembled scaffolds using Bowtie 2 (version 2.2.4) and STAR [81], respectively. Briefly, scaffolds were mainly assembled from the paired-end DNA reads, and the higher mapping rate of the paired-end DNA reads suggests a higher degree of the final assembly covering the raw reads. Taking the RNA sequencing reads from two individual Mikado pheasants and observing the mapping rate is another approach for assessing the completeness of the assembly. The per-base DNA read coverage was calculated using BEDTools

(BEDTools, RRID:SCR_006646), version 2.23.0 [82]. For each base, the expected coverage should be close to the sequencing depth of the paired-end reads (approximately 98.7x). The BUSCO benchmark is a single-copy ortholog set derived from the species of a major lineage. The gene models predicted from the draft genome in the Mikado pheasant were compared with the lineage of vertebrates (3023 single-copy orthologs in total) provided by BUSCO. Protein sequences from the chicken, duck, turkey, and zebra finch were also evaluated for comparison.

Genome comparison

To compare the genome of the Mikado pheasant with that of other avian species, we retrieved the whole-genome sequences of the chicken (Galgal4), turkey (UMD2) and zebra finch (taeGut3.2.4) from the Ensembl database. Using the genome-wide sequence aligner MUMmer (version 3.23), the chromosome-level differences and similarities among the species were investigated and visualized. The structural variants among the species were further reported using the "show-diff" utility in MUMmer. The chord diagrams of the alignment were generated using Circos [83].

Gene prediction and annotation

First, RepeatMasker (version 4.0.5; parameter: "-species chicken"), including rmblastn (version 2.2.23+) as the search engine, RepBase (version 20140131), and RM database (version 20140131), were applied to screen the scaffolds for interspersed repeats and low-complexity regions in the DNA sequences, and the masked genome was used for further gene prediction. Then, homology-based, RNA-Seq, and *ab initio* prediction approaches were used to identify protein-coding genes and build a consensus gene set that included all predicted genes. For the homology protein sequence alignment, the protein sequences of the chicken (Galgal4),

turkey (UMD2), duck (BGI_duck_1.0) and zebra finch (taeGut3.2.4) were collected from Ensembl. The protein sequence alignments were performed using Exonerate (version 2.2.0) [84]. All RNA-Seq reads were aligned against the repeat-masked genome using TopHat2 [80], which generated evidence of splice sites, introns, and exons. Additionally, Trinity (Trinity, RRID:SCR_013048), version 2.0.6, [85] was utilized to assemble transcripts, and PASA (version 2.0.0) [86] was used to group alternatively spliced isoforms. For the *ab initio* gene prediction, the standard Augustus (version 3.0.3) pipeline was used to yield potentially predicted genes with evidence from both homologous proteins and RNA-Seq. Next, the consensus gene set was determined by consolidating the 3 types of gene prediction using EVidenceModeler (version 1.1.1). Finally, the gene annotations were defined based on the best sequence alignment against NCBI NR proteins in Aves and Reptilians using BLASTP (version 2.2.29+), with the following criteria: identity ≥ 30%, alignment length ≥ 80 bp, and E-value ≤ 1e–5. For the protein domain identification, we annotated the domains using HMMER (version 3.1b2) [87] by scanning the Pfam database (version 30.0).

For MHC-B annotation and curation, we first took the scaffold208 sequence and used MAKER (version 2.31.8) [88] to predict the potential gene structures of MHC-B genes. Next, the RNA-Seq libraries from the Mikado pheasant and the homologous protein sequences from chicken and turkey were aligned to these predicted regions. Finally, we used Web Apollo (version 2.0.3), a web-based and visualization tool for curation and annotation, to manually curate these genes according to the alignment evidence.

Gene families

To identify gene families, the protein-coding genes of 5 birds (i.e., *Gallus gallus, Meleagris gallopavo, Anas platyrhynchos, Taeniopygia guttata*, and *Ficedula albicollis*) and 4 additional species (*Anolis carolinensis, Pelodiscus sinensis, Homo sapiens,* and *Mus musculus*) were downloaded from Ensembl (release 82). The sequence of the longest isoform was selected to represent the gene for each species, despite the presence of protein isoforms. The all-vs-all BLASTP was applied to align all protein sequences (including those of the Mikado pheasant) of the 10 species and 5 birds (excluding flycatcher) with E-value thresholds less than 1e–5 and 1e–20, respectively. Then, 18 220 gene families (including 5287 single-copy orthologs) were obtained from the 10 species, and 13 436 gene families (including 7132 single-copy orthologs) were obtained from the 5 birds by OrthoMCL (version 2.0.9) using default parameters. In the analysis of the 10 different species, 15 161 genes of the Mikado pheasant were grouped into 12 549 gene families. In the analysis of the 5 avian species, 14 375 Mikado pheasant genes were grouped into 12 078 gene families. Next, MUSCLE (MUSCLE, RRID:SCR_011812), version 3.8.1551, [89] was used with default parameters for the multiple sequence alignment of the converted coding DNA sequences from single-copy orthologs, and Gblocks (version 0.91b; parameters: "-t=d -b4=5 -b5=h -e=_cln") [90] was used to remove the poorly aligned regions. After trimming, the genes from each species were concatenated using the same order to reconstruct the phylogenies and evaluate the divergence time. The concatenated sequences were used to build a phylogenetic tree using RAxML (RAxML, RRID:SCR_006086), version 8.2.4, [91] via a maximum likelihood search with 500 bootstrap replicates; then, the divergence time was analyzed using BEAST (BEAST, RRID:SCR_010228), version 2.3.2, with the GTR+I+Γ model, which is the best substitution model selected by Modeltest (version 3.7) and PAUP* (version 4.0a150)

[92]. Four nodes were chosen as the fossil calibration points from the TimeTree database [93], including human-chicken (311.9 Mya), anole lizard-chicken (279.7 Mya), Chinese softshell turtle-chicken (253.7 Mya), and human-mouse (89.8 Mya). The phylogenetic tree was generated using the Strap R package [94]. To identify the gene families with a expansion or contraction between the Mikado pheasant and other species, CAFE (version 3.1) [95] was used to estimate the rates of gene family evolution from the observed gene numbers in each family and the given phylogenetic tree. A p-value < 0.05 was used to indicate significant changes in the gene family size.

Examination of genes under positive selection and enrichment analysis

To determine the genes that underwent positive natural selection in the Mikado pheasant, CODEML from PAML (PAML, RRID:SCR_014932), version 4.8, [96] was applied to the branch-site model to investigate the genes in positively selected sites of the Mikado pheasant. For the branch-site model, we implemented likelihood ratio tests to determine the statistical significance of positive selection for testing a null model (model = 2, NSsites = 2, fix_omega = 1, and omega = 1) against an alternative model (model = 2, NSsites = 2, and fix_omega = 0). Consequently, the false discovery rates (FDRs) were computed with a cutoff of 0.05 to adjust for multiple testing using the Benjamini-Hochberg procedure.

The GO annotations of 4 birds (i.e., chicken, duck, turkey, and zebra finch) retrieved from the Ensembl BioMart were used to characterize the functions of the identified orthologs. A hypergeometric test was performed to identify significant GO functions in these orthologs. However, the raw p-values of the hypergeometric tests can easily be affected by the number of genes [97]; therefore, to address the underlying bias of the hypergeometric distribution, we further calculated empirical

p-values [98]. The empirical *p*-values were determined through 100K simulated datasets by ranking the hypergeometric probability of enriched functional categories compared with the null baseline probabilities. The null baseline probability was established by randomly selecting a group of genes containing an equal number of PSGs with an FDR < 0.05 for the branch-site model. For massively enriched GO terms with similar functions, CateGOrizer [99] was used to classify the genes into basic categories. ClueGO [100] with the hypergeometric test and a Bonferroni adjustment were performed to enrich the KEGG pathways [101].

Mitochondrial genome assembly

Geneious (version 8.1.5) [102] was utilized with the default settings to assemble the whole mitochondrial genome. First, the reads were mapped to the 4 available *Syrmaticu*s mitochondrial genomes from GenBank (AB164622.1 - AB164625.1). The mapped reads were collected and then used for the further assembly of the mitochondrial genome of the Mikado pheasant. The genes were identified using MITOS [103] and curated by comparison with known sequences of other long-tailed pheasants from GenBank. The phylogenetic reconstruction and estimation of the divergence times among the 5 long-tailed pheasants were achieved using BEAST with the GTR+G model, which was selected as the best nucleotide substitution model by Modeltest and PAUP*. We added 2 nodes as the fossil calibration points according to the TimeTree database, including Elliot's pheasant-Reeves's pheasant (11.1 Mya) and Elliot's pheasant-Mrs. Hume's pheasant (0.2 Mya). A calibrated Yule speciation process was implemented in the analysis using BEAST. In the Markov chain Monte Carlo analysis, the chain length utilized 10 million generations.

Additional files

Additional file 1: Supplementary figures S1-S12 and supplementary tables S1-S7, S9, S12, and S15-S17.

Additional file 2: Supplementary tables S8, S10-S11, and S13-S14.

List of abbreviations

FDR: false discovery rate; GO: Gene Ontology; IUCN: International Union for Conservation of Nature; LRT: likelihood ratio test; MHC: major histocompatibility complex; Mya: million years ago; NR: non-redundant; PSG: positively selected gene.

Availability of supporting data

Data for the *Syrmaticus mikado* genome has been deposited in the GenBank/EMBL/DDBJ Bioproject database under the project number PRJNA389983. Raw genomic and transcriptomic sequence datasets were deposited in the Sequence Read Archive (SRA) under the accession number SRP10896. Other supporting data, including the draft genome, annotations, alignments, phylogenetic trees and scripts are available via the *GigaScience* repository, GigaDB [104].

Competing interests

The authors declare that they have no competing interests.

Funding

This work was supported by a grant from Taipei Zoo (No. 13, 2015 Animal Adoption Programs of Taipei Zoo). The funder had no role in the design, collection, analysis, or

interpretation of the data; writing the manuscript; or the decision to submit the manuscript for publication.

Acknowledgements

We thank Dr. Mei-Yeh Lu and the High Throughput Genomics Core at the Biodiversity Research Center, Academia Sinica, for the NGS library constructions and Illumina sequencing. We are also grateful to the Computer and Information Networking Center, National Taiwan University, and the National Center for High-performance Computing for the support of the high-performance computing facilities. We thank Dr. Chih-Ming Hung and Dr. Chen Siang Ng for discussion and comments on the phylogenetic analysis, Dr. Chia-Yang Cheng and Tai-Rong Hong for assistance in data analyses, Dr. Melissa Stauffer and Dr. Yao-Yin Chang for assistance with editing the manuscript, and Yu-Cheng Hsieh for providing photos of the Mikado pheasant.

Ethics approval and consent to participate

All experimental procedures and sample collection methods in this study involving Mikado pheasants were conducted according to the Wildlife Conservation Act (amendment on July 8, 2009, Taiwan) and were approved by the Council of Agriculture, Executive Yuan, Taipei, Taiwan with issue No. 1021700417.

Author Contributions

E.Y.C., C.-Y.C., M.-H.T., S.-T.D., and H.L. conceived the project. E.Y.C., C.-Y.C., M.-H.T., and E.-C.L. managed and coordinated the project. M.-H.T., S.-T.D., and E.-C.L. performed animal work and prepared biological samples. T.-P.L. and L.-C.L. designed bioinformatics and evolutionary analyses. C.-Y.L., P.-H.H., and K.-Y.L.

performed genome assembly. P.-H.H. performed assessment of the assembly quality. C.-Y.L. and P.-H.H. performed gene prediction and annotation. C.-Y.L. and L.-M.C. performed evolutionary analysis. C.-Y.L. performed mitochondrial genome assembly and gene annotation, and curated the MHC-B gene loci. Y.-F.L. wrote a visualization program for displaying MHC-B genes. C.-Y.L., P.-H.H., and A.C. wrote the manuscript. A.C., M.-H.T., and C.-Y.C. commented on the draft and revised the manuscript.

Mong-Hsun Tsai, Chien-Yu Chen, and Eric Y Chuang co-supervised the study.

All authors read and approved the final manuscript.

References

1. Bridgman CL. Habitat use, distribution and conservation status of the mikado pheasant (Syrmaticus mikado) in Taiwan. The University of Tennessee; 2002.
2. Severinghaus SR. A study of the Swinhoe's and Mikado pheasants in Taiwan with recommendations for their conservation. Cornell University, May; 1977.
3. McGowan PJ and Garson PJ. Pheasants: status survey and conservation action plan 1995-1999. IUCN; 1995.
4. Fuller RA. Pheasants: status survey and conservation action plan 2000-2004. IUCN; 2000.
5. Yu L, Wang GD, Ruan J, Chen YB, Yang CP, Cao X, et al. Genomic analysis of snub-nosed monkeys (Rhinopithecus) identifies genes and processes related to high-altitude adaptation. Nat Genet. 2016;48 8:947-52. doi:10.1038/ng.3615.
6. Le Duc D, Renaud G, Krishnan A, Almen MS, Huynen L, Prohaska SJ, et al. Kiwi genome provides insights into evolution of a nocturnal lifestyle. Genome biology. 2015;16:147. doi:10.1186/s13059-015-0711-4.
7. Li S, Li B, Cheng C, Xiong Z, Liu Q, Lai J, et al. Genomic signatures of near-extinction and rebirth of the crested ibis and other endangered bird species. Genome biology. 2014;15 12:557. doi:10.1186/s13059-014-0557-1.
8. Hung CM, Shaner PJ, Zink RM, Liu WC, Chu TC, Huang WS, et al. Drastic population fluctuations explain the rapid extinction of the passenger pigeon. Proceedings of the National Academy of Sciences of the United States of America. 2014;111 29:10636-41. doi:10.1073/pnas.1401526111.
9. Qiu Q, Zhang G, Ma T, Qian W, Wang J, Ye Z, et al. The yak genome and adaptation to life at high altitude. Nat Genet. 2012;44 8:946-9. doi:10.1038/ng.2343.
10. Li R, Fan W, Tian G, Zhu H, He L, Cai J, et al. The sequence and de novo assembly of the giant panda genome. Nature. 2010;463 7279:311-7. doi:10.1038/nature08696.
11. Qu Y, Zhao H, Han N, Zhou G, Song G, Gao B, et al. Ground tit genome reveals avian adaptation to living at high altitudes in the Tibetan plateau. Nat Commun. 2013;4:2071. doi:10.1038/ncomms3071.
12. Zhan XJ and Zhang ZW. Molecular phylogeny of avian genus Syrmaticus based on the mitochondrial cytochrome B gene and control region. Zoolog Sci. 2005;22 4:427-35. doi:10.2108/zsj.22.427.
13. Lee P, Lue K, Hsieh J, Lee Y, Pan Y, Chen H, et al. A wildlife distribution database in Taiwan. Council of Agriculture, Taipei. 1998.

14. Chikhi R and Medvedev P. Informed and automated k-mer size selection for genome assembly. Bioinformatics. 2014;30 1:31-7. doi:10.1093/bioinformatics/btt310.

15. Zimin AV, Marcais G, Puiu D, Roberts M, Salzberg SL and Yorke JA. The MaSuRCA genome assembler. Bioinformatics. 2013;29 21:2669-77. doi:10.1093/bioinformatics/btt476.

16. Smit AF, Hubley R and Green P. RepeatMasker Open-3.0. 1996.

17. Stanke M, Diekhans M, Baertsch R and Haussler D. Using native and syntenically mapped cDNA alignments to improve de novo gene finding. Bioinformatics. 2008;24 5:637-44.

18. Haas BJ, Salzberg SL, Zhu W, Pertea M, Allen JE, Orvis J, et al. Automated eukaryotic gene structure annotation using EVidenceModeler and the Program to Assemble Spliced Alignments. Genome biology. 2008;9 1:R7. doi:10.1186/gb-2008-9-1-r7.

19. Zhang G, Li B, Li C, Gilbert MT, Jarvis ED, Wang J, et al. Comparative genomic data of the Avian Phylogenomics Project. Gigascience. 2014;3 1:26. doi:10.1186/2047-217X-3-26.

20. Lee C-Y, Chiu Y-C, Wang L-B, Kuo Y-L, Chuang EY, Lai L-C, et al. Common applications of next-generation sequencing technologies in genomic research. Translational cancer research. 2013;2 1:33-45.

21. Simao FA, Waterhouse RM, Ioannidis P, Kriventseva EV and Zdobnov EM. BUSCO: assessing genome assembly and annotation completeness with single-copy orthologs. Bioinformatics. 2015;31 19:3210-2. doi:10.1093/bioinformatics/btv351.

22. Kurtz S, Phillippy A, Delcher AL, Smoot M, Shumway M, Antonescu C, et al. Versatile and open software for comparing large genomes. Genome biology. 2004;5 2:R12. doi:10.1186/gb-2004-5-2-r12.

23. Li L, Stoeckert CJ, Jr. and Roos DS. OrthoMCL: identification of ortholog groups for eukaryotic genomes. Genome research. 2003;13 9:2178-89. doi:10.1101/gr.1224503.

24. Drummond AJ, Suchard MA, Xie D and Rambaut A. Bayesian phylogenetics with BEAUti and the BEAST 1.7. Mol Biol Evol. 2012;29 8:1969-73. doi:10.1093/molbev/mss075.

25. Lu L, Chen Y, Wang Z, Li X, Chen W, Tao Z, et al. The goose genome sequence leads to insights into the evolution of waterfowl and susceptibility to fatty liver. Genome biology. 2015;16:89. doi:10.1186/s13059-015-0652-y.

26. Jiang L, Wang G, Peng R, Peng Q and Zou F. Phylogenetic and molecular dating analysis of Taiwan Blue Pheasant (Lophura swinhoii). Gene. 2014;539 1:21-9. doi:10.1016/j.gene.2014.01.067.

27. Cai Q, Qian X, Lang Y, Luo Y, Xu J, Pan S, et al. Genome sequence of ground tit Pseudopodoces humilis and its adaptation to high altitude. Genome biology. 2013;14 3:R29. doi:10.1186/gb-2013-14-3-r29.

28. Finn RD, Mistry J, Tate J, Coggill P, Heger A, Pollington JE, et al. The Pfam protein families database. Nucleic Acids Res. 2010;38 Database issue:D211-22. doi:10.1093/nar/gkp985.

29. Wang B, Ekblom R, Strand TM, Portela-Bens S and Hoglund J. Sequencing of the core MHC region of black grouse (Tetrao tetrix) and comparative genomics of the galliform MHC. BMC Genomics. 2012;13:553. doi:10.1186/1471-2164-13-553.

30. Kaufman J, Milne S, Gobel TW, Walker BA, Jacob JP, Auffray C, et al. The chicken B locus is a minimal essential major histocompatibility complex. Nature. 1999;401 6756:923-5. doi:10.1038/44856.

31. Kaufman J, Volk H and Wallny HJ. A "minimal essential Mhc" and an "unrecognized Mhc": two extremes in selection for polymorphism. Immunol Rev. 1995;143:63-88.

32. Shiina T, Briles WE, Goto RM, Hosomichi K, Yanagiya K, Shimizu S, et al. Extended gene map reveals tripartite motif, C-type lectin, and Ig superfamily type genes within a subregion of the chicken MHC-B affecting infectious disease. J Immunol. 2007;178 11:7162-72.

33. Lee E, Helt GA, Reese JT, Munoz-Torres MC, Childers CP, Buels RM, et al. Web Apollo: a web-based genomic annotation editing platform. Genome biology. 2013;14 8:R93. doi:10.1186/gb-2013-14-8-r93.

34. Chaves LD, Krueth SB and Reed KM. Defining the turkey MHC: sequence and genes of the B locus. J Immunol. 2009;183 10:6530-7. doi:10.4049/jimmunol.0901310.

35. Emerman M and Malik HS. Paleovirology--modern consequences of ancient viruses. PLoS Biol. 2010;8 2:e1000301. doi:10.1371/journal.pbio.1000301.

36. Sawyer SL, Wu LI, Emerman M and Malik HS. Positive selection of primate TRIM5alpha identifies a critical species-specific retroviral restriction domain. Proceedings of the National Academy of Sciences of the United States of America. 2005;102 8:2832-7. doi:10.1073/pnas.0409853102.

37. Wang N, Kimball RT, Braun EL, Liang B and Zhang Z. Assessing phylogenetic relationships among galliformes: a multigene phylogeny with

expanded taxon sampling in Phasianidae. PLoS One. 2013;8 5:e64312. doi:10.1371/journal.pone.0064312.

38. Powell FL, Shams H, Hempleman SC and Mitchell GS. Breathing in thin air: acclimatization to altitude in ducks. Respir Physiol Neurobiol. 2004;144 2-3:225-35. doi:10.1016/j.resp.2004.07.021.

39. Monge C and Leon-Velarde F. Physiological adaptation to high altitude: oxygen transport in mammals and birds. Physiol Rev. 1991;71 4:1135-72.

40. Weber RE, Jessen TH, Malte H and Tame J. Mutant hemoglobins (alpha 119-Ala and beta 55-Ser): functions related to high-altitude respiration in geese. J Appl Physiol (1985). 1993;75 6:2646-55.

41. Jessen TH, Weber RE, Fermi G, Tame J and Braunitzer G. Adaptation of bird hemoglobins to high altitudes: demonstration of molecular mechanism by protein engineering. Proceedings of the National Academy of Sciences of the United States of America. 1991;88 15:6519-22.

42. McCracken KG, Barger CP and Sorenson MD. Phylogenetic and structural analysis of the HbA (alphaA/betaA) and HbD (alphaD/betaA) hemoglobin genes in two high-altitude waterfowl from the Himalayas and the Andes: Bar-headed goose (Anser indicus) and Andean goose (Chloephaga melanoptera). Mol Phylogenet Evol. 2010;56 2:649-58. doi:10.1016/j.ympev.2010.04.034.

43. Gnerre S, Maccallum I, Przybylski D, Ribeiro FJ, Burton JN, Walker BJ, et al. High-quality draft assemblies of mammalian genomes from massively parallel sequence data. Proceedings of the National Academy of Sciences of the United States of America. 2011;108 4:1513-8. doi:10.1073/pnas.1017351108.

44. Chu TC, Lu CH, Liu T, Lee GC, Li WH and Shih AC. Assembler for de novo assembly of large genomes. Proceedings of the National Academy of Sciences of the United States of America. 2013;110 36:E3417-24. doi:10.1073/pnas.1314090110.

45. Margulies M, Egholm M, Altman WE, Attiya S, Bader JS, Bemben LA, et al. Genome sequencing in microfabricated high-density picolitre reactors. Nature. 2005;437 7057:376-80. doi:10.1038/nature03959.

46. Simpson JT and Durbin R. Efficient de novo assembly of large genomes using compressed data structures. Genome research. 2012;22 3:549-56. doi:10.1101/gr.126953.111.

47. Luo R, Liu B, Xie Y, Li Z, Huang W, Yuan J, et al. SOAPdenovo2: an empirically improved memory-efficient short-read de novo assembler. Gigascience. 2012;1 1:18. doi:10.1186/2047-217X-1-18.

48. Eo SH, Bininda-Emonds OR and Carroll JP. A phylogenetic supertree of the fowls (Galloanserae, Aves). Zoologica Scripta. 2009;38 5:465-81.

49. Kimball RT, Mary CM and Braun EL. A macroevolutionary perspective on multiple sexual traits in the phasianidae (galliformes). Int J Evol Biol. 2011;2011:423938. doi:10.4061/2011/423938.

50. Dunn CW, Hejnol A, Matus DQ, Pang K, Browne WE, Smith SA, et al. Broad phylogenomic sampling improves resolution of the animal tree of life. Nature. 2008;452 7188:745-9. doi:10.1038/nature06614.

51. Naylor GJ and Brown WM. Structural biology and phylogenetic estimation. Nature. 1997;388 6642:527-8. doi:10.1038/41460.

52. Rosenberg MS and Kumar S. Incomplete taxon sampling is not a problem for phylogenetic inference. Proceedings of the National Academy of Sciences of the United States of America. 2001;98 19:10751-6. doi:10.1073/pnas.191248498.

53. Li M, Tian S, Jin L, Zhou G, Li Y, Zhang Y, et al. Genomic analyses identify distinct patterns of selection in domesticated pigs and Tibetan wild boars. Nat Genet. 2013;45 12:1431-8. doi:10.1038/ng.2811.

54. Zhang B, Qiangba Y, Shang P, Wang Z, Ma J, Wang L, et al. A Comprehensive MicroRNA Expression Profile Related to Hypoxia Adaptation in the Tibetan Pig. PLoS One. 2015;10 11:e0143260. doi:10.1371/journal.pone.0143260.

55. Hsu F and Mao Y. The structure of phosphoinositide phosphatases: Insights into substrate specificity and catalysis. Biochim Biophys Acta. 2015;1851 6:698-710. doi:10.1016/j.bbalip.2014.09.015.

56. Rapoport SI, Primiani CT, Chen CT, Ahn K and Ryan VH. Coordinated Expression of Phosphoinositide Metabolic Genes during Development and Aging of Human Dorsolateral Prefrontal Cortex. PLoS One. 2015;10 7:e0132675. doi:10.1371/journal.pone.0132675.

57. Tan J, Yu CY, Wang ZH, Chen HY, Guan J, Chen YX, et al. Genetic variants in the inositol phosphate metabolism pathway and risk of different types of cancer. Sci Rep. 2015;5:8473. doi:10.1038/srep08473.

58. Zhu L, Li M, Li X, Shuai S, Liu H, Wang J, et al. Distinct expression patterns of genes associated with muscle growth and adipose deposition in tibetan pigs: a possible adaptive mechanism for high altitude conditions. High Alt Med Biol. 2009;10 1:45-55. doi:10.1089/ham.2008.1042.

59. Ullrich SE and Schmitt DA. The role of cytokines in UV-induced systemic immune suppression. Journal of dermatological science. 2000;23 Suppl 1:S10-2.

60. Baadsgaard O, Fox DA and Cooper KD. Human epidermal cells from ultraviolet light-exposed skin preferentially activate autoreactive CD4+2H4+ suppressor-inducer lymphocytes and CD8+ suppressor/cytotoxic lymphocytes. J Immunol. 1988;140 6:1738-44.
61. Dudley AC, Thomas D, Best J and Jenkins A. The STATs in cell stress-type responses. Cell Commun Signal. 2004;2 1:8. doi:10.1186/1478-811X-2-8.
62. Shuai K and Liu B. Regulation of JAK-STAT signalling in the immune system. Nat Rev Immunol. 2003;3 11:900-11. doi:10.1038/nri1226.
63. Stempien-Otero A, Karsan A, Cornejo CJ, Xiang H, Eunson T, Morrison RS, et al. Mechanisms of hypoxia-induced endothelial cell death. Role of p53 in apoptosis. J Biol Chem. 1999;274 12:8039-45.
64. Yang W, Qi Y, Lu B, Qiao L, Wu Y and Fu J. Gene expression variations in high-altitude adaptation: a case study of the Asiatic toad (Bufo gargarizans). BMC Genet. 2017;18 1:62. doi:10.1186/s12863-017-0529-z.
65. Storz JF and Cheviron ZA. Functional Genomic Insights into Regulatory Mechanisms of High-Altitude Adaptation. Adv Exp Med Biol. 2016;903:113-28. doi:10.1007/978-1-4899-7678-9_8.
66. Cheviron ZA and Brumfield RT. Genomic insights into adaptation to high-altitude environments. Heredity (Edinb). 2012;108 4:354-61. doi:10.1038/hdy.2011.85.
67. Bollmer JL, Vargas FH and Parker PG. Low MHC variation in the endangered Galapagos penguin (Spheniscus mendiculus). Immunogenetics. 2007;59 7:593-602. doi:10.1007/s00251-007-0221-y.
68. Wan QH, Zhu L, Wu H and Fang SG. Major histocompatibility complex class II variation in the giant panda (Ailuropoda melanoleuca). Mol Ecol. 2006;15 9:2441-50. doi:10.1111/j.1365-294X.2006.02966.x.
69. Zeng QQ, Zhong GH, He K, Sun DD and Wan QH. Molecular characterization of classical and nonclassical MHC class I genes from the golden pheasant (Chrysolophus pictus). Int J Immunogenet. 2016;43 1:8-17. doi:10.1111/iji.12245.
70. Kan XZ, Yang JK, Li XF, Chen L, Lei ZP, Wang M, et al. Phylogeny of major lineages of galliform birds (Aves: Galliformes) based on complete mitochondrial genomes. Genet Mol Res. 2010;9 3:1625-33. doi:10.4238/vol9-3gmr898.
71. Nishibori M, Shimogiri T, Hayashi T and Yasue H. Molecular evidence for hybridization of species in the genus Gallus except for Gallus varius. Anim Genet. 2005;36 5:367-75. doi:10.1111/j.1365-2052.2005.01318.x.

72. Liu T-K, Chen Y-G, Chen W-S and Jiang S-H. Rates of cooling and denudation of the Early Penglai Orogeny, Taiwan, as assessed by fission-track constraints. Tectonophysics. 2000;320 1:69-82.
73. Osozawa S, Shinjo R, Armid A, Watanabe Y, Horiguchi T and Wakabayashi J. Palaeogeographic reconstruction of the 1.55 Ma synchronous isolation of the Ryukyu Islands, Japan, and Taiwan and inflow of the Kuroshio warm current. International Geology Review. 2012;54 12:1369-88.
74. Boetzer M, Henkel CV, Jansen HJ, Butler D and Pirovano W. Scaffolding pre-assembled contigs using SSPACE. Bioinformatics. 2011;27 4:578-9. doi:10.1093/bioinformatics/btq683.
75. Gurevich A, Saveliev V, Vyahhi N and Tesler G. QUAST: quality assessment tool for genome assemblies. Bioinformatics. 2013;29 8:1072-5. doi:10.1093/bioinformatics/btt086.
76. Bolger AM, Lohse M and Usadel B. Trimmomatic: a flexible trimmer for Illumina sequence data. Bioinformatics. 2014;30 15:2114-20. doi:10.1093/bioinformatics/btu170.
77. Leggett RM, Clavijo BJ, Clissold L, Clark MD and Caccamo M. NextClip: an analysis and read preparation tool for Nextera Long Mate Pair libraries. Bioinformatics. 2014;30 4:566-8. doi:10.1093/bioinformatics/btt702.
78. Wood DE and Salzberg SL. Kraken: ultrafast metagenomic sequence classification using exact alignments. Genome biology. 2014;15 3:R46. doi:10.1186/gb-2014-15-3-r46.
79. Langmead B and Salzberg SL. Fast gapped-read alignment with Bowtie 2. Nat Methods. 2012;9 4:357-9. doi:10.1038/nmeth.1923.
80. Kim D, Pertea G, Trapnell C, Pimentel H, Kelley R and Salzberg SL. TopHat2: accurate alignment of transcriptomes in the presence of insertions, deletions and gene fusions. Genome biology. 2013;14 4:R36. doi:10.1186/gb-2013-14-4-r36.
81. Dobin A, Davis CA, Schlesinger F, Drenkow J, Zaleski C, Jha S, et al. STAR: ultrafast universal RNA-seq aligner. Bioinformatics. 2013;29 1:15-21. doi:10.1093/bioinformatics/bts635.
82. Quinlan AR and Hall IM. BEDTools: a flexible suite of utilities for comparing genomic features. Bioinformatics. 2010;26 6:841-2. doi:10.1093/bioinformatics/btq033.
83. Krzywinski M, Schein J, Birol I, Connors J, Gascoyne R, Horsman D, et al. Circos: an information aesthetic for comparative genomics. Genome Res. 2009;19 9:1639-45. doi:10.1101/gr.092759.109.

84. Slater GS and Birney E. Automated generation of heuristics for biological sequence comparison. BMC Bioinformatics. 2005;6:31. doi:10.1186/1471-2105-6-31.

85. Grabherr MG, Haas BJ, Yassour M, Levin JZ, Thompson DA, Amit I, et al. Full-length transcriptome assembly from RNA-Seq data without a reference genome. Nature biotechnology. 2011;29 7:644-52. doi:10.1038/nbt.1883.

86. Haas BJ, Delcher AL, Mount SM, Wortman JR, Smith RK, Jr., Hannick LI, et al. Improving the Arabidopsis genome annotation using maximal transcript alignment assemblies. Nucleic Acids Res. 2003;31 19:5654-66.

87. Eddy SR. Profile hidden Markov models. Bioinformatics. 1998;14 9:755-63.

88. Holt C and Yandell M. MAKER2: an annotation pipeline and genome-database management tool for second-generation genome projects. BMC Bioinformatics. 2011;12:491. doi:10.1186/1471-2105-12-491.

89. Edgar RC. MUSCLE: multiple sequence alignment with high accuracy and high throughput. Nucleic Acids Res. 2004;32 5:1792-7. doi:10.1093/nar/gkh340.

90. Castresana J. Selection of conserved blocks from multiple alignments for their use in phylogenetic analysis. Mol Biol Evol. 2000;17 4:540-52.

91. Stamatakis A. RAxML version 8: a tool for phylogenetic analysis and post-analysis of large phylogenies. Bioinformatics. 2014;30 9:1312-3. doi:10.1093/bioinformatics/btu033.

92. Posada D. Using MODELTEST and PAUP* to select a model of nucleotide substitution. Current protocols in bioinformatics / editoral board, Andreas D Baxevanis [et al]. 2003;Chapter 6:Unit 6 5. doi:10.1002/0471250953.bi0605s00.

93. Hedges SB, Dudley J and Kumar S. TimeTree: a public knowledge-base of divergence times among organisms. Bioinformatics. 2006;22 23:2971-2. doi:10.1093/bioinformatics/btl505.

94. Bell MA and Lloyd GT. Strap: an R package for plotting phylogenies against stratigraphy and assessing their stratigraphic congruence. Palaeontology. 2015;58 2:379-89.

95. Han MV, Thomas GW, Lugo-Martinez J and Hahn MW. Estimating gene gain and loss rates in the presence of error in genome assembly and annotation using CAFE 3. Mol Biol Evol. 2013;30 8:1987-97. doi:10.1093/molbev/mst100.

96. Yang Z. PAML 4: phylogenetic analysis by maximum likelihood. Mol Biol Evol. 2007;24 8:1586-91. doi:10.1093/molbev/msm088.

97. Bleazard T, Lamb JA and Griffiths-Jones S. Bias in microRNA functional enrichment analysis. Bioinformatics. 2015;31 10:1592-8. doi:10.1093/bioinformatics/btv023.

98. Lu TP, Lee CY, Tsai MH, Chiu YC, Hsiao CK, Lai LC, et al. miRSystem: an integrated system for characterizing enriched functions and pathways of microRNA targets. PLoS One. 2012;7 8:e42390. doi:10.1371/journal.pone.0042390.

99. Bao J and Reecy JM. CateGOrizer: a web-based program to batch analyze gene ontology classification categories. Online Journal of Bioinformatics. 2008;9 2:108-12.

100. Bindea G, Mlecnik B, Hackl H, Charoentong P, Tosolini M, Kirilovsky A, et al. ClueGO: a Cytoscape plug-in to decipher functionally grouped gene ontology and pathway annotation networks. Bioinformatics. 2009;25 8:1091-3. doi:10.1093/bioinformatics/btp101.

101. Kanehisa M and Goto S. KEGG: kyoto encyclopedia of genes and genomes. Nucleic Acids Res. 2000;28 1:27-30.

102. Kearse M, Moir R, Wilson A, Stones-Havas S, Cheung M, Sturrock S, et al. Geneious Basic: an integrated and extendable desktop software platform for the organization and analysis of sequence data. Bioinformatics. 2012;28 12:1647-9. doi:10.1093/bioinformatics/bts199.

103. Bernt M, Donath A, Juhling F, Externbrink F, Florentz C, Fritzsch G, et al. MITOS: improved de novo metazoan mitochondrial genome annotation. Mol Phylogenet Evol. 2013;69 2:313-9. doi:10.1016/j.ympev.2012.08.023.

104. Lee CY, Hsieh PH, Chiang LM, Chattopadhyay A, Li KY, Lee Y, et al. Supporting data for "Whole-Genome De Novo Sequencing Reveals Unique Genes that Contributed to the Adaptive Evolution of the Mikado Pheasant" GigaScience Database 2018. http://dx.doi.org/10.5524/100431

Figure Legends

Figure 1: A chromosome-level comparison of the Mikado pheasant and the chicken. **(A)** A syntenic map of the Mikado pheasant and chicken genomes. The x-axis specifies the chromosome position in the chicken, whereas the y-axis specifies the scaffold position in the Mikado pheasant. The red dots (or lines) indicate that the sequences were aligned in the same orientation, and the blue dots indicate an alignment with a reverse complement. **(B)** A chord diagram of scaffolds with a total length greater than 500 kb and an alignment length greater than 10 kb. The orange perimeters specify the chromosomes (chr) of the chicken, whereas the purple perimeters specify the scaffolds (sc) of the Mikado pheasant. The red links represent the sequences aligned in the same orientation, and the blue links represent an alignment with a reverse complement. Arrows colored in yellow indicate the scaffolds that were fully aligned, and grey ones indicate the multiple alignment.

Figure 2: Evolution of gene families among various animal species. A phylogenetic tree was reconstructed based on 5287 single-copy orthologs of 10 species. The most recent common ancestor (MRCA) contains 18 220 gene families that were used to examine gene families with expansions or contractions. The numbers of gene families with significant expansions and contractions are shown in red and blue, respectively, at each branch. The divergence times and associated 95% confidence intervals (in parentheses) are indicated at the nodes of the tree in Mya. All nodes had 100% support in 500 bootstrap replicates.

Figure 3: An identity plot of the MHC regions of the Mikado pheasant and the chicken. The chicken MHC sequence was downloaded from GenBank (AB268588).

Its nucleotide sequence from 17 978 to 241 251 was aligned against the Mikado pheasant MHC sequence from 2615 to 229 500 in scaffold208. The gene structure boxes on the horizontal and vertical axes, respectively, represent the gene loci in the Mikado pheasant and the chicken. Boxes with different sizes exhibit different gene locus sizes, and red/blue coloring indicates genes in forward/reverse orientation. The red dots (or lines) on the diagonal indicate that the sequences were aligned in the same orientation, whereas the blue dots indicate alignments with reverse complements. The green dotted lines highlight the sequence of the inverted *TAPBP* locus and *TAP1-TAP2* block. The orange peaks show the read counts on a natural log scale of the gene expression based on our RNA-Seq data. The box plot colored in purple indicates d_N/d_S ratios of genes.

Figure 4: A phylogenetic tree of *Syrmaticus* pheasants. The divergence times and associated 95% confidence intervals shown in parentheses are given at the branch nodes of the tree in Mya.

Tables

Table 1: DNA contigs and scaffolds from the genomic data of the Mikado pheasant.

	**Contigs**	**Scaffolds**
Total length	1 054 607 905	1 035 947 982
Maximum length	195 342	50 275 205
Number of Ns	0	19 577 473
Average length	5050	110 714
N50*	13 461	11 461 115
N75*	6528	5 708 287
L50†	22 195	28
L75†	50 081	59
Counts ≥300 bp	208 810	-
Counts ≥1 kb	123 006	9357
Counts ≥5 kb	61 237	1489
Counts ≥10 kb	32 868	928

* The N50/N75 length is defined as the shortest sequence length at 50%/75% of the genome.

† The L50/L75 count is defined as the smallest number of contigs (or scaffolds) that those length sum produces N50/N75.

Values of the genome assembly were calculated using the contigs ≥300 bp and scaffolds ≥1000 bp.

Table 2: Coding sequences of MHC-B genes in the Mikado pheasant and comparisons with the chicken.

	Mikado pheasant					Chicken					
**Gene**	**Position**	**Strand**	**Gene length**	**Amino acid length**	**Exon**	**Aligned base**	**Nucleotide identity (%)**	**Aligned amino acid**	**Amino acid identity (%)**	**Amino acid substitutions**	**d_N/d_S***
KIFC1	2615-5304	+	1140	380	7	1131	91.76	377	90.53	33	0.8669
Blec3	8997-11221	-	552	183	5	507	82.43	168	78.14	25	0.3821
Bzfp3	12126-18213	+	1449	482	13	1569	85.8	522	83.62	40	0.1884
TRIM7.2	19507-24562	-	1518	505	7	1518	95.98	505	98.61	7	0.0391
Bzfp2[†]	27027-29946	+	1368	455	4	1396	70.41	N/A	N/A	N/A	0.2438
Bzfp1	31049-33298	-	1425	474	2	1426	88.23	471	86.79	54	0.1900
44G24.1	37266-37673	-	408	136	1	408	85.78	136	80.15	27	0.2762
IL4I1	42730-46759	+	1578	525	6	1572	92.25	523	93.75	25	0.1011
TRIM7.1	51325-62131	-	1758	585	8	1767	92.49	588	92.69	40	0.1545
HEP21	63362-64247	-	324	107	3	324	93.52	107	91.59	9	0.2148
TRIM39.2	70980-74640	-	1392	464	6	1389	93.68	463	94.61	24	0.1167
TRIM27.2	76988-80522	+	1431	476	7	1431	94.13	476	92.23	37	0.2415
TRIM39.1	81560-85449	-	798	266	5	798	93.23	266	91.35	23	0.2753
TRIM27.1	86518-90228	-	1485	495	7	1485	94.48	495	94.34	28	0.1715
TRIM41	91918-96605	+	1656	551	7	1770	89.58	589	91.71	7	0.0375
GNB2L1	98038-101512	-	954	317	8	954	96.86	317	100	0	N/A
BTN1	103411-114264	+	930	309	8	939	74.64	339	57.26	96	0.8357
BTN2	117466-120157	+	1461	487	7	1481	90.41	469	83.37	52	0.3996
BG1	124105-125436	-	549	183	3	546	91.99	182	87.98	21	0.5591
Blec2	131358-133021	-	579	192	5	579	86.32	190	71.88	52	0.9375
Blec1	135818-137846	+	567	188	5	567	92.59	188	88.3	22	0.4683
BLB1	138411-139729	-	339	112	3	345	83.38	113	42.98	62	0.7904
TAPBP	140657-144216	+	1293	430	8	1293	92.19	430	89.77	44	0.3179
BLB2[‡]	N/A	N/A	792	263	N/A	792	92.93	263	85.93	37	1.4489
BRD2	150146-156295	-	2976	991	13	3078	86.85	776	75.28	30	0.0306
DMA	160545-162778	+	789	263	4	789	92.65	263	89.73	27	0.4528
DMB1	163010-165184	+	930	310	6	930	91.29	310	86.45	42	0.4978
DMB2	165617-168363	+	768	256	5	768	92.71	256	92.58	19	0.1622
BF1	169254-170740	+	996	331	5	1001	83.66	345	64.12	95	0.7116
TAP2	172793-176021	-	2100	700	9	2100	92.48	700	93.14	48	0.1675
TAP1	176574-180981	+	1752	584	11	1739	93.21	580	92.81	38	0.2191
BF2	181900-184038	-	1530	509	6	1213	62.28	326	57.39	119	0.8157
C4	185102-199258	+	5031	1676	40	4998	93.33	1665	93.2	101	0.1974
CenpA	199593-200795	+	396	131	4	396	96.72	131	99.24	1	0.0324
CYP21	201291-205141	+	1431	477	11	1431	92.67	477	94.13	28	0.7109
TNXB	209524-215604	-	2472	824	10	2496	92.14	832	92.34	50	0.2002
LTB4R1	221450-222538	+	1089	363	1	1089	94.12	363	94.49	20	0.1954
CD1A2	223740-225788	-	1044	348	6	1044	92.24	348	87.93	42	0.3796
CD1A1	227030-229500	-	1122	374	6	1122	93.4	374	90.64	35	0.3294

KIFC1, kinesin family member C1; Blec, C-type lectin-like receptor; Bzfp, B-locus zinc finger-like protein; TRIM, tripartite motif containing protein; 44G24.1, histone H2B-like protein; IL4I1, interleukin 4 induced 1; HEP21, hen egg protein 21 kDa; GNB2L1, guanine nucleotide binding-like protein; BTN, B-butyrophilin protein; BG1, BG-like antigen; CD1A1/A2, CD1-like proteins

* d_N/d_S = ratio of nonsynonymous (d_N) to synonymous (d_S) substitutions.

† Defined as a pseudogene in chicken.

‡ No predicted result was identified from the DNA assembly. The transcript sequence was alternatively derived from the transcriptome assembly by RNA-Seq.

Figure 1

Click here to download Figure Fig1.tif

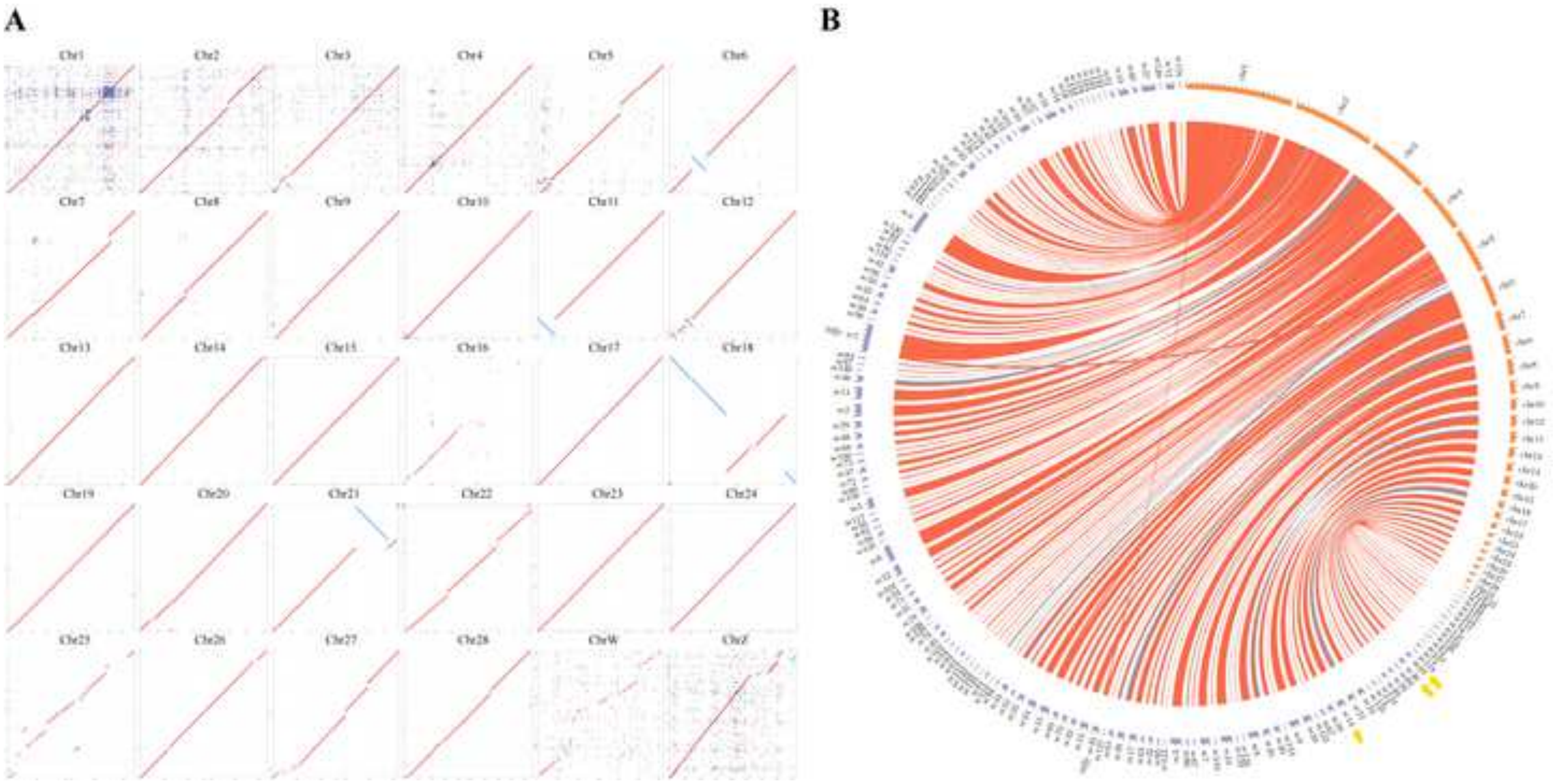

Figure 2

Click here to download Figure Fig2.tif

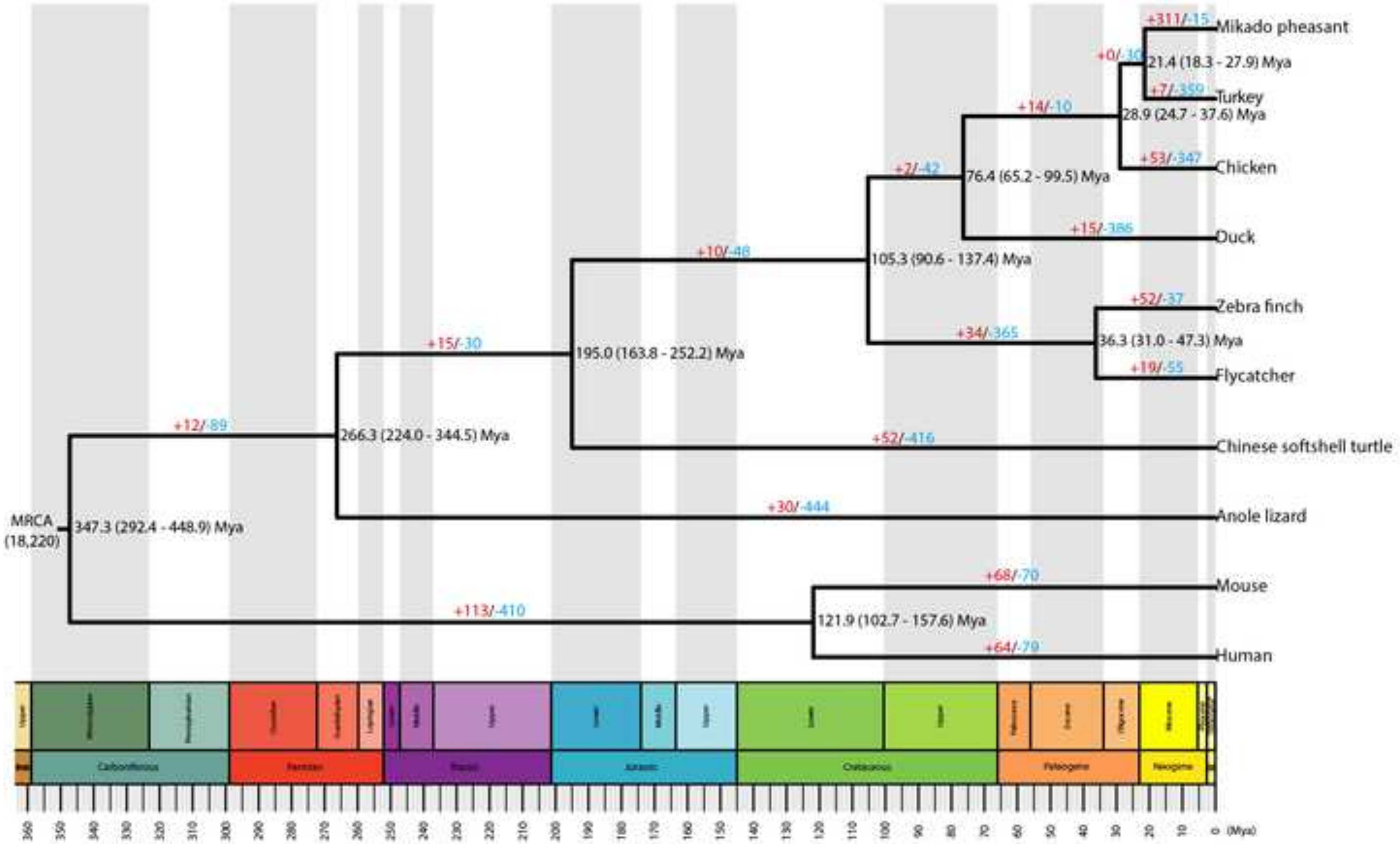

Figure 3

Click here to download Figure Fig3.tif

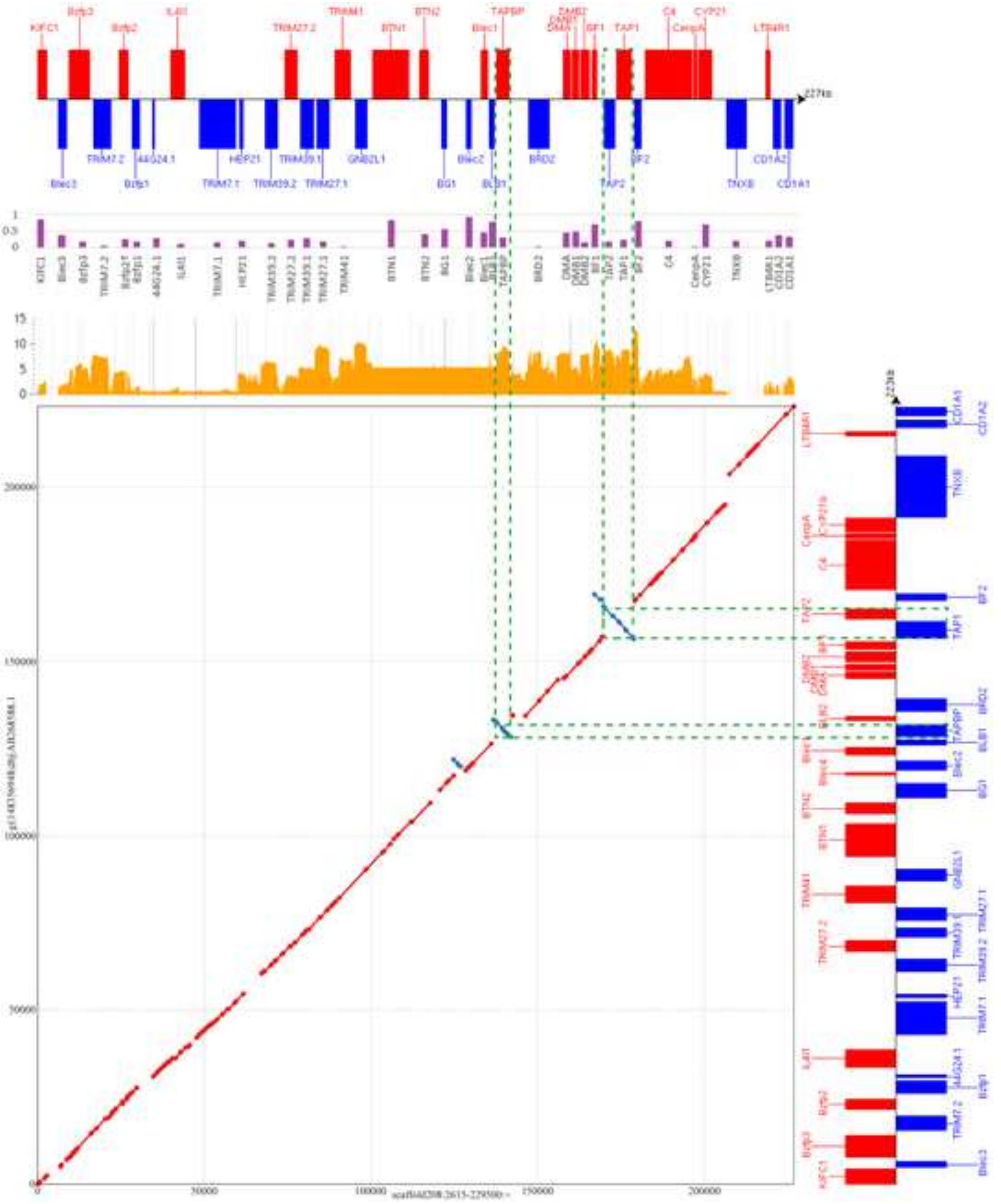

 Click here to download Figure Fig4.tif

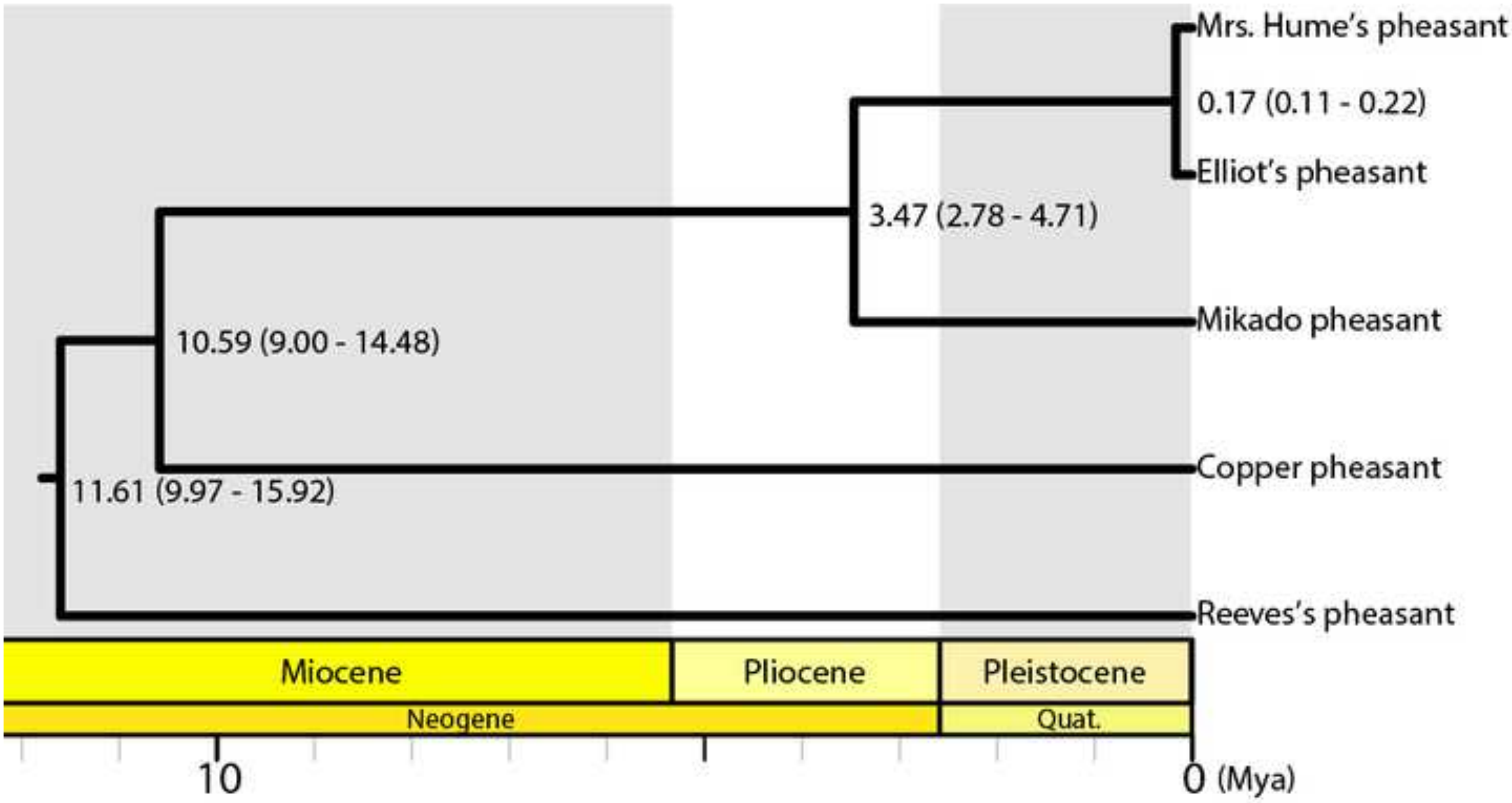

Additional file 1

Click here to access/download
Supplementary Material
Additional file 1_revised_ver.docx

Additional file 2

Click here to access/download
Supplementary Material
Additional file 2_revised_ver.xlsx
