# Supplementary material for: Whole-genome de novo sequencing reveals unique genes that contributed to the adaptive evolution of the Mikado pheasant
Source: Gigascience. 2018 May 2;7(5):giy044. doi: 10.1093/gigascience/giy044 (PMC5941149; doi:10.1093/gigascience/giy044)
Supplement: Additional Files [file giy044_supp.zip › Additional file 1_revised_ver.docx]

**Additional file 1**

**Whole-Genome *De Novo* Sequencing Reveals Unique Genes that Contributed to the Adaptive Evolution of the Mikado Pheasant**

**Lee, Hsieh *et al.***

[Supplementary Figures 2](#_Toc503757894)

[Figure S1: Photos of the Mikado pheasant. 2](#_Toc503757895)

[Figure S2: Geographic distribution of the Mikado pheasant. 3](#_Toc503757896)

[Figure S3: GC content distribution of the genomes from the Mikado pheasant, chicken, turkey, duck, and zebra finch genomes. 4](#_Toc503757897)

[Figure S4: Cumulative length plots for scaffolds showing sequences in length longer than (A) 1000 bp and (B) 392 444 bp (N95). 5](#_Toc503757898)

[Figure S5: Nx plot for all scaffolds. 6](#_Toc503757899)

[Figure S6: Distribution plot of per-base alignment coverage from assembled scaffolds. 7](#_Toc503757900)

[Figure S7: The chromosome-level alignment of the Mikado pheasant genome with turkey and zebra finch. 8](#_Toc503757901)

[Figure S8: Venn diagram of gene families. 9](#_Toc503757902)

[Figure S9: Gene Ontology enrichment of genes with positive selection in the Mikado pheasant. 10](#_Toc503757903)

[Figure S10: Amino acid comparison of the hemoglobin alpha-A subunit in 6 avian species. 11](#_Toc503757904)

[Figure S11: An identity plot of the MHC regions from the Mikado pheasant and the turkey. 12](#_Toc503757905)

[Figure S12: Workflow of the post-check for contamination in the Mikado pheasant genome. 13](#_Toc503757906)

[Supplementary Tables 14](#_Toc503757907)

[Table S1: Basic statistics of sequencing data collected from Mikado pheasant samples. 14](#_Toc503757908)

[Table S2: Statistics of repeated regions detected from the Mikado pheasant genome. 15](#_Toc503757909)

[Table S3: Statistics of annotated genes in the Mikado pheasant compared with chicken, turkey, duck, and zebra finch. 16](#_Toc503757910)

[Table S4: Summary of DNA read mapping rates. 17](#_Toc503757911)

[Table S5: Summary of RNA read mapping rates. 18](#_Toc503757912)

[Table S6: BUSCO benchmark results. 19](#_Toc503757913)

[Table S7. Alignment of the Mikado pheasant DNA assembly to chicken chromosomes. 20](#_Toc503757914)

[Table S9: Gene Ontology categories enriched for contracted gene families in the Mikado pheasant. 21](#_Toc503757915)

[Table S12: KEGG pathways enriched for metabolism with positively selected genes of the Mikado pheasant 22](#_Toc503757916)

[Table S15: KEGG pathways enriched for immune response with positively selected genes of the Mikado pheasant. 23](#_Toc503757917)

[Table S16: Gene annotation of the mitochondrial genome of the Mikado pheasant. 24](#_Toc503757918)

[Table S17: Statistics of the Mikado pheasant assemblies using six *de novo* genome assembly software programs. 25](#_Toc503757919)

# Supplementary Figures


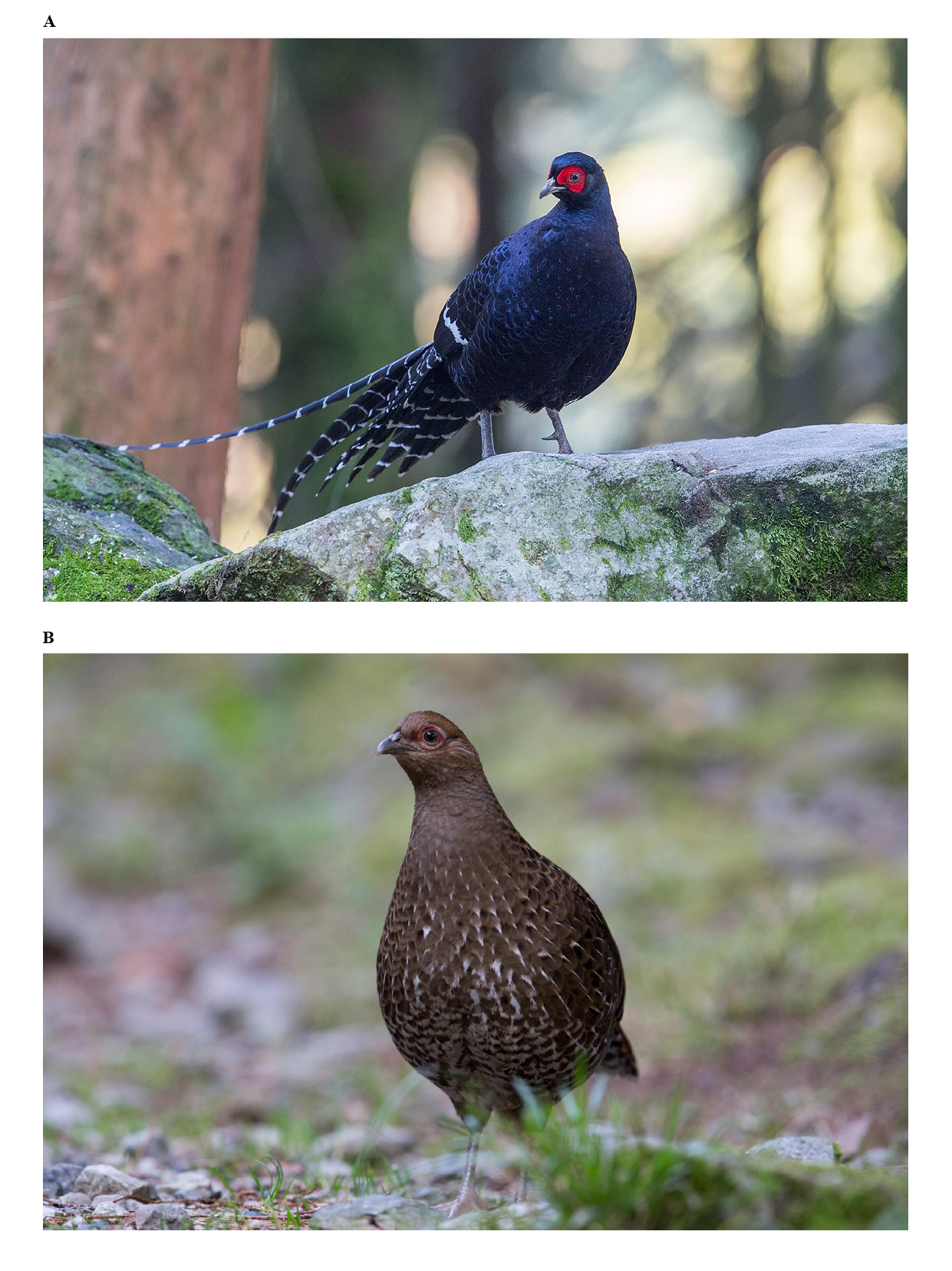


Figure S1: Photos of the Mikado pheasant. **(A)** Male. **(B)** Female.


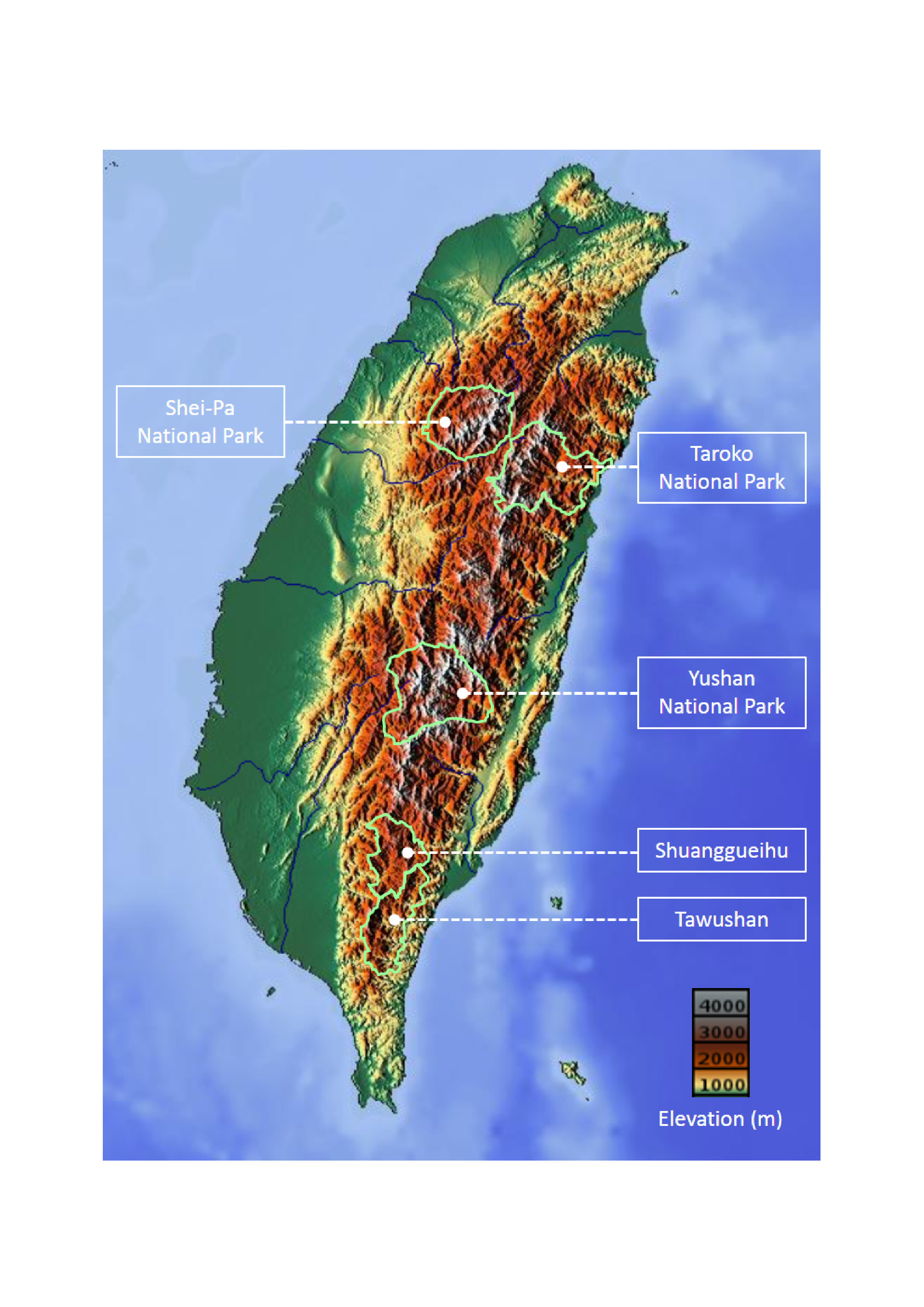


Figure S2: Geographic distribution of the Mikado pheasant. The light green areas delineate the primary habitats available for the Mikado pheasant.


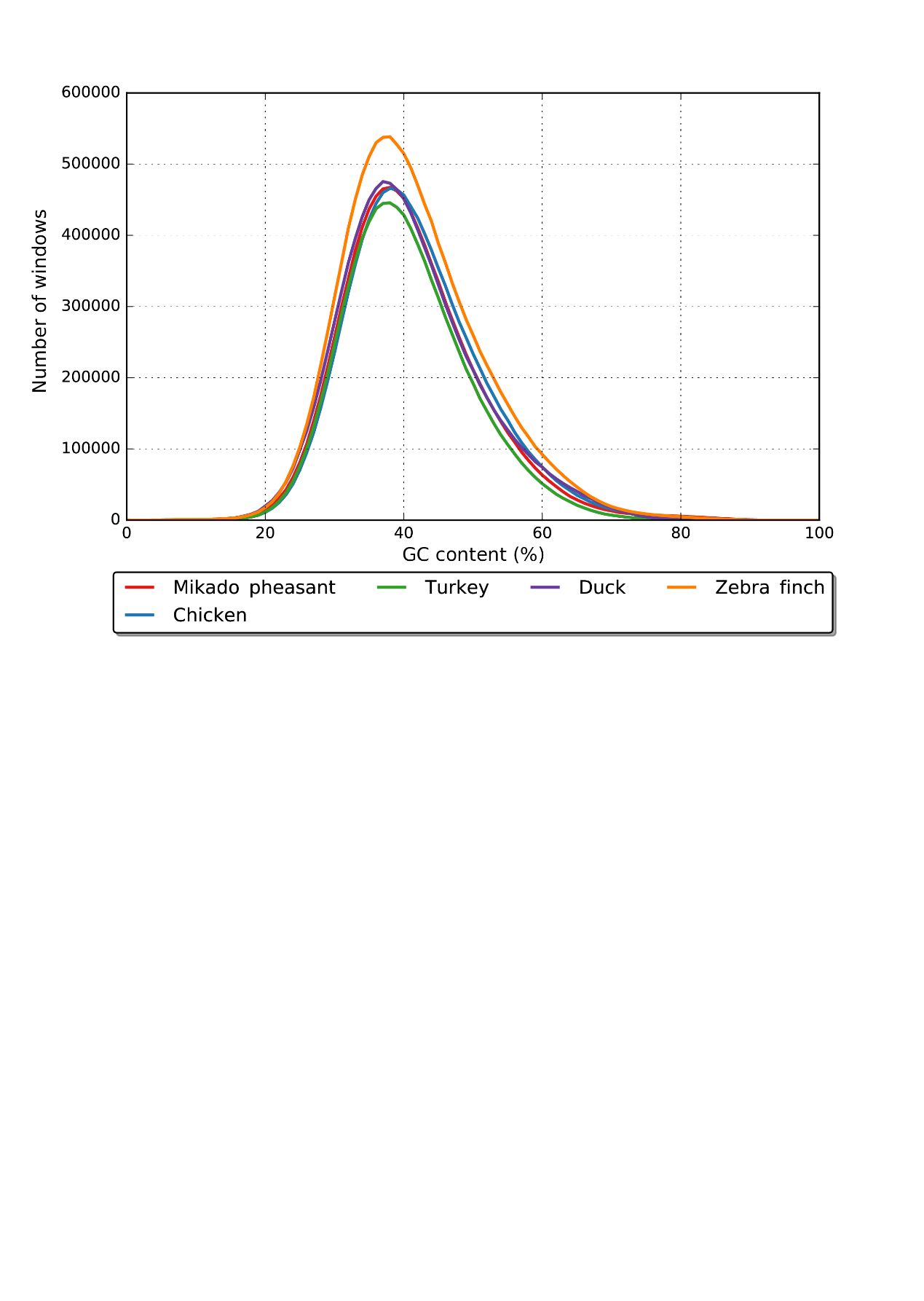


Figure S3: GC content distribution of the genomes from the Mikado pheasant, chicken, turkey, duck, and zebra finch genomes. The x-axis represents the percentage of GC content, whereas the y-axis represents the number of non-overlapping 100 bp windows in which the GC content was equal to x percentage.


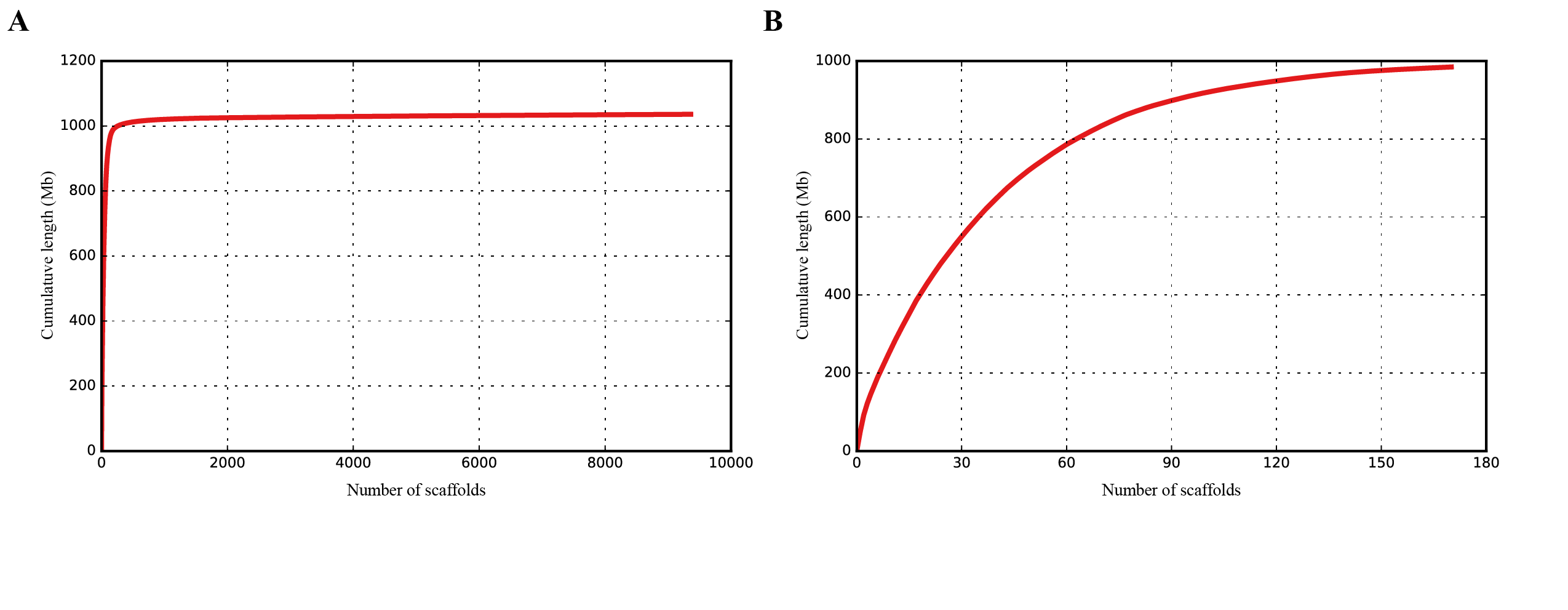


Figure S4: Cumulative length plots for scaffolds showing sequences in length longer than (A) 1000 bp and (B) 392 444 bp (N95). The x-axis indicates the scaffold number, sorted by length in descending order, whereas the y-axis indicates the cumulative length of scaffolds.


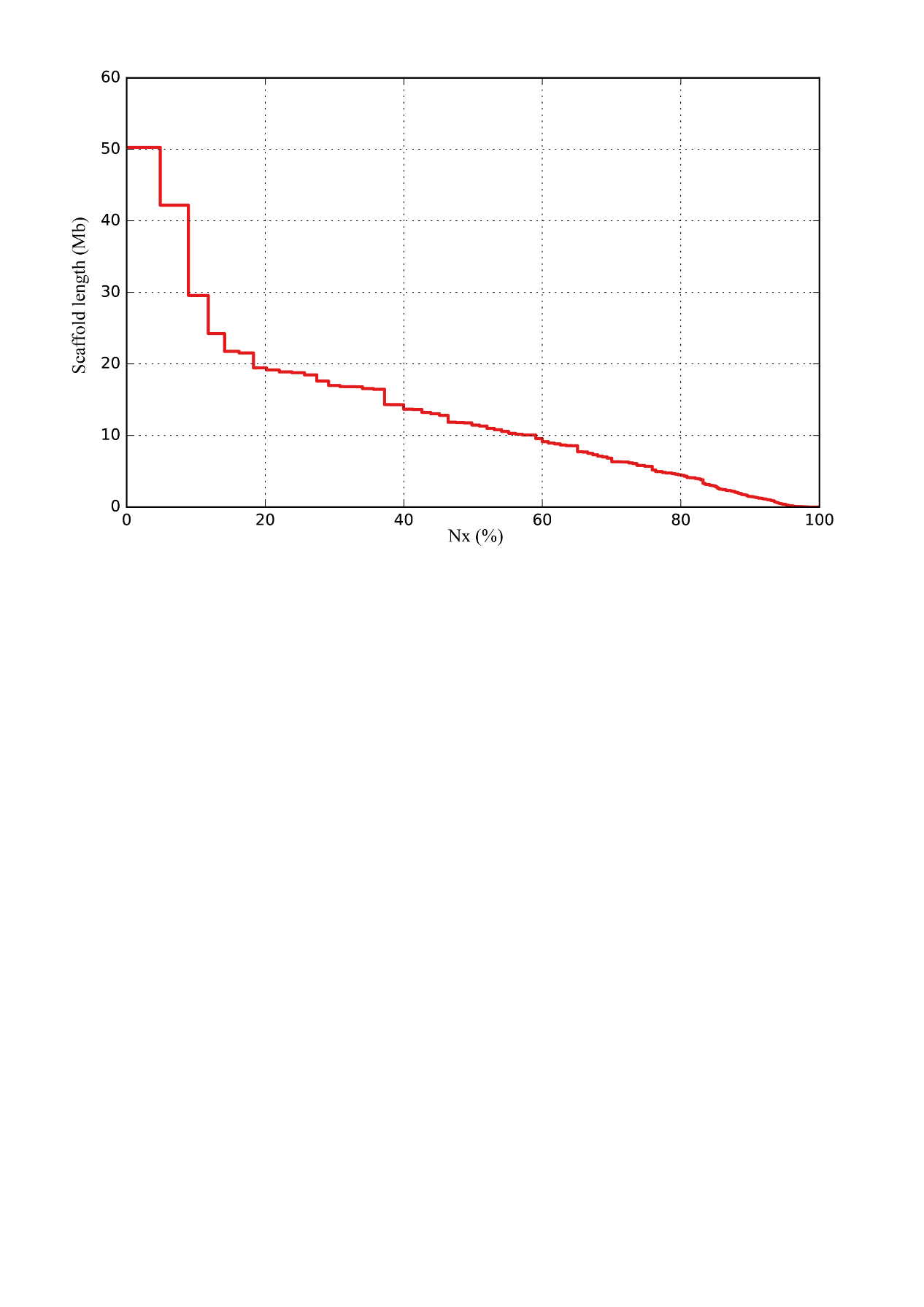


Figure S5: Nx plot for all scaffolds. Only sequences longer than 1000 bp in length were considered. The x-axis shows the proportion of the total genome length, whereas the y-axis shows the corresponding length of each scaffold, ranked from the largest scaffold to the smallest scaffold.


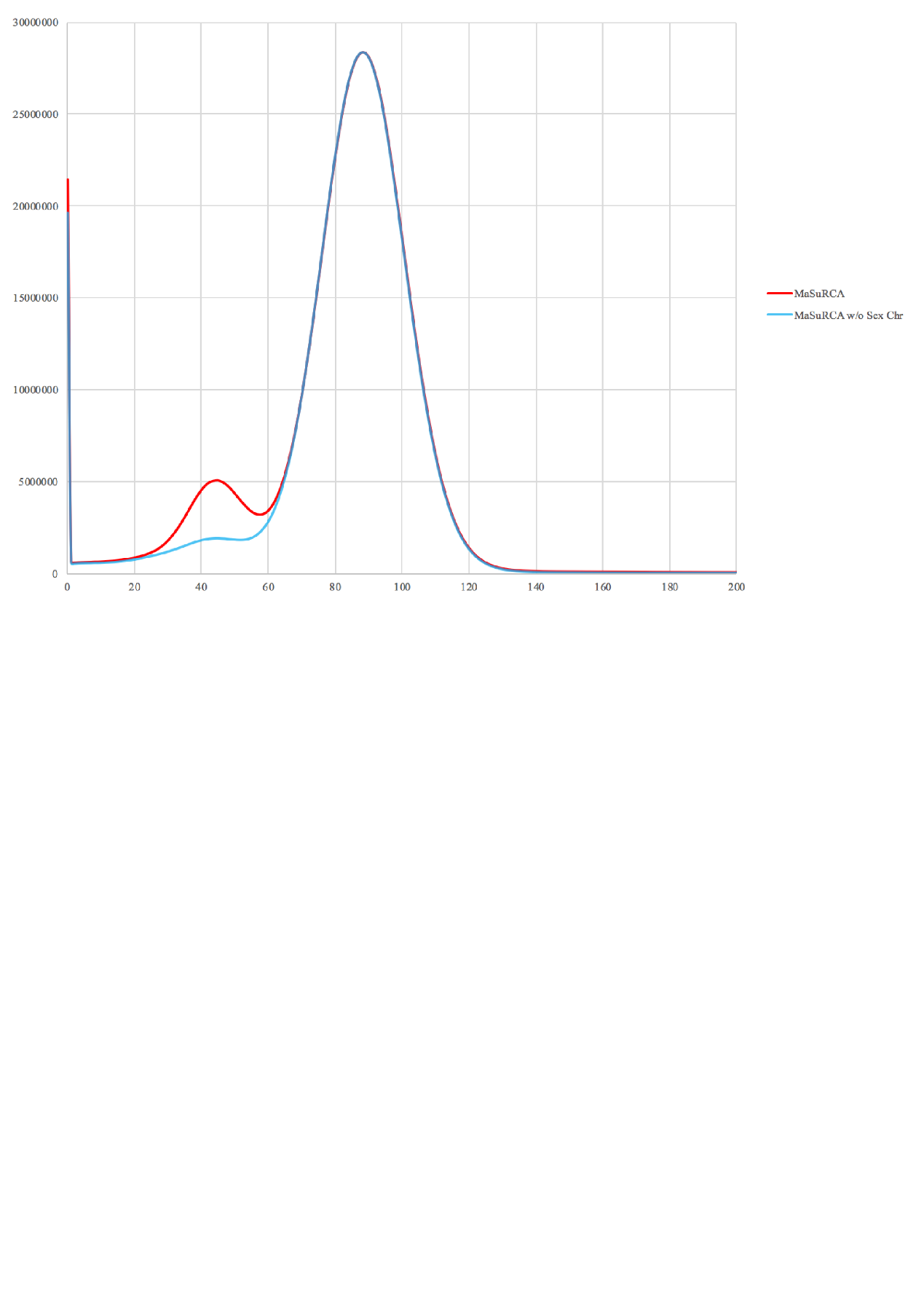


Figure S6: Distribution plot of per-base alignment coverage from assembled scaffolds. The x-axis represents the depth of coverage and the y-axis represents the frequency of covered depth. The coverage of the allosome was half compared to that of autosomes, as the DNA reads were extracted from a female species.


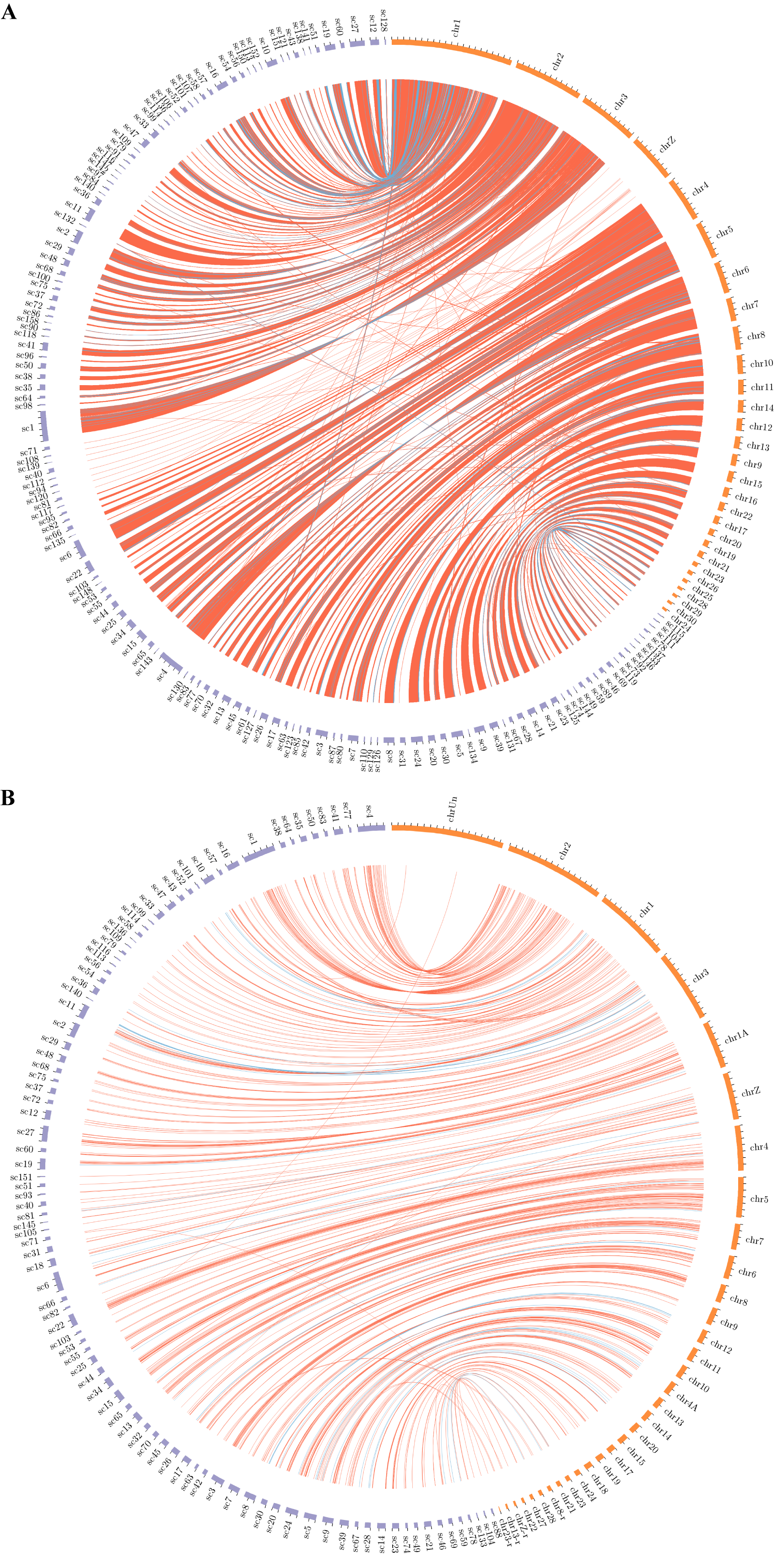


Figure S7: The chromosome-level alignment of the Mikado pheasant genome with turkey and zebra finch. Scaffolds with a total length greater than 500 kb and an alignment length greater than **(A)** 10 kb in the turkey and **(B)** 2 kb in the zebra finch are shown. The orange perimeters specify the chromosomes of the turkey/zebra finch, whereas the purple perimeters specify the scaffolds of the Mikado pheasant. The red links represent the sequences aligned in the same orientation, and the blue links represent an alignment with a reverse complement.


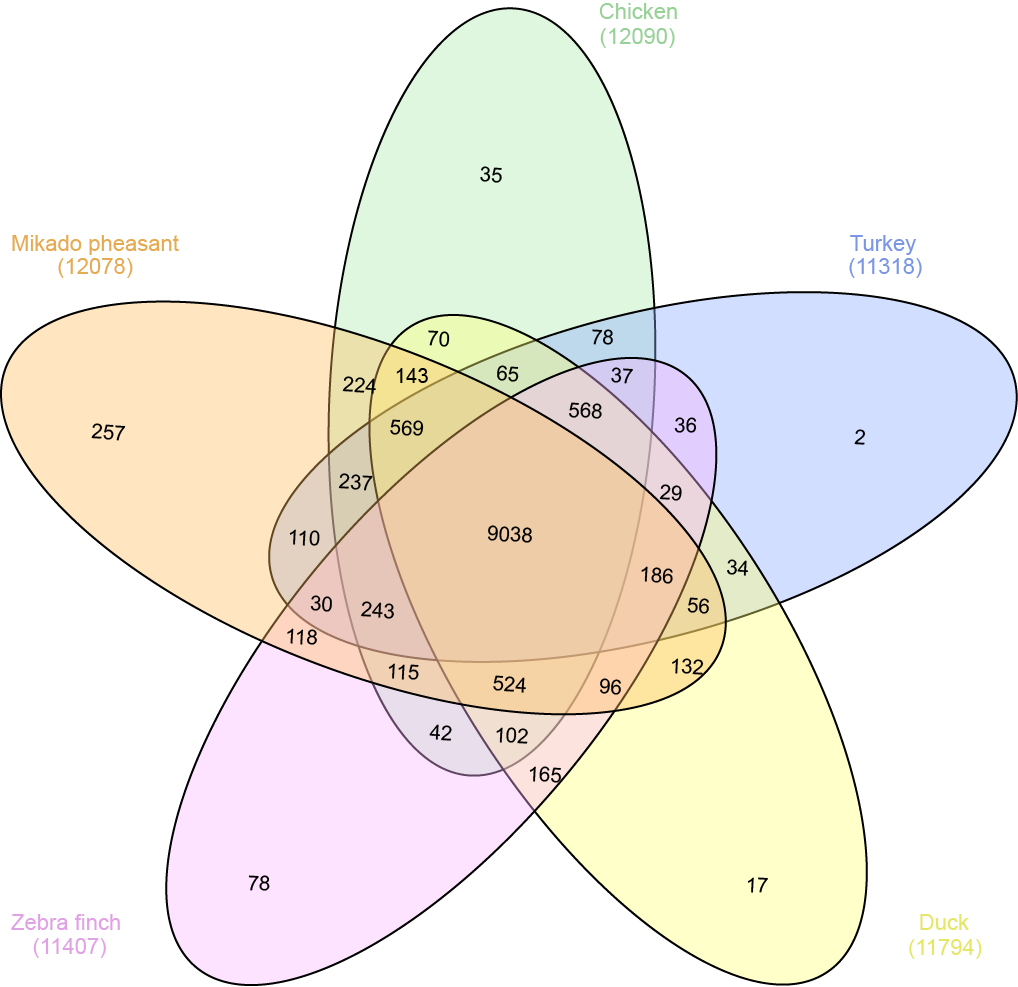


Figure S8: Venn diagram of gene families. The numbers of gene families from the Mikado pheasant, chicken, turkey, duck, and zebra finch are shown in parentheses. In total, 13 436 gene families were identified in this work. Additionally, 14 375 genes in the Mikado pheasant were classified into 12 078 gene families.


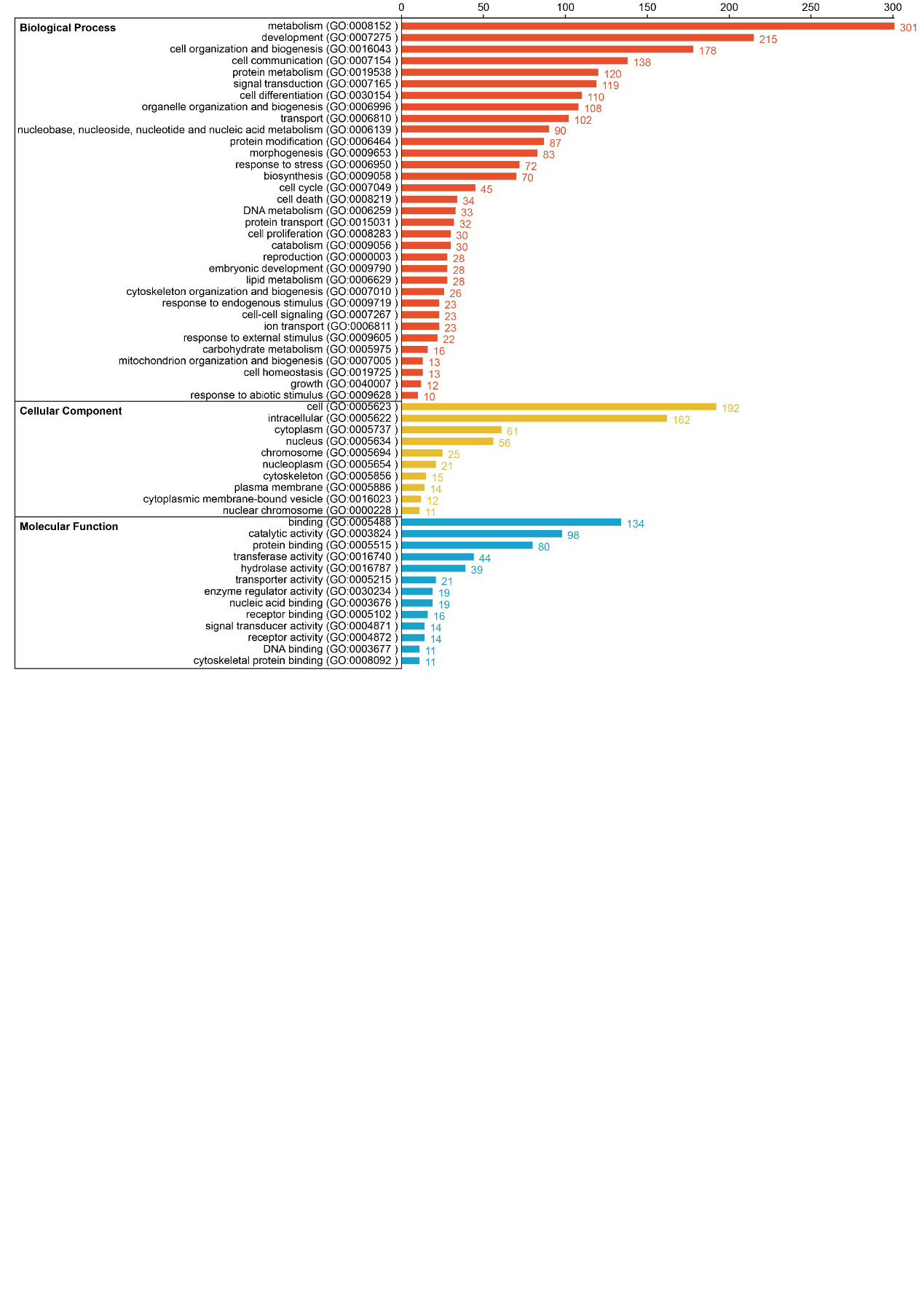


Figure S9: Gene Ontology enrichment of genes with positive selection in the Mikado pheasant. The x-axis represents the number of enriched GO functions classified into GOSlim categories. The y-axis indicates the GOSlim categories.


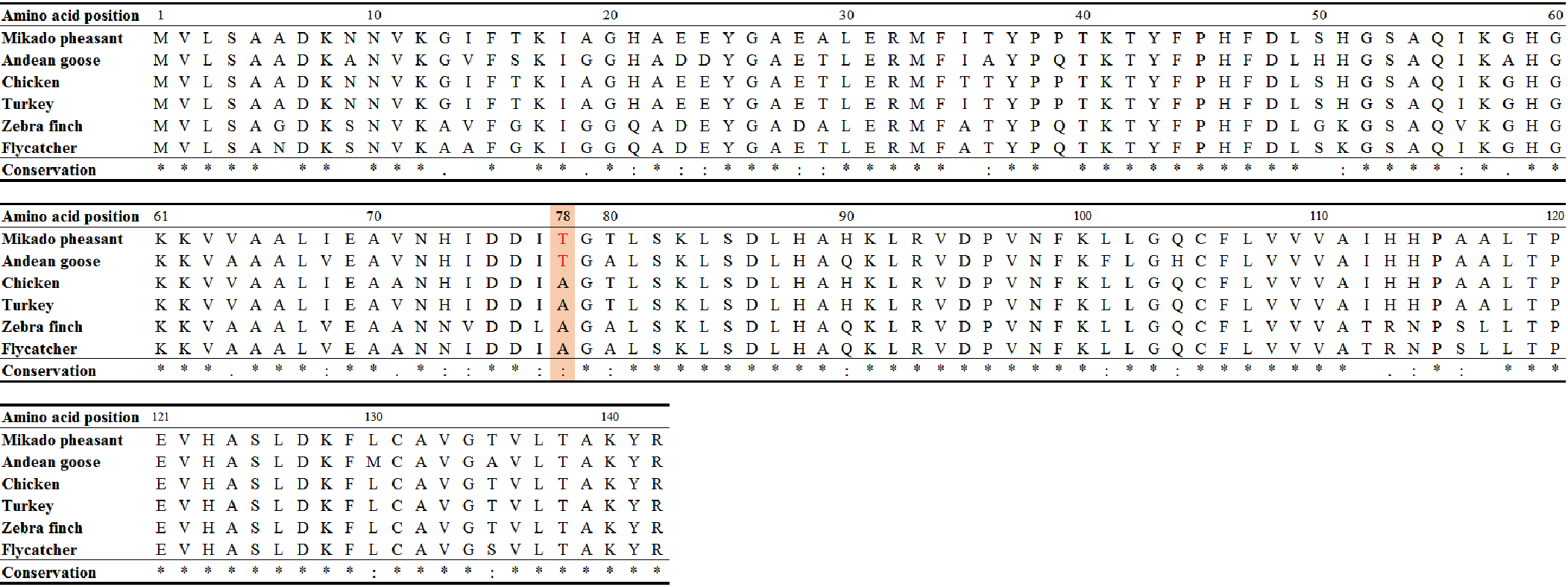


Figure S10: Amino acid comparison of the hemoglobin alpha-A subunit in 6 avian species. A unique substitution of alanine with threonine (colored in red) at residue 78 is highlighted. For representing the degree of conservation, an asterisk (*) indicates all residues are identical, a colon (:) indicates a conserved substitution, and a dot (.) indicates a semi-conserved substitution.


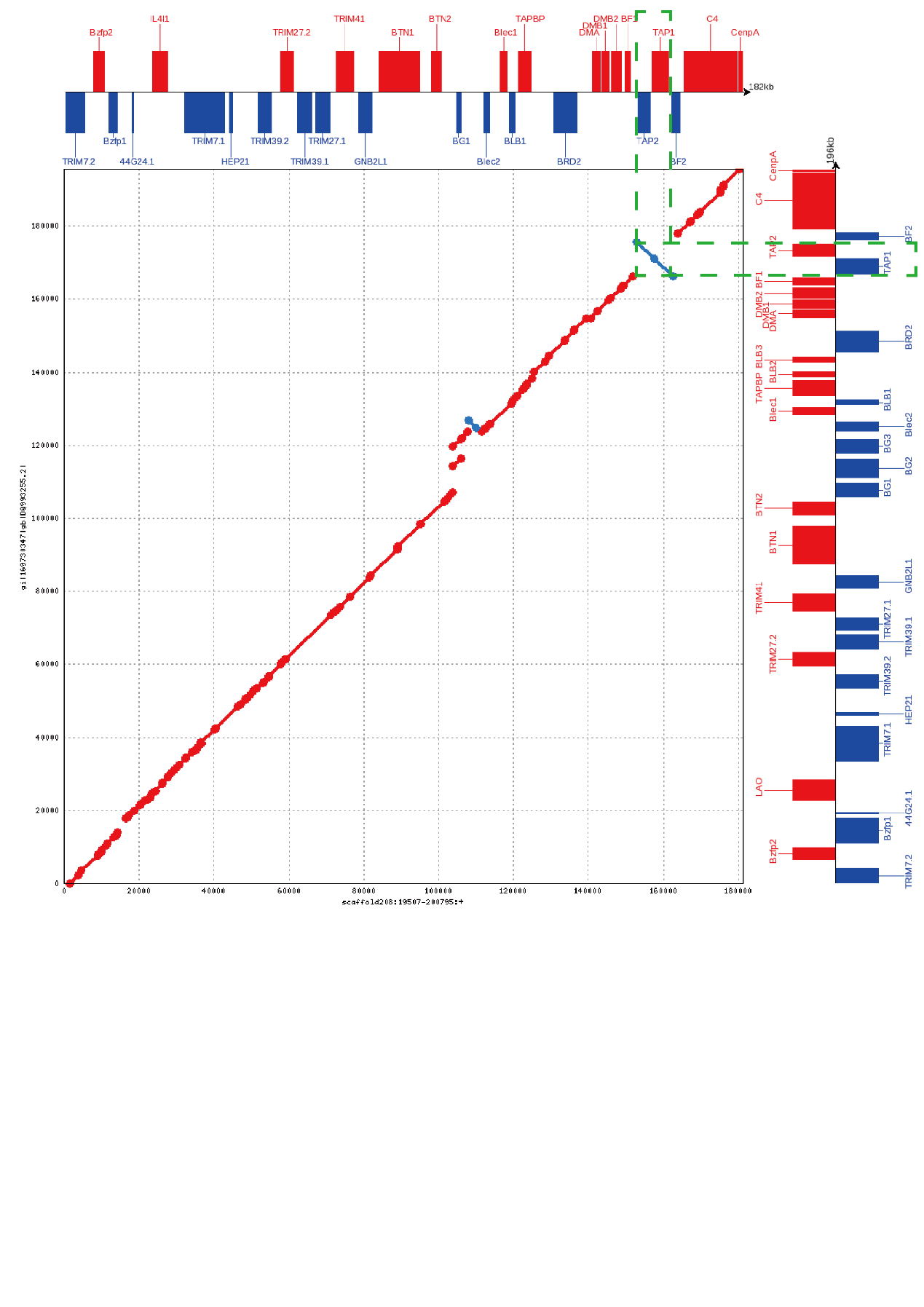


Figure S11: An identity plot of the MHC regions from the Mikado pheasant and the turkey. The turkey MHC sequence was downloaded from GenBank (DQ993255). Its nucleotide sequence from 1354 to 197 022 was aligned against the Mikado pheasant MHC sequence from 19 507 to 200 795 in scaffold208. The gene structure boxes on the horizontal and vertical axes, respectively, represent the gene loci in the Mikado pheasant and turkey. Boxes with different sizes exhibit different gene locus sizes, and red/blue coloring indicates genes in forward/reverse orientation. The red dots (or lines) on the diagonal indicate that the sequences were aligned in the same orientation, whereas the blue dots indicate alignments with reverse complements. The green dashed lines highlight the sequence of the inverted *TAP1*-*TAP2* block.


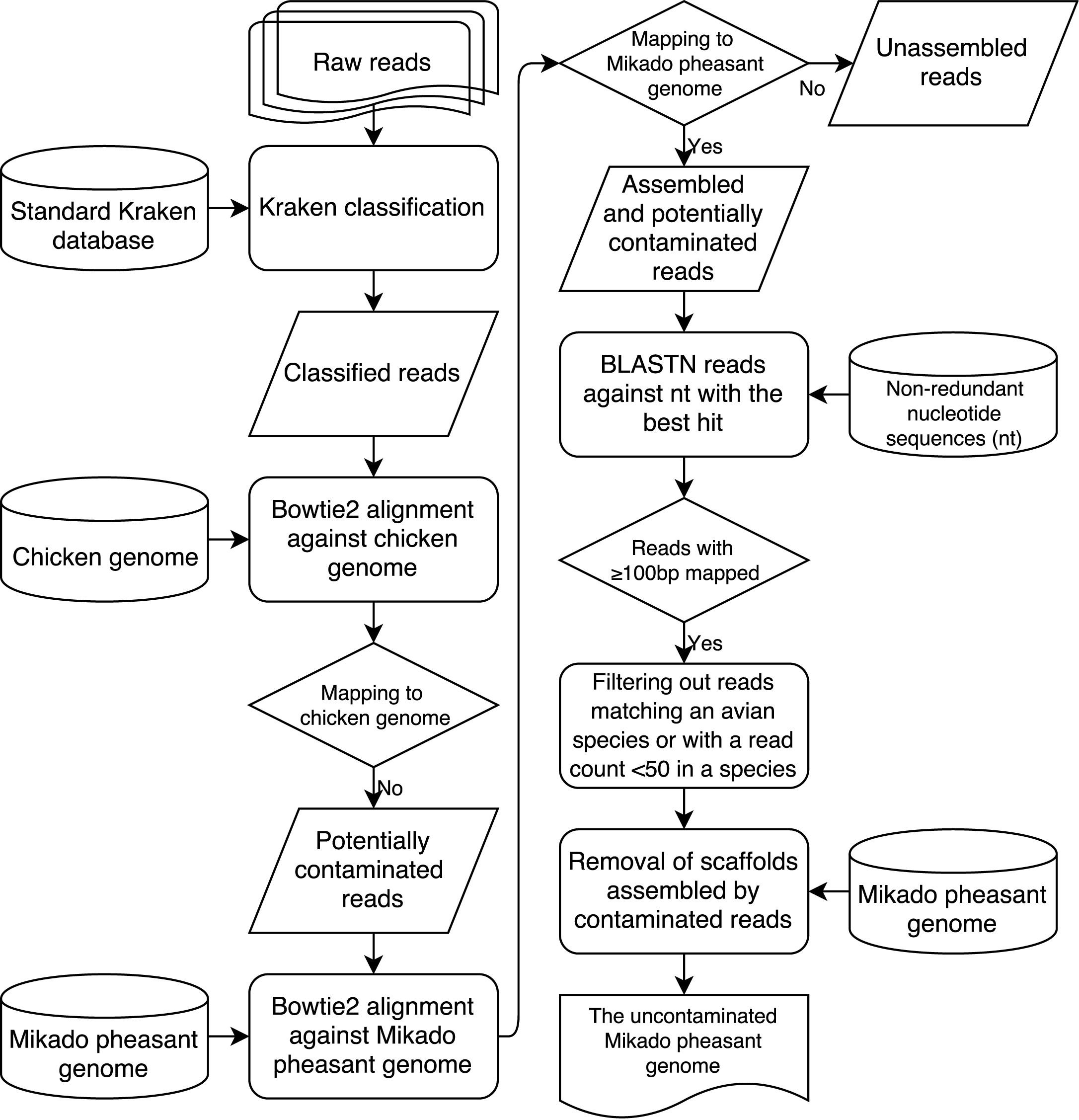


Figure S12: Workflow of the post-check for contamination in the Mikado pheasant genome.

# Supplementary Tables

| Table S1: Basic statistics of sequencing data collected from Mikado pheasant samples. | | | | | | | |
| --- | --- | --- | --- | --- | --- | --- | --- |
| **No.** | **Library Type** | | **Platform** | | **Read Length** | **Insert Size** | **Total Bases (Mb)** |
| **DNA-Seq** | | | | | | | |
| 1 | paired-end | HiSeq | | 151 | | 280 | 50 120 |
| 2 | paired-end | HiSeq | | 151 | | 480 | 48 665 |
| 3 | mate pair | HiSeq | | 101 | | 1000 | 17 315 |
| 4 | mate pair | HiSeq | | 101 | | 3000 | 13 984 |
| 5 | mate pair | HiSeq | | 101 | | 5000 | 15 084 |
| 6 | mate pair | HiSeq | | 101 | | 7000 | 12 912 |
| 7 | mate pair | HiSeq | | 101 | | 10 000 | 13 613 |
| **RNA-Seq** | | | | | | | |
| 8 | RF-first strand | HiScanSQ | | 101 | | 165 | 7622 |
| 9 | RF-first strand | HiSeq | | 35-151 | | 185 | 10 631 |

| Table S2: Statistics of repeated regions detected from the Mikado pheasant genome. | | | |
| --- | --- | --- | --- |
| Category | Count | Length Occupied | Percentage |
| SINEs | 4983 | 636 371 | 0.06% |
| Penelope | 114 | 23 506 | 0.00% |
| LINEs | 203 998 | 69 928 597 | 6.56% |
| LTR elements | 29 902 | 14 062 977 | 1.32% |
| hobo-Activator | 14 243 | 5 264 192 | 0.49% |
| Tc1-IS630-Pogo | 6336 | 3 029 793 | 0.28% |
| Tourist/Harbinger | 2999 | 276 170 | 0.03% |
| Total interspersed repeat | NA | 94 985 062 | 8.91% |
| Small RNA | 1845 | 233 508 | 0.02% |
| Satellites | 35 262 | 9 398 574 | 0.88% |
| Simple repeats | 331 044 | 14 807 256 | 1.39% |
| Low complexity | 56 491 | 2 986 686 | 0.28% |
| LINE: long interspersed nuclear element; LTR: long tandem repeat; SINE: short interspersed nuclear element. | | | |

| Table S3: Statistics of annotated genes in the Mikado pheasant compared with chicken, turkey, duck, and zebra finch. | | | | | |
| --- | --- | --- | --- | --- | --- |
| **Gene Statistics** | **Mikado pheasant** | **Chicken^*^** | **Turkey^*^** | **Duck^*^** | **Zebra finch^*^** |
| Gene count | 15 972 | 16 516 | 16 051 | 16 521 | 17 471 |
| Average gene length (kb) | 19.9 | 21.1 | 17.4 | 17.8 | 21.4 |
| Average CDS length (bp) | 1625 | 1433 | 1305 | 1317 | 1383 |
| Average exon length (bp) | 164.1 | 158.1 | 158.0 | 160.7 | 153.5 |
| Average intron length (bp) | 2053 | 2437 | 2215 | 2298 | 2493 |
| CDS: coding DNA sequence.  **^*^** Data from Zhang *et al*. (2014). | | | | | |

| Table S4: Summary of DNA read mapping rates. | | |
| --- | --- | --- |
|  | 280 bp Library | 480 bp Library |
| Mapped concordantly^*^ | 95.2% | 90.3% |
| Overall mapping rate^†^ | 97.5% | 96.9% |
| ^*^ Mapped concordantly means the read pairs were aligned to the genome with the expected distances and orientation.  ^†^ Overall mapping rate stands for the ratio of total mapped reads (in pairs or not) to total reads. | | |

| Table S5: Summary of RNA read mapping rates. | | |
| --- | --- | --- |
|  | RNA Sample 1 | RNA Sample 2 |
| Total mapped | 95.8% | 93.1% |
| Multiple Mapped | 2.04% | 2.04% |
| Uniquely Mapped | 93.8% | 91.1% |

| Table S6: BUSCO benchmark results. | | | | |
| --- | --- | --- | --- | --- |
|  | **Completely Found** | | **Partially Found** | **Missing** |
| *S. mikado* | | 2495 (82.53%) | 183 (6.06%) | 345 (11.41%) |
| *A. platyrhynchos* | | 2444 (80.85%) | 234 (7.74%) | 345 (11.41%) |
| *G. gallus* | | 2734 (90.44%) | 106 (3.51%) | 183 (6.05%) |
| *M. gallopavo* | | 2438 (80.65%) | 208 (6.88%) | 377 (12.47%) |
| *T. guttata* | | 2456 (81.24%) | 229 (7.58%) | 338 (11.18%) |
| BUSCO was performed in gene set/proteome mode with a total of 3023 orthologs in the Vertebrata lineage. The protein sequences of the Mikado pheasant (*S. mikado*) were converted from the predicted gene models. The protein sequences of the duck (*A. platyrhynchos*)*,* chicken (*G. gallus*)*,* turkey (*M. gallopavo*), and zebra finch (*T. guttata*) were retrieved from Ensembl. | | | | |

| Table S7. Alignment of the Mikado pheasant DNA assembly to chicken chromosomes. | | | | |
| --- | --- | --- | --- | --- |
| **Chicken chromosome** | **Count of aligned scaffold** | **Average identity (%)** | **Aligned coverage (%)** | **Chromosome length** |
| Chr1 | 36 | 88.77 | 83.10 | 195 276 750 |
| Chr2 | 17 | 88.73 | 85.70 | 148 809 762 |
| Chr3 | 17 | 88.73 | 89.00 | 110 447 801 |
| Chr4 | 12 | 88.55 | 88.20 | 90 216 835 |
| Chr5 | 6 | 88.74 | 88.50 | 59 580 361 |
| Chr6 | 5 | 88.62 | 89.50 | 34 951 654 |
| Chr7 | 5 | 88.88 | 91.20 | 36 245 040 |
| Chr8 | 5 | 89.04 | 91.10 | 28 767 244 |
| Chr9 | 4 | 88.97 | 92.20 | 23 441 680 |
| Chr10 | 6 | 89.51 | 92.00 | 19 911 089 |
| Chr11 | 4 | 89.52 | 92.30 | 19 401 079 |
| Chr12 | 4 | 89.37 | 90.70 | 19 897 011 |
| Chr13 | 5 | 89.13 | 90.20 | 17 760 035 |
| Chr14 | 3 | 89.10 | 88.50 | 15 161 805 |
| Chr15 | 1 | 89.44 | 92.30 | 12 656 803 |
| Chr16 | 4 | 86.24 | 35.30 | 535 270 |
| Chr17 | 3 | 89.69 | 90.10 | 10 454 150 |
| Chr18 | 3 | 89.43 | 85.20 | 11 219 875 |
| Chr19 | 5 | 89.69 | 90.40 | 9 983 394 |
| Chr20 | 3 | 89.36 | 87.40 | 14 302 601 |
| Chr21 | 3 | 89.59 | 91.70 | 6 802 778 |
| Chr22 | 11 | 89.45 | 90.50 | 4 081 097 |
| Chr23 | 1 | 89.19 | 89.60 | 5 723 239 |
| Chr24 | 1 | 89.98 | 93.00 | 6 323 281 |
| Chr25 | 10 | 88.43 | 59.40 | 2 191 139 |
| Chr26 | 5 | 88.94 | 86.30 | 5 329 985 |
| Chr27 | 6 | 88.74 | 71.10 | 5 209 285 |
| Chr28 | 5 | 88.88 | 80.10 | 4 742 627 |
| ChrW | 14 | 88.68 | 38.00 | 1 248 174 |
| ChrZ | 27 | 87.36 | 63.00 | 82 363 669 |

| Table S9: Gene Ontology categories enriched for contracted gene families in the Mikado pheasant. | | | | | | | |
| --- | --- | --- | --- | --- | --- | --- | --- |
| **GO accession** | **GO term** | **Ontology** | **Involved family number** | **Involved gene number in the family** | **Significant family number** | **Number of genes in the significant family** | **Empirical *P*** |
| **immune system process (GO:0002376) / response to stimulus (GO:0050896)** | | | | | | | |
| GO:0002504 | antigen processing and presentation of peptide or polysaccharide antigen via MHC class II | BP | 2 | 0 | 1 | 0 | 0.0143 |
| GO:0071346 | cellular response to interferon-gamma | BP | 15 | 26 | 1 | 1 | 0.0199 |
| GO:0042742 | defense response to bacterium | BP | 43 | 62 | 1 | 1 | 0.0380 |
| GO:0006955 | immune response | BP | 126 | 117 | 2 | 4 | 0.0490 |
| **apoptosis (GO:0006915)** | | | | | | | |
| GO:0008625 | extrinsic apoptotic signaling pathway via death domain receptors | BP | 17 | 22 | 1 | 1 | 0.0254 |
| GO:1902042 | negative regulation of extrinsic apoptotic signaling pathway via death domain receptors | BP | 18 | 20 | 1 | 1 | 0.0284 |
| GO:0006915 | apoptotic process | BP | 126 | 169 | 1 | 0 | 0.0497 |
| **Others** | | | | | | | |
| GO:1990393 | 3M complex | CC | 2 | 5 | 1 | 4 | 0.0130 |
| GO:0043056 | forward locomotion | BP | 2 | 4 | 1 | 0 | 0.0162 |
| GO:0060419 | heart growth | BP | 2 | 4 | 1 | 0 | 0.0162 |
| GO:0021591 | ventricular system development | BP | 16 | 26 | 1 | 2 | 0.0231 |
| GO:0090526 | regulation of gluconeogenesis involved in cellular glucose homeostasis | BP | 3 | 2 | 1 | 0 | 0.0253 |
| GO:0006278 | RNA-dependent DNA replication | BP | 3 | 3 | 1 | 0 | 0.0254 |
| GO:0003964 | RNA-directed DNA polymerase activity | MF | 3 | 3 | 1 | 0 | 0.0254 |
| GO:0033132 | negative regulation of glucokinase activity | BP | 3 | 3 | 1 | 1 | 0.0257 |
| GO:0006898 | receptor-mediated endocytosis | BP | 61 | 85 | 1 | 1 | 0.0260 |
| GO:0017148 | negative regulation of translation | BP | 38 | 51 | 1 | 1 | 0.0261 |
| GO:0055002 | striated muscle cell development | BP | 3 | 5 | 1 | 1 | 0.0286 |
| GO:0005516 | calmodulin binding | MF | 39 | 80 | 1 | 1 | 0.0286 |
| GO:0005044 | scavenger receptor activity | MF | 42 | 51 | 1 | 1 | 0.0347 |
| GO:0006457 | protein folding | BP | 93 | 101 | 1 | 1 | 0.0374 |
| GO:0007030 | Golgi organization | BP | 47 | 87 | 1 | 1 | 0.0476 |
| GO:0003007 | heart morphogenesis | BP | 47 | 86 | 1 | 1 | 0.0478 |
| GO:0042605 | peptide antigen binding | MF | 5 | 5 | 1 | 0 | 0.0482 |
| GO:0090502 | RNA phosphodiester bond hydrolysis, endonucleolytic | BP | 24 | 28 | 1 | 1 | 0.0496 |

Compared to the common ancestor of the Mikado pheasant and turkey. Ontology terms: Biological Process (BP); Cellular Component (CC); Molecular Function (MF).

| Table S12: KEGG pathways enriched for metabolism with positively selected genes of the Mikado pheasant. | | | | |
| --- | --- | --- | --- | --- |
| **Map ID** | **Term** | **Gene symbol** | ***P*-value** | **Adjusted *P*-value** |
| map00563 | Glycosylphosphatidylinositol (GPI)-anchor biosynthesis | *PIGK*, *PIGS*, *PIGV* | 9.20E-05 | 0.0054 |
| map00562 | Inositol phosphate metabolism | *INPP5A*, *INPP5J*, *PI4KB*, *PLCE1* | 1.10E-04 | 0.0064 |
| map04070 | Phosphatidylinositol signaling system | *INPP5A*, *INPP5J*, *PI4KB*, *PLCE1* | 0.0004 | 0.0230 |
| KEGG: Kyoto Encyclopedia of Genes and Genomes.  The Mikado pheasant (foreground branch) was compared to the chicken, turkey, duck, and zebra finch (background branches) by the branch-site model of PAML. | | | | |

| Table S15: KEGG pathways enriched for immune response with positively selected genes of the Mikado pheasant. | | | | |
| --- | --- | --- | --- | --- |
| **Map ID** | **Term** | **Gene symbol** | ***P*-value** | **Adjusted *P*-value** |
| map04630 | Jak-STAT signaling pathway | *BCL2, CCND3, IL12RB2, IL23R, IL7* | 4.40E-04 | 0.035 |
| KEGG: Kyoto Encyclopedia of Genes and Genomes.  The Mikado pheasant (foreground branch) was compared to the chicken, turkey, duck, and zebra finch (background branches) by the branch-site model of PAML. | | | | |

| Table S16: Gene annotation of the mitochondrial genome of the Mikado pheasant. | | | | | |
| --- | --- | --- | --- | --- | --- |
| **Name** | **Start** | **Stop** | **Strand** | **Length** | **Amino acid length** |
| Control Region | 1 | 1154 | + | 1154 |  |
| trnF(gaa) | 1155 | 1222 | + | 68 |  |
| rrnS | 1222 | 2189 | + | 968 |  |
| trnV(tac) | 2189 | 2261 | + | 73 |  |
| rrnL | 2265 | 3866 | + | 1602 |  |
| trnL2(taa) | 3867 | 3940 | + | 74 |  |
| nad1 | 3952 | 4926 | + | 975 | 324 |
| trnI(gat) | 4927 | 4998 | + | 72 |  |
| trnQ(ttg) | 5005 | 5075 | - | 71 |  |
| trnM(cat) | 5075 | 5143 | + | 69 |  |
| nad2 | 5144 | 6182 | + | 1039 | 346 |
| trnW(tca) | 6183 | 6260 | + | 78 |  |
| trnA(tgc) | 6267 | 6335 | - | 69 |  |
| trnN(gtt) | 6339 | 6411 | - | 73 |  |
| trnC(gca) | 6414 | 6481 | - | 68 |  |
| trnY(gta) | 6482 | 6551 | - | 70 |  |
| cox1 | 6553 | 8103 | + | 1551 | 516 |
| trnS2(tga) | 8095 | 8169 | - | 75 |  |
| trnD(gtc) | 8172 | 8240 | + | 69 |  |
| cox2 | 8242 | 8925 | + | 684 | 227 |
| trnK(ttt) | 8927 | 8994 | + | 68 |  |
| atp8 | 8996 | 9160 | + | 165 | 54 |
| atp6 | 9151 | 9834 | + | 684 | 227 |
| cox3 | 9834 | 10616 | + | 783 | 261 |
| trnG(tcc) | 10618 | 10686 | + | 69 |  |
| nad3 | 10687 | 11038 | + | 352 | 116 |
| trnR(tcg) | 11040 | 11108 | + | 69 |  |
| nad4l | 11109 | 11405 | + | 297 | 98 |
| nad4 | 11399 | 12776 | + | 1378 | 459 |
| trnH(gtg) | 12777 | 12845 | + | 69 |  |
| trnS1(gct) | 12846 | 12912 | + | 67 |  |
| trnL1(tag) | 12913 | 12983 | + | 71 |  |
| nad5 | 12984 | 14801 | + | 1818 | 604 |
| cob | 14801 | 15943 | + | 1143 | 380 |
| trnT(tgt) | 15946 | 16014 | + | 69 |  |
| trnP(tgg) | 16017 | 16085 | - | 69 |  |
| nad6 | 16090 | 16611 | - | 522 | 173 |
| trnE(ttc) | 16613 | 16680 | - | 68 |  |

| Table S17: Statistics of the Mikado pheasant assemblies using six *de novo* genome assembly software programs. | | | | | | |
| --- | --- | --- | --- | --- | --- | --- |
| Assembler | **MaSuRCA** | **ALLPATHS-LG** | **JR** | **Newbler** | **SGA** | **SOAPdenovo** |
| Total length | 1 035 950 077 | 961 089 354 | 1 020 323 050 | 1 012 802 775 | 1 032 902 386 | 957 903 436 |
| Maximum length | 50 275 205 | 10 761 509 | 9 135 257 | 27 421 239 | 43 707 223 | 12 868 164 |
| Number of Ns | 19 522 949 | 102 249 434 | 19 386 979 | 17 488 016 | 17 974 560 | 39 327 734 |
| Average length | 110 690 | 334 874 | 252 430 | 575 129 | 106 506 | 471 641 |
| N50 | 11 324 524 | 1 014 191 | 1 552 370 | 8 261 013 | 9 815 129 | 2 123 153 |
| Counts > 1 kb ^*^ | 9359 | 2870 | 4042 | 1761 | 9698 | 2031 |
| Counts > 5 kb | 1489 | 2131 | 2006 | 1083 | 1595 | 1500 |
| Counts > 10 kb | 928 | 1968 | 1748 | 923 | 1086 | 1260 |
| ^*^ The statistics considered only scaffolds longer than 1 kb in length. | | | | | | |
