# Supplementary material for: Whole-genome de novo sequencing reveals unique genes that contributed to the adaptive evolution of the Mikado pheasant
Source: Gigascience. 2018 May 2;7(5):giy044. doi: 10.1093/gigascience/giy044 (PMC5941149; doi:10.1093/gigascience/giy044)
Supplement: Response_to_Reviewer_Comments_Original_Submission.pdf [file giy044_response_to_reviewer_comments_original_submission.pdf]

Author's Response To Reviewer Comments

Close

Editor suggestions:

Please check for potential contamination, along the lines suggested by reviewer 2, and describe this step in the manuscript. Among other points, reviewer 1 also remarks that only mitochondrial data has been used for divergence time estimates - this should be discussed as a potential limitation in the revised manuscript.
Reviewer 3 has an important point regarding errors in the gal4 assembly - please carefully address this point, as it may affect your conclusions.

Response: Thank you for returning the constructive and useful comments from the three reviewers. Following the suggestion from Reviewer #2, contamination testing for sequencing reads was performed, and 31 contaminated scaffolds were identified and removed from the draft genome. Please refer to response 2-11 for the details. On Reviewer #1's suggestion, since nuclear genome data of the copper pheasant is not available now, we could only incorporate the five long-tailed pheasants into our analysis on the mitochondrial level. Discussion about the potential impact of this on the divergence time estimate has been added to the revised manuscript. Please refer to response 1-2 for the details. On Reviewer #3's point, instead of the chr16 sequence from the gal4 or gal5 genome references, we used the chicken MHC-B sequence in our manuscript. The sequence was obtained from GenBank (AB268588.1) and it was produced by Sanger sequencing technology. We apologize for only mentioning it in the legend of Figure 3, but not clearly noting it in the manuscript. The sentence has been rephrased in the revised manuscript to clarify this point. Also, a result identical to that of our previous analysis was obtained after we realigned the MHC region between the Mikado pheasant and chr16 of gal5 (NC_006103.4). Please refer to response 3-2 for the details. All these points are fully addressed, and they do not affect our conclusions.

Overall, the reviewers point out quite a large number of minor inaccuracies or places where further information is needed - please make an effort to address all of these points, as it will help to make your work more clear and more reproducible.

Response: We are grateful for the editor's and reviewers' helpful suggestions on our manuscript. All points have been fully addressed as described below.

Another minor issue: In the first paragraph of the abstract, you write "... adaptive behaviors of the Mikado pheasant .." - do you actually mean animal behaviour here? Or rather something like "patterns of adaptive evolution"?

Response: We apologize for the confusion and we have replaced all instances of "behavior" with "evolution" in the revised manuscript.

Please also clarify in the manuscript whether you had approval of an ethics committee and /or , if applicable, permission of wildlife authorities (if the bird was caught in the wild for this study?) for the animal research.

Response: We thank you for your reminding us of this requirement. We have added a statement on ethics approval and consent in the Declarations section.

Reviewer reports:

Reviewer #1: The manuscript entitled "Whole-Genome De Nova Sequencing Reveals Unique Genes that Contributed to the Adaptive Evolution of the Mikado Pheasant" utilizes the nuclear and mitochondrial genomes to identify genes related to adaptation and immunity. Furthermore, they use nuclear genome genes to reconstruct the phylogenetic position of the Mikado Pheasant among birds with sequenced genomes and estimate the divergence time using mitochondrial genomes of long-tailed pheasants. The manuscript is in very good shape and I have mostly minor comments (see below). Generally, I thought the Results section could be better presented, but the Discussion section was very well written and really brings the significance of these findings to light.

Response: We appreciate the favorable comments and have addressed each issue as described below.

Major comments

1-1: One critical issue I had with the results was the use of separate analyses for the identification of PSGs - 5 vs. 50 species. Can you remove one of these analyses? Or if you decide to retain both, I think a couple of statements about how many PSGs and GO terms overlap and a explanation for their use is required.

Response: We appreciate the suggestion and agree with the reviewer that the identification of PSGs from 50 avian species here may cause confusion. To emphasize the main purpose of the manuscript, we have removed the analysis of PSGs in 50 avian species, including the description of the branch model used for this analysis in the “Examination of genes under positive selection and enrichment analysis” paragraph in the Materials and Methods section, from the revised manuscript.

1-2: Also, for the divergence time estimate using mitochondrial genomes - Is there any nuclear genome data for the other long-tailed pheasants that can be incorporated into this analysis? If not, please discuss how the use of only mitochondrial data may affect the divergence time estimates.

Response: We appreciate the suggestion. Currently, there is no nuclear genome data available for the copper pheasant, so unfortunately, incorporating all five long-tailed pheasants into our analysis using nuclear genomes is impossible at present. For the other four pheasants, however, Wang N. et al. [1] used six nuclear intron and two mitochondrial gene sequences to construct a phylogenetic tree, and its topology was consistent with our result. Our estimate of the divergence time was more precise, considering that we employed complete mitochondrial genomes in the reconstruction of a high-resolution tree for the Syrmaticus genus instead of a few mitochondrial genes. Our estimated divergence time is also supported by the paleogeographical report of Taiwan island formation. Despite these corroborations of the proposed tree topology and estimated divergence time, the use of only mitochondrial data may be considered as a potential limitation. Going forward, it will be necessary to analyze the nuclear genome to obtain further insights into the evolution history of long-tailed pheasants. We have added the paragraph above into the Discussion section.

Minor comments

Abstract

1-3: Line 78: The second sentence in abstract Background is oddly worded. Please revise. A variant of this sentence is also in Background (line 117).

Response: We apologize for the confusion. We have rephrased the sentence in the abstract section as: “This pheasant provides an opportunity to investigate evolutionary processes following geographic isolation.” and the sentence in the background section as: “The Mikado pheasant possesses ideal characteristics for evolutionary research because of its flightlessness and habitat isolation.”

1-4: Line 89: "mitochondrial genome was further" would sound better written as "mitochondrial genome was ALSO"

Response: The correction was made.

Background

1-5: Lines 112-116: I think that genome resources for endangered species can provide great insight into effective population size. This should be added here.

Response: We appreciate the suggestion, and the suggested text has been added.

1-6: Line 119: "the Syrmaticus genus and belongs" should be written as "the Syrmaticus genus and FORMS (or comprises)"

Response: The correction was made.

1-7: Line 131: change to "insights into its adaptive mechanisms." Remove "of the pheasant".

Response: The correction was made.

Results

1-8: Lines 233-234: "between 21.4 and 28.9 million years ago" The Figure has different values - 18.3-27.9. Is the text or figure correct?

Response: We apologize for the confusion. The age between 21.4 and 28.9 million years ago indicates the divergence time of the Phasianidae lineage including 3 birds, Mikado pheasant, turkey, and chicken. The values (18.3-27.9 Mya) in Figure 2 in the revised manuscript represents 95% confidence intervals of the divergence time between the Mikado pheasant and turkey. To avoid the confusion, the sentence was revised to “The estimated time of the Mikado pheasant-turkey divergence was 21.4 million years ago (Mya); the divergence time between chicken and the sister clade of the Mikado pheasant-turkey was estimated at 28.9 Mya.”

1-9: Lines 262-265: Please rewrite this first sentence as it is awkward.

Response: We apologize for the confusion. We have rephrased the sentence as: “To detect the genes that evolved rapidly due to positive selection under the influence of high elevation (Mikado pheasant) as opposed to low elevation (chicken, turkey, duck, and zebra finch), …”

1-10: Line 266: How many PSGs were identified? Please list the number in the text.

Response: We apologize for the confusion. There were 889 PSGs identified and enriched in GO functions. We have added the number in the text.

1-11: Lines 270-272: It should be noted here that PSGs enriched for metabolism constituted the highest number of PSGs as that information is buried in the supplemental files.

Response: A correction was made.

1-12: Lines 327-329, first sentence. The use of "Recently" seems odd as there has been significant work looking at dN/dS ratios in relationship to MHC. Please consider adding more references here and removing "Recently".

Response: We appreciate the suggestion. A correction was made and two references from Harmit Malik's work have been added to strengthen this point.

Reviewer #2: In their study titled "Whole-Genome De Novo Sequencing Reveals Unique Genes that Contributed to the Adaptive Evolution of the Mikado Pheasant", Lee, Hsieh et al. describe a newly sequenced bird genome - which is always a good resource - including some comparative genomics studies. I believe that this work is solid and clearly explained, and as such is of interest and in the scope of GigaScience. I do have some (mostly minor) comments detailed below that I believe would increase the quality and clarity of the manuscript.

Response: We appreciate the favorable comments.

GENERAL COMMENTS:

2-1: - The use of the word "behavior" (adaptive behavior) is misleading. It seems that the authors mean it in the context of adaptive evolutionary history, and I would suggest to reformulate for accuracy (Abstract and Introduction p5 l129).

Response: We apologize for the confusion and have changed the word “behavior” to “evolution” both in the abstract and the introduction (background).

2-2: - some figures could be improved by having more information on the figure instead of in the legend (mostly Figure 3, see detailed comments below)
2-3: - The significance of some data could be improved at a few locations (see detailed comments below)

Responses to 2-2 & 2-3: We appreciate these suggestions and have addressed each issue as described

below.

DETAILED COMMENTS:

ABSTRACT:
2-4: - The authors emphasize in the abstract the details of their data about the MHC & comparison with chicken: having less details but more scope / significance would improve the abstract.

Response: We appreciate the suggestion and have rephrased the sentence as: "To investigate the molecular evolution of the major histocompatibility complex (MHC) across several avian species, 39 putative genes spanning 227 kb on a contiguous region were annotated and manually curated. The MHC loci of the pheasant revealed a high level of synteny, several rapidly evolving genes, and inverse regions compared to the same loci in the chicken."

INTRODUCTION:
2-5: - p4 l114: what does "behavioral attributes" means here?

Response: We apologize for the confusion. According to the reference paper, Diana Le Duc et al. reported a nocturnal lifestyle in kiwi [2]. We believe that this is an example of a behavioral attribute successfully identified by the genome assembly approach.

2-6: - p4 l114-117: consider splitting this sentence.

Response: We appreciate the suggestion and have made the correction.

2-7: - p5 l1: how was hypoxic stress observed? Is there any citation? Or is this an expectation/extrapolation?

Response: We apologize for the confusion, and this is our expectation/extrapolation. We have removed the word "is" and replaced it with "may be."

2-8: - p5 l138-141: same comment as abstract.

Response: The correction was made.

DATA DESCRIPTION:
2-9: - Refer to the Method section at least one time at the beginning of this section.

Response: We appreciate the suggestion and have added the following descriptive text at the beginning of the Data Description section.
'The details about sample collection, library construction, sequencing, assembly, gene prediction, and annotation can be found in the "Materials and Methods" section.'

2-10: - p6 l156-158: please revise the formulation of this sentence for clarity. Fig S4 shows that there are in fact a lot of scaffolds with short length, even if indeed most of the genome size is assembled in large scaffolds.

Response: We apologize for the confusion. We have rephrased the sentence as: "… showed that most of the draft genome consisted of large scaffolds; though many short scaffolds were present, they only contributed a small portion of the genome size."

RESULTS:
2-11: - p7 l 176-180: was there a step to verify that the sequencing samples were not contaminated? For example, the bald eagle genome assembly (file from Zhang et al. (2014), Science) has hundreds of bacterial contigs in it (absent from the refseq version because very short), coming from 2 samples contaminated with Yersinia (SRR1176808 and SRR1176809). This can be checked quickly with some software such as Kraken or Taxonomer (with www.taxonomer.com - note that for this website, for a bird genome reads would be nearly all unknown or ambiguous). I could not find the data on the SRA at the time of reviewing to look myself.

Response: We appreciate the suggestion. We used Kraken, combining the approaches of aligning reads against both the chicken genome and the assembled genome, as well as alignment against BLAST's non-redundant nucleotide sequences (NT) database, to perform the post-check for contamination in our assembled genome (Fig. R1 below; S12 in the revised manuscript). In this way, we obtained 31 contaminated scaffolds with 12 587 bp (~0.001% of the total length) including 290 649 (0.088% of total reads) and 300 871 (0.095% of total reads) reads in the 280-bp and 480-bp libraries, respectively. The major contaminating species were phiX174 and E. coli. We then removed these 31 contaminated scaffolds and a related gene, which had neither an annotation nor a classified gene family. Thus, our conclusions in this study were robust and they were not affected by the contamination problem. We have added a new paragraph to describe the details at the end of the "De novo genome assembly" paragraph in the Materials and Methods section.
'To examine sequencing reads for potential contamination, we used Kraken (version 1.0) [78] with the standard Kraken database to check the paired-end DNA libraries. Classified reads reported by Kraken were further examined using our proposed pipeline (Additional file 1: Fig. S12). Briefly, we employed Bowtie 2 (version 2.3.0) [79] to align these classified reads against the chicken genome reference (Galgal 5.0) downloaded from Ensembl (release 90), collecting unmapped reads and using Bowtie 2 again to align them against the assembled genome of the Mikado pheasant. We then took those reads mapped onto the Mikado pheasant genome and performed BLASTN alignment against the non-redundant nucleotide sequences (NT) database, downloaded from NCBI's FTP site (on Nov. 16, 2017), using parameters "-outfmt '6 std staxids' -max_target_seqs 1 -evalue 1E-10." Next, we collected reads with alignment length ≥100 bp (i.e., two thirds of read length), filtering out the reads matching an avian species or with a read count <50 in a species. The remaining reads were counted and the contaminated scaffolds calculated by applying a cutoff of a read count >20 on a given scaffold. Finally, we removed 31 contaminated scaffolds with 12 587 bp (~0.001% of the total length) from the assembled genome.'

2-12: - p7 l180-184: unless reads were excluded when mapping at multiple locations, do (some) high coverage regions correspond to repeats?

Response: We appreciate the question. To minimize the effect of repeat sequences, we performed Bowtie 2 alignment with the best alignment of each read to calculate the per-base alignment coverage. (Please refer to response 2-41 for more details.)

2-13: - p8 l202: This sentence would be more clear if "with pheasant scaffolds" was added after "The

identities of each chicken chromosome"

Response: We appreciate the suggestion, and the sentence has been rephrased as: “The identities of each chicken chromosome with the scaffolds of Mikado pheasant …”

2-14: - p8 l208: if this is notable, what is the significance?

Response: Intrachromosomal inversions occur frequently within avian genomes [3-5]. Despite the concrete mechanism being unclear, inversion is thought to play an important role in avian genome evolution, serving as a driver of speciation [6, 7]. For example, a recent study reported that some intrachromosomal inversions in the white-throated sparrow were related to behavioral attributes and feather features [8]. Our manuscript describes the first genome-wide analysis to identify multiple intrachromosomal inversions between the Mikado pheasant and chicken genomes.

2-15: - p8 l217: as expected?

Response: Yes, according to the phylogenetic tree (Fig. 2 in the revised manuscript) and molecular evidence from recent reports [9, 10], the Mikado pheasant is more closely related to the Galliformes order than to the Passeriformes order. Therefore, we can expect that the Mikado pheasant scaffolds were poorly aligned with the zebra finch genome.

2-16: - p8 l218-221: consider having the mention of "high frequency of potentially highly conserved regions" before the "but", to contrast conservation and dynamics.

Response: We appreciate the suggestion, and the sentence has been rephrased as: “In general, the Mikado scaffolds displayed high conservation with the genomes of chicken and turkey. We also observed several intrachromosomal inversions and chromosomal translocations. This is the first genome-wide analysis to identify multiple intrachromosomal inversions between the Mikado pheasant and chicken genomes.”

2-17: - p8 l 220: what are the "high frequency" numbers? How does this compare to the literature, if any similar other research?

Response: We apologize for the confusion. Originally, the “high frequency” denoted that the Mikado genome showed high conservation with the genomes of chicken and turkey. To avoid the confusion, we have rephrased the sentence as described above (please refer to response 2-16).

2-18: - p9 l229-230: The formulation here is confusing and should be revised to illustrate better that the 18 220 gene families (as mentioned in the legend of Figure 2) are for all species considered (and not just the Mikado pheasant) - since Figure S8 shows different numers. Additionally, the number of genes is lower than the number of annotated genes mentioned in the manuscript or than the one in Figure S8; why these three different values?

Response: We apologize for the confusion. The sentence has been rephrased to match the legend of Fig. 2. There are two possible reasons for the different gene numbers from these analyses in the Mikado pheasant. First, the gene families from 10 species (Fig. 2 in the revised manuscript) or 5 birds (Fig. S8) were classified by OrthoMCL using the protein sequences from Ensembl. Considering the phylogenetic

relationship, the E-value cutoff for running all-vs-all BLASTP was stricter in the analysis of 5 birds (1e-20) than in that of 10 species (1e-5) (please refer to Gene families in the Materials and Methods section). Thus, the number of genes in the Mikado pheasant was less in the analysis of 5 birds (14 375 genes) than in the 10 species (15 161 genes). Second, we used a completely different source—the Aves and Reptilians protein sequences from the NCBI NR database—to annotate 15 972 genes in the Mikado pheasant. Although these methods produced different numbers of genes, we believe that the numbers are in a reasonable range for the avian genome, based on a previous study [11].

2-19: - p9 l245: are fragmented annotations a possible issue here? i.e. are longer genes enriched or not in expanded families?

Response: Yes, we believe that fragmented annotations of longer genes may cause the evolutionary rates of expanded families to be overestimated. To reduce the potential errors, we used CAFE 3 to identify expanded and contracted genes in the study (see Gene families in the Materials and Methods section). The authors of CAFE 3 claim that they applied phylogenetic tree information to model the observed family sizes in the algorithm, which could recover accurate evolutionary rates of gene families with fragmented annotations [12].

2-20: - p9 l246: Are the numbers / rates surprising or not based on the literature?

Response: We did not expect so many gene ontology (GO) categories to be identified. However, the identified GO functions provided straightforward evidence to explain the Mikado pheasant's adaptation to high altitude.

2-21: - p9 l248-259: what about the ones in the chicken for example? And other birds?

Response: Analyzing genes with expansion and contraction is an approach to identify the gene number changes in each gene family. To infer these changes for a specific combination of interest, for example the Mikado pheasant versus chicken, would be ill-advised, due to the limitations of the statistical test provided by CAFE 3. The expansion and contraction can only be identified significantly between a specific species and its common ancestor. Based on the tree topology in Figure 2 in the revised manuscript, for example, chicken can only be compared with the node (labeled 28.9 Mya) which is the common ancestor of chicken and the other node (labeled 21.4 Mya; the common ancestor of the Mikado pheasant and turkey)—neither the Mikado pheasant, nor the turkey itself. For this reason, we cannot directly identify expanded/contracted genes between the Mikado pheasant and chicken/other birds.

2-22: - p9 l258: is 8/75 surprising? What is the fraction of all olfactory receptors among all gene families? Were there more olfactory receptors annotated in the pheasant than other birds? E.g. discuss based on the data from Steiger et al 2008 (DOI: 10.1098/rspb.2008.0607), or other literature if any.

Response: 1) Among the 75 expanded gene families of Mikado pheasant, 8 gene families were annotated as olfactory receptors (ORs). Since the proportion exceeds ten percent, we mentioned this finding in the manuscript to provide the result as a numeric basis for possible comparisons in future studies.
2) There were 12 549 gene families in the Mikado pheasant (total 18 220 gene families in the 10 species). Of these gene families, 44 were OR-related (with 65 genes predicted to be ORs).

3) Steiger et al. compared nine bird species from seven orders (blue tit, black coucal, brown kiwi, canary, galah, red jungle fowl, kakapo, mallard, and snow petrel) and drew the conclusion that the estimated total number of OR genes correlates positively with olfactory capability. However, some of the birds did not have an assembled draft genome at the time of the paper's publication, and some of the OR gene numbers might be overestimated by the authors. For instance, the paper displayed 600 estimated OR genes in the brown kiwi, but there were only 141 presented (82 OR genes were identified from the initial prediction) in a subsequent study when the genome sequence was available [2]. Despite these limitations, we can still compare our result with the kiwi [2]. There were more genes predicted to be ORs in the kiwi (N=82) than in the Mikado pheasant (N=65). However, this difference should not be overinterpreted, since ORs are highly duplicated across the genome, which may produce more overcollapsed contigs during the assembly process. This is a general problem in the short-read sequencing technology.

2-23: - p10 l262: this formulation is unclear: "because of living at and between high and low elevation".

Response: We apologize for the confusion. We have removed the unclear sentence and rephrased as:
"To detect the genes that evolve rapidly due to positive selection under the influence of high elevation (Mikado pheasant) as opposed to low elevation (chicken, turkey, duck, and zebra finch), …"

2-24: - p10 l264: Since these 7132 orthologues seem to be the same as the 7132 single-gene families mentioned in Methods, the change of terminology (gene family v.s. orthologs) is confusing (maybe use orthologs for single-gene families that were also annotated as orthologs by OrthoMCL, and gene families for the others?).

Response: We apologize for the confusion. Gene families contain orthologs and paralogs. Orthologs (or orthologous genes) indicate genes with similar sequences in different species, whereas paralogs (not part of this study) indicate genes with similar sequences from within the same species. Specifically, orthologs from within a gene family having one gene for each species are called single-copy orthologs (or single-gene families). In the manuscript, we classified gene families using OrthoMCL, and further identified single-copy orthologs from these gene families to construct a phylogenetic tree and analyze positively selected genes. To avoid confusion, we have unified the terminology and used "gene families" and "orthologs" in the revised manuscript.

2-25: - p11 l293: since the Jak-STAT pathway is not mentioned again in discussion, please add why this is worth noticing.

Response: We appreciate the suggestion. The following sentence for discussing the Jak-STAT pathway has been added in the Discussion section.
"Some of these PSGs were also involved in the Jak-STAT signaling pathway (Additional file 1: Table S15), which participates in chemical signal transmission and induces cellular stress responses, such as immunity, apoptosis, [61, 62], and hypoxia [63]. All these results provide wider support for the adaptive evolution of the Mikado pheasant."

2-26: - p11 l301: this number of 5287 orthologs between 48 birds is identical to the one of orthologs identified in 10 species (with mammals) - please check that this is accurate.

Response: We apologize for the mistake. The correct number of orthologs is 2209.

2-27: - p12 l305: the ubiquitin activity is not mentioned in discussion: what would be the significance of having expanded gene families associated with this GO term?

Response: The GO term associated with ubiquitin activity is associated with the degradation of proteins. The ubiquitin will mark the target protein by forming an isopeptide bond to the lysine residues on the protein. The complex will be sent to the proteasome, and the proteins will be subsequently degraded. Currently, few studies have reported the relationship between ubiquitin activity and phenotype in avian species. Thus, we have insufficient evidence to explain the enrichment of ubiquitin activity in the Mikado pheasant. To emphasize the main purpose of the manuscript, in response to Reviewer #1's suggestion, we have removed the results of the analysis of positively selected genes in 50 avian species from the Results section.

2-28: - p12 l320: Methods says MAKER, not manual curation; was MAKER used and then the annotations manually curated?

Response: Yes, MAKER was used to predict potential MHC-B genes, and then these genes were manually curated. We apologize for the confusion and have added a new paragraph to describe the details at the end of the "Gene prediction and annotation" paragraph in the Materials and Methods section.
"For MHC-B annotation and curation, we first took the scaffold208 sequence and used MAKER (version 2.31.8) [88] to predict the potential gene structures of MHC-B genes. Next, the RNA-Seq libraries from the Mikado pheasant and the homologous protein sequences from chicken and turkey were aligned to these predicted regions. Finally, we used Web Apollo (version 2.0.3), a web-based and visualization tool for curation and annotation, to manually curate these genes according to the alignment evidence."

2-29: - p12 l329: there is more general literature on this question (e.g. Harmit Malik's work and others); adding one or two references would strengthen this point.

Response: We appreciate the suggestion. The two references from Harmit Malik's work have been added to strengthen this point.

2-30: - p13 l330: BLB2 is mentioned here (probably because found in RNAseq data?), but it is is missing from the Figure and afterwards said missing from the Mikado pheasant assembly, which is confusing. Maybe the lines 445 to 451 should be part of this result section instead?

Response: We appreciate the suggestion. We have moved this part to the Results section.

2-31: - p13 l331: see comment about Figure 3

Response: A correction was made (please refer to response 2-48).

2-32: - p13 l240: significance of inversions?

Response: Yes, there are several MHC-related studies reporting that, in the Galliformes order, the TAPBP and/or TAP1-TAP2 blocks are in inverse orientation [13-15]. Wang B. et al. even proposed a hypothesis of MHC evolutionary history in black grouse based on these inversions [16]. In our study, it is the first time to observe them in the Mikado pheasant, and we believe that this finding will have a profound

influence on studies of the evolutionary history of the avian MHC.

DISCUSSION:
2-33: - p14 l375: since these extra steps are not detailed in the Method section, the parameters and versions should figure in Table S18 or in additional info.

We apologize for the unclear statement. We have rephrased the paragraph in the Materials and Methods section as follows.
"The quality of the raw reads was examined using FastQC (version 0.10.1). Trimmomatic (version 0.30; parameters: "ILLUMINACLIP:TruSeq3-PE.fa:2:30:15 SLIDINGWINDOW:4:20 MINLEN:100") [76] and NextClip (version 1.3.1) [77] with default parameters were used to trim sequencing reads. Genome assembly into contigs was performed by MaSuRCA (version 2.3.2) [15] with settings based on the instruction manual. ALLPATHS-LG (version 49722) [43], Newbler (version 2.9) [45] both with default parameters, JR (version 1.0.4; parameters: "-minOverlap 60 -maxOverlap 90 -ratio 0.3") [44], SGA (version 0.10.13; parameters: "assemble -m 125 -d 0.4 -g 0.1 -r 10 -l 200") [46], and SOAPdenovo (version 2.04; parameters: "-K 47 -R") [47] were also used to assemble contigs. We employed SSPACE (version 3.0; parameter: "-z 300") [74] to construct scaffolds for the draft genome. In this step, mate pair libraries with 35 bases from the 5'end of both reads were used for scaffolding. Scaffold sequences shorter than 300 bp were then excluded from the final assembly. The statistical results of the assembly were estimated using QUAST (version 3.2) [75]."

2-34: - p15 l387: how were the number of misassembled or fragmented sequences estimated and distinguished from real differences with the chicken genome (since Fig1 is referred to)?

Response: We apologize for the carelessness. We realize that it is difficult to estimate the number of misassembled or fragmented sequences from the information in Fig. 1 only. The degree of fragmented sequences may be distinguished by the composition of lines and points from Fig. 1A; on the other hand, we can expect that the more points on a syntenic map, the more fragmented sequences exist. To clarify the statement, we have rephrased the sentence as: "Although scaffolds of the draft genome displayed some degree of fragmentation (Fig. 1A) and showed translocation (Fig. 1B) in certain chicken chromosomes, …"

2-35: - p17 l438-441: is there any evidence that these inversions affect the expression of these genes…?

Response: No, there is no study reporting a correlation between these inversions and their gene expression.

2-36: - p17 l445-451: see comment for p13 l330.

Response: A correction was made (please refer to response 2-30).

2-37: - p17 l452: "the whole genome of a genus" would read better as "the whole genome of a bird of the genus"

Response: A correction was made.

MATERIAL AND METHODS:

2-38: - p19 l486: were both experiments done on pooled RNA from the 2 males, or was there one male per RNAseq experiment?

Response: We apologize for the confusion. There was one male individual per RNA-Seq experiment.

2-39: - p19 l494: FastQC version and exact tools and parameters used to trim reads and remove adapters?

Response: FastQC version is 0.10.1. The paired-end and mate pair reads were trimmed and adapters removed by Trimmomatic (version 0.30; parameters: "ILLUMINACLIP:TruSeq3-PE.fa:2:30:15 SLIDINGWINDOW:4:20 MINLEN:100") and NextClip (version 1.3.1) with default parameters, respectively. This information is included in the revised text.

2-40: - p19 l496: MaSuRCA reference missing here (even if elsewhere in the ms): Zimin, A. et al. Bioinformatics (2013). doi:10.1093/bioinformatics/btt476

Response: We appreciate the information and a correction was made.

2-41: - p20 l509: were the software's default parameters used? What were the parameters regarding non uniquely mapping reads?

Response: Yes, default parameters were used for both Bowtie 2 and TopHat2. When evaluating per-base alignment coverage and mapping rates for DNA reads, only the best alignment for each read was taken into account. However, when evaluating mapping rates for RNA reads, the non-uniquely mapped reads were considered by TopHat2. Table R1 shows the detailed information for the multi-read alignment. In addition, to improve RNA-Seq mapping rates at Reviewer #3's suggestion, we have replaced Table S5 in Additional file 1 with the results using the STAR alignment program in the revised manuscript.

Table R1: Summary of RNA read mapping rates using TopHat2.

	RNA Sample 1	RNA Sample 2
Reads		
Overall mapping rate (left)†	92.5%	84.4%
Multiple mapping rate (left)	6.6%	7.5%
Overall mapping rate (right)†	91.4%	77.3%
Multiple mapping rate (right)	6.6%	7.6%
Pairs		
Concordant mapping rate*	88.1%	72.4%
Multiple mapping rate	6.7%	7.7%

† Overall mapping rate stands for the ratio of total mapped reads to total reads.
* Mapped concordantly means the read pairs were aligned to the genome with the expected distances and orientation.

2-42: - p10 l513: Version of BEDTools is missing.

Response: The BEDTools version was 2.23.0. This information is included in the revised text.

2-43: - p21 l533: RepeatMasker version, parameters and library used are missing.

Response: We used RepeatMasker (version 4.0.5, parameter: "-species chicken"), including rmblastn (version 2.2.23+) as the search engine, RepBase (version 20140131), and RM database (version 20140131), to identify repeat regions. This information is included in the revised text.

2-44: - p22 l565: consider adding the numbers of genes and gene families identified that are not single genes before switching to the Method of ortholog identification.

Response: We appreciate the suggestion. We have added gene and gene family numbers in the sentence as follows.
"Then, 18 220 gene families (including 5287 single-copy orthologs) were obtained from the 10 species, and 13 436 gene families (including 7132 single-copy orthologs) were obtained from the 5 birds by OrthoMCL (version 2.0.9) using default parameters. In the analysis of the 10 different species, 15 161 genes of the Mikado pheasant were grouped into 12 549 gene families. In the analysis of the 5 avian species, 14 375 Mikado pheasant genes were grouped into 12 078 gene families."

2-45: - p22 l573: bootstraps?

Response: We performed RAxML with 500 bootstrap replicates. This information is included in the revised text.

FIGURES:
FIGURE 1B:
2-46: - point/label differently on the figure scaffolds 1 and 45 (since mentioned in the text), and maybe also the ones that fully align to chicken chromosomes?

Response: We appreciate the suggestion. We have added arrows colored in yellow to indicate scaffolds that fully aligned to the chicken chromosomes; grey arrows are added to point out the multiple alignment ones.

FIGURE2:
2-47: - consider adding numbers per My, to facilitate the comparison between branches.

Response: A correction was made.

FIGURE3:
2-48: - it would help the reader a lot if instead of having a color code for forward/reverse (that information is already coded by the position above or under the bar), the genes could be color coded based on their dN/dS ratios.

Response: We appreciate the suggestion. A dN/dS ratio bar chart has been added under the gene structure boxes to enhance the readability of Figure 3. At the same time, the forward/reverse color codes are retained, since it clearly visualized the gene orientation.

2-49: - the coverage scale does not allow to see lower coverage genes; consider using a log scale?

Response: A correction was made.

SUPP DATA:
2-50: FIGURE S2: does 'habitats available' correspond to where they are found generally, or a protected habitat?

Response: The primary habitats available for the Mikado pheasant include both areas where they are generally found and protected habitats. Regions in national parks are protected habitats, but Shuanggueihu and Tawushan are areas where they are generally found.

2-51: FIGURE S8: see comment for Results p9 l230.

Response: Values in the figure are correct. Please refer to responses 2-18 and 2-44 for more details.

2-52: TABLE S1: which RNAseq was HiSeq and which was HiSanSQ?

Response: We apologize for the confusion and we have added platform information in Table S1.

2-53: TABLE S9: for ex. for the GO:0002504 line, could these annotations be missing from the assembly or be fragmented? Since there are only 2 genes in this family, it sounds possible.

Response: We apologize for the confusion. There were 2 gene families involved in the GO:0002504 function rather than 2 genes in this family. These gene families contained genes in other species instead of the Mikado pheasant. One of these gene families showed significant change in its size, which met our expectation of gene contraction in the Mikado pheasant.

2-54: TABLE S18: see comment about Discussion p14 l375.

Response: We have added detailed steps, parameters, and versions in the Materials and Methods section, so the table is not changed (please refer to response 2-33).

Reviewer #3: The manuscript presents the genome and gene annotation of the Mikado pheasant (MP), a protected species living in geographical isolation and adapted to high altitude habitats. The genome was assemble into 208.8k contigs (>300 bp) and 9,359 scaffolds (>1 kb) using Illumina short read technology of paired-end and mate-pair libraries. Annotation was generated by ab initio and homology based gene predictions and from short-read RNA-seq data which was followed by defining the phylogenetic position of the species and analyses of gene and gene-family evolution. The study provides a genome resource and annotation for the species and contributes to the understanding of gene family evolution for adaptation to high altitude and immunity in birds.

3-1: One of the main aims of the authors was to provide a genomic resource for the MP to support future studies of the species and this work fulfils this aim. Properties of the genome sequence (contig/scaffold N50, coverage, repeat content) is very similar to the medium quality bird genome assemblies released by the Avian Phylogenetic Consortium (Zhang G et al. 2014. Science 346: 1311-1320). The annotation approach should be sufficient and the methods used adequate to define the place of the species in the phylogeny of pheasants as it is built on orthologous peptide regions.

Nevertheless the fragmented genome assembly will limit the scope of future analyses which can be done with the assembly. Also, the annotation chiefly relies on annotation from orthologous peptides with only limited information coming from transcriptome sequencing. While it is possible to find gene family expansions and contraction events and infer adaptively evolving regions in key genes, many of the adaptations to high altitude can be assumed to happen at changes in regulatory regions modulating levels of gene expressions, neither of which is even mentioned in the study.

Response: We appreciate the favorable comments. As the reviewer pinpointed, the relation between adaptations to high altitude and changes in regulatory regions modulating levels of gene expression is not mentioned in our manuscript. In this study, we set our sights on the de novo genome assembly of the Mikado pheasant and identification of high-altitude adaptation based on genomic information. Identifying genes related to adaption from the perspective of gene expression and biologically verifying the findings of this study are all potentially interesting topics and can be set as one of our long-term goals in the research of Mikado pheasants. The following paragraph was added into the Discussion section.
"To sum up, this study reveals the high-altitude adaptation mechanisms of the Mikado pheasant at the genomic level. However, there are some adaptive mechanisms for high altitude that happen via changes in regulatory regions modulating the levels of gene expression [64-66]. We believe that this is an intriguing topic and worthy of further research to be undertaken in the future."

3-2: My main concern is with the part of the paper which describes the observed differences in the MHC region between the MP and the gal4 chicken assembly. It is known that chr16 of the gal4 assembly contained errors. Unfortunately the authors failed to mention the presence of these errors and how these would affect their results. Chr 16 has got improved in the gal5 assembly (Warren WC et al. 2016 G3 (Bethesda) 7: 109-117.) and the improved sequence would/could have provided a much better reference for this comparison. If, for this part of the work, the authors would realign the MHC region between MP and chr16 of gal5 that would make their results more reliable and relevant for the bird communities.

Response: We appreciate the suggestion and apologize for the confusion. Instead of the chr16 sequence from the gal4 or gal5 genome references, the chicken MHC-B sequence that we used to compare with the Mikado pheasant was obtained from GenBank (AB268588.1). The sequence was published by Shiina et al. and analyzed DNA molecules from constructed bacterial artificial chromosome (BAC) clones and long-PCR products by Sanger sequencing technology [17]. To avoid the confusion, we have rephrased the sentence as "..., an assembled scaffold (scaffold208) was almost able to cover the known chicken sequence of the MHC-B contiguous region published by Shiina et al. (GenBank Accession: AB268588.1)." However, out of respect for the reviewer's comment we also realigned the MHC region between the Mikado pheasant and chr16 of gal5 (NC_006103.4). As shown in Fig. R2, except for the strand orientation, the alignment showed identical results between the AB268588.1 sequence and chr16 of gal5, which proved that our results are reliable.

Apart from the above I found the manuscript generally well written and I only have a few small comments:

3-3: I assumed to find tissue information for the samples from which the genomic short read and RNA-seq data was generated, but could not find it in the materials and methods section (MM).

Response: We apologize for the confusion. Originally, the information on tissue samples was at the beginning of the “De novo genome assembly” paragraph in the Materials and Methods section. To avoid the confusion, the paragraph has been split into an independent paragraph and titled “Sample preparation and sequencing.”

3-4: A technical note: TopHat2 was shown to underperform most of the other RNA-seq read mapping softwares (e.g. STAR). As the RNA-seq data is limited and the genome is fragmented the limitations coming from the usage of a "weaker" aligner is probably not that significant for this study.

Response: We appreciate the suggestion. Compared with TopHat2, the mapping rates using STAR (version 2.4.0) with default settings were significantly improved in both of the RNA-Seq samples (Table R2). We have replaced the TopHat2 results with STAR results for the assembly assessment in the revised manuscript.

Table R2: Comparison of RNA read mapping rates using STAR and TopHat2.

	RNA Sample 1	RNA Sample 2
STAR		
Total mapped	95.8%	93.1%
Multiple Mapped	2.04%	2.04%
Uniquely Mapped	93.8%	91.1%
TopHat2		
Mapped concordantly	88.1%	72.4%
Overall mapping rate	91.9%	80.9%

There were a few sentences which I found hard to understand:

3-5: P9: L229. "First, 15 161 Mikado pheasant genes were identified in 18 220 families, and 5287 single-gene families that were common across the 10 species were then used to construct a Bayesian maximum clade credibility phylogenetic tree to estimate the time of divergence"
Do you mean 15,161 genes in 18,220 families? Did you have genes belonging to multiple gene families?

Response: 1) We apologize for the confusion, the 18 220 gene families in total were obtained from the 10 species. There were 15 161 genes of the Mikado pheasant were grouped into 12 549 gene families in the analysis of the 10 different species (please refer to Gene families in the Materials and Methods section). To clarify the statement, we have rephrased the sentence at Reviewer #2’s suggestion.
2) No, we performed OrthoMCL to classify gene families. The tool assigned a gene to a gene family.

3-6: P20: L514." Regarding the RNA reads, the mapping rate showed the completeness of the final assembly with respect to the independent sequencing data from the transcriptomes of the Mikado pheasant."

Response: We apologize for the confusion. The sentence has been rephrased as: “Taking the RNA sequencing reads from two individual Mikado pheasants and observing the mapping rate is another approach for assessing the completeness of the assembly.”

References:

1. Wang N, Kimball RT, Braun EL, Liang B and Zhang Z. Assessing phylogenetic relationships among galliformes: a multigene phylogeny with expanded taxon sampling in Phasianidae. PLoS One. 2013;8 5:e64312. doi:10.1371/journal.pone.0064312.
2. Le Duc D, Renaud G, Krishnan A, Almen MS, Huynen L, Prohaska SJ, et al. Kiwi genome provides insights into evolution of a nocturnal lifestyle. Genome biology. 2015;16:147. doi:10.1186/s13059-015-0711-4.
3. Hooper DM and Price TD. Chromosomal inversion differences correlate with range overlap in passerine birds. Nat Ecol Evol. 2017;1 10:1526-34. doi:10.1038/s41559-017-0284-6.
4. Aslam ML, Bastiaansen JW, Crooijmans RP, Vereijken A, Megens HJ and Groenen MA. A SNP based linkage map of the turkey genome reveals multiple intrachromosomal rearrangements between the turkey and chicken genomes. BMC Genomics. 2010;11:647. doi:10.1186/1471-2164-11-647.
5. Zhang G, Li C, Li Q, Li B, Larkin DM, Lee C, et al. Comparative genomics reveals insights into avian genome evolution and adaptation. Science. 2014;346 6215:1311-20. doi:10.1126/science.1251385.
6. Volker M, Backstrom N, Skinner BM, Langley EJ, Bunzey SK, Ellegren H, et al. Copy number variation, chromosome rearrangement, and their association with recombination during avian evolution. Genome research. 2010;20 4:503-11. doi:10.1101/gr.103663.109.
7. Ellegren H. Molecular evolutionary genomics of birds. Cytogenet Genome Res. 2007;117 1-4:120-30. doi:10.1159/000103172.
8. Davis JK, Mittel LB, Lowman JJ, Thomas PJ, Maney DL, Martin CL, et al. Haplotype-based genomic sequencing of a chromosomal polymorphism in the white-throated sparrow (Zonotrichia albicollis). J Hered. 2011;102 4:380-90. doi:10.1093/jhered/esr043.
9. Prum RO, Berv JS, Dornburg A, Field DJ, Townsend JP, Lemmon EM, et al. A comprehensive phylogeny of birds (Aves) using targeted next-generation DNA sequencing. Nature. 2015;526 7574:569-73. doi:10.1038/nature15697.
10. Jarvis ED, Mirarab S, Aberer AJ, Li B, Houde P, Li C, et al. Whole-genome analyses resolve early branches in the tree of life of modern birds. Science. 2014;346 6215:1320-31. doi:10.1126/science.1253451.
11. Zhang G, Li B, Li C, Gilbert MT, Jarvis ED, Wang J, et al. Comparative genomic data of the Avian Phylogenomics Project. Gigascience. 2014;3 1:26. doi:10.1186/2047-217X-3-26.
12. Han MV, Thomas GW, Lugo-Martinez J and Hahn MW. Estimating gene gain and loss rates in the presence of error in genome assembly and annotation using CAFE 3. Mol Biol Evol. 2013;30 8:1987-97. doi:10.1093/molbev/mst100.
13. Ye Q, He K, Wu SY and Wan QH. Isolation of a 97-kb minimal essential MHC B locus from a new reverse-4D BAC library of the golden pheasant. PLoS One. 2012;7 3:e32154. doi:10.1371/journal.pone.0032154.
14. Chaves LD, Krueth SB and Reed KM. Defining the turkey MHC: sequence and genes of the B locus. J Immunol. 2009;183 10:6530-7. doi:10.4049/jimmunol.0901310.
15. Hosomichi K, Shiina T, Suzuki S, Tanaka M, Shimizu S, Iwamoto S, et al. The major histocompatibility complex (Mhc) class IIB region has greater genomic structural flexibility and diversity in the quail than the chicken. BMC Genomics. 2006;7:322. doi:10.1186/1471-2164-7-322.
16. Wang B, Ekblom R, Strand TM, Portela-Bens S and Hoglund J. Sequencing of the core MHC region of black grouse (Tetrao tetrix) and comparative genomics of the galliform MHC. BMC Genomics. 2012;13:553. doi:10.1186/1471-2164-13-553.
17. Shiina T, Briles WE, Goto RM, Hosomichi K, Yanagiya K, Shimizu S, et al. Extended gene map reveals tripartite motif, C-type lectin, and Ig superfamily type genes within a subregion of the chicken MHC-B affecting infectious disease. J Immunol. 2007;178 11:7162-72.

Close
