# Supplementary material for: Whole-genome de novo sequencing reveals unique genes that contributed to the adaptive evolution of the Mikado pheasant
Source: Gigascience. 2018 May 2;7(5):giy044. doi: 10.1093/gigascience/giy044 (PMC5941149; doi:10.1093/gigascience/giy044)
Supplement: Reviewer_1_Report_(Original_Submission) -- Matthew Greenwold — 10/11/2017 Reviewed [file giy044_reviewer_1_report_(original_submission).pdf]

Reviewer Report

Title: Whole-Genome De Novo Sequencing Reveals Unique Genes that Contributed to the Adaptive Evolution of the Mikado Pheasant

Version: Original Submission **Date:** 10/11/2017

Reviewer name: Matthew Greenwold

Reviewer Comments to Author:

The manuscript entitled "Whole-Genome De Nova Sequencing Reveals Unique Genes that Contributed to the Adaptive Evolution of the Mikado Pheasant" utilizes the nuclear and mitochondrial genomes to identify genes related to adaptation and immunity. Furthermore, they use nuclear genome genes to reconstruct the phylogenetic position of the Mikado Pheasant among birds with sequenced genomes and estimate the divergence time using mitochondrial genomes of long-tailed pheasants. The manuscript is in very good shape and I have mostly minor comments (see below). Generally, I thought the Results section could be better presented, but the Discussion section was very well written and really brings the significance of these findings to light.

Major comments

One critical issue I had with the results was the use of separate analyses for the identification of PSGs - 5 vs. 50 species. Can you remove one of these analyses? Or if you decide to retain both, I think a couple of statements about how many PSGs and GO terms overlap and a explanation for their use is required.

Also, for the divergence time estimate using mitochondrial genomes - Is there any nuclear genome data for the other long-tailed pheasants that can be incorporated into this analysis? If not, please discuss how the use of only mitochondrial data may affect the divergence time estimates.

Minor comments

Abstract

Line 78: The second sentence in abstract Background is oddly worded. Please revise. A variant of this sentence is also in Background (line 117).

Line 89: "mitochondrial genome was further" would sound better written as "mitochondrial genome was ALSO"

Background

Lines 112-116: I think that genome resources for endangered species can provide great insight into effective population size. This should be added here.

Line 119: "the Syrmaticus genus and belongs" should be written as "the Syrmaticus genus and FORMS (or comprises)"

Line 131: change to "insights into its adaptive mechanisms." Remove "of the pheasant".

Results

Lines 233-234: "between 21.4 and 28.9 million years ago" The Figure has different values - 18.3-27.9. Is

the text or figure correct?

Lines 262-265: Please rewrite this first sentence as it is awkward.

Line 266: How many PSGs were identified? Please list the number in the text.

Lines 270-272: It should be noted here that PSGs enriched for metabolism constituted the highest number of PSGs as that information is buried in the supplemental files.

Lines 327-329, first sentence. The use of "Recently" seems odd as there has been significant work looking at dN/dS ratios in relationship to MHC. Please consider adding more references here and removing "Recently".

Methods

Are the methods appropriate to the aims of the study, are they well described, and are necessary controls included? Yes

Conclusions

Are the conclusions adequately supported by the data shown? Yes

Reporting Standards

Does the manuscript adhere to the journal's guidelines on minimum standards of reporting? Yes

Choose an item.

Statistics

Are you able to assess all statistics in the manuscript, including the appropriateness of statistical tests used? Yes, and I have assessed the statistics in my report.

Quality of Written English

Please indicate the quality of language in the manuscript: Acceptable

Declaration of Competing Interests

Please complete a declaration of competing interests, considering the following questions:

- Have you in the past five years received reimbursements, fees, funding, or salary from an organisation that may in any way gain or lose financially from the publication of this manuscript, either now or in the future?
- Do you hold any stocks or shares in an organisation that may in any way gain or lose financially from the publication of this manuscript, either now or in the future?

- Do you hold or are you currently applying for any patents relating to the content of the manuscript?
- Have you received reimbursements, fees, funding, or salary from an organization that holds or has applied for patents relating to the content of the manuscript?
- Do you have any other financial competing interests?
- Do you have any non-financial competing interests in relation to this paper?

If you can answer no to all of the above, write 'I declare that I have no competing interests' below. If your reply is yes to any, please give details below.

I declare that I have no competing interests.

I agree to the open peer review policy of the journal. I understand that my name will be included on my report to the authors and, if the manuscript is accepted for publication, my named report including any attachments I upload will be posted on the website along with the authors' responses. I agree for my report to be made available under an Open Access Creative Commons CC-BY license (http://creativecommons.org/licenses/by/4.0/). I understand that any comments which I do not wish to be included in my named report can be included as confidential comments to the editors, which will not be published.

I agree to the open peer review policy of the journal

To further support our reviewers, we have joined with Publons, where you can gain additional credit to further highlight your hard work (see: https://publons.com/journal/530/gigascience). On publication of this paper, your review will be automatically added to Publons, you can then choose whether or not to claim your Publons credit. I understand this statement.

Yes Choose an item.
