# Supplementary material for: Whole-genome de novo sequencing reveals unique genes that contributed to the adaptive evolution of the Mikado pheasant
Source: Gigascience. 2018 May 2;7(5):giy044. doi: 10.1093/gigascience/giy044 (PMC5941149; doi:10.1093/gigascience/giy044)
Supplement: Reviewer_2_Report_(Original_Submission) -- Aurelie Kapusta, Ph.D. — 10/30/2017 Reviewed [file giy044_reviewer_2_report_(original_submission).pdf]

Reviewer Report

Title: **Whole-Genome De Novo Sequencing Reveals Unique Genes that Contributed to the Adaptive Evolution of the Mikado Pheasant**

Version: **Original Submission** **Date:** 10/30/2017

Reviewer name: **Aurelie Kapusta, Ph.D.**

Reviewer Comments to Author:

In their study titled "Whole-Genome De Novo Sequencing Reveals Unique Genes that Contributed to the Adaptive Evolution of the Mikado Pheasant", Lee, Hsieh et al. describe a newly sequenced bird genome - which is always a good resource - including some comparative genomics studies. I believe that this work is solid and clearly explained, and as such is of interest and in the scope of GigaScience. I do have some (mostly minor) comments detailed below that I believe would increase the quality and clarity of the manuscript.

GENERAL COMMENTS:

- The use of the word "behavior" (adaptive behavior) is misleading. It seems that the authors mean it in the context of adaptive evolutionary history, and I would suggest to reformulate for accuracy (Abstract and Introduction p5 l129).
- some figures could be improved by having more information on the figure instead of in the legend (mostly Figure 3, see detailed comments below)
- The significance of some data could be improved at a few locations (see detailed comments below)

DETAILED COMMENTS:

ABSTRACT:
- The authors emphasize in the abstract the details of their data about the MHC & comparison with chicken: having less details but more scope / significance would improve the abstract.

INTRODUCTION:
- p4 l114: what does "behavioral attributes" means here?
- p4 l114-117: consider splitting this sentence.
- p5 l1: how was hypoxic stress observed? Is there any citation? Or is this an expectation/extrapolation?
- p5 l138-141: same comment as abstract.

DATA DESCRIPTION:
- Refer to the Method section at least one time at the beginning of this section.
- p6 l156-158: please revise the formulation of this sentence for clarity. Fig S4 shows that there are in fact a lot of scaffolds with short length, even if indeed most of the genome size is assembled in large scaffolds.

RESULTS:
- p7 l 176-180: was there a step to verify that the sequencing samples were not contaminated? For example, the bald eagle genome assembly (file from Zhang et al. (2014), Science) has hundreds of bacterial contigs in it (absent from the refseq version because very short), coming from 2 samples contaminated with Yersinia (SRR1176808 and SRR1176809). This can be checked quickly with some software such as Kraken or Taxonomer (with www.taxonomer.com - note that for this website, for a bird genome reads would be

nearly all unknown or ambiguous). I could not find the data on the SRA at the time of reviewing to look myself.
- p7 l180-184: unless reads were excluded when mapping at multiple locations, do (some) high coverage regions correspond to repeats?
- p8 l202: This sentence would be more clear if "with pheasant scaffolds" was added after "The identities of each chicken chromosome"
- p8 l208: if this is notable, what is the significance?
- p8 l217: as expected?
- p8 l218-221: consider having the mention of "high frequency of potentially highly conserved regions" before the "but", to contrast conservation and dynamics.
- p8 l 220: what are the "high frequency" numbers? How does this compare to the literature, if any similar other research?
- p9 l229-230: The formulation here is confusing and should be revised to illustrate better that the 18 220 gene families (as mentioned in the legend of Figure 2) are for all species considered (and not just the Mikado pheasant) - since Figure S8 shows different numers. Additionally, the number of genes is lower than the number of annotated genes mentioned in the manuscript or than the one in Figure S8; why these three different values?
- p9 l245: are fragmented annotations a possible issue here? i.e. are longer genes enriched or not in expanded families?
- p9 l246: Are the numbers / rates surprising or not based on the literature?
- p9 l248-259: what about the ones in the chicken for example? And other birds?
- p9 l258: is 8/75 surprising? What is the fraction of all olfactory receptors among all gene families? Were there more olfactory receptors annotated in the pheasant than other birds? E.g. discuss based on the data from Steiger et al 2008 (DOI: 10.1098/rspb.2008.0607), or other literature if any.
- p10 l262: this formulation is unclear: "because of living at and between high and low elevation".
- p10 l264: Since these 7132 orthologues seem to be the same as the 7132 single-gene families mentioned in Methods, the change of terminology (gene family v.s. orthologs) is confusing (maybe use orthologs for single-gene families that were also annotated as orthologs by OrthoMCL, and gene families for the others?).
- p11 l293: since the Jak-STAT pathway is not mentioned again in discussion, please add why this is worth noticing.
- p11 l301: this number of 5287 orthologs between 48 birds is identical to the one of orthologs identified in 10 species (with mammals) - please check that this is accurate.
- p12 l305: the ubiquitin activity is not mentioned in discussion: what would be the significance of having expanded gene families associated with this GO term?
- p12 l320: Methods says MAKER, not manual curation; was MAKER used and then the annotations manually curated?
- p12 l329: there is more general literature on this question (e.g. Harmit Malik's work and others); adding one or two references would strengthen this point.
- p13 l330: BLB2 is mentioned here (probably because found in RNAseq data?), but it is is missing from the Figure and afterwards said missing from the Mikado pheasant assembly, which is confusing. Maybe the lines 445 to 451 should be part of this result section instead?
- p13 l331: see comment about Figure 3
- p13 l240: significance of inversions?

DISCUSSION:
- p14 l375: since these extra steps are not detailed in the Method section, the parameters and versions should figure in Table S18 or in additional info.
- p15 l387: how were the number of misassembled or fragmented sequences estimated and distinguished from real differences with the chicken genome (since Fig1 is referred to)?
- p17 l438-441: is there any evidence that these inversions affect the expression of these genes...?
- p17 l445-451: see comment for p13 l330.
- p17 l452: "the whole genome of a genus" would read better as "the whole genome of a bird of the genus"

MATERIAL AND METHODS:

- p19 l486: were both experiments done on pooled RNA from the 2 males, or was there one male per RNAseq experiment?
- p19 l494: FastQC version and exact tools and parameters used to trim reads and remove adapters?
- p19 l496: MaSuRCA reference missing here (even if elsewhere in the ms): Zimin, A. et al. Bioinformatics (2013). doi:10.1093/bioinformatics/btt476
- p20 l509: were the software's default parameters used? What were the parameters regarding non uniquely mapping reads?
- p10 l513: Version of BEDTools is missing.
- p21 l533: RepeatMasker version, parameters and library used are missing.
- p22 l565: consider adding the numbers of genes and gene families identified that are not single genes before switching to the Method of ortholog identification.
- p22 l573: bootstraps?

FIGURES:
FIGURE 1B:
- point/label differently on the figure scaffolds 1 and 45 (since mentioned in the text), and maybe also the ones that fully align to chicken chromosomes?
FIGURE2:
- consider adding numbers per My, to facilitate the comparison between branches.
FIGURE3:
- it would help the reader a lot if instead of having a color code for forward/reverse (that information is already coded by the position above or under the bar), the genes could be color coded based on their dN/dS ratios.
- the coverage scale does not allow to see lower coverage genes; consider using a log scale?

SUPP DATA:
FIGURE S2: does 'habitats available' correspond to where they are found generally, or a protected habitat?
FIGURE S8: see comment for Results p9 l230.
TABLE S1: which RNAseq was HiSeq and which was HiSanSQ?
TABLE S9: for ex. for the GO:0002504 line, could these annotations be missing from the assembly or be fragmented? Since there are only 2 genes in this family, it sounds possible.
TABLE S18: see comment about Discussion p14 l375.

Methods

Are the methods appropriate to the aims of the study, are they well described, and are necessary controls included? Yes

Conclusions

Are the conclusions adequately supported by the data shown? Yes

Reporting Standards

Does the manuscript adhere to the journal's guidelines on minimum standards of reporting? No

Choose an item.

Statistics

Are you able to assess all statistics in the manuscript, including the appropriateness of statistical tests used? There are no statistics in the manuscript.

Quality of Written English

Please indicate the quality of language in the manuscript: Acceptable

Declaration of Competing Interests

Please complete a declaration of competing interests, considering the following questions:

- Have you in the past five years received reimbursements, fees, funding, or salary from an organisation that may in any way gain or lose financially from the publication of this manuscript, either now or in the future?
- Do you hold any stocks or shares in an organisation that may in any way gain or lose financially from the publication of this manuscript, either now or in the future?
- Do you hold or are you currently applying for any patents relating to the content of the manuscript?
- Have you received reimbursements, fees, funding, or salary from an organization that holds or has applied for patents relating to the content of the manuscript?
- Do you have any other financial competing interests?
- Do you have any non-financial competing interests in relation to this paper?

If you can answer no to all of the above, write 'I declare that I have no competing interests' below. If your reply is yes to any, please give details below.

I declare that I have no competing interests

I agree to the open peer review policy of the journal. I understand that my name will be included on my report to the authors and, if the manuscript is accepted for publication, my named report including any attachments I upload will be posted on the website along with the authors' responses. I agree for my report to be made available under an Open Access Creative Commons CC-BY license (http://creativecommons.org/licenses/by/4.0/). I understand that any comments which I do not wish to be included in my named report can be included as confidential comments to the editors, which will not be published.

I agree to the open peer review policy of the journal

To further support our reviewers, we have joined with Publons, where you can gain additional credit to further highlight your hard work (see: https://publons.com/journal/530/gigascience). On publication of this paper, your review will be automatically added to Publons, you can then choose whether or not to claim your Publons credit. I understand this statement.

Yes Choose an item.
