# Supplementary material for: Whole-genome de novo sequencing reveals unique genes that contributed to the adaptive evolution of the Mikado pheasant
Source: Gigascience. 2018 May 2;7(5):giy044. doi: 10.1093/gigascience/giy044 (PMC5941149; doi:10.1093/gigascience/giy044)
Supplement: Reviewer_3_Report_(Original_Submission) -- Lél Eöry — 11/1/2017 Reviewed [file giy044_reviewer_3_report_(original_submission).pdf]

Reviewer Report

Title: Whole-Genome De Novo Sequencing Reveals Unique Genes that Contributed to the Adaptive Evolution of the Mikado Pheasant

Version: Original Submission **Date:** 11/1/2017

Reviewer name: Lél Eöry

Reviewer Comments to Author:

The manuscript presents the genome and gene annotation of the Mikado pheasant (MP), a protected species living in geographical isolation and adapted to high altitude habitats. The genome was assemble into 208.8k contigs (>300 bp) and 9,359 scaffolds (>1 kb) using Illumina short read technology of paired-end and mate-pair libraries. Annotation was generated by ab initio and homology based gene predictions and from short-read RNA-seq data which was followed by defining the phylogenetic position of the species and analyses of gene and gene-family evolution. The study provides a genome resource and annotation for the species and contributes to the understanding of gene family evolution for adaptation to high altitude and immunity in birds.

One of the main aims of the authors was to provide a genomic resource for the MP to support future studies of the species and this work fulfils this aim. Properties of the genome sequence (contig/scaffold N50, coverage, repeat content) is very similar to the medium quality bird genome assemblies released by the Avian Phylogenetic Consortium (Zhang G et al. 2014. Science 346: 1311-1320). The annotation approach should be sufficient and the methods used adequate to define the place of the species in the phylogeny of pheasants as it is built on orthologous peptide regions. Nevertheless the fragmented genome assembly will limit the scope of future analyses which can be done with the assembly. Also, the annotation chiefly relies on annotation from orthologous peptides with only limited information coming from transcriptome sequencing. While it is possible to find gene family expansions and contraction events and infer adaptively evolving regions in key genes, many of the adaptations to high altitude can be assumed to happen at changes in regulatory regions modulating levels of gene expressions, neither of which is even mentioned in the study.

My main concern is with the part of the paper which describes the observed differences in the MHC region between the MP and the gal4 chicken assembly. It is known that chr16 of the gal4 assembly contained errors. Unfortunately the authors failed to mention the presence of these errors and how these would affect their results. Chr 16 has got improved in the gal5 assembly (Warren WC et al. 2016 G3 (Bethesda) 7: 109-117.) and the improved sequence would/could have provided a much better reference for this comparison. If, for this part of the work, the authors would realign the MHC region between MP and chr16 of gal5 that would make their results more reliable and relevant for the bird communities.

Apart from the above I found the manuscript generally well written and I only have a few small comments:

I assumed to find tissue information for the samples from which the genomic short read and RNA-seq data was generated, but could not find it in the materials and methods section (MM).

A technical note: TopHat2 was shown to underperform most of the other RNA-seq read mapping softwares (e.g. STAR). As the RNA-seq data is limited and the genome is fragmented the limitations coming from the usage of a "weaker" aligner is probably not that significant for this study.

There were a few sentences which I found hard to understand:

P9: L229. "First, 15 161 Mikado pheasant genes were identified in 18 220 families, and 5287 single-gene

families that were common across the 10 species were then used to construct a Bayesian maximum clade credibility phylogenetic tree to estimate the time of divergence"
Do you mean 15,161 genes in 18,220 families? Did you have genes belonging to multiple gene families?

P20: L514." Regarding the RNA reads, the mapping rate showed the completeness of the final assembly with respect to the independent sequencing data from the transcriptomes of the Mikado pheasant."

Methods

Are the methods appropriate to the aims of the study, are they well described, and are necessary controls included? Yes

Conclusions

Are the conclusions adequately supported by the data shown? No

Reporting Standards

Does the manuscript adhere to the journal's guidelines on minimum standards of reporting? Yes

Choose an item.

Statistics

Are you able to assess all statistics in the manuscript, including the appropriateness of statistical tests used? Yes, and I have assessed the statistics in my report.

Quality of Written English

Please indicate the quality of language in the manuscript: Acceptable

Declaration of Competing Interests

Please complete a declaration of competing interests, considering the following questions:

- Have you in the past five years received reimbursements, fees, funding, or salary from an organisation that may in any way gain or lose financially from the publication of this manuscript, either now or in the future?
- Do you hold any stocks or shares in an organisation that may in any way gain or lose financially from the publication of this manuscript, either now or in the future?
- Do you hold or are you currently applying for any patents relating to the content of the manuscript?
- Have you received reimbursements, fees, funding, or salary from an organization that holds or has applied for patents relating to the content of the manuscript?

- Do you have any other financial competing interests?
- Do you have any non-financial competing interests in relation to this paper?

If you can answer no to all of the above, write 'I declare that I have no competing interests' below. If your reply is yes to any, please give details below.

I declare that I have no competing interests

I agree to the open peer review policy of the journal. I understand that my name will be included on my report to the authors and, if the manuscript is accepted for publication, my named report including any attachments I upload will be posted on the website along with the authors' responses. I agree for my report to be made available under an Open Access Creative Commons CC-BY license (http://creativecommons.org/licenses/by/4.0/). I understand that any comments which I do not wish to be included in my named report can be included as confidential comments to the editors, which will not be published.

I agree to the open peer review policy of the journal

To further support our reviewers, we have joined with Publons, where you can gain additional credit to further highlight your hard work (see: https://publons.com/journal/530/gigascience). On publication of this paper, your review will be automatically added to Publons, you can then choose whether or not to claim your Publons credit. I understand this statement.

Yes Choose an item.
